# Investigating the biomarkers of diabetic-cardiomyopathy with the high mobility group box-1 as a potential anti-inflammatory therapeutic target: Systematic Review and meta-analysis

**DOI:** 10.3389/fendo.2025.1714219

**Published:** 2026-01-14

**Authors:** Ranmali Ranasinghe, Michael Mathai, Anthony Zulli

**Affiliations:** Institute for Health and Sport, Victoria University, Melbourne, VIC, Australia

**Keywords:** cardiomyopathy, diabetes, endothelium, HMGB1, inflammation, meta-analysis, rodent models, systematic review

## Abstract

**Aim:**

The aim was to carry out a preliminary investigation to identify new biomarkers and test the suitability of the pro-inflammatory nuclear protein, HMGB1, as a potential diagnostic or treatment target for DCM.

**Background:**

Diabetic cardiomyopathy (DCM) is a complex metabolic disease group which manifests in persons diagnosed with poorly managed *Diabetes mellitus*. This study investigates whether HMGB1 is capable of attenuating the inflammation that manifests from DCM in pre-clinical models of mouse and rat combined.

**Methodology:**

A systematic review and a meta-analysis were performed by searching 5 electronic databases and retrieving 2979 articles from which 29 qualified as included studies for reporting 37 biomarkers that were grouped into 8 preclinical DCM biomarker models. The standardized mean difference (SMD or the effect size), non-parametric Mann Whitney U test, ROC, correlation coefficient and coefficient of determination were carried out in this evaluation.

**Results:**

28 heterogeneous proinflammatory biomarkers were identified as carrying a significantly high risk of developing DCM out of the total of 37 biomarkers evaluated in forest plots in which, the highest SMD was produced by cardiac troponin (CTPN). 8 significantly high biomarkers (HMGB1, HW/BW, EF%, FS%, BG, TC, TG, NF-kB) were identified out of 37 in the non-parametric Mann Whitney U test in the DCM group compared to the HC. The correlation coefficient between HMGB1 as the independent variable produced a significant negative (ecological) correlation with HR, EF% and TLR4 at p < 0.05.

**Conclusion:**

The ability of HMGB1 in downregulating inflammation or the direct inhibition of HMGB1 using small molecules or blocking of HMGB1/TLR4/NF-kB signalling pathway could be a novel potential mechanism to resolving DCM which requires further investigations.

**Systematic review registration:**

https://www.crd.york.ac.uk/prospero/, identifier CRD42024597641.

## Highlights

- 28 out of the 37 biomarkers were identified as carrying high risk for manifesting DCM in a mouse and rat rodent model, based on a forest plot and the Cohen’s D calculation.
- The biomarkers of the highest 18 significant effect sizes in descending order of the SMD that showed significant changes between the DCM group and the healthy control were CTPN, TG, BG, TC, LDH, IL-6, AGEs, MDA, HW, GSH, IL-1β, HW/BW, CC3, NLRP3, CK-MB, FB%, NF-KB, HMGB1.
- Cardiac troponin is featured as the best non-traditional biomarker which had the highest effect size (SMD 37.93 at *p* = 0.03) and provided the potentially best therapeutic or diagnostic target for DCM. (The selection criteria included data reported from an equal to or greater than 3 studies and equal to or more than 20 DCM animals).
- According to the Mann Whitney U test results, the best biomarkers are the ones which recorded zero at p<0.05, which were HMGB1, HW/BW, EF%, FS%, BG, TC, TG, and NF-kB.
- The HR, EF% and TLR4 cardiometabolic biomarkers showed a significant negative correlation with HMGB1 indicating that with increasing HMGB1 titres in body fluids, the heart rate, ejection fraction percentage and toll-like receptor -4 levels were downregulated.
- It is suggested that the pathway to resolving DCM may include downregulating inflammation as evidenced by the elevation of pro-inflammatory cytokine markers: TNF-A, IL-6, IL-1β including HMGB1 that upregulate inflammation *via* the HMGB1/TLR4/NF-kB molecular signalling pathway.

## 1 Introduction

### 1.1 Background

Diabetic cardiomyopathy (DCM) is a group of heterogenous heart diseases which adversely affect the morphological and functional integrity of the heart (1) and manifests from the metabolic dysfunction arising due to the mismanagement of *Diabetes mellitus* (2). As the name implies, DCM is mainly a group of myocardial diseases, all of which collectively display cardiac remodelling over time that could end in heart failure (3). Therefore, DCM is a major adverse outcome of diabetes which could develop in any age group of diabetic patients that exposes them to premature mortality (4).

### 1.2 Description of the condition

DCM was first discovered in 1972 followed by the Framingham heart study in 1974 (4), which confirmed a 5-fold risk in females and a 2.4 fold risk in males, diagnosed with diabetes in the absence of confounding risk factors such as the age, hypertension and coronary heart disease (5). In 2013, the European Association for the Study of Diabetes in collaboration with the American Heart Association and several other authoritative bodies defined DCM as a clinical condition which affected the ventricular function also in the absence of coronary atherosclerosis and hypertension in persons diagnosed with *Diabetes mellitus* (6).

DCM is mostly diagnosed with performing an echocardiogram and has a prevalence of I in 250–400 or a 17% among the diabetic populations (7, 8). The influence of hyperglycaemia on the pathogenicity of DCM is still poorly defined. Not only hyperglycaemia but also lipid peroxidation, oxidative stress, apoptosis, and fibrosis contribute towards inducing myocardial inflammation (9).

### 1.3 Description of the intervention

HMGB1 is a non-histone chromosomal nuclear protein which is abundant within the nucleosomes that also serves as a ligand for the RAGE molecules (10). It performs distinct functions in the nucleus and the cytoplasm and carries out divergent functions depending on its subcellular location (11). The main activities of HMGB1 are gene transcription, maintaining nucleosome structure and upregulating inflammation by acting as a pro-inflammatory cytokine which also serves as a damage -associated molecular pattern molecule or a DAMP molecule that produces innate immune responses (12, 13). It relocates from the nucleus to the cytoplasm in response to cellular stress signals (14). The cellular oxidative stress and autophagy that arises due to uncontrolled hyperglycaemia and dyslipidaemia could also result in damaging nuclear and mitochondrial DNA, inducing interstitial inflammation, and collagen accumulation in the diabetic heart and vasculature (15, 16).

### 1.4 How the intervention might work

**Figure 1:**
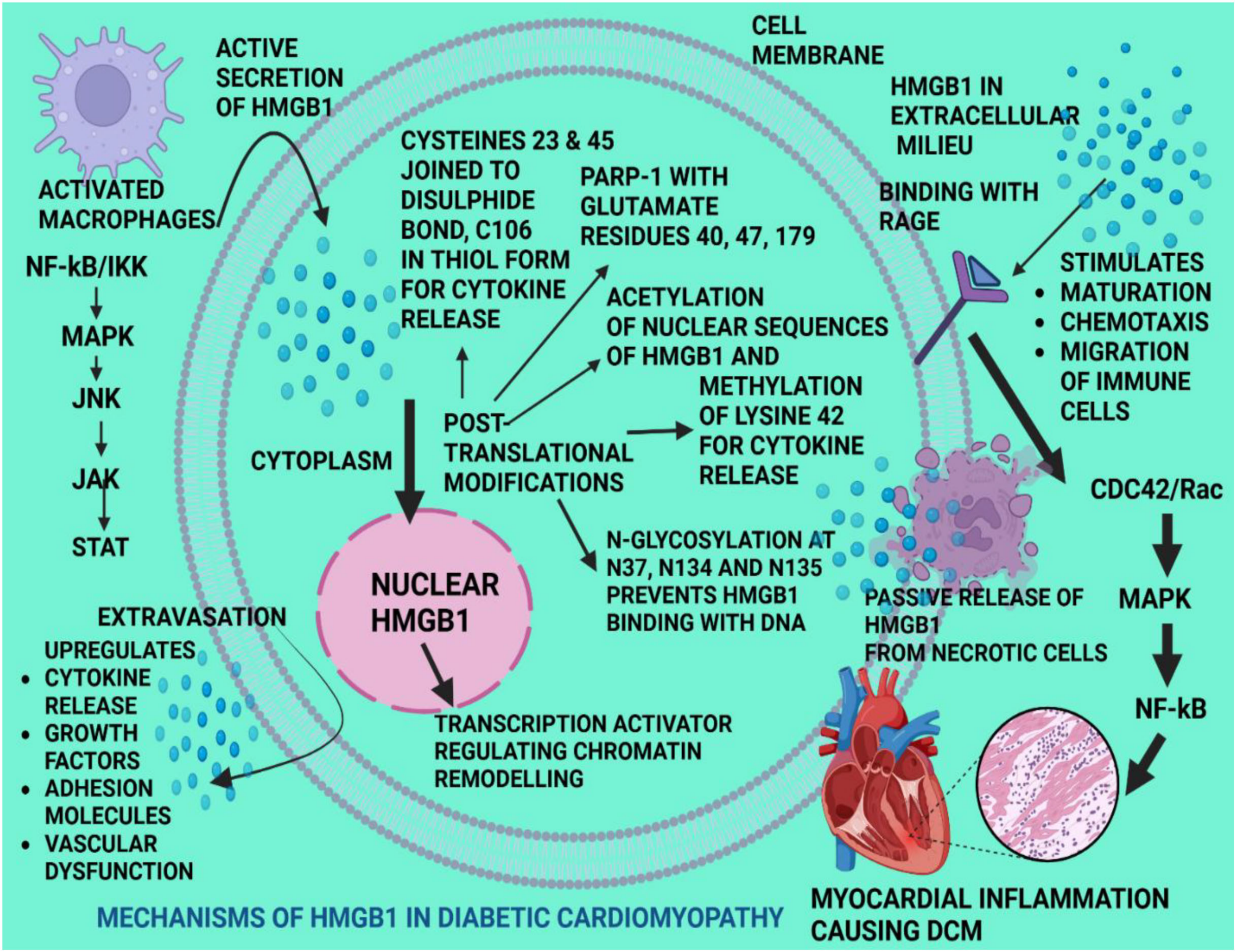
Mechanisms of HMGB1 in diabetic cardiomyopathy. HMGB1 is a highly evolutionarily conserved nuclear protein which is ubiquitous in nature, being in the nucleus, cytoplasm or extracellular matrix, and performing different functions depending on its subcellular location. Nuclear HMGB1 performs DNA repair processes and activates transcription factors such as NF-KB, MAPK, JNK, JAK and STAT. HMGB1 is actively secreted by activated immune cells such as the macrophages, monocytes and dendritic cells. It is passively released from necrotic, pyroptotic and apoptotic cells. In the cell cytoplasm, HMGB1 induces post-translational modifications resulting in cytokine release, growth factors and adhesion molecular release, leading to vascular endothelial dysfunction. HMGB1 in extracellular milieu, stimulates maturation, chemotaxis and migration of immune cells and the upregulation of inflammatory signalling cascades producing myocardial inflammation and remodelling. Legend: NF-KB, nuclear factor kappa beta; MAPK, mitogen-activated protein kinase; JNK, c-Jun N-terminal kinase; JAK, janus kinase; HMGB1, high mobility group box-1; STAT, signal transducer and activator of transcription; CDC42/Rac, Cdc42-interacting protein 4 (CIP4)/small GTPase molecules; PARP, poly ADP -ribose polymerase; RAGE, receptor for advanced glycation end products. (Created with Biorender.com).

There are many ways in which HMGB1 could exert anti-inflammatory effects to attenuate the severity and complexity of DCM. Modifying the redox state of the HMGB1 molecules by switching from oxidation to reduction status helps in the transformation of pro-inflammatory properties to anti-inflammatory conditions (17). The specific binding affinities of HMGB1 with several other molecules could trigger anti-inflammatory activity by stimulating the release of cytokines such as IL-10 or interacting with signalling cascades such as Nrf2/HMOX1 which could produce small molecules that are self-inhibitory to the pro-inflammatory HMGB1, similar to glycyrrhizin. Enzymatic cleavage by cathepsin G could rapidly cleave and remove HMGB1 from certain inflammatory environments, or could block the action of the NF-KB activity by interacting with receptors that engage in anti-inflammatory action such as CD24 and siglec-10 (18) (**Figure 1**).

Myocarditis or myocardial inflammation could be attenuated by the HMGB1 protein, which promotes inflammation and in turn upregulates adaptive immunity (19). HMGB1 induces its inflammatory potential by binding with RAGE, its main binding partner for internalization, and also with TLR4, for which HMGB1 acts as a ligand (20). These interactions occurred in experimental autoimmune myocarditis that was induced by cardiac troponin I treatment as well as using HMGB1 or RAGE -knockout wild-type mice or with anti-HMGB1 antibody treatment (21). Further, HMGB1 was overexpressed both locally and systemically after immunization with cardiac troponin I enzyme in mice. Overall, cardiac tissue injury could be attenuated and initiate tissue repair and remodelling by extracellular HMGB1 using glycyrrhizin which is HMGB1’s main blocking agent (22). These findings have remained true in both preclinical and clinical models, displaying that HMGB1, RAGE and TLR4 molecules provide prospective therapeutic targets in DCM.

### 1.5 Why is it important to do this review

The significance of DCM is premature heart failure, which occurs in 30 to 40% of individuals while patients remain asymptomatic until the condition is worsened (23). Cardiomyopathy can manifest in any age group or gender (24). The DCM patients develop heart failure and death up to 55.9% in the first 5 years of diagnosing DCM (25). For those having had diabetes for 15 years, the likelihood of developing heart failure and its mortality rate is 65.8% (26). The Australian men diagnosed with DCM are more prone to developing DCM than their female counterparts (27) because of the lack of consensus in defining criteria for DCM and having poor prognosis. Considering all these facts, there remains an urgent need for producing effective diagnostic criteria and treatment strategies for DCM. Therefore, defining the biomarkers and identifying HMGB1 as a possible inflammatory target in DCM are reasons for undertaking this study.

### 1.6 Objectives

- To identify and assess the prominent biomarkers of *Diabetes mellitus* -induced cardiomyopathy.
- To investigate the influence of the HMGB1 nuclear protein on the aforesaid biomarkers.

## 2 Results

### 2.1 Description of the studies

#### 2.1.1 Search strategy

The search strategy consisted of searching the databases with keywords after specifying the year range. The databases were accessed *via* the Victoria University’s library resources webpage. The keywords consisted of:

- Diabetic cardiomyopathy AND mouse AND/OR rat AND HMGB1
- Diabetes AND cardiomyopathy AND mouse AND high mobility group box-1
- T2D AND rodent model AND HMGB1
- Diabetes mellitus AND cardiomyopathy AND mouse OR rat AND hmgb1
- Myocardial fibrosis AND mouse AND rat AND HMGB1
- HMGB1 AND type-2 diabetes AND mouse AND rat AND heart dysfunction/fibrosis
- Type-1 diabetes AND hmgb1 AND rat AND mouse
- T2D AND T1D AND RAT AND MOUSE AND HMGB1
- Myocardial infarction/reperfusion injury AND mouse/Rat AND hmgb1

These searches were carried out during the whole of year 2023. Certain searches have also yielded no results at certain times.

#### 2.1.2 Results of the search

**Figure 2:**
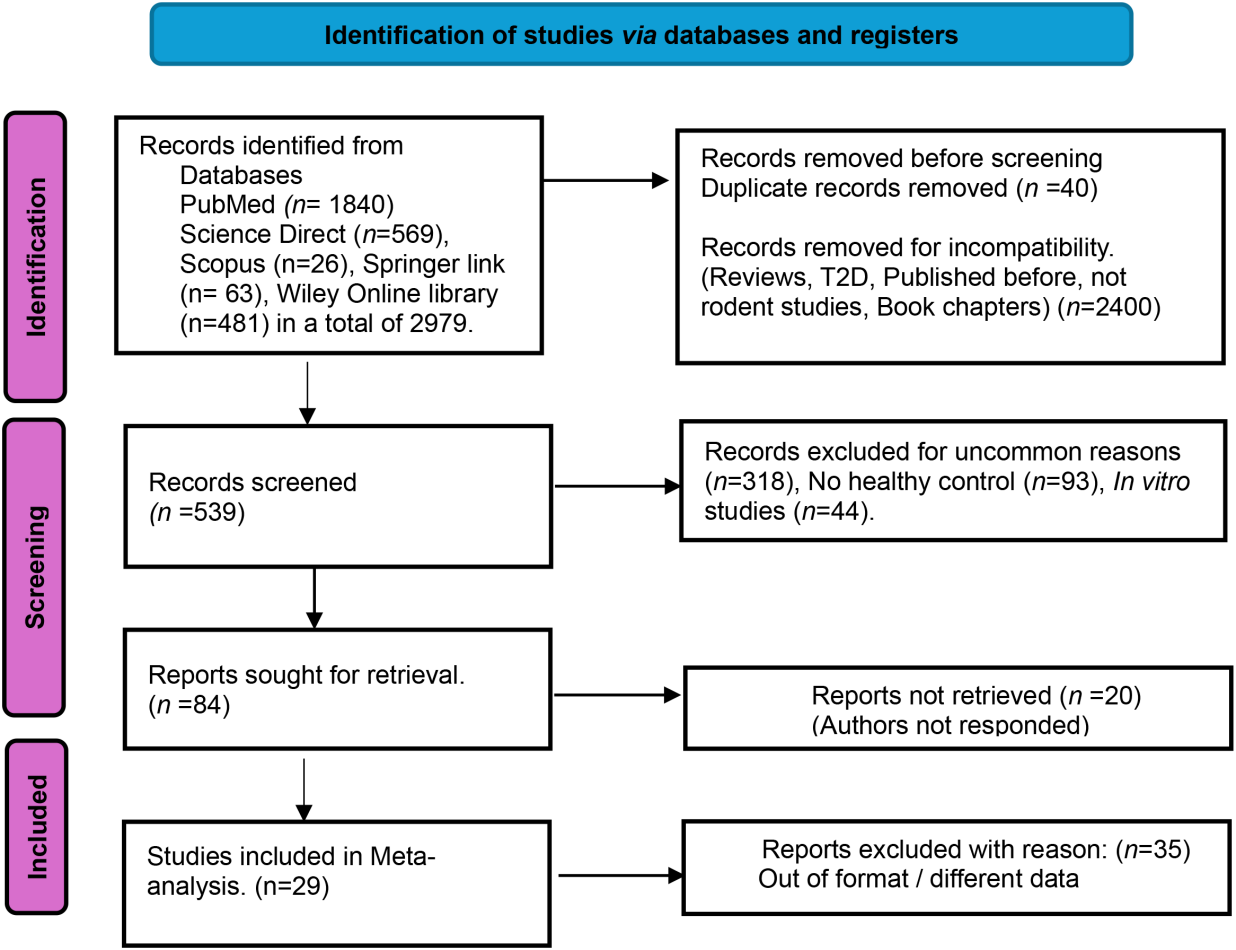
A modified version of the PRISMA flowchart of year 2020 used for reporting the included studies, (Adopted from the *Cochrane Collaboration. Org*). This is a flowchart which describes the screening process of the databases with selected keywords to obtain the relevant research articles that fulfill predetermined inclusion and exclusion criteria. The inclusion criteria consisted of only research articles, published within the applied year range, published in English, only *in vivo* mouse and rat models in the age range of 8–14 weeks and body weights not exceeding 30g or 250 g for mouse and rat respectively. Mouse and rat that were included had DCM induced by STZ/High fat diet model and had matched healthy controls.

The electronic databases that were searched included the PubMed, Science Direct, SCOPUS, Springer Link and the Wiley online library from which 2979 potentially relevant documents were obtained during the primary search. Among those records were collectively 2400 publications that included duplicates, reviews, animal studies other than of rodents and other irrelevant publications. The document search that was carried out is presented in **Figure 2**.

#### 2.1.3 Included studies

**Figure 3:**
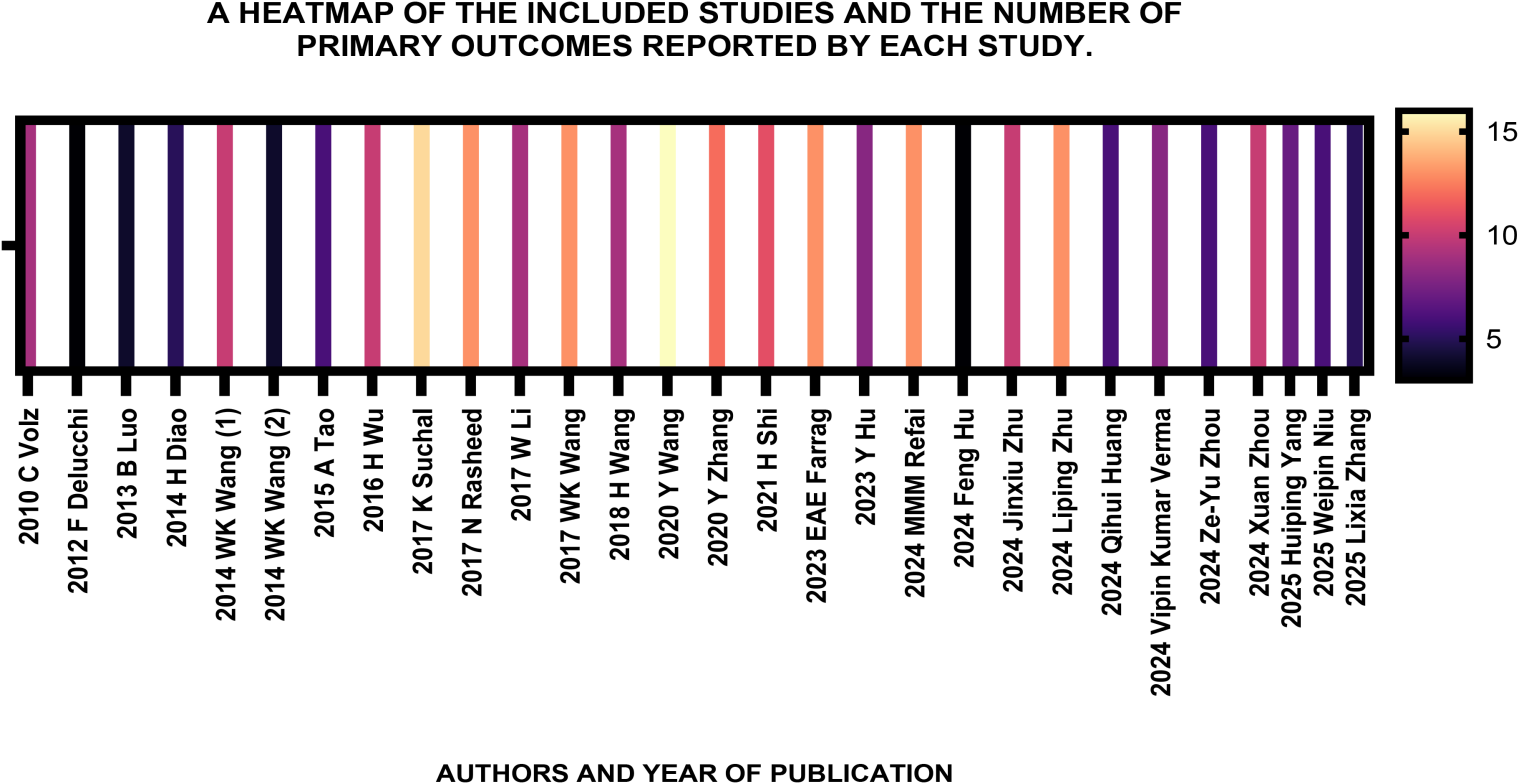
The heatmap of the 29 included studies and the number of primary outcomes evaluated by each study. This systematic review and meta-analysis included research articles published between 2010 to early 2025, given that an update up to 300 articles was done on PubMed recently and the total included studies increased from 19 to 29. The newly added studies also had some of the 37 biomarkers tested and reported. The highest number of articles that were identified, belonged to the year 2024. On average, a single study had evaluated up to 10–15 biomarkers as per this heatmap.

A total of 29 studies fulfilled the eligibility criteria and were included in this review: Volz, HC 2010, Delucchi, F 2012, Luo, B 2013, Diao, H 2014, Wang, WK-January 2014, Wang, WK-July 2014, Tao, A 2015, Wu, H 2016, Suchal, K 2017, Al-Rasheed, NM 2017, Li, WF 2017, Wang, WK 2017, Wang, HW 2018, Wang, Y 2020, Zhang, Y 2020, Shi, H 2021, Farrag, EAE 2023, Hu, Y 2023, Refaie, MMM 2024, Hu, F 2024, Zhu, J 2024, Zhu, L 2024, Huang, Q 2024, Verma, VK 2024, Zhou, X 2024, Zhou, ZY 2024, Niu, W -a 2025, Niu, W-b 2025, Yang, H 2025, Zhang, L 2025. All these studies examined the biomarkers of DCM in rodent models and the influence of HMGB1 on DCM in some which displayed substantial heterogeneity of the data that was documented under the characteristics of the included studies. The study design of all these individual primary studies conformed to baseline case-control studies and all the 29 included studies had age and sex-matched healthy controls (free of disease conditions) (**Figure 3**).

#### 2.1.4 Animals

**Figure 4:**
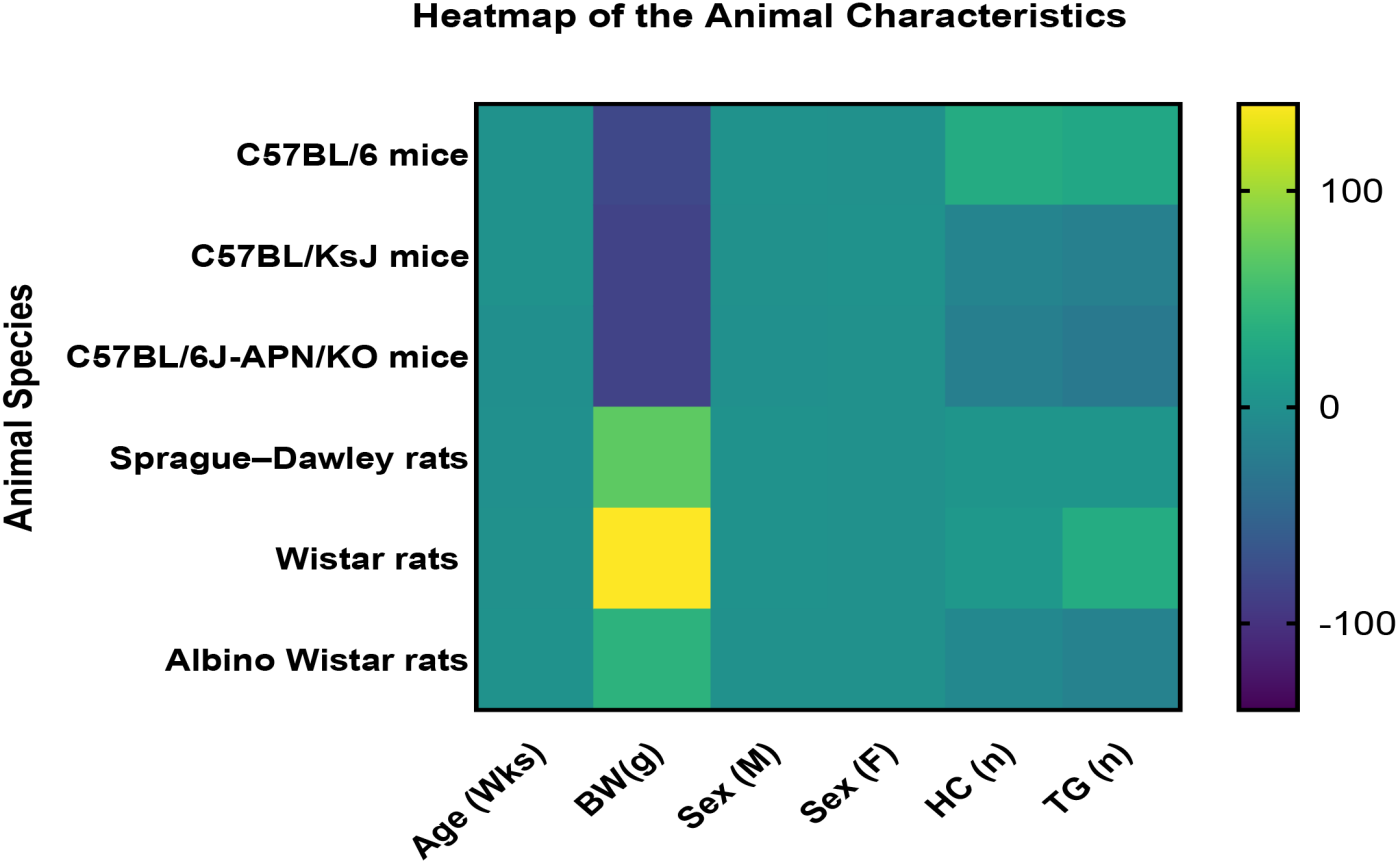
Animal characteristics. This study utilized both mouse and rat species collectively naming them as the rodent model. There were 3 mouse species and 3 rat species that were included. The age, body weight, gender, the number or the sample size in the healthy control group and the treatment group have been tabulated for each study and included in this heatmap. HC, healthy control; TG, treatment group; n, number; Wks, weeks; BW, body weight; KO, gene knockout.

**Figure 5:**
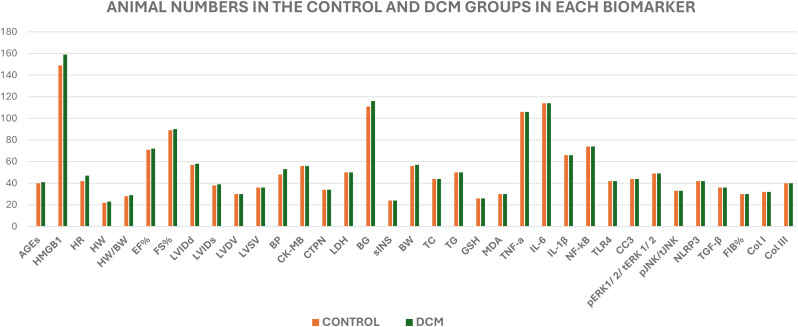
The animal numbers in each primary outcome. This graph at a glance reveals the minimum and maximum numbers of animals used in this study per each biomarker. The number of animals, mouse or rat that exceeded n=100 was displayed by HMGB1, BG, TNF-A and IL-6 biomarkers. The minimum number of animals utilised is 20 while the maximum number was 160. However, the least number of animals were used only for 2 biomarkers, namely, the heart weight (HW) and serum insulin (sINS) concentration.

This study consisted of 588 rodents (265 mice and 323 rats). All the eligible included studies had induced diabetes, and the rodents were mostly adult males aged between 8 to 14 weeks. The highest number of animals were reported from China, which was 391, 6 in Canada, 79 in Italy, 38 from Egypt, 20 from Germany, 38 from India, and 16 from Saudi Arabia. The average body weight of the mice was 18 to 30 g and the body weight of the rat species was 90 to 373 g (**Figures 4**, **5**).

Animal models are best suited to study the complex molecular mechanisms of DCM as heart tissue samples cannot be often taken from clinical studies (55). DCM represents a multifactorial disease complex which enables the researchers to investigate into different therapeutic targets which may be novel strategies, or by splitting disease stages at a convenient pace when studying the disease pathophysiology (56). DCM is the collective outcome of the impairment of many metabolic processes such as inflammation, oxidative stress, cardiac hypertrophy, myocardial fibrosis, insulin resistance, calcium handling, apoptosis and autophagy that can be studied with animal models because those models provide a conducive controlled environment that can be genetically manipulated and for preclinical-drug testing before embarking on clinical trials (57). Among the animal models that are utilized, such as the *db/db* mouse models or the high sucrose or high -fat induced insulin resistance which are diet-induced DCM models, and the gene knock-in or knock-out models are easily able to overcome the limitations of clinical research (58). Another advantage is they allow the study of the full disease progression which is not feasible with human studies (59).

The present study utilized several different mouse and rat models which had one common factor that induced DCM in the animals. That was to have used streptozotocin (STZ) in various doses for varying durations with intraperitoneal delivery. All the included studies had STZ-induced DCM with a blood glucose cutoff point which confirmed hyperglycaemia. Therefore, despite the mouse and rat breeds that were of different genera or species, had the same characteristics of a DCM preclinical model which enabled the induction of DCM combined with a high fat and high sugar diet, to which all the different models had complied. These animal models consist of different adverse effects notwithstanding the similarities between the species such as the mouse and rat models which vary in genetic predisposition. An increase in the advanced glycation end products (AGEs) occurs with the exposure of proteins and fat interlinking with high glucose and is known to destabilize the extracellular matrix proteins which have impaired degradation resulting in cardiac collagen deposits that leads into myocardial stiffness (60). Cardiac fibrosis over time manifests as left ventricular diastolic dysfunction (61). There are several such examples that involve the GLUT4 proteins, the SGLT-2 inhibitors, N-acetyl-cysteines as an antioxidant agent, and RAAS inhibitors have expressed cardioprotective effects in both T1D or T2D -induced DCM in rodent models (62).

There are advantages of utilizing rodent models in preclinical DCM research such as most of the species carry up to thirty thousand genes also encoding DCM proteins which have corresponding homologues in the human genome (63). Additionally, their short breeding cycle and the ability to represent gene-knockout or knock-in functional models have been highly advantageous (64).

#### 2.1.5 Excluded studies

The excluded studies comprised of reviews, posters, editorials and short communications. articles which were not in English, preclinical studies involving animals other than mice and rats, studies which had authors non-responsive to our queries. Also, studies which were on diabetes but without DCM, studies of CVD outside of cardiomyopathies and those did not have the standard deviation or the standard error of the mean calculated (but given as percentages), and the *in vitro* and/or *ex-vivo* models and cross-sectional studies were excluded.

### 2.2 Risk of bias in the included studies

The SYRCLE risk of bias tool which is relevant to preclinical research was used to assess the ROB parameters in this study. The following ten criteria were used to assess each of the individual studies for the risk of bias that is inherent in the primary studies.

- Random sequence generation -selection bias
- Allocation concealment -selection bias
- Baseline characteristics-selection bias
- Random housing-selection bias
- Blinding performance – performance bias
- Blinding detection-performance bias
- Blinding of outcome assessment - detection bias
- Incomplete outcome data -attrition bias
- Selective reporting – reporting bias
- Bias due to other factors

**Figure 6:**
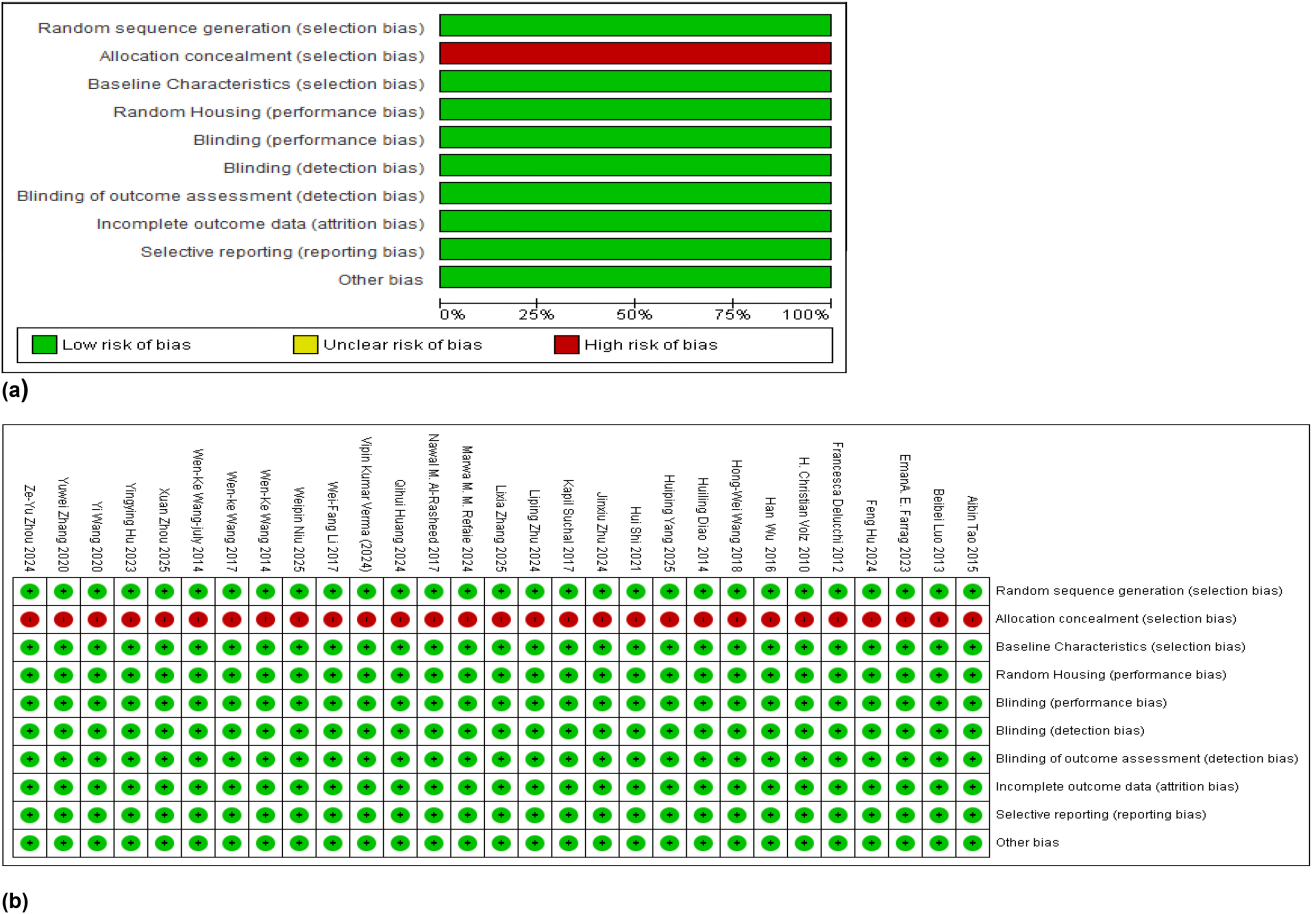
**(a, b)** Risk of bias summary and ROB individual assessments of the included studies.

These ten criteria were evaluated in tabular form with a selected outcome stating either of the 3 responses-high, low or unclear. The risk of bias arises due to confounding factors which are used in determining the prognostic values and they are also used to predict the level of exposure or the exposure group. It included randomization, allocation concealment, baseline characteristics, random housing, blinding of personnel and performance, blinding of outcome assessment, incomplete reporting of data, selective reporting and other biases which were assessed in each individual study. A risk of bias graph and a ROB summary of the 29 included studies are given in **Figures 6A, B**.

#### 2.2.1 Randomization and the allocation bias

Allocation of the rodents in the included studies was carried out randomly without preference. Some studies mentioned randomization of the animals, some studies did not. There was no significant allocation concealment that was mentioned. Yet, they did mention that the studies were conducted according to the pre-approved animal ethics guidelines and in compliance with the rules that required following the standard operational procedures or the SOPs where they were relevant. Therefore, the allocation or selection bias was considered at low risk in all the included studies.

#### 2.2.2 Performance bias and detection bias

Random housing was adhered to by placing the rats and mice in the cages selected randomly. The blinding of the investigators was not mentioned in the individual research studies nor blinding their performance or the outcome assessments, making those parameters to be having a considerable risk of bias.

#### 2.2.3 Attrition bias

This refers to the bias arising from incomplete reporting of the data which was not apparent in the included studies. Both positive and negative results were reported in those publications without selective reporting which was deemed at having low risk of bias. Any discrepancies arising from selective reporting could be detected from the funnel plots which are purported to have studies with large effect sizes gathered towards the top of the funnel plot while the studies having small effect sizes clustered towards the bottom.

#### 2.2.4 Other potential sources of bias

The age and the body weight of the rodents in these case-control studies provided a potential source of bias in almost all the included studies because both rats and mice were included. Sometimes the control group had a higher or lower average age and body weight compared to the diabetic group which led to a high risk of bias as they lead to confounders that could produce false positives in determining the average risk of cardiomyopathies in a given diabetic rodent population. When all aspects considered, the overall risk of bias was low in all the 29 included studies which is further supported by the funnel plots which depict publication bias, drawn per each primary outcome. The ROB analysis was performed in each included study, rating the outcome as one of the three levels specified as low risk, unclear risk, or high risk. These types of observational studies are always exposed to and consist of sources that create high ROB. Confounders may arise from not applying proper eligibility criteria for the control and experimental populations, because they had failed to develop an appropriate study design. There could be under or over-matching the population groups in the case-control studies, or when selecting the exposed and unexposed cohorts from different populations. Further, there could be flawed measurement of exposure or outcome, and having differences in the measurement of an exposure, or differences in the surveillance up to the outcomes in the exposed and unexposed groups. There could be inaccuracies in measuring prognostic factors and lack of appropriate adjustment in statistical analysis and/or incomplete follow-up.

This author carried out the ROB evaluation and arrived at a conclusion under the guidance of an expert in this field. In the meta-analysis, a summary of the overall ROB items is depicted in a risk stratified summary ROB graph. The quality of the body of evidence was maintained with the PRISMA 2020 guidelines, published by the Cochran collaboration, given in the handbook.

The reporting biases can originate in numerous ways such as multiple publications appearing as duplicates, not being included in a given database, missing out a rapid or delayed publication, not being included in a citation list as well as reporting of selective outcomes only. There are also publications with limited access, publications in different languages other than English and unpublished relevant research items. All these reasons constitute of reporting bias which could overtly influence the effect size of a given primary outcome. The reporting bias tends to produce overly optimistic outcomes from the intervention effects. Our search strategy was designed to comprehensively mitigate those flaws associated with reporting bias and for this, funnel plots and the ROC for each biomarker was carried out.

### 2.3 Assessment of heterogeneity

The statistical heterogeneity among the included studies was evaluated by the Q statistic, the I squared metric, and the TAU squared value that were significant at 0.1 probability or 99.9% significance or accuracy. The Q statistic was specific to identifying the accuracy of the included data for a given primary outcome by stating whether it was an outlier or not. The I squared statistic is self-explanatory of heterogeneity in the included data, being low if less than 65% and being high if it was more than 75%. The I squared statistic indicated the observable percentage variability of the data that could be attributed to true heterogeneity. The TAU squared value indicated the average squared deviation around the mean effect size.

#### 2.3.1 Subgroup analysis and investigation of heterogeneity

The subgroup analysis is undertaken if there is a considerable amount of participant data, which is > 6–10 is present in each subgroup. It is commonplace to create subgroups if the units of measurement varied substantially such as the concentration of a biomolecule or a primary outcome was reported in μg/ml, g/l or mg/dl. the descriptive statistics are also separately reported in the forest plots with an overall effect size with the commonly reported Z score, Chi squared distribution, and I squared metric at a probability of p<0.05.

### 2.4 Effects of the intervention

The chemical antidiabetic agents that are in clinical use have not been successful to subdue the extreme pathophysiology which ensues with DCM and therefore it still remains as a need which is not fulfilled. The drugs in present-day use have not been effective in downregulating the adverse side effects and for this reason, a plethora of different therapies have been evaluated using animal models, where rodent models have been the most extensively used to confirm the suitability and validity of the prospective therapies. Farrag et al. in 2023 had experimented with artemisinin, which was previously used as an antimalarial drug that has shown promise in regulating all the increased pathological estimates in the diabetic cohort of Sprague Dawley rats opposed to the healthy control rats. The mechanism of action in this rat DCM model that exacerbated the CVD complications was by upregulating the AGES/RAGE/HMGB1 pathway (Eman A. E. Farrag, 2023). Myocardial ischemia/reperfusion injury is a common morbidity that is associated with DCM and was induced in diabetic albino Wistar rats by ligating the left anterior descending coronary artery, in a study conducted by Suchal et al. in 2017. These researchers also experimented with Kaempferol, a dietary flavonoid which suppressed the AGEs/RAGE and the MAPK axis to produce attenuation of the ischemia/reperfusion injury by downregulating the biomarkers of hyperglycaemia, apoptosis, oxidative stress and inflammation (Kapil Suchal, 2017). Delucchi and colleagues in 2012 showed that the cardiac stem cells or the adult cardiac cell pools are involved in the pathophysiology of DCM in adult Wistar rats induced with type 1 diabetes and there was significant improvement in the cardiomyocyte function when treated with Resveratrol, a natural antioxidant polyphenolic compound. They proved there was improvement in the cardiac haemodynamic performance, the contractile properties and intracellular calcium dynamics whereas the untreated animals displayed ventricular remodelling. This rat model of DCM had demonstrated the induction of the pro-inflammatory cytokine cascade by HMGB1 along with the passive release of it from necrotic cells and an active release of it from the macrophages, and when suppressed by Resveratrol, produced remarkable attenuation of the DCM phenotype (Francesca Delucchi, 2012).

HMGB1 titres in heart tissue was quantified by Wen Ke Wang and the collaborators (2017), with testing an antidiabetic drug namely, Ulinastatin as a prospective therapy for DCM in a mouse model. Treatment with Ulinastatin had conspicuously reduced the level of inflammation and the level of cytokines; TNF-a, IL-6 and IL-1β along with serum HMGB1. The results of this study also provided evidence that the ischemic/reperfusion injury in the DCM mice was attenuated by Ulinastatin *via* the inhibition of the phosphorylated p38, JNK and MAPK signalling pathway (Weng-Ke Wang, 2017). Thus, it is confirmed that hyperglycaemia together with increased protein glycation activity, induces the formation of the AGEs through the biochemical reaction named the Maillard reaction. The AGEs upregulate DCM by binding with the membrane-bound TLR4 and myeloid differentiation 2 (MD2) receptors for internalization, to stimulate the MAPK and NF-kB signalling pathways which increase inflammation causing intense cardiac fibrosis and cardiac dysfunction (Yi Wang, 2020). This study hypothesised that the inhibition of the AGEs synthesis along with the stimulated RAGE and HMGB1 could strongly inhibit several signalling cascades which could finally attenuate DCM. It comprises of many complicated heart and vasculature-associated diseases that would eventually cause premature mortality in the diabetic population if not diagnosed and treated in its early stages. A recent research work on oxidative stress biomarkers have introduced the Transient receptor potential melastatin 2 (TRPM2) activity which promoted systolic and diastolic dysfunction, myocardial apoptosis and the inhibition of autophagy in a C57BL/6N mouse model. It was achieved by injecting adeno-associated virus type 9 carrying *TRPM2* shRNA, into the STZ/HFD - induced diabetic heart *via* the tail vein *in vivo* and to high glucose-stimulated cardiomyocytes *in vitro*. TRPM2 - knockdown displayed attenuation of myocardial impairment of structure and function, thus indicating its potential to be a diagnostic or treatment target in preclinical diabetic cardiomyopathy (Hu and Lin 2024). A study that investigated the influence of ferroptosis in a DCM - induced mouse model by targeting the activity of haem-oxygenase -1 (HMOX-1) has revealed the enrichment of differentially expressed genes during primary fatty acid metabolism and mitochondrial fatty acid beta oxidation that closely mimics the ferroptosis process. The occurrence of ferroptosis was further supported by the increased expression of LDH, MDA and the decreased expression of GSH, both *in vitro* with H9C2 cells stimulated with high glucose and palmitic acid and *in vivo*. The knockdown of *HMOX-1* has revealed the amelioration of ferroptosis by improving cardiac function and reducing cardiac fibrosis in those murine hearts (Yang et al, 2024). Zhu et al, 2024 reported a link between the short-chain fatty acid -producing gut microbiota and DCM utilising STZ/HFD mice that were treated with Myricetin, a polyphenolic compound which has antioxidant, anti-inflammatory and anti-atherosclerotic properties. Myricetin is a cardioprotective compound but has very low bioavailability although it can regulate the gut microbiome to improve the enhanced DCM pathologies (Zhu et al, 2024). Researchers named Zhu and He have reported the therapeutic potential of Morin, a flavanol on the improvement of DCM in an albino Wistar rat model. Long-term treatment with Morin had ameliorated DCM pathogenesis related to apoptosis, autophagy, inflammation and oxidative stress which also improved cardiac hypertrophy and fibrosis in those rats (Zhu and He, 2024). Zhang et al. (2025) have documented that the uncoupling protein 2 inhibition serves to worsen the DCM pathologies by the impairment of the mitochondrial membrane potential, increased ROS, activation of the NLRP3 and pyroptosis-related proteins (Zhang et al., 2025). Male rats induced with T2DM were subjected to epicardial fibrillation in order to produce cardiac arrest followed by cardiopulmonary resuscitation to investigate the effect of canagliflozin, a sodium glucose co-transporter inhibitor class 2 drug by a study group. Pre-treatment of rats for 4 weeks with canagliflozin had improved the cardiac injuries caused by high glucose and hypoxia-reoxygenation *via* the PI3K/AKT/mTOR pathway (Huang et al, 2024). Another study which gave long-term pre-treatment with Morin to a group of male Wistar rats, induced a myocardial infarction at the end of the oral gavage period. Morin treatment had significantly decreased inflammatory markers (TNF-A, IL 6) oxidative stress markers (GSH, MDA and SOD), cardiac biomarkers (CK-MB, LDH), and both intrinsic and extrinsic apoptosis by upregulating the Akt/eNOS/Nrf2/HO-1 and MAPK pathways (Verma et al, 2024). Zhou and co-workers, 2024, suggested the supplementation of branched chain amino acids into the diet which exhibited attenuation of DCM progression by inhibiting autophagy in the cardiac fibroblasts (Zhou et al, 2024). The amniotic mesenchymal stem cells (AMSCs) injected *via* the tail vein, have been introduced to provide multiple benefits in attenuating DCM in a mouse model, particularly pyroptosis, by suppressing the TLR4/NF-kB/NLRP3 signalling pathway. These AMSCs being of low immunogenicity and pluripotent, serve as a potential therapeutic target in murine DCM pathologies. The AMSCs were shown to inhibit pro-inflammatory cytokines by suppressing the MyD88 and NF-kB signalling cascade and the TGF-β Smad pathway which upregulated myocardial fibrosis. Further, treatment of DCM mice with AMSCs have displayed an improvement of pancreatic functions, reduction in the blood glucose level and increasing insulin secretion (Zhou et al, 2025). Piezo 1 is a mechanosensitive ion channel which participates in cardiovascular homeostasis and a multitude of physiological activities, including blood pressure regulation, cell fate determination and stem cell aging. Piezo 1 was upregulated in the diabetic myocardium which in turn induced excessive mitochondrial fission, cardiac fibrosis and cardiac injury in both *in vitro* testing with H9C2 cells, and mouse neonatal cardiomyocytes exposed to high glucose and in an *in vivo* cardiac - specific Piezo 1-knockout mouse model. Piezo 1- deficiency enabled the restoration of mitochondrial dysfunction while ameliorating cardiac pathologies *via* the phosphorylation of Drp1 and ERK 1/2 pathways (Niu et al, 2025).

### 2.5 Overview of the outcome measures

#### 2.5.1 Primary outcomes

A total of 37 primary outcomes were reported by the 29 included studies (**Figure 5**). Thirty primary outcomes showed a significantly high risk of developing endothelial deregulation (ED) in diabetic animals from forest plots that calculated the SMD with a 95% confidence intervals (CI). The overall percentage of the significantly high risk of DCM shown by the forest plots of the primary outcomes in this meta-analysis is 80.5% (at p < 0.05). The minimum number of diabetic animals included was 3 per study and the maximum number of diabetic animals were around 75 (**Figure 5**). Therefore, all these primary studies were utilized in constructing forest plots for the calculation of the standardized mean difference (SMD).

#### 2.5.2 Secondary outcomes

**Figure 7:**
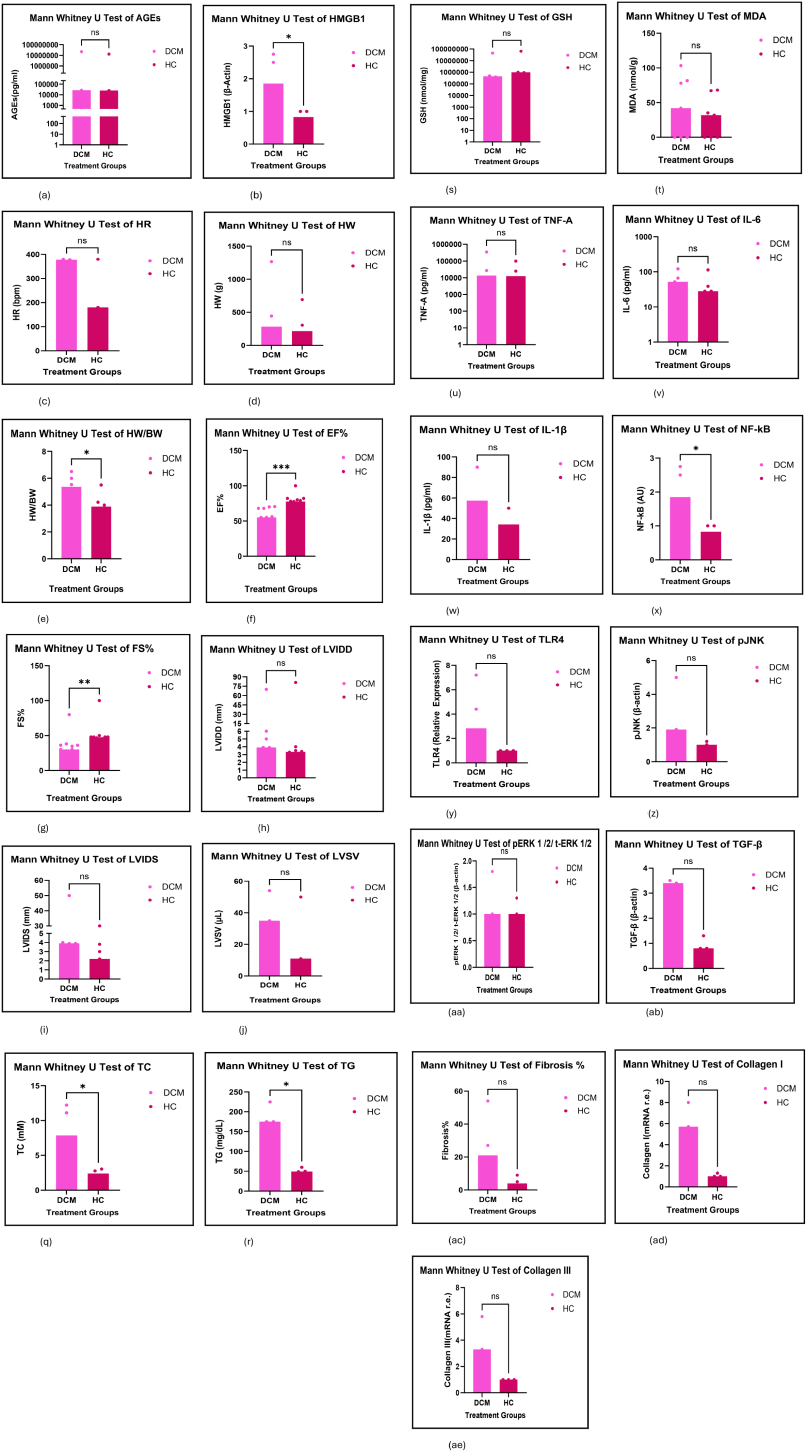
**(A-AE)** Displays the non-parametric Mann Whitney U test results of the biomarkers of this study in bar graphs as the secondary outcomes. Only 31 biomarkers had sufficient data that could be evaluated, and 8 biomarkers showed a significant difference between the means of the treatment group and the healthy control group. Significant increases in the treatment group were shown by BG, HW/BW, TC, TG, NF-kB and HMGB1, while significant decreases were shown by the EF%, and FS%. These results indicate the manifestation of DCM in the proposed rodent model and are deemed to be high-risk factors for the manifestation of DCM in our rodent model.

The secondary outcomes include the non-parametric Mann Whitney U test bar graphs, plotted between the healthy control and the DCM cohort, and the calculation of both Pearson’s and Spearman’s correlation coefficients. (**Figure 7**, **Table 1**).

**Table 1:** Statistical data obtained from the non-parametric Mann Whitney U test on the biomarkers as secondary outcomes.

Figure	Biomarker	Mann Whitney U	P<0.05	Number of animals (n)	Median of column A	Median of column B	Hodges Lehmann difference (HL) value
9A	AGEs	3	0.7000	3	26540	24890	-1650
9B	HMGB1	0	0.0286*	4	1850	0.8250	-1.150
9C	HR	2.5	0.5000	3	378	180	-122
9D	HW	7	0.8857	4	282.51	215.6	-16.40
9E	HW/BW	3	0.0152*	6	5.365	3.890	-1766
9F	EF%	20	0.0004*	13	55.0	77.60	21.60
9G	FS%	28	0.0093*	12	30.0	47.38	13.33
9H	LVIDD	23	0.3128	8	3.905	3.350	-55.50
9I	LVIDS	16.50	0.3281	7	3.9	2.2	-0.9
9J	LVSV	4	>0.9999	3	35	11	-0.4
9K	CK-MB	13	0.4848	6	581.3	436.3	-145
9L	CTPN	6	0.6857	4	200095	45085	-152.5
9M	LDH	19	0.5350	7	350	300	-104.3
9N	BP	3	0.7000	3	115	108	-7000
9O	BG	9	<0.0001*	12	23.18	7.35	-16.12
9P	BW	18	0.4557	7	26.80	30.40	3.170
9Q	TC	0	0.0286*	4	7.86	2.38	-5.435
9R	TG	3	0.0476*	5	175	49	-125
9S	GSH	5	0.4857	4	45000	985000	535000
9T	MDA	15	0.2593	7	42.0	31.77	-10
9U	TNF-A	6	0.6856	4	13546	12540	-45.96
9V	IL-6	10	0.2229	6	59.12	33.26	-20
9W	IL-1β	1	06667	2	57.42	34.12	-23.31
9X	NF-KB	0	0.0286*	4	7.1	1.0	-6.1
9Y	TLR4	3	0.2000	4	2.825	1.0	-2.230
9Z	PERK1/2/T-ERK 1/2	3.5	0.8000	3	1.0	1.0	-0.35
9AA	PJNK/T-JNK	1.5	0.3000	3	1.9	1.0	-0.9
9AB	TGF- β	1	0.2000	3	3.4	0.8	-2.2
9AC	FB%	1	0.0571	3	21.0	4.0	-15.25
9aAD	Col I	0	0.1	3	5.7	1.0	-4.7
9aAE	Col III	0	0.1	3	3.3	1.0	-2.3

### 2.6 Adverse effects

The diabetic individuals who develop cardiomyopathies are also prone to developing other systemic diseases due to the complexity of the risk factors such as hypertension, hyperglycaemia and hyperlipidaemia. The repertoire of DCM itself manifests into a spectrum of cardiometabolic diseases. Therefore, clinical diabetes could lead to severe morbidity and premature mortality. The existing treatment is not effective to fully contain the disease and suppress the mechanisms of oxidative stress, ER stress, the unfolded protein response. Further, the available treatment has failed to contain the various forms of cellular death such as apoptosis, pyroptosis, necroptosis, myocardial fibrosis and impaired autophagy which add to increase the complexity of the cardiomyopathy disease burden. Additionally, the cost of treatment and hospitalization is very high in some countries adding on to its spending on healthcare. Similarly, when young adults are affected, their earnings are reduced, and the economic burden becomes high along with a decrease in productivity. The severe morbidity that ensues with the manifestation of DCM could trigger psychological problems such as depression.

### 2.7 Sensitivity analysis

Sensitivity analysis is carried out to prove that the statistical plots, particularly the forest plots are flexible and can be updated. It demonstrates that the forest plot is sensitive to any change in its values and it is not merely a static plot but a dynamic entity.

### 2.8 Biomarker results in brief

#### 2.8.1 Advanced glycation end products

The AGEs were evaluated in a forest plot having two subgroups of which; one showed a high risk for developing DCM while the other subgroup showed no risk although the overall effect indicated a significantly high-risk of developing DCM. The arbitrary units of the AGEs when plotted in a non-parametric Mann Whitney U test bar graph showed no change in the DCM group compared to the HC. The ROC showed the data utilised did not consist of outliers (**Supplementary Figure S1-1**; **Supplementary Table S3-1**; **Figure 7A**; **Table 1**; **Supplementary Figure S4-1**).

#### 2.8.2 High mobility group box-1

HMGB1 was evaluated in a forest plot having six subgroups; of which two showed a high risk for developing DCM while other subgroups showed no risk although the overall effect indicated a significantly high-risk for developing DCM. The relative β-Actin units of HMGB1 in a western blot when plotted in a non-parametric Mann Whitney U test bar graph showed a significant increase in the DCM group compared to the HC. The ROC showed the data utilised did not consist of outliers. (**Supplementary Figure S1-2**; **Supplementary Table S3-1**; **Figure 7b**; **Table 1**; **Supplementary Figure S2-1**; **Supplementary Figure S4-2**).

#### 2.8.3 Heart rate

The HR was evaluated in a forest plot which showed no risk for developing DCM; The HR when plotted in a Mann Whitney U test bar graph in beats per minute (bpm) showed no change in the DCM group compared to the HC; The ROC showed most of the data utilised were not outliers; (**Supplementary Figure S1-3**; **Supplementary Table S3-2**; **Figure 7C**; **Table 1**; **Supplementary Figure S4-3**);

#### 2.8.4 Heart weight

The HW was evaluated in a forest plot which showed no risk for developing DCM. The HW when plotted in a Mann Whitney U test bar graph in grams (g) showed no change in the DCM group compared to the HC. The ROC showed most of the data utilised were not outliers. (**Supplementary Figure S1-4**; **Supplementary Table S3-2**; **Figure 7D**; **Table 1**; **Supplementary Figure S4-4**).

#### 2.8.5 Heart weight to body weight ratio

The HW/BW ratio was evaluated in a forest plot which showed a significantly high risk for developing DCM. The HW/BW ratio when plotted in a Mann Whitney U test bar graph showed a significant increase in the DCM group compared to the HC. The ROC showed the data utilised were not outliers. (**Supplementary Figures S1-5**; **Supplementary Tables S3-2**; **Figure 7E**; **Table 1**; **Supplementary Figures S2-8**; **Supplementary Figures S4-5**).

#### 2.8.6 Ejection fraction

The EF% was evaluated in a forest plot which showed a significantly high risk for developing DCM. The EF% when plotted in a Mann Whitney U test bar graph showed a significant decrease in the DCM group compared to the HC. The ROC showed the data utilised did not contain outliers (**Supplementary Figures S1-6**; **Supplementary Tables S3-2**; **Figure 7F**; **Table 1**; **Supplementary Figures S2-2**; **Supplementary Figures S4-6**).

#### 2.8.7 Fractional shortening

The FS% was evaluated in a forest plot which showed a significantly high risk for developing DCM. The FS% when plotted in a Mann Whitney U test bar graph showed a significant decrease in the DCM group compared to the HC. The ROC showed the data utilised did not contain outliers. (**Supplementary Figures S1-7**; **Supplementary Tables S3-2**; **Figure 7G**; **Table 1**; **Supplementary Figures S2-3**; **Supplementary Figures S4-7**).

#### 2.8.8 Left ventricular internal dimension at end-diastole

The LVIDd was evaluated in a forest plot which showed no risk for developing DCM. The LVIDd in mm when plotted in a Mann Whitney U -test bar graph showed no significant change in the DCM group compared to the HC. The ROC showed most of the data utilised did not contain outliers. (**Supplementary Figures S1-8**; **Supplementary Tables S3-2**; **Figure 7H**; **Table 1**; **Supplementary Figures S2-4**; **Supplementary Figures S4-8**).

#### 2.8.9 Left ventricular internal dimension at end-systole

The LVIDs was evaluated in a forest plot which showed a significantly high risk for developing DCM. The LVIDs in mm when plotted in a Mann Whitney U test bar graph showed no significant change in the DCM group compared to the HC. The ROC showed most of the data utilised did not contain outliers. (**Supplementary Figures S1-9**; **Supplementary Tables S3-2**; **Figure 7I**; **Table 1**; **Supplementary Figures S2-5**; **Supplementary Figures S4-9**).

##### 2.8.10 Left ventricular diastolic volume

The LVDV was evaluated in a forest plot from 1 study which showed a significantly high risk for developing DCM; but the data was insufficient to arrive at a conclusion. The data was insufficient to plot a Mann Whitney U test bar graph and the ROC (**Supplementary Figures S1-10**; **Supplementary Table S3-2**).

#### 2.8.11 Left ventricular systolic volume

The LVSV was evaluated in a forest plot which showed no significantly high risk for developing DCM. The LVSV in microlitres when plotted in a Mann Whitney U test bar graph showed no significant difference in the DCM group compared to the HC. The ROC showed the data utilised did not contain outliers. (**Supplementary Figures S1-11**; **Supplementary Table S3-2**; **Figure 7J**; **Table 1**; **Supplementary Figures S4-10**).

#### 2.8.12 Creatine Kinase-muscle and brain

The CK-MB was evaluated in a forest plot which consisted of two subgroups in which one showed a significantly high risk but not the other with an overall high significant risk for developing DCM; The CK-MB in U/L when plotted in a Mann Whitney U test bar graph showed no difference in the DCM group compared to the HC; The ROC showed the data utilised did not contain outliers; (**Supplementary Figures S1-12**; **Supplementary Table S3-2**; **Figure 7k**; **Table 1**; **Supplementary Figures S2-6**; **Supplementary Figures S4-11**);

#### 2.8.13 Lactate dehydrogenase

Lactate dehydrogenase (LDH) was evaluated in a forest plot which showed a significantly high risk for developing DCM. The expression of LDH in U/L when plotted in Mann Whitney U test bar graph showed no change in the DCM group compared to the HC. The ROC showed the data utilised did not consist of outliers. (**Supplementary Figures S1-13**; **Supplementary Table S3-2**; **Figure 7L**; **Table 1**; **Supplementary Figures S2-7**; **Supplementary Figures S4-13**).

#### 2.8.14 Cardiac troponin

Cardiac troponin was evaluated in a forest plot which showed a significantly high risk of developing DCM. The expression of CTN in pg/ml when plotted in a Mann Whitney U test bar graph showed no difference in the DCM group compared to HC. The ROC showed the data utilised did not consist of outliers. (**Supplementary Figures S1-14**; **Supplementary Table S3-2**; **Figure 7M**; **Table 1**; **Supplementary Figures S4-12**).

#### 2.8.15 Blood pressure

Blood pressure (BP) was measured in the rodents but without specification. The results of the forest plot showed no significant difference between the two groups which was the same in the Mann Whitney U test bar graph. In the ROC; most of the data did not consist of outliers. (**Supplementary Figures S1-15**; **Supplementary Table S3-2**; **Figure 7N**; **Table 1**; **Supplementary Figures S4-14**).

#### 2.8.16 Blood glucose

Blood glucose (BG) was evaluated in a forest plot which showed a significantly high risk for developing DCM. The expression of BG in mM when plotted in a Mann Whitney U test bar graph showed a significant increase in the DCM group compared to the HC. The ROC showed the data utilised did not consist of outliers. (**Supplementary Figures S1-16**; **Supplementary Table S3-3**; **Figure 7O**; **Table 1**; **Supplementary Figures S2-9**; **Supplementary Figures S4-15**).

#### 2.8.17 Serum insulin

Serum insulin (sINS) was evaluated in a forest plot which had only 2 studies and showed no significant high risk for developing DCM; although insufficient data may become a limitation. The data was insufficient to plot a Mann Whitney U test bar graph and the ROC (**Supplementary Figures S1-17**; **Supplementary Table S3-2**; **Supplementary Figures S4-16**).

#### 2.8.18 Body weight

Body weight (BW) was evaluated in a forest plot which showed a significantly high risk for developing DCM with an overall decrease in BW in the treatment group. The expression of BW in grams (g) when plotted in a Mann Whitney U test bar graph showed no difference in the DCM group compared to the HC. The ROC showed that most of the data utilised were not outliers. (**Supplementary Figures S1-18**; **Supplementary Table S3-3**; **Figure 7P**; **Table 1**; **Supplementary Figures S2-10**; **Supplementary Figures S4-17**).

#### 2.8.19 Total cholesterol

Total cholesterol (TC) was evaluated in a forest plot which showed a significantly high risk for developing DCM. The expression of TC in mM when plotted in a Mann Whitney U test bar graph showed a significant increase in the DCM group compared to the HC. The ROC showed the data utilised did not consist of outliers. (**Supplementary Figures S1-19**; **Supplementary Table S3-4**; **Figure 7Q**; **Table 1**; **Supplementary Figures S4-18**).

#### 2.8.20 Total triglycerides

Total triglycerides (TG) were evaluated in a forest plot which showed a significantly high risk for developing DCM. The expression of TG in mg/dl when plotted in a Mann Whitney U test bar graph showed a significant increase in the DCM group compared to the HC. The ROC showed the data utilised did not consist of outliers. (**Supplementary Figures S1-20**; **Supplementary Table S3-4**; **Figure 7R**; **Table 1**; **Supplementary Figures S2-11**; **Supplementary Figures S4-19**).

#### 2.8.21 High-density lipoprotein

The high-density lipoproteins were evaluated in only a forest plot consisting of one study which had data from 6 animals. Not having sufficient data is a limitation. The data was insufficient to plot a Mann Whitney U test bar graph and the ROC (**Supplementary Figures S1-21**; **Supplementary Table S3-4**).

#### 2.8.22 Low-density lipoprotein

The low-density lipoproteins were evaluated in only a forest plot consisting of one study which had data from 6 animals. Not having sufficient data is a limitation. The data was insufficient to plot a Mann Whitney U test bar graph and the ROC (**Supplementary Figures S1-22**; **Supplementary Table S3-4**).

#### 2.8.23 Glutathione

Glutathione (GSH) was evaluated in a forest plot which showed a significantly high risk for developing DCM with an overall decrease in titres in the DCM group. The expression of GSH in nmol/mg when plotted in a Mann Whitney U test bar graph showed no difference in the DCM group compared to the HC. The ROC showed the data utilised did not consist of outliers. (**Supplementary Figures S1-23**; **Supplementary Table S3-5**; **Figure 7S**; **Table 1**; **Supplementary Figures S4-20**).

#### 2.8.24 Malondialdehyde

Malondialdehyde (MDA) was evaluated in a forest plot which showed a significantly high risk for developing DCM. The expression of MDA in nmol/mg when plotted in a Mann Whitney U test bar graph showed no change in the DCM group compared to the HC. The ROC showed the data utilised did not consist of outliers. (**Supplementary Figures S1-24**; **Supplementary Table S3-5**; **Figure 7T**; **Table 1**; **Supplementary Figures S2-12**; **Supplementary Figures S4-21**).

#### 2.8.25 Tumour necrosis factor - alpha

Tumour necrosis factor - Alpha (TNF-A) was evaluated in a forest plot having four subgroups of which one showed a significantly high risk for developing DCM with an overall high risk for DCM. The expression of TNF-A in pg/ml when plotted in a Mann Whitney U test bar graph showed no change in the DCM group compared to the HC. The ROC showed the data utilised did not consist of outliers. (**Supplementary Figures S1-25**; **Supplementary Table S3-6**; **Figure 7U**; **Table 1**; **Supplementary Figures S2-13**; **Supplementary Figures S4-22**).

#### 2.8.26 Interleukin-6

Interleukin-6 (IL-6) was evaluated in a forest plot having four subgroups of which all showed a significantly high risk for developing DCM with an overall high risk for DCM. The expression of IL-6 in pg/ml when plotted in a Mann Whitney U test bar graph showed no difference in the DCM group compared to the HC. The ROC showed the data utilised did not consist of outliers. (**Supplementary Figures S1-26**; **Supplementary Table S3-6**; **Figure 7V**; **Table 1**; **Supplementary Figures S2-14**; **Supplementary Figures S4-23**).

#### 2.8.27 Interleukin-1β

Interleukin-1β (IL-1β) was evaluated in a forest plot having four subgroups of which three showed a significantly high risk for developing DCM with an overall high risk for DCM. The data was insufficient to plot a Mann Whitney U test bar graph and the ROC (**Supplementary Figures S1-27**; **Supplementary Table S3-6**; **Supplementary Figures S2-15**).

#### 2.8.28 Nuclear factor kappa beta

NF-kB was evaluated in a forest plot having four subgroups of which all showed a significantly high risk of developing DCM with an overall high-risk for DCM. The fold-effect of arbitrary units of NF-kB when plotted in a Mann Whitney U test bar graph; it showed a significant increase in the DCM group compared to HC. The ROC showed the data utilised did not consist of outliers. (**Supplementary Figures S1-28**; **Supplementary Table S3-7**; **Figure 7W**; **Table 1**; **Supplementary Figures S2-20**; **Supplementary Figures S4-24**).

#### 2.8.29 Toll-like receptor 4

TLR4 was evaluated in a forest plot having two subgroups; both of which showed high risk of developing DCM. The relative expression of TLR4 when plotted in a Mann Whitney U test bar graph; showed no change in the DCM group compared to HC. The ROC showed the data utilised did not consist of outliers. (**Supplementary Figures S1-29**; **Supplementary Table S3-7**; **Figure 7X**; **Table 1**; **Supplementary Figures S2-16**; **Supplementary Figures S4-25**).

#### 2.8.30 Cleaved caspase 3

CC3 was evaluated in a forest plot having four subgroups of which all showed a high risk of developing DCM; resulting in an overall significantly high risk of DCM. The data was insufficient to plot a Mann Whitney U test bar graph and the ROC. (**Supplementary Figures S1-30**; **Supplementary Table S3-7**).

#### 2.8.31 NLR family pyrin domain containing 3

NLRP3 was evaluated in a forest plot having four subgroups of which two showed high risk of developing DCM; resulting in an overall high risk of DCM. The data was insufficient to plot a Mann Whitney U test bar graph and the ROC (**Supplementary Figures S1-31**; **Supplementary Table S3-7**).

#### 2.8.32 Phosphorylated extracellular regulated kinase 1/2 to total extracellular regulated kinase 1/2 ratio

The pERK1/2/t-ERK1/2 ratio was evaluated in a forest plot having two subgroups in which both showed no risk for developing DCM; resulting in an overall no risk of DCM. The relative protein expression of pERK1/2/t-ERK1/2 ratio when plotted in a Mann Whitney U test bar graph also showed no change in the DCM group compared to the HC. The ROC showed the data utilised did not consist of outliers. (**Supplementary Figures S1-32**; **Supplementary Table S3-7**; **Figure 7Y**; **Table 1**; **Supplementary Figures S2-17**; **Supplementary Figures S4-26**).

#### 2.8.33 Phosphorylated c-Jun -N terminal kinase 1/2 to total c-Jun -N terminal kinase ratio

The pJNK/t-JNK ratio was evaluated in a forest plot having three subgroups of which two showed a high risk for developing DCM; resulting in an overall high risk for DCM. The relative protein expression of pJNK/t-JNK ratio when plotted in a Mann Whitney U test bar graph showed no significant change in the DCM group compared to the HC. The ROC showed the data utilised did not consist of outliers. (**Supplementary Figures S1-33**; **Supplementary Table S3-7**; **Figure 7Z**; **Table 1**; **Supplementary Figures S2-18**; **Supplementary Figures S4-27**).

#### 2.8.34 Transforming growth factor -one beta

The TGF-β was evaluated in a forest plot having three subgroups of which two showed a high risk for developing DCM; resulting in an overall high risk for DCM. The relative protein expression of TGF-β when plotted in a Mann Whitney U test bar graph; showed no significant difference in the DCM group compared to the HC. The ROC showed the data utilised did not consist of outliers. (**Supplementary Figures S1-34**; **Supplementary Table S3-7**; **Figure 7AA**; **Table 1**; **Supplementary Figures S2-19**; **Supplementary Figures S4-28**).

#### 2.8.35 Fibrosis %

The FB% was evaluated in a forest plot which showed an overall high risk for developing DCM. The percentage area of fibrosis when plotted in a Mann Whitney U test bar graph showed no difference in the DCM group compared to the HC. The ROC showed the data utilised did not consist of outliers. (**Supplementary Figures S1-35**; **Supplementary Table S3-8**; **Figure 7AB**; **Table 1**; **Supplementary Figures S4-29**).

#### 2.8.36 Collagen I

The Collagen I was evaluated in a forest plot having two subgroups of which one showed a high risk for developing DCM, resulting in an overall high risk for DCM. The relative expression of mRNA when plotted in a t-test bar graph showed a non-significant increase in the DCM group compared to the HC. The ROC showed the data utilised did not consist of outliers. (**Supplementary Figures S1-36**; **Supplementary Table S3-8**; **Supplementary Figures S2-21**, **Supplementary Figures S4-30**).

#### 2.8.37 Collagen III

The Collagen III was evaluated in a forest plot having three subgroups of which two showed a high risk for developing DCM, resulting in an overall high risk for DCM. The relative mRNA expression of Collagen III when plotted in a t-test bar graph showed a non-significant increase in the DCM group compared to the HC. The ROC showed the data utilised did not consist of outliers. (**Supplementary Figures S1-37**; **Supplementary Table S3-8**; **Supplementary Figures S2-22**; **Supplementary Figures S4-31**).

## 3 Discussion

### 3.1 Summary of the main results

A total of 37 biomarkers of DCM obtained from 6 different rodent species have been grouped into 8 biomarker models. The effect size of each biomarker was calculated and has been compared based on the Cohen’s d classification of the effect size (**Table 2**). Additionally, the Pearson’s and Spearman’s correlation coefficient and the coefficient of determination at a probability of *p=0.05* or below have been calculated between HMGB1 as the independent variable and the biomarkers of DCM as the dependent variables are given in **Table 3**.

**Table 2:** The effect sizes of each risk factor calculated from the forest plots.

Description of the model	Biomarkers/risk factors of DCM	Animals (N)	Small effect size (0.2-0.5)	Medium effect size (0.5-0.8)	Large effect size (>0.8)	P<0.05
ADVANCED GLYCATION END PRODUCTS	AGEs	81			5.43	0.004
	HMGB1	256			3.0	0.0001
CARDIOMETABOLIC BIOMARKERS	HR	40			12.20	0.08
	HW	65			4.73	0.05
	HW/BW	89			2.31	0.007
	EF%	207			-4.13	0.00001
	FS%	187			-2.91	0.00001
	LVIDd	119	-0.09			0.91
	LVIDs	93			1.64	0.04
	LVDV	28			4.93	0.00001
	LVSV	52			-1.38	0.33
	CK-MB	112			3.28	0.004
	CTPN	60			37.93	0.03
	LDH	84			5.66	0.0005
	BP	56			1.71	0.17
GLYCAEMIC BIOMARKERS	BG	187			7.54	0.00001
	SINS	32			-9.46	0.31
	BW	105			-1.74	0.05
LIPID BIOMARKERS	TC	64			7.38	0.009
	TG	76			16.60	0.0007
	HDL	12*			-5.84	0.0002
	LDL	12*			39.70	0.0001
OXIDATIVE STRESS BIOMARKERS	GSH	58			-4.60	0.009
	MDA	90			4.78	0.00001
INFLAMMATORY BIOMARKERS	TNF-A	132			1.11	0.03
	IL-6	186			5.49	0.00001
	IL-1β	92			4.44	0.003
SIGNALLING PATHWAY BIOMARKERS	NF-KB	112			3.13	0.0001
	TLR4	84			2.38	0.009
	CC3	76			3.91	0.002
	NLRP3	68			3.59	0.03
	pERK 1/2/ t-ERK 1/2	88	0.46			0.42
	pJNK/t-JNK	66			1.87	0.0001
	TGF- β	84			1.50	0.05
FIBROSIS BIOMARKERS	FIBROSIS %	64			3.25	0.01
	Collagen I	84			2.31	0.007
	Collagen III	84			2.43	0.010

* Not included in the analysis because there is only data from one study consisting of 6 animals per group.

**Table 3:** Summary of the correlation data between HMGB1 and the biomarker models.

Model	Variable	Pearson’s correlation coefficient (r_p_)	Coefficient of determination (R^2^)	p<0.05	Spearman’s correlation coefficient (r_s_)	p<0.05
CARDIOMETABOLIC BIOMARKERS	HR	-0.9998	0.9997	0.0113*	-0.5000	>0.9999
	HW	0.8545	0.7301	0.1455	1.000	0.0833
	HW/BW	-0.9551	0.9122	0.0449*	1.000	0.0833
	EF%	0.9580	0.9178	0.0420*	1.000	0.0833
	FS%	-0.8289	0.6870	0.1711	-0.8000	0.3333
	LVIDd	-0.5847	0.3418	0.4153	-0.4000	0.7500
	LVIDs	0.5464	0.2986	0.4536	0.4000	0.7500
	LVSV	0.6635	0.4402	0.3365	0.8000	0.3333
	CK-MB	-0.4633	0.2147	0.5367	0.4000	0.7500
	CTPN	-0.1332	0.01775	0.8668	-0.8000	0.3333
	LDH	-0.6803	0.4628	0.3197	-0.6000	0.4167
	BP	-0.3226	0.1041	0.7909	0.5000	>0.9999
GLYCAEMIC BIOMARKERS	BG	0.5962	0.3555	0.4038	0.8000	0.3333
	sINS	-0.3042	0.09254	0.6958	0.4000	0.7500
	BW	0.4763	0.2268	0.5237	0.4000	0.7500
LIPID BIOMARKERS	TC	-0.5215	0.2720	0.4785	0.4000	0.7500
	TG	-0.5156	0.2658	0.4844	0.4000	0.7500
OXIDATIVE STRESS BIOMARKERS	GSH	0.4644	0.2157	0.5356	-0.2000	0.9167
	MDA	0.6932	0.4805	0.3068	0.8000	0.3333
INFLAMMATORY BIOMARKERS	TNF-A	0.6723	0.4520	0.3277	0.2000	0.9167
	IL-6	-0.3846	0.1479	0.6154	-0.4000	0.7500
	IL-1β	-0.1238	0.01532	0.9210	0.5000	>0.9999
SIGNALLING PATHWAY BIOMARKERS	NF-kB	0.4092	0.1675	0.5908	0.8000	0.3333
	TLR4	0.9617	0.9248	0.0383*	0.8000	0.3333
	pERK 1/2/t-ERK 1/2	-0.3336	0.1113	0.7835	-0.5000	>0.9999
	pJNK	-0.3090	0.09546	0.8000	-0.5000	>0.9999
	TGF-β	0.5372	0.2885	0.6390	1.000	0.3333
FIBROSIS BIOMARKER	FB%	-0.2487	0.06187	0.7513	0.4000	0.7500
	Col I	0.8384	0.7030	0.3669	0.5000	>0.9999
	Col III	0.9565	0.9149	0.1885	1.000	0.3333

### 3.2 Overall completeness and applicability of evidence

**Figure 8:**
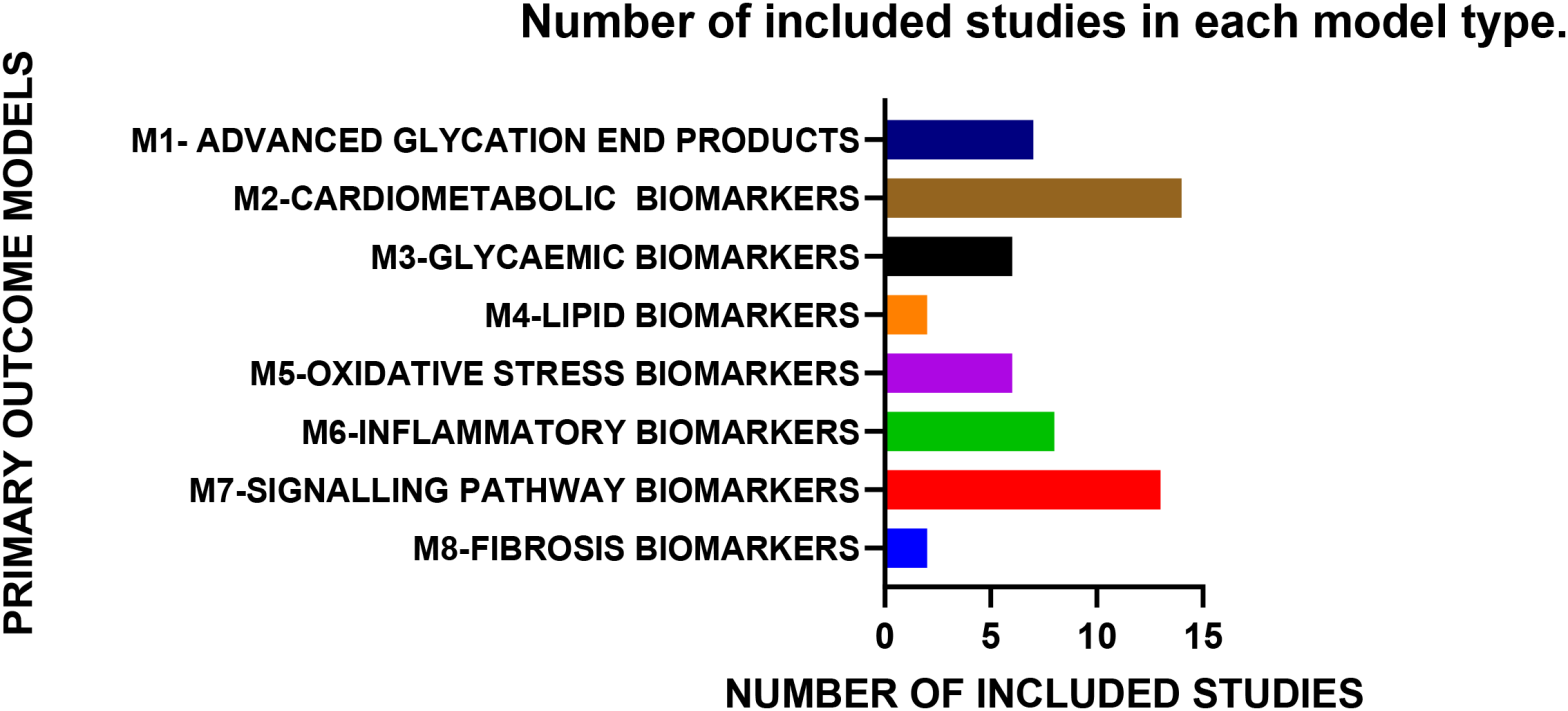
Number of included studies in each primary outcome model. The 29 included studies were allocated to 8 biomarker models and whether those biomarkers carried a high or low risk of manifesting DCM in the present rodent model was tested. The highest number of included studies (>12) are included in the cardiometabolic and signalling pathway biomarker models in this study.

**Figure 9:**
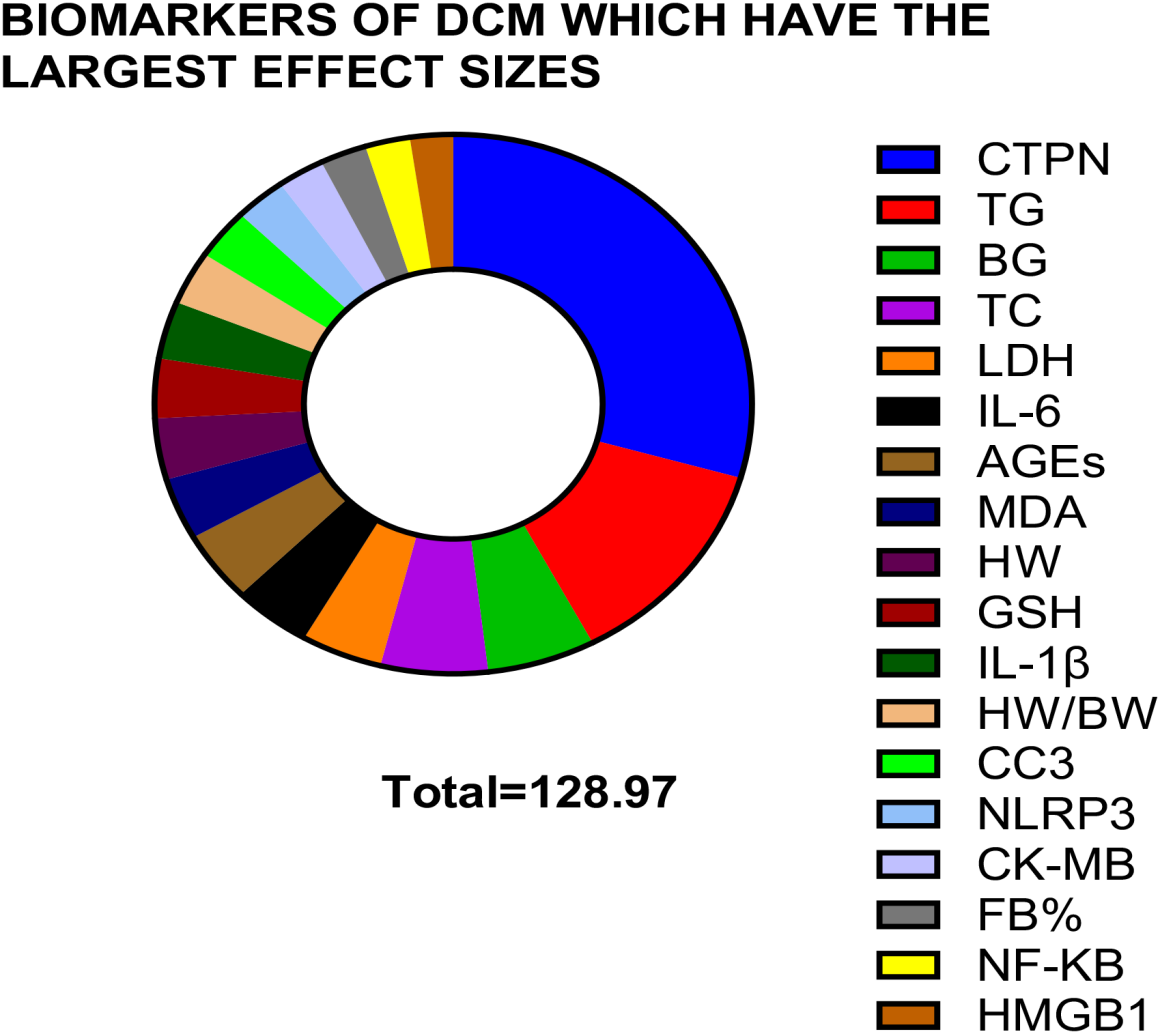
The biomarkers of DCM which have the significant largest effect sizes in descending order based on the forest plots. Cardiac troponin is featured as the biomarker which displayed the largest effect size or the standardized mean difference (SMD). CTPN (SMD 37.93, *p* = 0.03), TG (SMD 16.60, *p* = 0.0007), BG (SMD 7.54, *p* = 0.00001), TC (SMD 7.48, *p* = 0.008), LDH (SMD 5.66, *p* = 0.0005), IL-6 (SMD 5.49, *p* = 0.00001), AGEs (SMD 5.43, *p* = 0.004), MDA (SMD 4.78, *p* = 0.00001), HW (SMD 4.73, *p* = 0.05), GSH, (SMD -4.60, *p* = 0.009), IL-1β (SMD 4.44, *p* = 0.003), CC3 (SMD 3.91, *p* = 0.002), NLRP3 (SMD 3.59, *p* = 0.03), CK-MB (SMD 3.28, *p* = 0.004), FB% (SMD 3.25, *p* = 0.01), NF-KB (SMD 3.13, *p* = 0.0001) and HMGB1 (SMD 3.00, *p* = 0.0001) feature as the best 16 biomarkers of DCM based on the rodent models of this study.

This study has investigated 8 biomarker models (**Figure 8**) comprising of 37 biomarkers to understand their action within diabetic preclinical models and to evaluate the potential to diagnose or treat DCM by examining the statistics of those markers (**Figure 9**). Secondly, it evaluated the effect of the HMGB1 inflammatory biomarker on those clinical markers of DCM and compiled the correlation coefficient between HMGB1 and the rest of the markers. In computing the correlation coefficients, both Pearson’s and Spearman’s the limitation was not being able to obtain individual data but the mean values of each included study that reported each biomarker. This leads to the deficiency of depicting the ecological correlation coefficient that had risen from ecological fallacies, particularly in not being able to compute simple linear regression analysis between HMGB1 and the individual biomarkers.

*Diabetes mellitus* is a metabolic disorder caused by prolonged untreated hyperglycaemia and/or dyslipidaemia in the laboratory-bred rodent populations that include the rats and mice fed with a high-fat diet (65). The fasting blood glucose levels above 75 mg/dl and above for rats or 200 mg/dl for mice confirm the induction of diabetes in these animals (66). In experimental rodents, diabetes is induced by the intraperitoneal injection of streptozotocin given 50 mg/kg as a single dose to adult mice aged over 8 weeks (67). The induction of diabetes in the mice is confirmed by a fasting oral glucose tolerance test (68). A high blood glucose levels directly impact the metabolism in the liver, skeletal muscles and the vasculature as the development of CVD is a major adverse outcome of diabetes be it in the rodents or in the humans (68). Mice develop extreme thirst, frequent urination with high glucose excretion, extreme hunger, gastric dysfunction, unexplained weight loss and fatigue for which insulin treatment is considered as the remedial measure (69).

The DCM model 1 consists of 2 biomarkers which showed significantly high risk of manifesting diabetic-ED in this study: AGEs (SMD 5.43, *p* = 0.004) and HMGB1 (SMD 3.00, *p* = 0.0001). HMGB1 acts as a ligand which binds with the receptor for the AGEs, known as RAGE (**Supplementary Figures S1–1**, **S1-2**; **Supplementary Table S3-1**).

The DCM model 2 consists of 10 heart-related biomarkers (HR, HW, HW/BW, EJF%, FS%, LVIDD, LVIDS, LVDV, LVSV, BP) with which the risk of developing DCM in mice and rats were evaluated. DCM can cause abnormalities in the heart rate (HR) with elevated resting values that could increase the risk of heart failure and premature mortality in people with diabetes (70). Cardiac autonomic neuropathy is characterized by impaired heart rate control that could develop as a primary complication of diabetes caused by an increase in the sympathetic nervous control and a reduction in the corresponding parasympathetic coordination (115). Diabetic neuropathy is a primary adverse disease outcome of diabetes that affects the nerves supplying the microvasculature causing nerve-mediated vascular abnormalities (116). Cardiomyopathy is any disorder that affects the cardiac tissue, and it impairs the ability of the heart to pump the blood well (8). This condition may also lead to heart arrythmias, which is developing irregular heartbeat (117). The average heartbeat of a healthy conscious, unrestrained mouse and rat are 500–700 bpm and 250–400 bpm respectively (118). In this study, the heart rate (HR) was reported by only 3 included studies. The HR study showed a moderate increase (48.34%) in the diabetic cohort. The combined total average HR for the pooled diabetic rodents was 327.33 bpm as opposed to 220.66 bpm in the control counterparts. These values vary depending on the time of day, environmental factors and the activity level of the animals. Since these are restrained, rodents held in an animal facility, sometimes in crowded cages, their emotional wellbeing is disturbed, given the fact that anxiety contributes to greater variation in the HR. Additionally, the age, sex and the ambient temperature are factors which cause a variable HR with it decreasing with increasing age and the females having higher HR than the males (**Supplementary Figure S1-3**; **Supplementary Table S3-2**).

The heart weight (HW) of a mouse varies among the mouse line or the strain. This study included mostly the C57BL/6J mice with one subspecies (C57BL/6J-KsJ). In this study, the heart weight (HW) was reported by only 4 included studies. The HW study showed a moderate increase (58.78%) in the diabetic cohort. The combined total average HW for the pooled diabetic rodents was 486.72 g as opposed to 306.53 g in the control counterparts. The normal HW of a mouse falls between 150–180 g, while the diabetic HW of the rats is between 180–220 g, although these values are considered to be highly variable. The study which stated the abnormally high HW had fed the animals a high-fat diet containing 34.5% fat, 17.5% protein and 48% carbohydrate that was continued up to 4 weeks. Several such other studies have reported myocardial impairment due to changes in the contractile proteins, specifically myosin in the myocardium and the papillary muscles of the heart (119) (**Supplementary Figure S1-4**; **Supplementary Table S3-2**).

The HW is well-coordinated with the body weight (BW) in the rodents, and the HW/BW ratio is often considered as an estimate which measures the heart health, particularly the extent of cardiac hypertrophy, which is a progressive DCM condition in the animals. It usually takes a value of 3.3 to 3.8 mg/g for every 100 g increase in the BW for rats, and it is based on the body weight fluctuations. The cardiac hypertrophy can be detected by the ratio of the dry weight of the heart to the total BW. As the rats age, the BW increases too, reducing the HW/BW ratio. The patients undergone myocardial infarction displays a higher HW/BW ratio. Some investigators measure the HW against the tibial length, which does not fluctuate like the BW, but remains constant after reaching maturity, indicating the heart’s morphometric changes had taken place due to ageing. The average HW to BW ratio of the rodents is around 5 to 6 mg/g which increases in DCM conditions as well as with ageing and hypertension. The overall findings of the HR, HW and the HW/BW ratio, was that all showed neither a high risk nor no risk for the manifestation of DCM as evident in the forest plots and the t test bar graphs (**Supplementary Figure S1-5**; **Supplementary Table S3-2**).

Among the heart-related other biomarkers is the ejection fraction (EF%) and the fractional shortening percentage (FS%) which are other important estimates that indicate the heart functions and are risk factors of heart failure. The EF% is the volume of blood that is pumped out of the left ventricle in one heartbeat or one cardiac cycle. It is between 60 -90% in mouse and is slightly elevated than the rats. Also, the mouse EF% is considered closer to the values of a healthy human heart. An EF% below 30 is a matter of concern as a failing heart is indicated by a decrease in the cardiac output. In a mouse study of the heart functions, the HR and the cardiac index showed positive correlation with the cardiac output, which is approximately 20.4 ± 3.4 mL/min for a mouse. There is no perfect correlation between the EF% and other parameters of heart function in both the rodents and the humans. The median left ventricular ejection fraction in Sprague Dawley rats was 40.3% with compensated heart failure, and the EF% in Wistar rats in one study was approximately 88%, indicating that these values are highly variable between the species. The present heart -related analysis contained 12 rodent studies, in which 3 were rat studies and the other 9 studies consisting of mice. The average decrease in EF% was 26.2% when compared between the diabetic animals and the healthy control counterparts. When taken separately, the rats showed a mean 34.2% decrease, and the mice recorded a mean 17.4% decrease in the EF% in comparison to the healthy control. All these decreases were significant at p<0.05 (**Supplementary Figure S1-6**; **Supplementary Table S3-2**).

Fractional shortening percentage (FS%) is a measure of the contraction of the left ventricle during systole or how much the left ventricle decreases in size during systole. A normal FS% value is greater than 28% and it indicates the status of the left ventricular function. This model included 8 rodent studies in which there were 3 rat studies and 5 mouse studies. The overall mean FS% in the diabetic rodent cohort was 33.34% compared to the 46.86% of the healthy control and when the mouse group taken separately, displayed a 30.63% decrease while the rat group exhibited a 25.51% decrease in comparison to the healthy control groups. The mean FS% in the mouse diabetic cohort was 28.23% and, in the rat, diabetic cohort it was 48.66% whereas the control values were 40.70% and 65.33%, respectively. The FS% in the diabetic rat group appeared to be not affected by the induced DCM although the mice were affected. All these decreases were significant at p<0.05 (**Supplementary Figure S1-7**; **Supplementary Table S3-2**).

The left ventricular internal dimension in diastole (LVIDD) is a measure of the left ventricular enlargement which exposes the subjects to developing DCM and subsequent heart failure (120). In mice, the LVIDD ranges between 1.7 to 2.5 millimetres and is used to evaluate diastolic dysfunction in preclinical models. It is also utilized in determining the left ventricular filling velocity, isovolumic relaxation time, the left atrial size and the E wave deceleration time. The included studies comprised of 7 murine models and one rat model. The average LVIDD in the healthy control mouse models was 2.99 mm and the average of the diabetic group was 3.75 mm, with an increase of 25.42% in the diabetic mice. The left ventricular internal dimension in systole (LVIDS) in the mouse also ranges from 1.5 to 2.6 mm (**Supplementary Figures S1**-**8**, **S1**-**9**; **Supplementary Table S3-2**).

The left ventricular end-diastolic volume (LVEDV) gives the amount of blood in the left ventricle just before systole occurs, or when the ventricles contract. It is also known as the cardiac preload, with which the EJF, and the stroke volume can be measured by an electrocardiogram. This together with the end-systolic volume, abbreviated as LVESV and the LVEDV are important parameters of heart function. Also, they are indicators of ventricular compliance and diastolic filling time. The cardiac muscle fibre stretches increasing the blood volume within the left ventricle which could develop cardiac hypertrophy in the ventricular wall and the septum, leading to cardiac remodelling. It is also a measure of atrial enlargement which is a sign of progressive premature heart failure. The forest plot of LVSV showed a high risk of developing DCM with a significant high effect size measured by the SMD 1.38; 95% CI -1.38, 4.13; *p* = 0.33. This measurement is used to estimate the adequacy of cardiac emptying related to the systolic function (**Supplementary Figures S1**-**10**; **S1**-**11**; **Supplementary Table S3-2**).

The DCM model 2 also included the heart-related biochemical biomarkers; CK-MB, Cardiac troponin and LDH. Creatine kinase in the muscle and brain tissue (CK-MB) is an important biochemical biomarker of DCM and an enzyme involved in the process of cellular energy homeostasis (101). It catalyses the transfer of phosphate groups between ATP and creatine in the synthesis of a pool of phosphocreatine in the mitochondria acting as a buffer stock of ATP when the energy demands fluctuate such as during a bout of physical exercise (102). CK-MB is localized to the cytoplasm and the cellular membrane in the skeletal muscles, brain, and the myocardium in high concentrations (103). The mice that are deficient in CK have impaired voluntary running capacity and cardiac and skeletal muscle degeneration (104). The CK levels are measured in blood and elevated CK concentrations indicate muscle injury, heart attack or brain damage (104). Seven rodent models are included in the present study that have measured the blood titres of CK-MB which has indicated a significant considerable risk of developing DCM in the two mouse models and the five rat models that were included (SMD 3.28; 95% CI 1.04, 5.52; p<0.00001) (**Supplementary Figures S1**-**12**; **Supplementary Table S3-2**).

The lactate dehydrogenase (LDH) is a biochemical biomarker of DCM, an important enzyme prevalent in the cell cytoplasm in all the organs and tissues in the human body, mouse and rat models (110). It is an oxidoreductase enzyme which catalyses the anaerobic cellular respiratory pathway, with converting the lactate to pyruvate and reducing the NAD to NADH. LDH is also present in the erythrocytes and the reticuloendothelial system (111). It is readily available in muscles, liver and kidney and is associated with anaemia and heart attacks. It is best tested along with the CK-MB and cardiac troponin and serves as an energy source in oxygen-deficit conditions in the muscle tissue such as during strenuous physical exercise (112). The average range of LDH in mouse plasma is 140 to 280 units per millilitre and in rat plasma, it is 61–121 U/L (113). The overall outcome has a high effect size and carries a significantly high risk of developing DCM in a forest plot (SMD: 5.66; 95% CI 2.48, 8.84; p<0.0005) (**Supplementary Figure S1-13**; **Supplementary Table S3-2**).

Cardiac Troponin (C-TN or C-TPN) is an enzyme present within the cardiac muscle tissue, and its levels in blood rises as the pathological damage to the cardiac tissue is increased (105). The troponin I and troponin T are the variants which are measured separately with a blood test (106). When troponin level in blood is greater than 10 ng/ml, it is an indication of becoming exposed to a heart attack or being at a very high risk of exposure to developing DCM, in both the mouse and rat CVD models (107). Apart from developing DCM, there are other pathological conditions that increase the troponin levels in blood such as other heart diseases, drug toxicity, myocardial infarction and sepsis (108). The healthy control will only contain very low levels of troponin, being unable to detect it in blood for not having a test that is sensitive enough for its detection (109). In the present study, there are only 4 rodent studies that had measured cardiac troponin as part of their investigations on DCM in mouse and rat models. Based on their units of measurements and having >8 numbers of mouse or rat, the data was segregated in the forest plot into subgroups, The effect size nevertheless was high and the difference between the healthy control and diabetes groups of cardiac troponin was significant, thus making it a reliable biomarker for evaluating the risk of manifesting DCM in clinical subjects as well as evaluating the molecular mechanism of troponin to assess its therapeutic potential (SMD: 37.93, 95%CI 4.28, 71.58; P< 0.0001) (**Supplementary Figures S1**-**14**; **Supplementary Table S3-2**). Blood pressure was measured in the rodents which had 3 included studies but showed no significant difference between the DCM and the HC ((**Supplementary Figure S1-15**; **Supplementary Table S3-2**).

The DCM model 3 comprising the glycaemic estimates of the rats and mice were obtained from monitoring the blood glucose level, the fasting insulin sensitivity, and the body weight (71). A total of 12 included rodent studies comprising 93 animals evaluated the risk of manifesting DCM by monitoring the blood glucose level. The mean blood glucose level in the diabetic group was 23.67 mmol/L and 901.31 mg/dl whereas the healthy control mice had a corresponding mean of 7.047 mmol/L and 300.085 mg/dl which signified an average percentage increase of 235.88% and 200% respectively. These reported values were further confirmed by the estimates obtained from the forest plot which recorded a SMD of 7.54 (indicating a large effect size) having a 95% CI containing 4.97, 10.12 at a significant level of p < 0.00001 (**Supplementary Figures S1**-**16**, **Supplementary Table S3-3**).

The fasting insulin sensitivity test is carried out to determine the insulin -responsive tissues in the rodent body (68). It is a measurement of the glucose that is remaining in circulation after the deliverance of insulin intraperitoneally as a bolus (a single large dose) (72, 73). The intermittent fasting is said to improve the tissue insulin responsivity as well as the blood pressure and the oxidative stress levels without causing weight-loss (74). In order to improve the tissue responsivity to insulin, a fasting protocol has been introduced to experimental mice, which is giving access to food for only 5 out of 7 days of the week (75). The present analysis had only 4 rodent studies that had assessed the insulin sensitivity of the mouse tissues, which indicated no real impact of insulin treatment on causing DCM in the mice (SMD: -9.46, 95% CI -27.72, 8.80; *p* = 0.31). However, this result remains inconclusive due to non-significant and insufficient availability of data (**Supplementary Figures S1**-**17**; **Supplementary Table S3-3**).

The body weight estimates were also inconclusive due to fluctuating murine body weight data, which in some animals recorded a loss while there were instances of weight gain as well. The overall outcome was that the body weight did not cause a significant impact on the determining of DCM manifestation in mice (SMD -1.74; 95% CI -3.46, -0.02; *p* = 0.05) (**Supplementary Figures S1**-**18**; **Supplementary Table S3-3**).

The DCM model 4 consisted of the serum lipid estimates, TC and TG of this rodent study. Both serum total cholesterol (TC) and the total triglycerides (TG) in the rodent models showed a significantly high risk of developing DCM when the mice and the rat data were pooled together (TC: SMD 7.38, *p* = 0.009; TG: SMD 16.60; *p* = 0.0007. The TC includes the HDL-C, LDL-C and the TG, where lipids play a vital role in the humans, developing atherosclerosis, and other heart diseases including heart attacks and cerebrovascular events (76). The lipids constitute the major components in the cell membrane and accumulating in the adipose tissue and around the vital organs for protection and participate in cellular energy generating mechanisms being a surrogate respiratory substrate in the event of glucose depletion (77). The mean total TC in this study in the diabetic group was 4.165 mmol/L and the mean total TC of the control was 1.895 mmol/L, indicating a 119.7% increase in the diabetic rodents in comparison to the control. Similarly, the TG level in the diabetic group was raised by 194%compared to the control (**Supplementary Figures S1**-**19**, **S1**-**20**, **Supplementary Table S3-4**). The HDL-C and LDL-C were not included since they had only one study each (**Supplementary Figures S1**-**21**; **S1**-**22**; **Supplementary Table S3-4**).

The DCM model 5 included the oxidative stress biomarkers, GSH and MDA. The serum glutathione concentration is an important biomarker of oxidative stress and the heart-related abnormalities that will occur within the diabetic cohort (78). Glutathione is a tripeptide substance produced by the liver consisting of the amino acids glycine, glutamic acid and cysteine (79) It functions as a protector of skin blemishes, liver detoxification and immune reactions (80). Glutathione peroxidase is an antioxidant enzyme which is a scavenger of free radicals and reduces: oxidative stress, coronary artery disease, lipid peroxidation, maintaining redox balance and intracellular homeostasis (81). Glutathione concentration in blood was assessed by only 4 studies and reported in 3 different units; nanomoles, micromoles, and millimoles per milligrams or grams which were included in a forest plot as 1 group measuring GSH as nanomoles. In our findings, glutathione concentration decreased more in the diabetic mice as opposed to the healthy control mice. It showed a negative correlation with DCM in the rodents tested (SMD -4.60; 95% CI -8.04, -1.16; *p* = 0.009) (**Supplementary Figures S1**-**23**; **Supplementary Table S3-5**).

Malondialdehyde (MDA) is a biomarker of oxidative stress in diabetic CVD which is produced from lipid peroxidation of the polyunsaturated fatty acids that promoted atherosclerotic plaque buildup in diabetic mice (82). Further, high levels of MDA can produce stiffening of collagen fibres associated with diabetic CVD (83). The elevated MDA levels could be treated with an extract of *Ficus* and the flavonoid -containing substance chrysin (84). This study included four rodent models in which MDA was quantified, which also had produced a significantly high MDA concentration in each. The total mean value of the pooled MDA in the healthy control mice was 28.85 nmol/g and in the diabetic cohort, it was 43.59 nmol/g which showed a percentage increase of approximately 51% between the two groups (SMD 4.78; 95% CI 2.71, 6.86; p < 0.00001) (**Supplementary Figures S1**-**24**; **Supplementary Table S3-5**).

The DCM model 6 included the inflammatory biomarkers; TNF-a, IL-6 and IL-1β. Tumour necrosis factor-alpha (TNF-a) is a pro-inflammatory cytokine that can cause both acute and chronic inflammation (85). It is produced by monocytes and macrophages and plays a role in the pathogenesis of dilated cardiomyopathy, myocardial fibrosis and in certain autoimmune diseases (86). The mRNA and protein of TNF-a are present in the myocardium and the endomyocardial endothelium in the heart and it is also a constituent of the immune system (87, 88). TNF-a promotes cell proliferation, as well as apoptosis and necroptosis of the cardiomyocytes in response to its cell signalling mechanisms related to the dilated cardiomyopathy condition (89). In the present study, TNF-a has produced an overall large effect size and is a biomarker carrying a high risk of developing diabetic cardiomyopathies in the seven rodent models included in this study. The total average of the TNF-a concentration quantified in serum in the diabetic cohort was 94,287.52 pg/ml and 31277.37 pg/ml in the healthy control group confirming that the TNF-a titre in rodent serum was increased by 201.4% due to diabetes, comparative to the control group (SMD: 1.11; 95% CI 0.11, 2.11; *p* = 0.03) (**Supplementary Figure S1-25**; **Supplementary Table S3-6**).

Interleukin-six (IL-6) is another inflammatory cytokine which plays a significant role in the pathogenesis of DCM in humans as well as the rodents (90). IL-6 tends to increase the collagen content leading to myocardial fibrosis and increasing collagen in the cardiac fibroblasts *via* enhancing the TGF-β and inhibiting the miR29 pathway (91, 92). Blocking IL-6 receptor had reduced the pathogenicity of DCM and interstitial fibrosis by inhibiting SOCS -3, an immune signalling marker (92). The IL-6 induces insulin resistance in brown adipose tissue and synergizes with other pro-inflammatory cytokines to exacerbate beta cell damage in the pancreas (93). In the 6 included rodent studies which measured the IL-6 titre in rodent serum, the average of the diabetic group was 83,385.21 pg/ml and the average in the healthy control group was 36,704.43 pg/ml, which was an increase of 127.18% between the two groups (SMD: 5.49; 95% CI 3.35, 7.64; p<0.00001) (**Supplementary Figure S1-26**; **Supplementary Table S3-6**).

Interleukin-one beta (IL-1β) contributes to the cardiac electrical disturbances, arrythmias and heart failure in mouse models, thus playing an important role in the manifestation of DCM in the rodents (94, 95). It is a prominent proinflammatory cytokine which impairs insulin secretion and beta cell apoptosis leading towards T2D. Furthermore, inflammation is a key process of the unstable plaque transition resulting in atherosclerosis in which IL-1β performs a significant role as an inducer of sterile inflammation in several ischemic incidents (96). In the present analysis, 5 rodent studies reported the IL-1β titres in serum and/or tissue and the mean total of a subgroup of the diabetic group with 20 animals was 57.42 pg/ml and the matching mean total in the healthy control was 34.115 pg/ml, which indicates an increase of 68.31% between the two treatment groups (**Supplementary Figure S1-27**; **Supplementary Table S3-6**).

The DCM model 7 included the biomarkers of the molecular mechanisms; NF-kB (**Supplementary Figure S1-28**; **Supplementary Table S3-7**), TLR4 (**Supplementary Figure S1-29**; **Supplementary Table S3-7**) and Cleaved Caspase 3 (**Supplementary Figure S1-30**; **Supplementary Table S3-7**) and NLRP3 inflammasome (**Supplementary Figure S1-31**; **Supplementary Table S3-7**), ratio of phosphorylated ERK 1/2 to the total ERK 1/2 (**Supplementary Figure S1-32**, **Supplementary Table S3-7**), phosphorylated JNK to total JNK ratio (**Supplementary Figure S1-33**; **Supplementary Table S3-7**), TGF-1β (**Supplementary Figure S1-34**; **Supplementary Table S3-7**). Nuclear factor kappa beta (NF-kB) is a pleiotropic transcriptional factor which is present within the cell nucleus and activates molecular mechanisms related to oxidative stress, inflammation, endothelial dysfunction, myocardial hypertrophy and fibrosis and cardiomyocyte apoptosis (97). One of its underlying mechanisms is the diabetes-associated myocardial remodelling and myocardial hypertrophy (98). A plethora of research work has been carried out on this nucleus-bound DCM biomarker and also its multiple molecular pathways which are interconnected and have been characterised (PI3K/AKT/MAPK) although its inhibition had not produced an effective anti-DCM outcome that could be utilized as a valid therapeutic target. Five rodent studies were included in the forest plot of NF-kB and have confirmed that it carries a high risk of developing DCM in the diabetic cohort. The mean total of the diabetic group with 3 studies was 6.31 AU and the mean total in the healthy control was 1.0 AU, which indicated an increase of 531% between the two treatment groups.

Toll-like receptor -four (TLR4) are innate immune activators which upregulate leucocyte infiltration into the myocardium and as a consequence, stimulates the secretion of cytokines, promotes oxidative stress, and releases proteases which in turn induce myocardial infarction or a heart attack, ischemic-reperfusion injury, myocarditis and heart failure. The activation of TLR4 on dendritic cells leads towards acute myocarditis. Similarly, the activation of TLR4 and its downstream markers leads to autoimmune myocarditis. There were 5 included studies in the analysis of DCM and the average of the total TLR4 measurements amounted to 3.38 relative expression in the diabetic group while the mean total of the healthy control group, was 0.79, which displayed a 327% increase in the diabetic cohort. (**Supplementary Figure S1-29**; **Supplementary Table S3-7**).

Cleaved Caspase 3 (CC3) is a biomarker which signifies cell death by apoptosis, and it is an indicator of the loss of cardiomyocytes that cause damage to the myocardium. The cardiac muscle weakens and enlarges when undergoing apoptosis, leading to DCM. CC3 is a protease which cleaves by itself and upregulates apoptosis (99). In this study, 4 included research studies reported relatively high CC3 titres in rodents induced with DCM compared to the controls (SMD 3.91, 95% CI 1.46, 6.36, *p* = 0.002). (**Supplementary Figure S1-30**; **Supplementary Table S3-7**).

NLRP3 inflammasome is a member of the innate immune system which initiates DCM when its functions are dysregulated. It is a prominent biomarker of cell death by pyroptosis. Dysregulated NLRP3 produces pro-inflammatory cytokines, IL-1β and IL-18, which serve to exacerbate myocardial damage initiating widespread inflammation within the cardiac tissue The activation of the NLRP3 inflammasome is related to several high-risk factors of DCM such as hyperlipidaemia, diabetes, obesity and hyperhomocysteinemia (100). In this study, NLRP3 was identified as a high-risk biomarker for manifesting DCM (SMD 3.59, 95%CI 0.28, 6.89 *p* = 0.03) although the outcome was not significant in the t-test. NLRP3 titres were measured by 4 included studies, and it displayed a non-significant increase in the diabetes cohort compared to the controls in the t-test bar graph. (**Supplementary Figure S1-31**; **Supplementary Table S3-7**).

The ratio of phosphorylated ERK 1/2 to the total ERK 1/2 plays an important role in the manifestation of DCM as it is linked to various pathological changes in the diabetic heart tissue. Chronic hyperglycaemia activates the Ras/MEK/ERK pathway that leads to the phosphorylation of the ERK 1/2 molecules which promote cardiomyocyte dysfunction and apoptosis, that progress into DCM. ERK activation leads to various disease conditions by promoting collagen synthesis resulting in cardiac fibrosis, disruption of mitochondrial activities by reducing the synthesis of ATP and releasing ROS, upregulating ER stress, the activation of JNK signalling and altering the signal cascades related to myocardial dysfunction resulting in DCM. Further, the inhibition of MEK (with UO126) and subsequently the ERK 1/2 has led to the attenuation of experimental DCM by improving cardiac function and reducing cardiomyocyte damage (101) (SMD 0.46; 95%CI, -0.67, 1.59, *p* = 0.42). (**Supplementary Figure S1-32**; **Supplementary Table S3-7**).

The phosphorylated JNK to total JNK ratio is another biomarker of importance, which is activated by high blood glucose and inflammation, which are the hallmarks of diabetes that can progress into DCM. The activation of JNK or c-Jun-N-terminal kinase upregulates insulin resistance, cardiac fibrosis and cell death. Stimulating its phosphorylation and reducing its titres in cardiac tissue and blood would act as a deterrent to increasing inflammation and subsequent progression into DCM (102). This study has tested the pJNK/t-JNK ratio by constructing a forest plot with 5 included studies, displaying a mean value of 1.0 and 2.63 measured in western blots against β-Actin in the control and DCM cohorts respectively. The DCM cohort that was observed showed an increase of over 163% between the control and the DCM groups which used a total of 66 animals (SMD 1.87, 95%CI 0.95, 2.79, *p* = 0.0001). (**Supplementary Figure S1-33**; **Supplementary Table S3-7**).

Transforming growth factor – beta (TGF-β) is an important mediator of the molecular signalling pathways that are closely linked with fibrogenesis. Fibrogenesis is known to progress into myocardial impairment leading to DCM through the SMAD -dependent pathways and independent pathways such as the non-canonical MAPK. The hyperglycaemia in diabetes causes the activation of the TGF-β genes, proteins and receptors which in turn promote cardiac fibrosis which is one of the DCM phenotypes (126). The average TGF-β level in the healthy control was 0.966 units measured with beta-actin as its comparator using the western blot technique and an average of 2.66 units in the DCM cohort that indicated a 175% increase in the diabetic animals, producing a significant difference in the forest plot (SMD 1.50;.95%CI; -0.03, 3.03, *p* = 0.05). (**Supplementary Figure S1-34**; **Supplementary Table S3-7**).

Myocardial fibrosis is a major contributor to heart failure in the diabetic subjects, and they are more prone to developing DCM with the scar tissue that is formed in the heart wall, particularly in the myocardium. TGF-β is a major player in myocardial fibrosis by upregulating collagen synthesis and inhibiting collagen degradation, while the production of TGF-β is stimulated by high blood glucose levels and insulin resistance which are the main characteristics of diabetes which also promote the manifestation of DCM. The cardiac fibroblasts are the main cell type in the heart wall that produces collagen, which is stimulated by hyperglycaemia, lipotoxicity and the release of pro-inflammatory cytokines. The activation of TGF-β is also caused by the advanced glycation end products and the renin-angiotensin-aldosterone system. The neurohormones, growth factors and cytokines too contribute towards inducing fibrosis in the cardiac tissue in diabetic patients. Oxidative stress and inflammation are other contributory factors of fibrosis which damage the wall structure and stiffen cardiac muscle thereby impairing the relaxation of the heart muscle, leading to diastolic dysfunction. The diabetic conditions also activate pro-fibrotic pathways which cause dangerous arrythmias in the heart by interfering with its electrical conduction system (103). This study investigated the Fibrosis percentage as a biomarker of DCM in 4 studies which utilized 32 animals induced with diabetes. The control cohort had a mean value of 4.8 as opposed to the DCM group which had a higher mean value, 26, increased by 441% in the DCM cohort (SMD 3.25, 95%CI 0.77, 5.74, *p* = 0.01) (**Supplementary Figure S1-35**; **Supplementary Table S3-8**).

Collagens I and III are major structural proteins present within the cardiac tissue, which maintains the cardiac muscle strength and offers support during heart functions. The deposition of collagen in cardiac tissue is known as fibrosis. Excessive collagen deposition impairs the cardiac tissue leading to myocardial fibrosis. Cardiac fibroblasts in the heart wall produce collagen when stimulated by hyperglycaemia (104). This study tested and rated the fibrosis percentage, collagen I and III levels in heart tissue to understand its impact on DCM as a risk factor. Each of these three biomarkers had 6 studies each and carried a significantly high risk of developing DCM in the forest plots but not in the non-parametric Mann Whitney U test. Interestingly, the percentage increase in Collagen I between the control and DCM cohorts was observed at 436% (SMD 2.31, 95%CI 0.62, 3.99, *p* = 0.007) and 276% for Collagen III (SMD 2.43, 95%CI 0.59, 4.27, *p* = 0.010). (**Supplementary Figures S1**-**36**, **S1**-**37**; **Supplementary Table S3-8**).

### 3.3 Quality of evidence

PRISMA 2020.

### 3.4 Potential biases of the review process

This study was conducted by this author, under the guidance of an expert in this method and the research articles were identified from the databases with the same set of keywords, then by reading the title and the abstract of each hit for relevance based on the inclusion and the exclusion criteria which were pre-determined. Whenever there was a doubt or a problem in selecting the included studies, the expert was consulted, and the doubt was resolved through discussion. The full text of the articles which conformed to the inclusion and exclusion criteria were read and selected and the data was extracted onto an MS Excel sheet for tabulation and was exported to the RevMan software for the construction of forest plots for each of the biomarkers which totalled 36. The continuous data followed a random effects model, the data included being biochemical and metabolic biomarkers which were produced continuously by the body. The SYRCLE tool of ROB which is more relevant to preclinical studies was utilised and applied to each individual study which is displayed in the ROB summary that is provided. Randomisation prevailed in all the studies but blinding of the investigators was not mentioned in most of them. Given the fact that the rodents utilized were ear-clipped, blinding of the investigators was considered meaningless. There was no evidence of selective reporting or incomplete reporting. The forest plot also calculated the I squared metric alongside the Tau squared and the Chi squared metrics, all of which were tests for heterogeneity of the data that was evaluated. The forest plots calculated the standardized mean difference or the effect size with the weighted average for each biomarker displaying the size of the intervention effect in each study relative to the variability observed in that study. As descried above, the present study had minimal ROB and did not contain any major confounding effects in it. In the funnel plots of each risk factor, the studies with larger effect sizes accumulated towards the top of the funnel plot while the studies having smaller effect sizes gathered towards the bottom. The overall ROB was moderately low with three out of 10 parameters in the SYRCLE ROB tool showing up high risk, which did not mention the blinding of investigators (detection) blinding of performance and allocation concealment in all the 29 included studies. Randomization, random housing, baseline characteristics, blinding of the outcome measures, selective reporting, publication and other forms of bias were minimal. A PRISMA 2020 checklist was produced for minimising or removing confounders and increase the validity of the present study. Funnel plots were constructed for the detection of publication bias with computing the Q statistic and the receiver operator curves (ROC) to prove there were no major confounders.

### 3.5 Agreements and disagreements with other studies or reviews

The circular RNA, also known as non-coding RNA, has been more stable, ubiquitous and has longer half-life in providing a treatment strategy for DCM, more than linear RNA or the mRNA on account of it exerting an inhibitory effect on DCM induced in mice. DCM is characterized by spontaneous cardiac dysfunction progressing into cardiac hypertrophy, fibrosis and remodelling following early onset of diastolic dysfunction and late onset of systolic dysfunction. Although there was no involvement of circular RNA in our study, DCM was associated with left ventricular diastolic dysfunction (LVDV: SMD: 4.93, 95%CI 3.35, 6.50, *p* = 0.00001) followed by systolic dysfunction (LVSV: SMD: -1.38, 95%CI -1.38, 4.13, *p* = 0.33) between the DCM and the healthy control groups which confirm the establishment of DCM in mouse models which gradually progress into DCM in both mice and the humans. The myeloid differentiation -2 or MD2 is a co-receptor of TLR4 which exacerbates hyperglycaemia -induced inflammation upon binding of the AGEs leading to myocardial fibrosis (**Supplementary Figure S1-35**; **Supplementary Table S3-8**) a key characteristic feature of DCM. Mice deficient in MD2 have displayed inhibition of TLR4 resulting in reduced myocardial remodelling and attenuation of the severity of DCM pathology, thereby improving cardiac function. In the present study, TLR4 exhibited no difference (TLR4: SMD: 2.38, 95%CI 0.61, 4.15, p=0.009) between the healthy control and mice induced with DCM probably given the small sample size, but there was high elevation of the AGEs (AGEs: SMD: 5.43, 95%CI 1.73, 9.13, *p* = 0.004) (**Supplementary Figure S1**-**1**; **Supplementary Table S3-1**) directly binding with MD-2 which confirmed the synergistic activation of hyperglycaemia driven MD2, TLR4 and the AGEs, leading towards the establishment of worsened myocardial injury. Fibroblast growth factor-9 (FGF-9) was identified as a mediator which reduces vascular smooth muscle cell apoptosis in mice induced with a myocardial infarction (MI). This was proven by echocardiography which monitored the ejection fraction percentage (EF%) and the fractional shortening percentage (FS%) in comparing the two groups of diabetic and non-diabetic mice treated with FGF-9 after inducing MI. Our results of the EF% (EF%: SMD: -4.13, 95% CI -5.56, -2.69, *p* = 0.00001) and FS% (FS%: SMD: -2.91, 95% CI -3.96, -1.86, *p* = 0.00001) are in agreement with having decreased values in mice induced with MI, as opposed to the mice treated with FGF-9 after inducing MI, which displayed an improvement in the left ventricular output measured by the elevation of the EF% and the FS% as parameters of cardiomyopathy function compared to the non-diabetic controls. A study investigating the protective function of Ulinastatin in DCM had reported the downregulation of the pro-inflammatory cytokines TNF-a, IL-6 and HMGB1 with the improvement of cardiac function, which agrees with the outcome of the present study. It has demonstrated increasing levels of HMGB1 (HMGB1: SMD: 3.00, 95%CI 1.58, 4.42, *p* = 0.00001) (**Supplementary Figure S1-2**; **Supplementary Table S3-1**), TNF-A (TNF-A: SMD: 1.11, 95%CI 0.11, 2.11, *p* = 0.03) and IL-6 (IL-6: SMD 5.49: 95% CI 3.35, 7.64, *p* = 0.00001) in association with impaired DCM function. In a study of experimental autoimmune myocarditis induced by cardiac troponin-I immunization (CTPN: SMD: 37.93, 95% CI 4.28, 71.58, *p* = 0.03), both the biomolecules, HMGB1 and RAGE (not computed) were considered as potential therapeutic targets in heart failure treatment, given the ability of HMGB1 to stimulate immunity and their ability to interact with each other, RAGE being its principal binding partner in both preclinical and clinical studies. The blockade of HMGB1 had achieved attenuation of experimental autoimmune myocarditis (EAM) by producing a significant decrease in myocardial fibrosis, which was significantly elevated in mouse serum in this study given by the fibrotic % (FB%: SMD: 3.25, 95%CI 0.77, 5.74, *p* = 0.01), in which the secretion of HMGB1 by myocardial fibroblasts as well as normal fibroblasts was also reported (105).

### 3.6 New DCM theragnostic trends

#### 3.6.1 Proteomics and transcriptomics

Diabetic cardiomyopathy (DCM) is a disease nexus which could expeditiously progress into heart failure, and hence, cause premature mortality in the diabetic patients (106). For this reason, a vast number of therapeutic options central to developing diabetes and subsequent impairment of the cardiovascular system utilising traditional biomarkers and their molecular networks have been continuously evaluated (107). The recent progress in DCM drug development has shifted to exploring the transcriptomes and the proteomics with the motive of identifying the essential protein and lipid targets that are unique to the resolving mechanisms of DCM (108). The introduction of high throughput genomics and proteomics techniques have enabled the scientists to expand the classical studies into large databases of DCM-relevant genes and proteins (108).

Wei et al. in 2022 investigating the gene profile of a DCM mouse model revealed thousands of relevant genes from the Kyoto Encyclopaedia of Genes and Genomes (KEGG) analysis and that most of the important genes were found enriching lipid and amino acid metabolomic pathways (109). A differential gene analysis had shown 139 protein-based targets which had 67 upregulated and 72 downregulated in the DCM mouse model (109). The same study also investigated the effect of spermine, a naturally formed metabolite of the cellular processes, a small alkyl polyamine as a prospective new therapy which could downregulate certain proteins related to the molecular mechanisms that aggravate the severity of the disease. Alox15 (arachidonate 15-lipoxygenase), Pnpla2 (patatin-like phospholipase domain containing 2), pla2g12a (phospholipaseA2-group XIIA), Gm13033 (prostaglandin-endo-peroxide synthase 2 pseudogene), Ptges (prostaglandin E synthase), and Acot1 (acyl-CoA thioesterase1) are a set of protein-encoded genes which were either upregulated or downregulated within the molecular pathways of DCM (109).

DCM proteomics consist of identifying and quantifying of the cardiac proteome in either an animal or human model (110). It helps to assess their contribution to upregulating DCM pathogenesis and test the protein types and the abundance of each to relate the protein activity with the progression of DCM as a disease. There are several processes which are fundamental to DCM progression that also involve the proteome of the myocardium that will be altered in structure and quantity as a response to the diabetes-induced tissue damage in the heart (111). They consist of mitochondrial dysfunction affecting the cellular energy metabolism, calcium homeostasis in the heart affecting contractility of the myocardium, changes caused to the protein structure and function such as proteins in the extracellular matrix and contractile proteins, and post translational modifications that occur like O-GlcNAcylation and lysine β- hydroxybutyrylation which are dysregulated in the diabetic heart (112). Further, protein-interaction mapping allows the researchers to understand the protein interactions which take place between altered or changed proteins in the main metabolic processes of DCM (113).

Hamblin et al. in 2007 measured the glutathione levels and a prostaglandin in a STZ-induced chronic type 1 diabetic rat model after which the left ventricular proteomic profile was evaluated along with the haemodynamic parameters (114). They concluded that the upregulated levels of 8-iso PGF 2α and decreased glutathione (oxidized GSSG and reduced GSH) levels indicated a link between increased oxidative stress arising from lipid peroxidation and the downregulation of certain key proteins in the myocardium (114).

A transcriptome comprises of all the RNA molecules that are present within a genome at a specific time in a living cell or a collection of cells (115). It consists of all the messenger RNA (mRNA), transfer RNA (tRNA), ribosomal RNA (rRNA) and non-coding RNA pooled together which represents the active RNA component in a genome. The importance of identifying a transcriptome is it can relate to the activity of a given gene and the changes that take place in response to different stimuli. Hence, a transcriptome is an ever-changing dynamic snapshot of gene expression, and the study of transcriptomes is known as transcriptomics (116). Transcription is the crucial first step of synthesizing proteins by copying a segment of DNA into a composite messenger RNA sequence which carries the genetic instructions from the nucleus to the cytoplasm of a cell, where it is decoded by a translation process. A transcriptome is useful in identifying gene regulation as it highlights a given functional change in gene expression that is related to a biological process at a given moment (117). Also, there are other types of RNA that are transcribed from DNA which involve regulating cell structure and functions.

The function of many genes is still not known. There are two genome-based projects that are underway by the National Human Genome Research Institute (NHGRI) which include the mammalian gene collection initiative and the mouse transcriptomic project (118). The former involves genome mapping of humans, mouse and rat as they provide good models for the study of biology while the latter involves tissue-specific gene expression data of the mouse and rat species. One of the uses of knowing the transcriptomes of a given animal species is that when an antibody study involves false negatives in which case the gene is expressed but not captured by the test (119).

Wei et al. (2022) investigated both the transcriptomes and the proteomes in the myocardium in a STZ-induced DCM rat model, in which 37 protein – encoded genes were found co-expressed in which protein-protein interaction method delineated *Cyp1a1, Comt, Acox1, Hadhb, Hmgcs2, Acot2, Ephx2, Cyp1a1*, and *Acot1* as expressed in lipid metabolism while *Inmt* and *Cat* were involved in amino acid metabolic pathways (109). The remaining proteins interacted with each other and what is expressed above in regulating cellular functions related to DCM development. The *Acot1* was found regulating intracellular signal transduction, fatty acid oxidation and reducing cardiac toxicity through promoting anti-ferroptosis (109). Abudoureyimu et al. (2024) in their transcriptomic analysis of DCM identified 21,655 genes which showed significant separations among the sample groups (120). Genes of inflammation and pyroptosis were strongly implicated in the DCM gene profile which was downloaded from the Gene Expression Ominibus (GEO) database (120).

#### 3.6.2 Epigenetics of DCM

With advancing technological breakthroughs that have been achieved in the recent years, more attention has been focussed on to the epigenetic mechanisms that parallelly compliment the traditional approaches of testing biomarkers and present as viable alternatives in the quest for drug discovery. Epigenetics is a heritable intricate mix of pathobiological and environmental factors which impinge upon disease development and/or resolution without any changes taking place in the organismal DNA (121). Thus, several epigenetic regulatory networks have been investigated in cardiovascular diseases with the introduction of sequencing technology (122), and the three main epigenetic mechanisms constitute of DNA methylation, histone modification and the involvement of non-coding RNA (123).

DNA methylation produces dynamic changes in the transcriptomes such as hypermethylation at CpG islands accounting for up to 60% of the promoter region and effectively inhibiting gene expression by maintaining heterochromatin status, whereas the non-coding regions are negatively correlated with DNA methylation (124). The initiation and sustenance of the DNA methylation activities involve three DNA methyltransferases (DNMTs) whereas the removal of the DNA methylation process is achieved by the ten-eleven translocation enzyme families, the TETs, which are directly associated with cardiovascular dysfunction, fibrosis and the disruption of apoptotic signalling in the heart (125).

Diabetic status is highly related to the DNA methylation process which promotes vital pathological symptoms that are linked to the development of DCM (126). Calcium homeostasis in the cardiomyocytes and the myocardium, cardiac ageing, angiogenesis, genetic expression of glutathione peroxidase, a crucial antioxidant agent in cardiac metabolism, cardiomyocyte hypertrophy, the overactivation of the RAAS, ventricular hypertrophy and remodelling are all pathological processes of DCM which are also closely related to the DNA methylation process which affects the cardiovascular system (127).

Histone acetylation and deacetylation are also crucial epigenetic mechanisms which are proven processes that directly impact the DCM pathophysiology in both preclinical and clinical models (128). The most studied histone -modified mechanisms of DCM include heart failure, arrythmias, coronary artery disease, hypertension, endothelial hyperplasia and smooth muscle cell migration (129). Bromodomain -4 is a transcriptional regulator which promotes disease progression in DCM which can bind to the promoters of multiple metabolic genes and importantly regulate the p65 expression of the inflammatory NF-kB pathway in the beta cells (130). Histone lactation is a newly introduced concept which involves lactic acid as a precursor to forming lactylate histones that initiate early repair of post-myocardial infarctions and mitochondrial pyruvate carriers (131).

The non-coding RNAs are broadly of two types, the long non-coding RNAs and small non-coding RNAs (132). The former are not evolutionarily conserved, but the latter type are evolutionarily conserved molecules. Additionally, there are the micro RNAs which are critically involved in DCM development and progression while proving the circulating micro RNAs can serve as epigenetic blood-based biomarkers. They consist of the miRNAs 9, 21, 29, 30d, 34a, 144, 150, 320 and 378 out of which miRNA 21 has been targeted as a novel biomarker that provided mechanistic insights and one that satisfied the criteria for therapeutic application (133). The epigenetic mediation processes do not occur independently, but they are mostly interrelated networks that consist of a complex regulatory entity.

The inflammatory responses that are elicited from DCM development mainly originate from oxidative stress and cardiomyocyte apoptosis and pyroptosis reactions. Among the sources of inflammation, NLRP3 is a highly featured cytosolic inflammasome which is assembled based on metabolic derangement, mitochondrial dysfunction, ageing, and environmental influences that promote inflammation and cell death. NLRP3 inflammasome is formed due to hyperglycaemia and hyperlipidaemia, which promote pro-inflammatory cytokines by upregulating IL-1β, IL-6 and IL-18, leading towards exacerbation of DCM pathology (134).

The study of epigenetics is gaining momentum as a plausible means of theragnostic application which can be easily extended to the discovery of epi-drugs that relate to the epigenome of DCM pathogenesis. In the pursuit of drug development, there are many constraints that must be satisfied as the pharmacokinetics, pharmacodynamics, safety and tolerable limits, delivering modalities and translatability from preclinical to clinical models (135). There is evidence of bromodomain inhibitors being successful in reducing heart failure admissions in diabetic patients comorbid with acute coronary syndrome (136). Extensive further studies are required before performing clinical trials. Among the newly discovered technologies of epigenetic studies are single cell RNA sequencing, mass spectrometry -related technologies, RNA modifications, splicing and editing, whole genome bisulphite sequencing and single cell assay for transposase accessible chromatin sequencing, all of which have contributed to theragnostic research (137, 138). Additionally, a new field of epigenetics that has been pursued is epitranscriptomic alterations involving DCM pathologies (139). However, all these epigenetic modifiers hold promise in developing diagnostic and therapeutic applications for the attenuation and amelioration of complex DCM pathobiology (139).

### 3.7 Conclusion

#### 3.7.1 Implications for practice

It can be concluded that DCM manifests as severe inflammation in the heart muscles and correlates with the increases in the pro-inflammatory cytokines including HMGB1 and the immune mediators which require their inhibition or at least partial suppression of those parameters by manipulating the genetic and epigenetic factors. There are better prognostic markers of DCM other than blood glucose according to the effect size analysis carried out by this study. The best biomarker for assessing DCM is cardiac troponin followed by the triglycerides, and thirdly the proinflammatory cytokine IL-6. The best example is the mechanisms for downregulating HMGB1, which we could deduce to be a therapeutic target for alleviating inflammatory reactions and thereby the associated DCM, particularly the myocardial damage evidenced by the rodent models utilized in the present study. HMGB1 can also be considered as an alarmin in myocardial tissues which exacerbates the pathological characteristics involving DCM by inducing the release of cytokines, promoting chemotaxis of immune cells, activating immune cells, endothelial cells and fibroblasts and serving as an adjuvant in the production of autoantibodies. Therefore, the introduction of diagnostic techniques for making an early prognosis of DCM, based on the above attributes and monitoring myocardial health with routine pathological testing of HMGB1 in serum and by performing an electrocardiogram are implied as good practice for the DM population.

#### 3.7.2 Implications for research

A complete update for the year 2025 is suggested as future work. The recent technology-based diabetes and CVD research have been focussing more on artificial intelligence that incorporated machine learning and deep learning with neuronal network mapping for the discovery of predictive markers, which are supposedly more effective. These tools are expected to offer better prognosis and diagnosis that will enable customised resolution of cardiometabolic diseases. The use of imaging and smart wearables have made considerable strides towards new discoveries based on the traditional risk factors. The measuring of interstitial glucose levels in sera with sensors picking up data based on fluidics systems and monitoring the duration of sleep, blood pressure, heart rate and the heart rhythm have aided in collecting biometrics on DCM enabling the clinicians to both diagnose and offer treatment in real time. These measures are expected to reduce the morbidity and premature mortality associated with DCM pathophysiology in the clinic.

**Figure 10:**
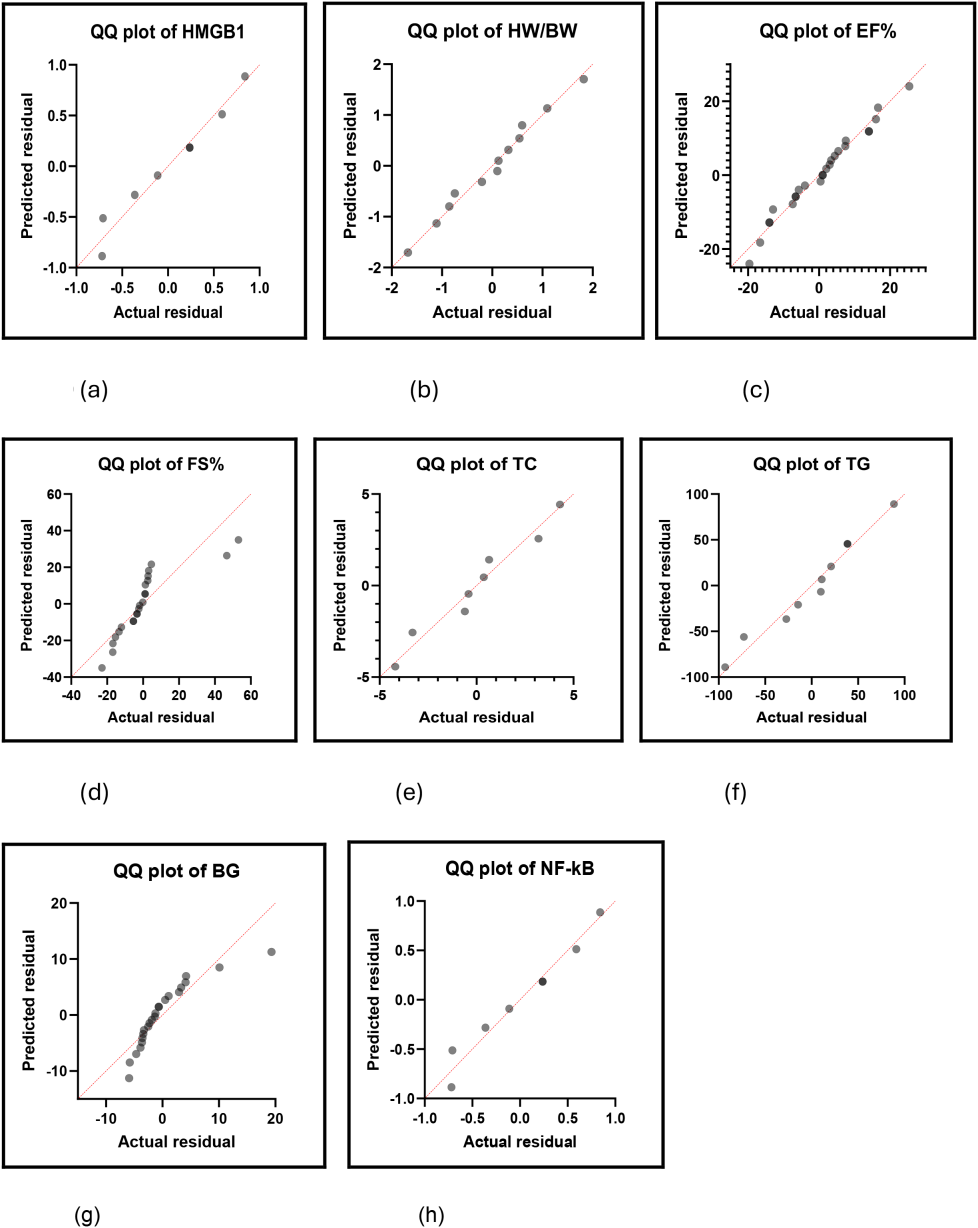
**(A–H)** The QQ plots of the biomarkers which produced probabilities less than 0.05. QQ plots are means of determining the distribution of data which occurs according to the Gaussian principles. Data points appearing on a straight line indicates a normal distribution. As evidenced by these graphs shown above 8 biomarkers conform to a normal distribution while the rest of the biomarkers do not. Legend: **(A)**-HMGB1 **(B)** HW/BW, **(C)** EF%, **(D)** FS%, **(E)** TC, **(F)** TG, **(G)** BG, **(H)** NF-kB.

Several key assumptions are made in utilising the non-parametric Mann Whitney U test in statistical data analysis, which includes, being not normally distributed data, random sampling, being continuous data which is perpetually occurring and being two groups independent of each other such as the control versus treatment groups. A QQ plot is plotted to determine normality in the data for each biomarker, and a linear line indicates conformity with normality (**Figure 10**). A perfectly symmetrical bell-shaped curve or a histogram and the mean, median and mode being equal or a p<0.05 value indicate normally distributed data.

## 4 Outcome of meta-analysis

### 4.1 Summary results

There are three types of tables,

- The pooled basic attributes or the characteristics of each eligible or included study (**Table 4**)
- Summary of findings calculated in the forest plot results (**Tables 1**-**3**)
- A risk of bias summary for each individual study [**Figures 6a, b**]

**Table 4:** Consists of the important and prominent characteristics of the twenty-nine (29) included studies in this systematic review and meta-analysis.

Study #	Year	First author	Country	Rodent model	Disease type	Control/treatment #	STZ dose	BG cutoff point	Sex	Age (wks)	Body weight (g)	Primary outcomes	Ref
1	2010	Christian Volz	GERMANY	C57BL/6	DM	10/10	50 mg/kg i.p. for 5 days	>22 mM	M	10-14	NS	HMGB1, TNF-A, IL-6, NF-KB, pERK1/2, pJNK, TLR4, LVEDD, LVEDS	(15)
2	2012	Francesca Delucchi	ITALY	Wistar rats (*Rattus norvegicus*)	T1D	25/54	60 mg/kg single i.p.	13.9 mM	M	12-14	373.9 ± 2.7	LVEDP, LVSP, HMGB1	(28)
3	2013	Beibei Luo	CHINA	Sprague Dawley rats	T2D	15/15	35 mg/kg single i.p	≥11.1 mM	NS	NS	100-120	NLRP3, TC, TG, IL-1β	(29)
4	2014	Huiling Diao	CHINA	Sprague Dawley rats	DM	10/10	50 mg/kg single i.p.	11-22 mM	M	NS	NS	HMGB1, TNF-A, IL-6, BG, NF-KB	(30)
5	2014	Wen-Ke Wang (January)	CHINA	C57BL/6j	T1D	8/8	50 mg/kg i.p. for 5 days	16 mM	NS	8-12	25-30	HMGB1, LVEDd, LVEF, FS%, BG, Fibrosis%, Collagen I, Collagen III, MMP9, TGF-1β	(31)
6	2014	Wen-Ke Wang (July)	CHINA	C57BL/6j	DM	8/8	60 mg/kg i.p. for 5 days	≥16.7 mM	M	8	NS	HMGB1, ClC3, Bax/Bcl-2, ERK1/2	(32)
7	2015	Aibin Tao	CANADA	C57BL/6	DM	3/3	50 mg/kg i.p. for 3 days	≥ 22 mM	M	6	NS	HMGB1, IL-33, Myocardial Fibrosis %, Collagen I, ESPVR, EDPVR	(16)
8	2016	Han Wu	CHINA	Mice	DM	8/8	40 mg/kg i.p. for 5 days	13.9 mM	M	NS	30.4 ± 1.5	Serum HMGB1, HMGB1/β-Actin, BG, BW, RAGE, TLR4, NF-KB, TNF-A, iNOS, NADPH,	(33)
9	2017	Kapil Suchal	INDIA	Albino Wistar rats	DM	10/12	70 mg/kg i.p. for 3 days	13.9 mM	M	10-12	150+ 200	ERK1/2, phospho (p)-ERK1/2 (p-ERK1/2), c-Jun N-terminal kinase (JNK), p-JNK, NF-κBp65, caspase-3, β-actin, Bcl-2, Bax, p38, RAGE, TXNIP, GSH, MDA, SOD, CAT	(34)
10	2017	Nawal M. Al-Rasheed	SAUDI ARABIA	Wistar rats	T1D	8/8	55 mg/kg single i.p	11 mM	M	10	160-180	NO, GSH, MDA, SOD, TNF-a, CK-MB, LDH, troponin I, H&E, CRP, HDL-C, LDL-C. VLDL-C	(35)
11	2017	Wei-Fang Li	CHINA	Sprague- Dawley rats	DM	8/8	60 mg/kg single i.p	NS	M	6-8	220-250	LVIDs, LVIDd, IVSd, FS%, GP, SOD, MDA, BW, HR	(36)
12	2017	Weng-Ke Wang	CHINA	C57/BL6J	DM	8/8	50 mg/kg i.p. for 5 days	≥16.7 mM	M	6-8	25-30	PBG, HR, SBP, BW, LVIDs, EF%, TNF-a, IL-6, MAPK, P38, P-JNK, Bcl-2, HMGB1	(37)
13	2018	Hong-Wei Wang	CHINA	Wistar rats	DM	6-8/6-8	50 mg/kg Single i.p	> 16.67 mM	M	NS	200± 220	cTn-I, CK-MB, TGF-β1, α-SMA, p-Smad3, Bcl-2, cleaved-caspase-3, collagen I and III	(38)
14	2020	Yi Wang	CHINA	C57BL/6	T1D	6/7	100 mg/kg single i.p	>12 mM	M	8	18-22, 200-220	NF-KB, MAPK, TLR4, MyD88, CK-MB, ANP, TGFβ1, MMP2, MMP9, AGEs, FBG, BW, IVSD, IVSS, TNF-a, IL-6	(39)
15	2020	Yuwei Zhang	CHINA	C57BL/6	T1D, T2D	14/14	50 mg/kg i.p. (days NS)	16.7 mM	M	6-8	NS	TLR4, ANP, TNF-a, IL-6, ICAM-1, VCAM-1, EF%, FS%, LVSV, LVDV, Type I and III collagen	(40)
16	2021	Hui Shi	CHINA	Sprague Dawley rats	DM	15/15	35 mg/kg i.p. for 3 days	NS	M	8-9	200 ± 20	TLR4, MyD88, NF-kB p65, TNF-a, IL-1b, IL-6, EF%, FS%, Caspase-3	(41)
17	2023	Eman A. E. Farrag	EGYPT	Sprague–Dawley rats	T2D	6/12	27.5 mg/kg single i.p.	>16.7 mM	M	NS	160 ± 20	FBG, INS, CK-MB, LDH, HW/BW, H&E, HOMA-IR, AGE, RAGE, HMGB1, IL-1β, MDA, GSH	(42)
18	2023	Yingying Hu	CHINA	Sprague- Dawley rats	T2D	8/8	30 mg/kg i.p. for 3 days	NS	NS	NS	180-220	NLRP3, NF-KB, TLR4, HMGB1, ASC, GSDMD, IL-1β, IL-18	(43)
19	2024	Marwa M. M. Refaie	EGYPT	Wistar albino rats	DM	10/10	45 mg/kg single i.p.	NS	M	4	90 ± 100	FBG, HbA1C, TAC, GSH, MDA, ATII, Caspase1, HW, HW/BW, NLRP3, TNF-A, IL-1β, NF-KB	(44)
20	2024	Feng Hu	CHINA	C57BL/6N	T2D	6/6	100 mg/kg single i.p.	11.1 mM	M	8	NS	LVEF, LVSF, pERK 1/2/ERK 1/2	(45)
21	2024	Jinxiu Zhu	CHINA	C57BL/6J	DM	10/10	50 mg/kg i.p. for 5 days	> 16.7 mM	M	4	NS	LVEF%, FS%, LVIDd, LVIDs, FBG, BW, HW, HW/BW, TLR4, NF-kB	(46)
22	2024	Liping Zhu	CHINA	Albino Wistar rats	T2D	6/6	60 mg/kg single i.p.	11.11 mM	M	NS	180-200	IL-1β, IL-6, TNF-α, MDA, TGF-β1, BG, Caspase 3, TC, TG, HDL, LDL, CK-MB, CTPN	(47)
23	2024	Qihui Huang	CHINA	Sprague Dawley rats	T2D	6/6	30 mg/kg single i.p.	≥ 11.1 mM	M	10	250-300	CK-MB, LDH, EF%, FS%, LVSV, MDA	(48)
24	2024	Vipin Kumar Verma	INDIA	Albino Wistar rats	T1D	8/8	40 mg/kg single i.p.	22.2 mM	M	10-12	150-200	GSH, MDA, CK-MB, LDH, TNF-A, IL-6, Caspase 3, HMGB1.	(49)
25	2024	Ze-Yu Zhou	CHINA	C57BL/6	T2D	6/6	80 mg/kg single i.p.	NS	M	8	NS	EF%, FS%, LVIDd, LVIDs, Collagen I, Collagen III	(50)
26	2024	Xuan Zhou	CHINA	C57BL/ 6JNifdc	T2D	10/10	30 mg/kg i.p. for 7 days	≥ 11.1 mM	M	6	NS	CK-MB, LDH, TC, TG, IL-1β, IL-6, EF%, FS%, LVIDd, LVIDs	(51)
27	2025	Huiping Yang	CHINA	C57BL/6J	T2D	5/5	40 mg/kg i.p. for 3 days	≥ 11.1 mM	M	3	NS	GSH, MDA, LDH, LVEF%, FS%, LVIDd, LVIDs	(52)
28a	2025	Weipin Niu	CHINA	Piezo1 dtT/dtT	T1D	10/10	55 mg/kg i.p. for 5 days	> 16.7 mM	M	8	NS	EF%, FS%, LVIDd, LVIDs, HW, HW/TL	(53)
28b	2025	Weipin Niu	CHINA	Piezo1 ΔMyh6	T2D	10/10	35 mg/kg i.p. for 3 days	> 16.7 mM	M	3	NS	EF%, FS%, LVIDd, LVIDs, HW, HW/TL	(53)
29	2025	Lixia Zhang	CHINA	C57BL/6J	DM	10/10	60 mg/kg i.p. for 5 days	≥ 11.1 mM	M	6-8	NS	EF%, FS%, Fibrosis%, NLRP3, IL-1β	(54)

The year of publication, name of the first author, the country in which this study was conducted, the rodent species; rat or mouse, the disease subtype of *Diabetes mellitus* (DM), the number of animals in this study utilized as the control group and the experimental or the DCM group, the dose, route of administration and the number of consecutive days the STZ was injected, the blood sugar cutoff point which confirms that diabetes was induced, the gender, age and bodyweight of the rodent species, and the primary outcomes reported by each included study are described in this table (Table 4). The included studies were selected if they had used streptozotocin (STZ) as the drug which was injected to the mice and rats to develop diabetic cardiomyopathy in addition to a high-fat diet. Various included studies had used different dosages of streptozotocin to induce DCM in the rodents. The cutoff point for blood glucose, all given in millimolar units, confirmed the onset of DCM. The primary outcomes included all the tests that were performed by the authors. Both the rat and mouse species were included in this study in order to increase heterogeneity and to have a high sample size for each biomarker. The age of the rodents oscillated between 3 to 14 weeks. Where the diabetes subtype was not indicated, it was referred to as DM, for *Diabetes mellitus*. Abbreviations: M-male, NS-not specified, i.p.-intraperitoneal, mM – millimolar.

### 4.2 Narrative summary

#### 4.2.1 Description of the type of intervention in the included studies and how they were implemented

The type of intervention used is HMGB1, the ubiquitous and highly evolutionarily conserved nuclear protein, which was tested in experimentally induced DCM to elicit a cure phenotype from this rodent model. HMGB1 is passively secreted by injured and necrotic cells while it is actively secreted by activated immunocompetent cells such as the dendritic cells and macrophages. Inside the nucleus it controls transcription regulation, DNA repair and regeneration processes. The extracellular HMGB1 acts as a DAMP molecule which produces several redox forms that bind with multiple receptor types thereby inducing a cascade of inflammatory responses, particularly in diabetes. DCM comprises of a debilitating disease complex of the heart and major blood vessels, which are when injured, induce inflammation in the cardiac tissue and also promotes adaptive immunity. The experimental inhibition of HMGB1 in myocardial ischemia or reperfusion injury, myocarditis, and cardiomyopathies have been able to attenuate disease by reducing inflammation and therefore, is protective towards the DCM phenotype. As such, there are instances where HMGB1 has played dual roles in DCM, being both beneficial and detrimental.

#### 4.2.2 A description of the primary and secondary outcomes in the included studies

The primary outcomes consisted of the biomarkers of DCM for which a forest plot each was constructed. The biomarkers, some of which can act as risk factors were grouped into 8 models of DCM (AGEs, cardiometabolic, glycaemic, lipid, oxidative stress, inflammatory, the molecules of signalling pathways and fibrosis) in which a total of 37 biomarkers were evaluated. The biomarkers which had the effect sizes that were significant comprised of 28 biomarkers which are in descending order of the effect size given as: CTPN (SMD 37.93, *p* = 0.03), TG (SMD 16.60, *p* = 0.0007), BG (SMD 7.54, *p* = 0.00001), TC (SMD 7.38, *p* = 0.009), HDL (SMD -5.84, p= 0.0002), LDH (SMD 5.66, *p* = 0.0005), IL-6 (SMD 5.49, *p* = 0.00001), AGEs (SMD 5.43, *p* = 0.004), MDA (SMD 4.78, *p* = 0.00001), HW (SMD 4.73, *p* = 0.05), GSH (SMD -4.60, *p* = 0.009), IL-1β (SMD 4.44, *p* = 0.003), EF% (SMD -4.13, *p* = 0.00001), CC3 (SMD 3.91, *p* = 0.002), NLRP3 (SMD 3.59, p= 0.03), CK-MB (SMD 3.28, *p* = 0.004), FB% (SMD 3.25, *p* = 0.01), NF-KB (SMD 3.13, *p* = 0.0001), HMGB1 (SMD 3.00, *p* = 0.0001), FS% (SMD -2.91, p= 0.00001), COL III (SMD 2.43, *p* = 0.01), TLR4 (SMD 2.38, *p* = 0.009), HW/BW (SMD 2.31, *p* = 0.007), pJNK (SMD 1.87, *p* = 0.0001), BW (SMD -1.74, p= 0.05), LVIDs (SMD 1.64, *p* = 0.04), TGF-1β (SMD 1.50, *p* = 0.05), and TNF-A (SMD 1.11, *p* = 0.03). These 28 primary outcomes have indicated cardiac tissue damage and the disruption of the metabolic responses in the heart and major blood vessels in this rodent model.

In the non-parametric Mann Whitney U test, eight biomarkers showed a significant difference, in the DCM-induced animals displaying a significant increase in: TG (SMD 16.60, *p* = 0.0007), BG (SMD 7.54, *p* = 0.00001), TC (SMD 7.38, *p* = 0.009), EF% (SMD -4.13, *p* = 0.00001), NF-KB (SMD 3.13, *p* = 0.0001), HMGB1 (SMD 3.00, *p* = 0.0001), FS% (SMD -2.91, *p* = 0.00001) and HW/BW ratio (SMD 2.31, *p* = 0.007).

In the correlation analysis, there were only four biomarkers: HR (r= - 0.9998, R^2^ = 0.9997, *p* = 0.0113), TLR4 (r= - 0.9617, R^2^ = 0.9248, *p* = 0.0383), EF% (r= - 0.9580, R^2^ = 0.9178, *p* = 0.0420) and HW/BW (r= -0.9551, R^2^ = 0.9122, *p* = 0.0449) that showed a significant negative correlation with HMGB1 when it is treated as the independent variable.

All the pro-inflammatory cytokines tested (HMGB1, TNF-A, IL-6 and IL-1β) indicated increasing inflammation and damage to the structure and function of the heart, which is established DCM. Of the signalling molecular mechanisms related to high inflammation, NF-kB, NLRP3, TGF-β, TLR4, cleaved caspase 3 and phosphorylated JNK showed significant increases in the forest plots, which also reflect the establishment of DCM in this rodent model and the need for an anti-inflammatory approach to attenuate DCM in the preclinical subjects.

#### 4.2.3 A review of findings for the secondary outcomes

In the non-parametric Mann Whitney U test, five biomarkers showed a marked difference with the DCM-induced animals displaying a significant increase or decrease reflecting the pathophysiological status of the rodent model used. When the Mann Whitney U statistic is considered the significantly highest were those which recorded a zero, that included high titres of HMGB1, total cholesterol, NF-kB, and the collagens I and III (**Table 1**) in the DCM group. HMGB1 which is the biomarker that was evaluated as a therapeutic target of DCM and NF-kB, a nuclear transcription factor, both of which cause inflammation, contribute to myocardial damage. Out of the cardiometabolic biomarkers which had significantly high or low titres in the DCM group, the EF%, FS%, total cholesterol, triglycerides the HW/BW ratio, and the hyperglycaemic biomarker, the blood glucose level, also indicated high risk and susceptibility to cardiac dysfunction by causing significant impairment of the functional parameters of CVD health. There was significant correlation between the decreasing ejection fraction percentage with HMGB1 as well as a significant positive correlation with the immune biomarker, TLR4 which has HMGB1 as one of its corresponding ligands highlighting their importance as biomarkers of diabetes-induced cardiomyopathy.

#### 4.2.4 Adverse outcomes or potential threats, harm or losses

The adverse outcomes are not having sufficient numerical data or being limited to one or two included studies or the outcomes being reported in variable units such as nanomolar, micromolar or millimolar that are difficult to be converted into a single unit, due to the generation of large standard deviations or the SEM. Data is lost when the missing data are not provided by the authors of the articles. When the data must be extracted from graphs or images, they need to be removed due to copyright issues when the publishers do not respond to the requests being made.

#### 4.2.5 Associated financial costs in implementing the study design

Since this study was mostly paper-based, and computer-based, the cost of implementing the study design remained low. The access to free online services *via* the VU library did not require large funds.

#### 4.2.6 Possible important contextual details pertaining to the study design and/or analysis

The PICOS study design:

**Table:** 

POPULATION	DCM-induced rodents and age and sex-matched healthy control
INTERVENTION	HMGB1 nuclear protein
COMPARATORS	DCM VS HC
OUTCOME	High risk or low risk of DCM
STUDY DESIGN	Case-control baseline studies in a meta-analysis.

This study does not have any perceivable adverse contextual details regarding the study design as this is a systematic review and a meta-analysis based on the Cochrane Organisation template for systematic review and meta-analyses.

#### 4.2.7 Perceived strengths, weaknesses and contributions made on the studies

The overall results obtained from the analyses confirm that HMGB1 exacerbates DCM in the diabetic rodent group (HMGB1: SMD 3.00, *p* = 0.00001 and Mann Whitney U statistic = zero at *p* = 0.0286). The strength of this study is the ability to utilise a considerably high volume of data from published research papers over a time period decided by the authors. The more participants or animal models are in use, the more reliable the data would be and the outcome of this study will be validated as well. This method of research is more useful because the data come from several parts of the world, and hence be more heterogeneous. The weakness is that some of the epigenetic risk factors will not be more popular, therefore, the individual study authors tend to mostly quantify the traditional risk factors which are more common such as the glycaemic and lipid markers but not many vascular biomarkers. Another drawback is that when the study authors are contacted over missing data *via* their correspondence emails, some of them tend to ignore the requests and do not reply. However, when conducting RCTs, in most of such trials, no healthy control participants are enrolled. Similarly, when studies are based upon data taken from repositories, they may contain obsolete or invalid data. As this is mentioned before, this research method is highly cost-effective and do not require a large grant or sponsors but requires only a few consumables and travelling expenses such as for conferences.

### 4.3 Report on practical significance

The biomarker analysis that is calculated from the individual forest plots include 37 grouped into 8 DM-induced vasculopathy models. 29 biomarkers showed a significant difference (*p* < 0.05) between the DCM and the HC groups in the forest plots. Their effect sizes or the SMDs are tabulated in **Table 2**. They form a comprehensive list of DCM-related biomarkers which have potential for formulating anti-inflammatory therapies for mitigating the severe inflammation that is associated with the manifestation of DCM even in the rodent models of mouse and rat. Most of these biomarkers consist of large effect sizes which are greater than 0.8 according to the Cohen’s D index. Out of the 29 biomarkers, those which exhibited the ten highest SMDs in the rodents included CTPN, TG, BG, TC, LDH, IL-6, AGEs, MDA, HW, GSH. In identifying the biomarkers which could also serve as diagnostic tools for monitoring the serum levels could include Cardiac Troponin, LDH, CK-MB, glutathione, IL-1β, TNF-alpha as alternatives to blood glucose which is the strongest biomarker which acts as the best diagnostic tool up to now. Further, in an electrocardiographic analysis, LVDV, LVSV, EF% and FS% may offer reliable results. DCM is a debilitating disease which produces worse outcomes in diabetic persons, and the results obtained from rodent models serve to confirm some of these already established DCM evaluation criteria. Therefore, we offer this list of biomarkers for serious consideration by researchers in assessing these markers as drug targets for attenuating the pathophysiology of DCM serving as preliminary metabolic checkpoints for making an early diagnosis of clinical DCM. In suggesting the changes that are needed by way of lifestyle and epigenetic factors in the patients diagnosed with DCM, physical exercise and a customised meal plan are suggested alongside treatment for insulin resistance. Additionally, some of these markers could be adopted for formulating a suitable strategy not only for making an early diagnosis of DCM, but carrying out regular screening, initiating treatment and monitoring of therapeutic progress. The burden of hospitalisation costs for an individual with hypertrophic cardiomyopathy in the USA was estimated to be between US dollars 10k to 17k. The median annual costs per patient for CVD, coronary artery disease, heart failure, and stroke were, 112%, 107%, 59%, and 322% respectively higher compared with those for T2D patients without CVD as reported by a Canadian research team. On average, treating patients with CVD and T2D resulted in a cost increase ranging from $3418 to $9705 compared with treating patients with T2D alone. As for suggestions on novel research ideas which promote attenuation of inflammation in DCM, the researchers have focussed on novel drug targets such as TNF-alpha (an inflammatory biomarker), HOMA-c peptide (a biomarker of insulin resistance), FOXO1 (biomarker for cardiac fibrosis). The present study has reported a significant negative correlation between TNF-alpha and HMGB1, the evolutionarily conserved nuclear protein which has been experimented with for many years and may lead towards pertinent information. DCM may begin as initial endothelial dysfunction which has been suggested as a useful area to concentrate in DCM research, alongside investigating means to reduce lipid stores and lipo-toxicity, particularly in cardiac tissue which is also supported by data from this study. More research is needed in the pharmacotherapy of the SGLT2- inhibitors and the GLP-1 receptor agonists that are used for treating T2D at present, due to the fact that they improve myocardial contractile function in clinical DCM. As such, the present pilot study offers many useful insights into the future pathophysiological and pharmacotherapeutic research on clinical DCM which is considered as fulfilling a gap that exists in DCM research.

## Data Availability

The original contributions presented in the study are included in the article/**Supplementary Material**. Further inquiries can be directed to the corresponding author.

## Glossary

ACE: Angiotensin converting enzyme
AcH: Acetylcholine
AGE: Advanced glycation end products
AI%: Augmentation index percent
AIHW: Australian institute of health and welfare
ACR: Albumin creatinine ratio
ALT: Alanine amino transferase
α7nAchR: Alpha 7 nicotinic acetylcholine receptor
AMP: Adenosine mono phosphate
AMPK: Adenosine mono phosphate kinase
Ang-1: Angiopoietin-one
Ang-2: Angiopoietin-two
ANP: Atrial natriuretic peptide
APO-A: Apolipoprotein-A
APO-B: Apolipoprotein-B
Arf6: ADP-ribosylation factor -6
AST: Aspartate amino transferase
AT2R: Angiotensin receptor two
BBB: Blood brain barrier
BMI: Body mass index
BNP: Brain natriuretic peptide
BUN: Blood urea nitrogen
CAD: coronary artery disease
cm: Centimetre
CAM: Cell adhesion molecules
C5aR2: Complement receptor C5aR2
CBA: Control before and after
CCTA: Coronary computed tomography angiography
CK-MB: Creatine kinase in muscle and brain
COX: Cyclooxygenase
CPG-ODN: Cpg-oligodeoxynucleotides
CTP: Cardiac troponin
CXCR4: CXC-chemokine receptor four
CXCL12: CXC-motif- chemokine ligand 12
CVD: cardiovascular disease
DAMP: Damage associated molecular pattern
DBP: Diastolic blood pressure
DCM: Diabetic cardiomyopathy
DM: Diabetes mellitus
DNA: Deoxyribonucleic acid
DOI: Digital object identifier
DPPI-4: Dipeptidyl peptidase inhibitor 4
ECG/EKG: Electrocardiogram
ED: Endothelial dysregulation/ dysfunction/ deregulation
EGFR: Estimated glomerular filtrate
EF%: Ejection fraction percentage
eNOS: Endothelial nitric oxide synthase
ER: Endoplasmic retic
ERK: Extracellular signal regulate
ERS: Endoplasmic reticulum s
ESAM: Endothelial-cell selective adhesion molecule
ET-1: Endothelin-1
ETC: Electron transport chain
FBG: Fasting blood glucose
FMD: Flow-mediated dilatation perc
FMD%: Flow-mediated dilatation perc
FS%: Fractional shortening percentage
GGT: Gamma-glutamyl transferase
GLP-1: Glucagon-like peptide-1
GPCR: G protein coupled receptor
GRP78: Glucose regulated protein 78
GSH: Glutathione
HbA1c: Glycated haemoglobin
HC: Healthy control
HDL-C: High density lipoprotein cholesterol
HIV: Human immunodeficiency virus
HMG: High mobility group molecular family
HMGB1: High mobility group box-1
HO-1: Haem oxygenase-one
HOMA-IR: Homeostatic model assessment of Insulin resistance
HR: Heart rate
HT: Height
HW: Heart weight
ICAM: Intercellular cell adhesion molecule
IDF: International diabetes federation
INF-JNK: Interferon JNK
INS: Insulin
IR: Insulin resistance
JNK: Janus kinase
KDA: Kilo Dalton
LDH: Lactate dehydrogenase
LDL-C: Low-density lipoprotein cholesterol
LPS: Lipopolysaccharide
LVIDd: Left ventricular internal diameter at end-diastole
LVIDs: Left ventricular internal diameter at end-systole
LVDV: Left ventricular diastolic volume
LVSV: Left ventricular systolic volume
MAPK: Mitogen-activated protein kinas
MASLD: Metabolic -dysfunction associated- steatosis liver disease
MCP-1: Monocyte chemo attractant protein-1
MDA: Malondialdehyde
MD2: Myeloid differentiation protein-2
MFN2: Mitofusin-2
MIR: Micro RNA
MI: Myocardial infarction
mm: millimetre
MyD88: Myeloid differentiation factor 88
NADPH: Nicotinamide adenine dinucleotide phosphate
NES: non-exercised -based fitness assessment
NF-KB: Nuclear factor kappa beta
NLRP3: NLR family pyrin binding domain 3
NOD: Nucleotide-binding oligomerization
NO: Nitrous oxide
NOX: NADPH oxidase
Nrf2: Nuclear factor erythroid 2-relate
NT-Pro BNP: Natriuretic peptide test
PAI-1: Plasminogen activator inhibitor -one
PAD: Peripheral artery disease
PAMP: Pathogen-associated molecular pattern
PARP-1: Poly (ADP ribose) polymerase-one
PTM: post-translational modifications
PRISMA: Preferred reporting items for systematic reviews and meta-analyses
PWV: Pulse wave velocity
RAAs: Renin Angiotensin aldosterone system
RAGE: Receptor for AGEs
RNA: Ribonucleic acid
RNS: Reactive nitrogen species
ROB: Risk of bias
ROC: Receiver operator curve
ROBINS-1: Risk of bias of non-randomized studies
ROS: Reactive oxygen species
RR: Risk ratio
SARS-Cov-2: severe acute respiratory syndrome-corona virus-2
SBP: Systolic blood pressure
SCr: Serum creatinine
SD: Standard deviation
SEM: Standard error of the mean
SMD: Standardized mean difference
SGLT2: Sodium glucose co-transporter 2 inhibitors
SIRT-1: Sirtuin -1
SMAD-2: Mothers against decapentaplegic homolog 2
SO: Super oxide
SOCE: Store-operated calcium entry
SOD: Superoxide dismutase
STAT-1: Signal transducer and activator of transcription-1
TBARS: Thio barbituric acid
TC: Total cholesterol
T1D/T1DM: Type one diabetes
T2D/T2DM: Type two diabetes
TG: Triglyceride
TGF-β1: Transforming growth factor -beta one
Tie-2: Tyrosine protein kinase
TNF-A: Tumour necrosis factor-alpha
TLR: Toll-like receptor
TLR4: Toll-like receptor 4
TIR: Time in range
TP53: Tumour protein 53
TREM-1: Triggering receptor expressed on myeloid cell group 1
TRPV4: Transient receptor potential vanilloid family member 4
US/USA: United States of America
VAP-1: Vascular adhesion protein-one
VCAM-1: Vascular cell adhesion molecule – one
VE-Cadherin: Vascular endothelial cadherin
VEGF: Vascular endothelial growth factor
VLDL-c: Very low-density lipoprotein cholesterol
vWF: Von Willebrand factor
WHO: World Health Organization
XO: Xanthine oxidase
