# Supplementary material for: Investigating the biomarkers of diabetic-cardiomyopathy with the high mobility group box-1 as a potential anti-inflammatory therapeutic target: Systematic Review and meta-analysis
Source: Front Endocrinol (Lausanne). 2026 Jan 14;16:1714219. doi: 10.3389/fendo.2025.1714219 (PMC12846985; doi:10.3389/fendo.2025.1714219)
Supplement: Supplementary file 1 [file DataSheet1.pdf]

SUPPLEMENTARY SECTION 1: FOREST PLOTS OF THE BIOMARKERS.

Model 1 – Advanced Glycation Ends Products (AGEs)

Advanced Glycation End Products (AGEs)

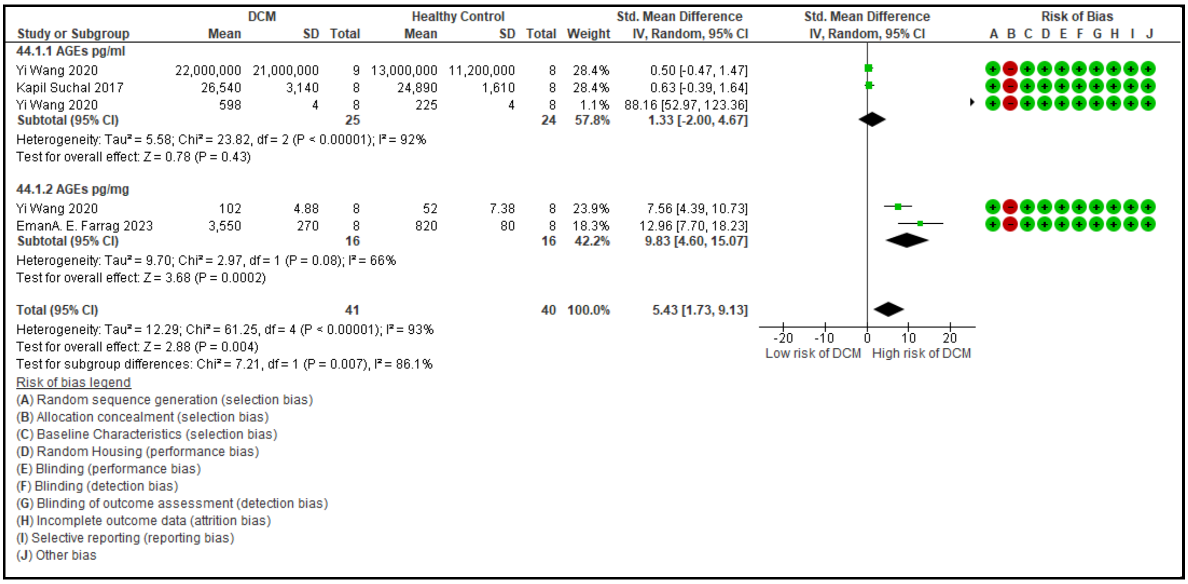

Figure S1-1: Forest plot of AGEs for the calculation of the effect size or SMD.

High Mobility Group Box -1 (HMGB-1)

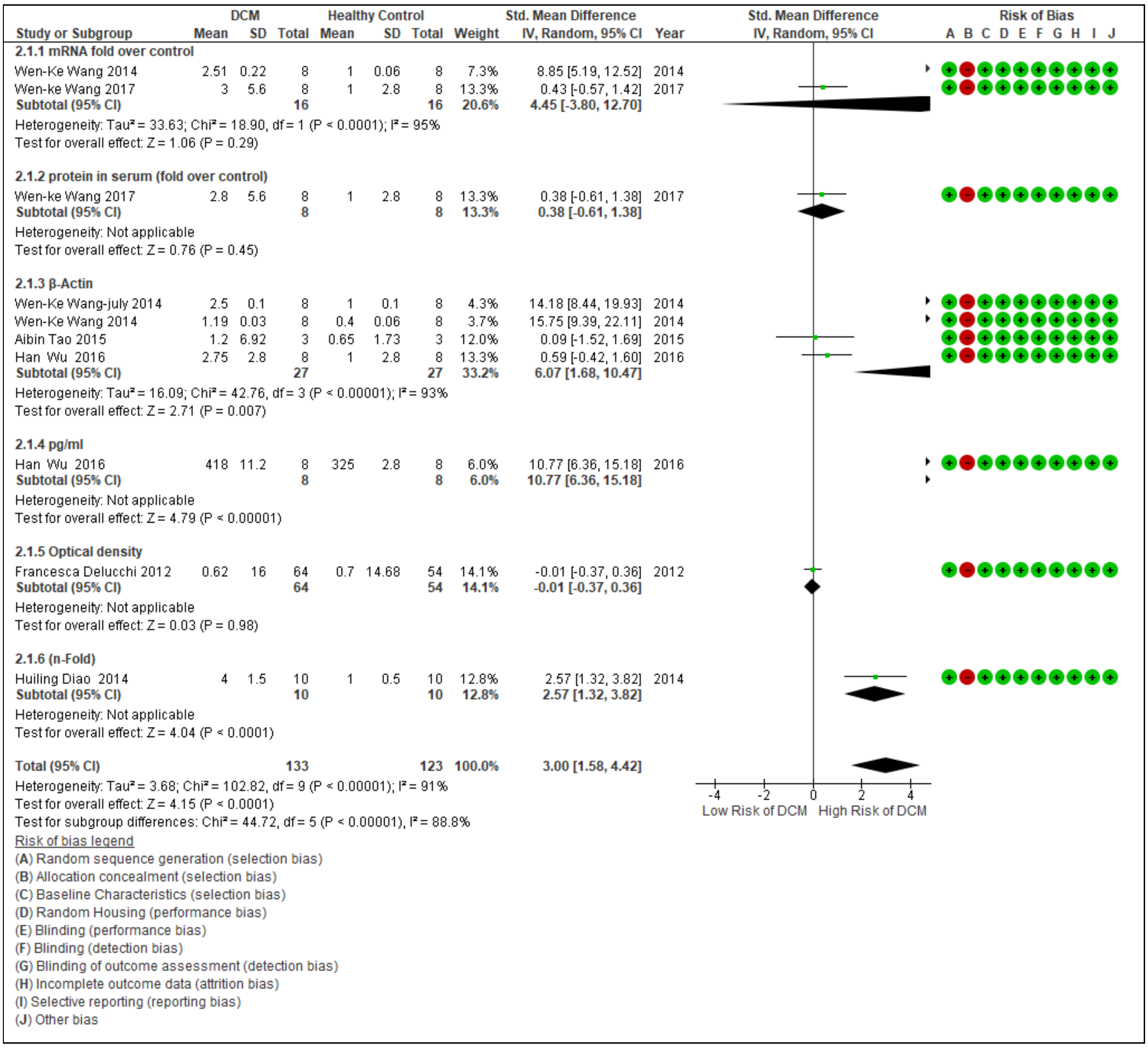

Figure S1-2: Forest plot of HMGB1 for the calculation of the effect size or SMD.

Model 2 - Cardiometabolic Biomarkers

Heart Rate (HR)

Study or Subgroup	DCM Mean	DCM SD	DCM Total	Healthy Control Mean	Healthy Control SD	Healthy Control Total	Weight	Std. Mean Difference IV, Random, 95% CI
Qihui Huang 2024	378	0.5	6	380	0.7	6	44.6%	-3.04 [-4.90, -1.17]
EmanA. E. Farrag 2023	224	19.85	6	102	6.49	6	43.4%	7.63 [3.74, 11.52]
Wei-Fang Li 2017	380	2.8	8	180	1.4	8	12.0%	85.42 [51.32, 119.53]
**Total (95% CI)**			**20**			**20**	**100.0%**	**12.20 [-1.59, 26.00]**

Heterogeneity: Tau² = 110.18; Chi² = 48.09, df = 2 (P < 0.00001); I² = 96%
Test for overall effect: Z = 1.73 (P = 0.08)

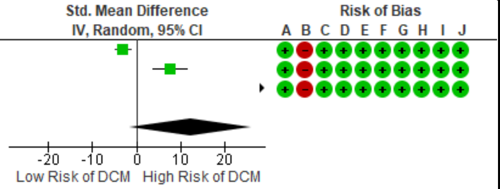

Risk of bias legend
(A) Random sequence generation (selection bias)
(B) Allocation concealment (selection bias)
(C) Baseline Characteristics (selection bias)
(D) Random Housing (performance bias)
(E) Blinding (performance bias)
(F) Blinding (detection bias)
(G) Blinding of outcome assessment (detection bias)
(H) Incomplete outcome data (attrition bias)
(I) Selective reporting (reporting bias)
(J) Other bias

Figure S1-3: Forest plot of HR for the calculation of the effect size or SMD.

Heart Weight (HW)

Study or Subgroup	DCM Mean	DCM SD	DCM Total	Healthy Control Mean	Healthy Control SD	Healthy Control Total	Weight	Std. Mean Difference IV, Random, 95% CI
Yi Wang 2020	117.8	3.75	7	125.1	8	6	26.9%	-1.12 [-2.33, 0.09]
Marwa M. M. Refaie 2024	443.1	35.9	10	306	25.2	10	26.4%	4.23 [2.53, 5.94]
Jinxiu Zhu 2024	121	2.46	10	103	1.75	10	24.7%	8.08 [5.15, 11.00]
EmanA. E. Farrag 2023	1,265	69	6	692	51	6	22.0%	8.72 [4.31, 13.12]
**Total (95% CI)**			**33**			**32**	**100.0%**	**4.73 [0.09, 9.37]**

Heterogeneity: Tau² = 20.45; Chi² = 57.44, df = 3 (P < 0.00001); I² = 95%
Test for overall effect: Z = 2.00 (P = 0.05)

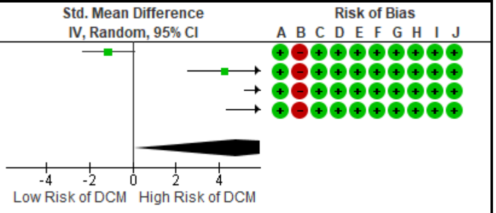

Risk of bias legend
(A) Random sequence generation (selection bias)
(B) Allocation concealment (selection bias)
(C) Baseline Characteristics (selection bias)
(D) Random Housing (performance bias)
(E) Blinding (performance bias)
(F) Blinding (detection bias)
(G) Blinding of outcome assessment (detection bias)
(H) Incomplete outcome data (attrition bias)
(I) Selective reporting (reporting bias)
(J) Other bias

Figure S1-4: Forest plot of HW for the calculation of the effect size or SMD.

Heart Weight / Body Weight Ratio (HW/BW)

Study or Subgroup	DCM Mean	DCM SD	DCM Total	Healthy Control Mean	Healthy Control SD	Healthy Control Total	Weight	Std. Mean Difference IV, Random, 95% CI
Ze-Yu Zhou 2024	6	2.23	6	5.5	4.46	6	19.3%	0.13 [-1.00, 1.26]
Hong-Wei Wang 2018	5.2	12.2	6	2	4.88	6	19.2%	0.32 [-0.82, 1.46]
H. Christian Volz 2010	6.5	6.32	10	4	3.16	10	19.9%	0.48 [-0.41, 1.37]
Yi Wang 2020	4.66	0.081	7	4.218	0.159	6	16.7%	3.35 [1.46, 5.24]
Jinxiu Zhu 2024	4.55	0.19	10	3.78	0.09	10	16.5%	4.96 [3.03, 6.89]
EmanA. E. Farrag 2023	5.53	0.3	6	2.57	0.31	6	8.4%	8.96 [4.44, 13.47]
**Total (95% CI)**			**45**			**44**	**100.0%**	**2.31 [0.63, 3.99]**

Heterogeneity: Tau² = 3.48; Chi² = 38.96, df = 5 (P < 0.00001); I² = 87%
Test for overall effect: Z = 2.69 (P = 0.007)

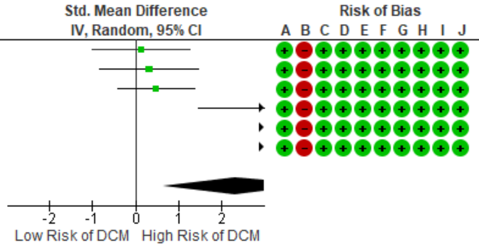

Risk of bias legend
(A) Random sequence generation (selection bias)
(B) Allocation concealment (selection bias)
(C) Baseline Characteristics (selection bias)
(D) Random Housing (performance bias)
(E) Blinding (performance bias)
(F) Blinding (detection bias)
(G) Blinding of outcome assessment (detection bias)
(H) Incomplete outcome data (attrition bias)
(I) Selective reporting (reporting bias)
(J) Other bias

Figure S1-5: Forest plot of HW/BW for the calculation of the effect size or SMD.

Ejection Fraction Percentage (EF%)

Study or Subgroup	DCM Mean	DCM SD	DCM Total	Healthy Control Mean	Healthy Control SD	Healthy Control Total	Weight	Std. Mean Difference IV, Random, 95% CI
Wei-Fang Li 2017	70	1	8	100	0.5	8	0.9%	-35.88 [-50.23, -21.53]
Jinxiu Zhu 2024	40	1.58	10	68	1.58	10	3.9%	-16.97 [-22.91, -11.04]
Yingying Hu 2023	40	2	4	79	2	4	1.3%	-16.96 [-28.70, -5.21]
Weipin Niu 2025	55	2.44	6	75	2.44	6	6.3%	-7.57 [-11.43, -3.70]
Ze-Yu Zhou 2024	41	1.22	6	55	2.44	6	6.9%	-6.70 [-10.16, -3.24]
Qihui Huang 2024	68	2	6	78	1	6	7.6%	-5.84 [-8.90, -2.77]
Yuwei Zhang 2020	48.26	5.21	14	67.18	4.73	14	10.6%	-3.69 [-4.97, -2.41]
Huiping Yang 2025	50	9	5	82	7	5	8.8%	-3.59 [-5.95, -1.22]
Wen-Ke Wang 2014	55	11	8	77.6	7.1	8	10.5%	-2.31 [-3.65, -0.96]
Yi Wang 2020	70.57	6.07	7	82.12	4.88	6	10.4%	-1.93 [-3.34, -0.52]
Hui Shi 2021	40	15.2	15	68	19	15	11.2%	-1.58 [-2.42, -0.75]
Feng Hu 2024	56	1.11	5	58	2.23	5	10.5%	-1.03 [-2.39, 0.34]
Lixia Zhang 2025	68	15.8	10	80	9.48	10	11.1%	-0.88 [-1.81, 0.05]
**Total (95% CI)**			**104**			**103**	**100.0%**	**-4.13 [-5.56, -2.69]**

Heterogeneity: Tau² = 4.61; Chi² = 89.69, df = 12 (P < 0.00001); I² = 87%
Test for overall effect: Z = 5.65 (P < 0.00001)

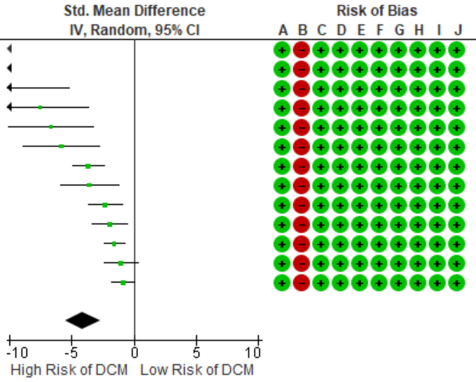

Risk of bias legend
(A) Random sequence generation (selection bias)
(B) Allocation concealment (selection bias)
(C) Baseline Characteristics (selection bias)
(D) Random Housing (performance bias)
(E) Blinding (performance bias)
(F) Blinding (detection bias)
(G) Blinding of outcome assessment (detection bias)
(H) Incomplete outcome data (attrition bias)
(I) Selective reporting (reporting bias)
(J) Other bias

Figure S1-6: Forest plot of EF% for the calculation of the effect size or SMD.

Fractional Shortening Percentage (FS%)

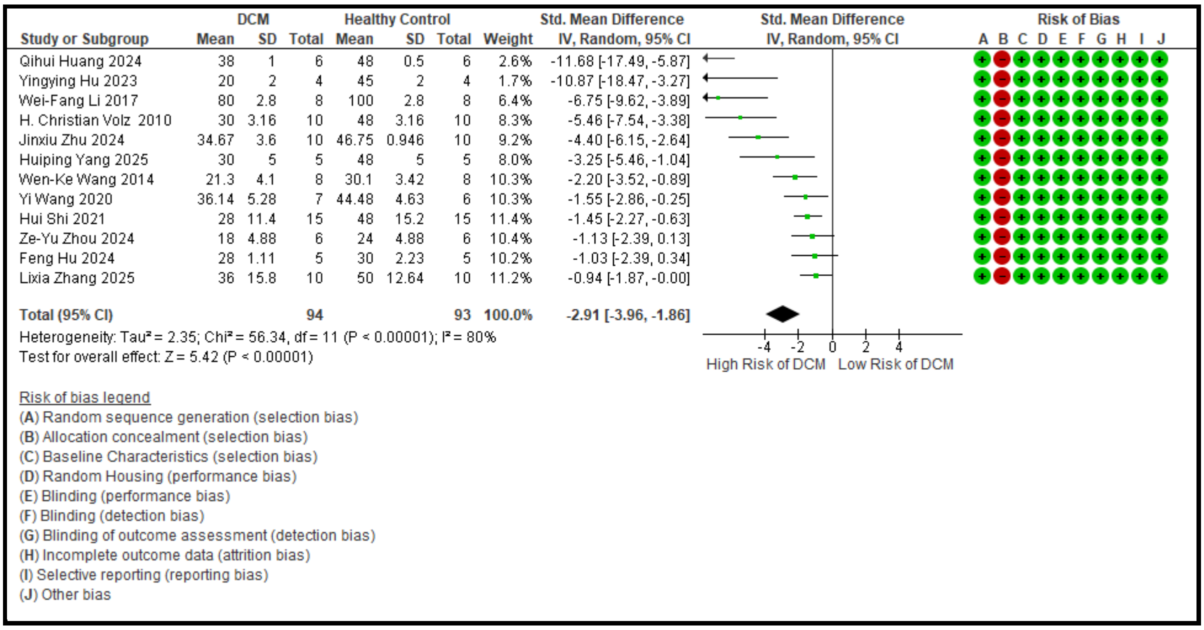

Figure S1-7: Forest plot of FS% for the calculation of the effect size or SMD.

Left Ventricular Internal Diameter at Diastole (LVIDd)

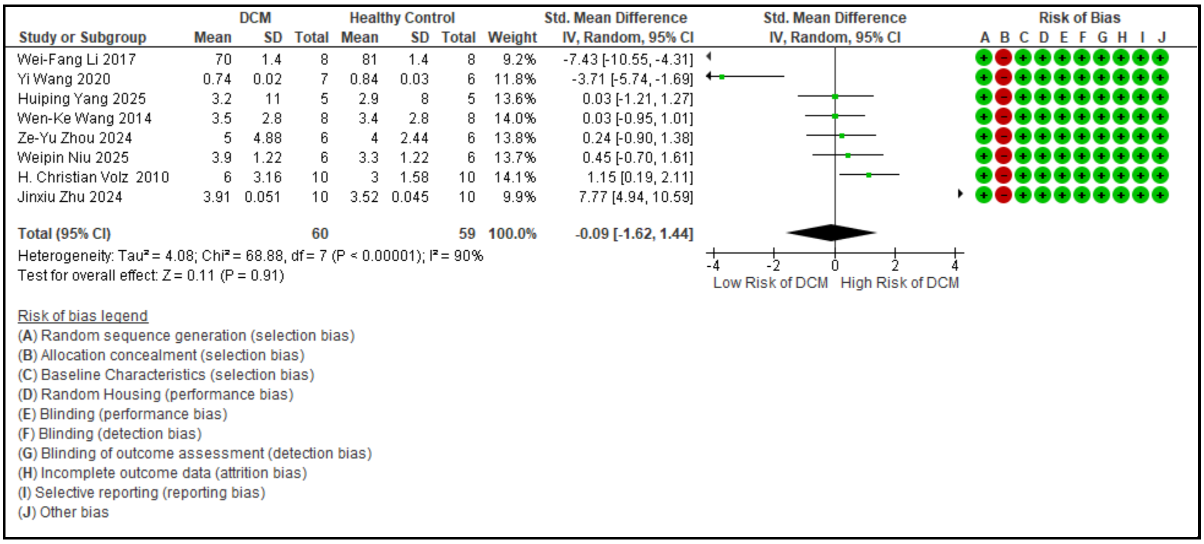

Figure S1-8: Forest plot of LVIDd for the calculation of the effect size or SMD.

Left Ventricular Internal Diameter at Systole (LVIDs)

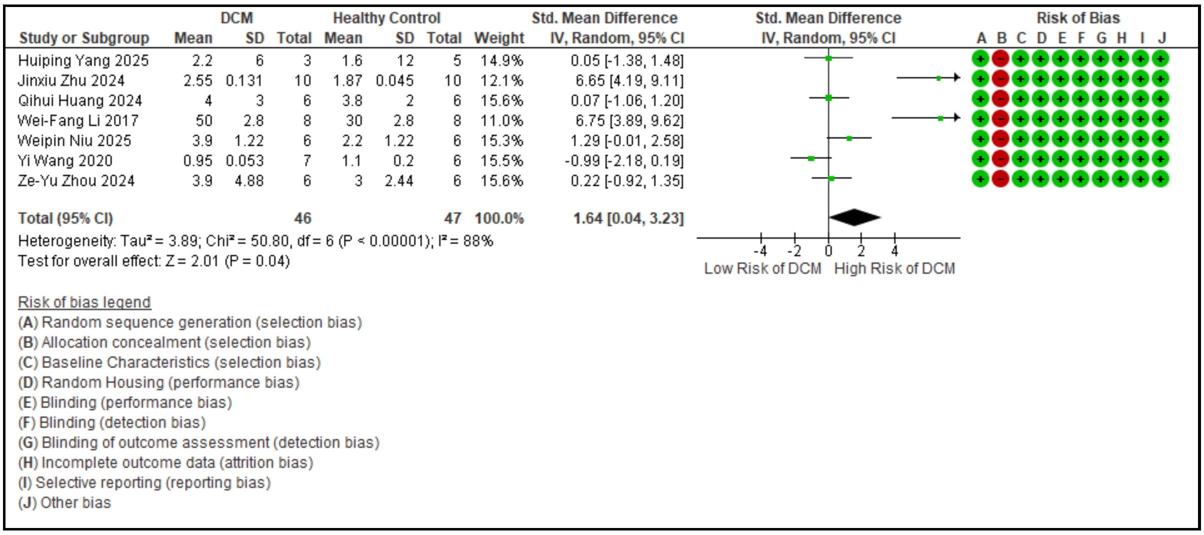

Figure S1-9: Forest plot of LVIDs for the calculation of the effect size or SMD.

Left Ventricular Diastolic Volume (LVDV)

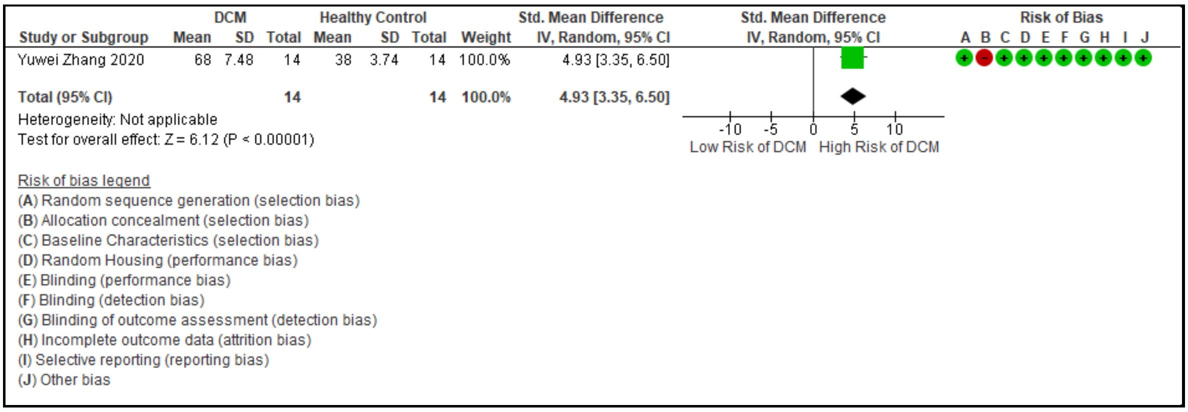

Figure S1-10: Forest plot of LVDV for the calculation of the effect size or SMD.

Left Ventricular Systolic Volume (LVSV)

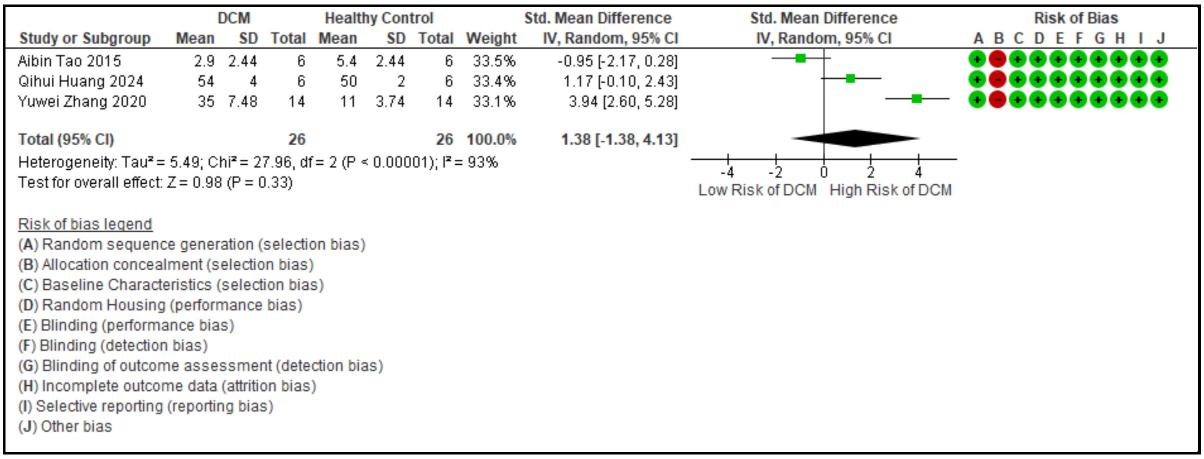

Figure S1-11: Forest plot of LVSV for the calculation of the effect size or SMD.

CREATINE KINASE – MUSCLE AND BRAIN (CK-MB)

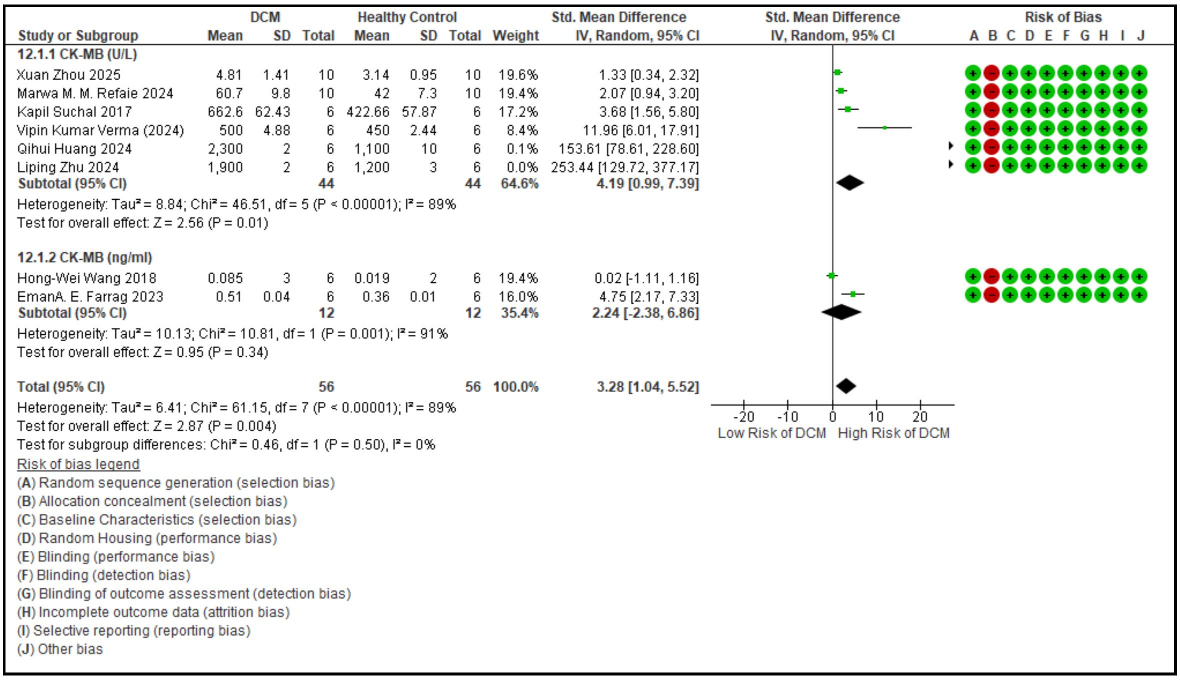

Figure S1-12: Forest plot of CK-MB for the calculation of the effect size or SMD.

Lactate Dehydrogenase (LDH)

Study or Subgroup	DCM Mean	DCM SD	DCM Total	Healthy Control Mean	Healthy Control SD	Healthy Control Total	Weight	Std. Mean Difference IV, Random, 95% CI	A	B	C	D	E	F	G	H	I	J
Xuan Zhou 2025	309.8	48.26	10	245.7	35.07	10	24.2%	1.46 [0.44, 2.47]	+	−	+	+	+	+	+	+	+	+
Qihui Huang 2024	20	7	6	10	2	6	23.7%	1.79 [0.36, 3.22]	+	−	+	+	+	+	+	+	+	+
Kapil Suchal 2017	764.98	69.29	6	505.94	53.65	6	22.2%	3.86 [1.66, 6.06]	+	−	+	+	+	+	+	+	+	+
Marwa M. M. Refaie 2024	271.1	18	10	150.6	8.8	10	20.5%	8.15 [5.20, 11.10]	+	−	+	+	+	+	+	+	+	+
Vipin Kumar Verma (2024)	350	2.44	6	300	2.44	6	7.9%	18.92 [9.61, 28.22]	+	−	+	+	+	+	+	+	+	+
EmanA. E. Farrag 2023	2,235	14.14	6	1,400	13.04	6	1.3%	56.67 [28.98, 84.36]	+	−	+	+	+	+	+	+	+	+
Huiping Yang 2025	1,000	2	3	850	1	3	0.2%	75.89 [2.59, 149.20]	+	−	+	+	+	+	+	+	+	+
**Total (95% CI)**			**47**			**47**	**100.0%**	**5.66 [2.48, 8.84]**										

Heterogeneity: Tau² = 10.59; Chi² = 51.04, df = 6 (P < 0.00001); I² = 88%
Test for overall effect: Z = 3.49 (P = 0.0005)

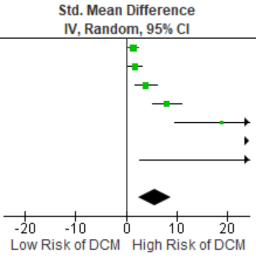

Risk of bias legend
(A) Random sequence generation (selection bias)
(B) Allocation concealment (selection bias)
(C) Baseline Characteristics (selection bias)
(D) Random Housing (performance bias)
(E) Blinding (performance bias)
(F) Blinding (detection bias)
(G) Blinding of outcome assessment (detection bias)
(H) Incomplete outcome data (attrition bias)
(I) Selective reporting (reporting bias)
(J) Other bias

Figure S1-13: Forest plot of LDH for the calculation of the effect size or SMD.

Cardiac Troponin (CTPN)

Study or Subgroup	DCM Mean	DCM SD	DCM Total	HC Mean	HC SD	HC Total	Weight	Std. Mean Difference IV, Random, 95% CI	A	B	C	D	E	F	G	H	I	J
Huiling Diao 2014	190	50	10	170	30	10	27.9%	0.46 [-0.43, 1.36]	+	−	+	+	+	+	+	+	+	+
Nawal M. Al-Rasheed 2017	400,000	8,400	8	90,000	5,600	8	26.2%	41.06 [24.64, 57.47]	+	−	+	+	+	+	+	+	+	+
Hong-Wei Wang 2018	400,000,000	10,000,000	6	40,000,000	1,000,000	6	24.7%	46.76 [23.91, 69.62]	+	−	+	+	+	+	+	+	+	+
Liping Zhu 2024	135	2	6	10	1	6	21.2%	72.98 [37.33, 108.62]	+	−	+	+	+	+	+	+	+	+
**Total (95% CI)**			**30**			**30**	**100.0%**	**37.93 [4.28, 71.58]**										

Heterogeneity: Tau² = 1056.98; Chi² = 54.89, df = 3 (P < 0.00001); I² = 95%
Test for overall effect: Z = 2.21 (P = 0.03)

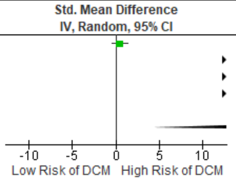

Risk of bias legend
(A) Random sequence generation (selection bias)
(B) Allocation concealment (selection bias)
(C) Baseline Characteristics (selection bias)
(D) Random Housing (performance bias)
(E) Blinding (performance bias)
(F) Blinding (detection bias)
(G) Blinding of outcome assessment (detection bias)
(H) Incomplete outcome data (attrition bias)
(I) Selective reporting (reporting bias)
(J) Other bias

Figure S1-14: Forest plot of CTN (pg/ml) for the calculation of the effect size or SMD.

Blood Pressure (BP)

Study or Subgroup	DCM Mean	DCM SD	DCM Total	Healthy Control Mean	Healthy Control SD	Healthy Control Total	Weight	Std. Mean Difference IV, Random, 95% CI	A	B	C	D	E	F	G	H	I	J
Wen-ke Wang 2017	102.75	14.3	8	108.24	13.9	8	35.1%	-0.37 [-1.36, 0.62]	+	−	+	+	+	+	+	+	+	+
Huiling Diao 2014	115	9	10	108	9	10	35.4%	0.74 [-0.17, 1.66]	+	−	+	+	+	+	+	+	+	+
Marwa M. M. Refaie 2024	164.5	12.1	10	107.1	8	10	29.4%	5.36 [3.31, 7.41]	+	−	+	+	+	+	+	+	+	+
**Total (95% CI)**			**28**			**28**	**100.0%**	**1.71 [-0.71, 4.13]**										

Heterogeneity: Tau² = 4.08; Chi² = 24.30, df = 2 (P < 0.00001); I² = 92%
Test for overall effect: Z = 1.39 (P = 0.17)

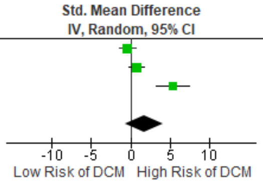

Risk of bias legend
(A) Random sequence generation (selection bias)
(B) Allocation concealment (selection bias)
(C) Baseline Characteristics (selection bias)
(D) Random Housing (performance bias)
(E) Blinding (performance bias)
(F) Blinding (detection bias)
(G) Blinding of outcome assessment (detection bias)
(H) Incomplete outcome data (attrition bias)
(I) Selective reporting (reporting bias)
(J) Other bias

Figure S1-15: Forest plot of BP for the calculation of the effect size or SMD.

Model 3 – Glycaemic Biomarkers

Blood Glucose (BG)

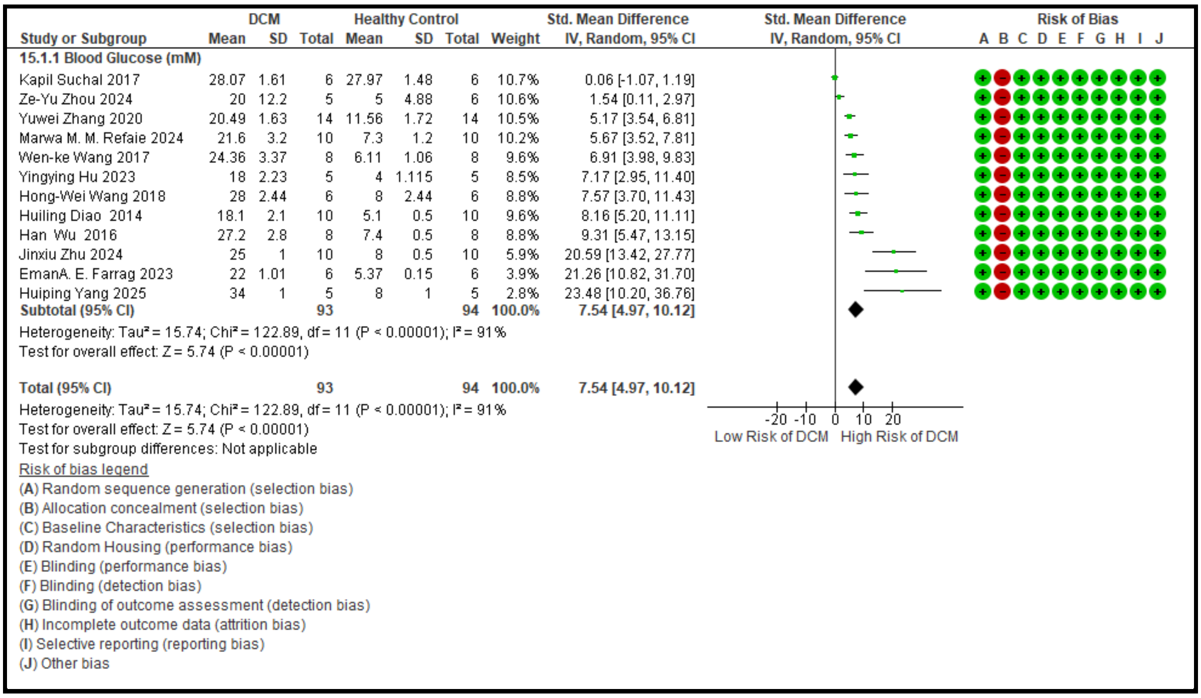

Figure S1-16: Forest plot of BG for the calculation of the effect size or SMD.

Serum Insulin (sINS)

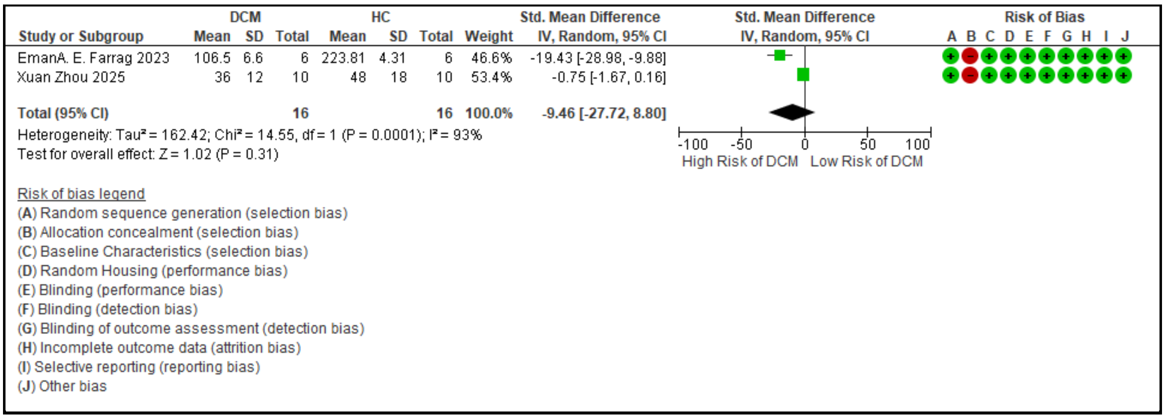

Figure S1-17: Forest plot of sINS for the calculation of the effect size or SMD.

Body Weight (BW)

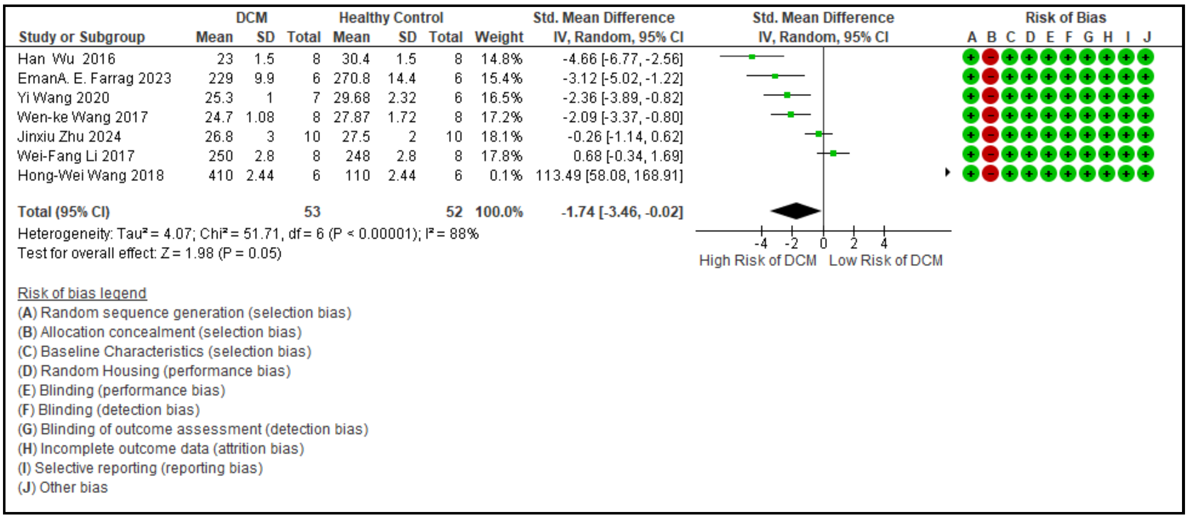

Study or Subgroup	DCM Mean	DCM SD	DCM Total	Healthy Control Mean	Healthy Control SD	Healthy Control Total	Weight	Std. Mean Difference IV, Random, 95% CI
Han Wu 2016	23	1.5	8	30.4	1.5	8	14.8%	-4.66 [-6.77, -2.56]
EmanA. E. Farrag 2023	229	9.9	6	270.8	14.4	6	15.4%	-3.12 [-5.02, -1.22]
Yi Wang 2020	25.3	1	7	29.68	2.32	6	16.5%	-2.36 [-3.89, -0.82]
Wen-ke Wang 2017	24.7	1.08	8	27.87	1.72	8	17.2%	-2.09 [-3.37, -0.80]
Jinxiu Zhu 2024	26.8	3	10	27.5	2	10	18.1%	-0.26 [-1.14, 0.62]
Wei-Fang Li 2017	250	2.8	8	248	2.8	8	17.8%	0.68 [-0.34, 1.69]
Hong-Wei Wang 2018	410	2.44	6	110	2.44	6	0.1%	113.49 [58.08, 168.91]
**Total (95% CI)**			**53**			**52**	**100.0%**	**-1.74 [-3.46, -0.02]**

Heterogeneity: Tau² = 4.07; Chi² = 51.71, df = 6 (P < 0.00001); I² = 88%
Test for overall effect: Z = 1.98 (P = 0.05)

Risk of bias legend
(A) Random sequence generation (selection bias)
(B) Allocation concealment (selection bias)
(C) Baseline Characteristics (selection bias)
(D) Random Housing (performance bias)
(E) Blinding (performance bias)
(F) Blinding (detection bias)
(G) Blinding of outcome assessment (detection bias)
(H) Incomplete outcome data (attrition bias)
(I) Selective reporting (reporting bias)
(J) Other bias

Figure S1-18: Forest plot of BW for the calculation of the effect size or SMD.

Model 4 - Lipid Biomarkers

Total Cholesterol (TC)

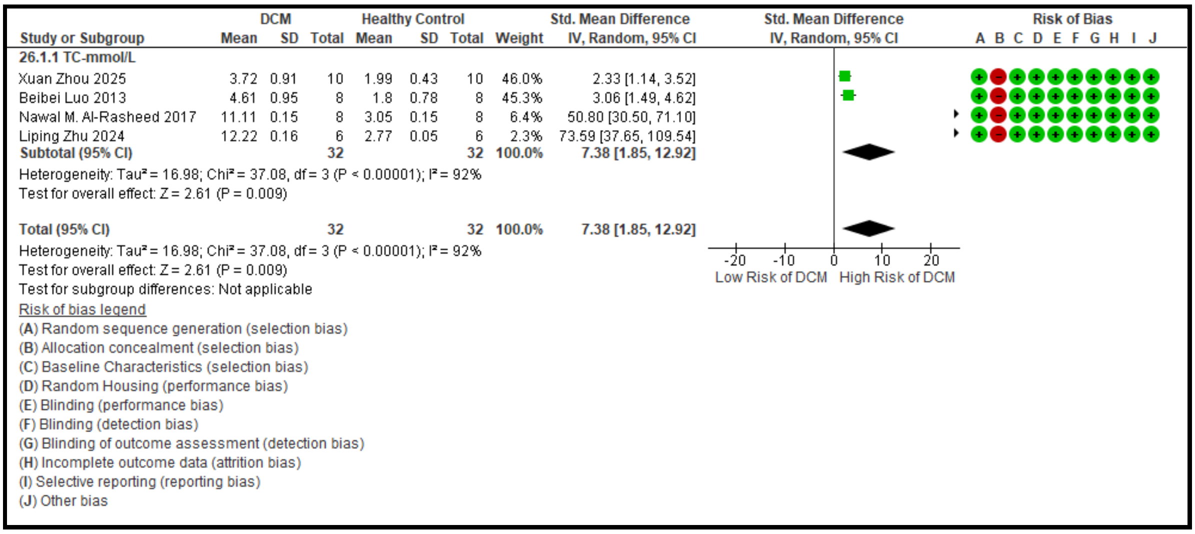

Study or Subgroup	DCM Mean	DCM SD	DCM Total	Healthy Control Mean	Healthy Control SD	Healthy Control Total	Weight	Std. Mean Difference IV, Random, 95% CI
**26.1.1 TC-mmol/L**								
Xuan Zhou 2025	3.72	0.91	10	1.99	0.43	10	46.0%	2.33 [1.14, 3.52]
Beibei Luo 2013	4.61	0.95	8	1.8	0.78	8	45.3%	3.06 [1.49, 4.62]
Nawal M. Al-Rasheed 2017	11.11	0.15	8	3.05	0.15	8	6.4%	50.80 [30.50, 71.10]
Liping Zhu 2024	12.22	0.16	6	2.77	0.05	6	2.3%	73.59 [37.65, 109.54]
**Subtotal (95% CI)**			**32**			**32**	**100.0%**	**7.38 [1.85, 12.92]**
**Total (95% CI)**			**32**			**32**	**100.0%**	**7.38 [1.85, 12.92]**

Subtotal: Heterogeneity: Tau² = 16.98; Chi² = 37.08, df = 3 (P < 0.00001); I² = 92%
Test for overall effect: Z = 2.61 (P = 0.009)

Total: Heterogeneity: Tau² = 16.98; Chi² = 37.08, df = 3 (P < 0.00001); I² = 92%
Test for overall effect: Z = 2.61 (P = 0.009)
Test for subgroup differences: Not applicable

Risk of bias legend
(A) Random sequence generation (selection bias)
(B) Allocation concealment (selection bias)
(C) Baseline Characteristics (selection bias)
(D) Random Housing (performance bias)
(E) Blinding (performance bias)
(F) Blinding (detection bias)
(G) Blinding of outcome assessment (detection bias)
(H) Incomplete outcome data (attrition bias)
(I) Selective reporting (reporting bias)
(J) Other bias

Figure S1-19: Forest plot of TC for the calculation of the effect size or SMD.

Total Triglycerides (TG)

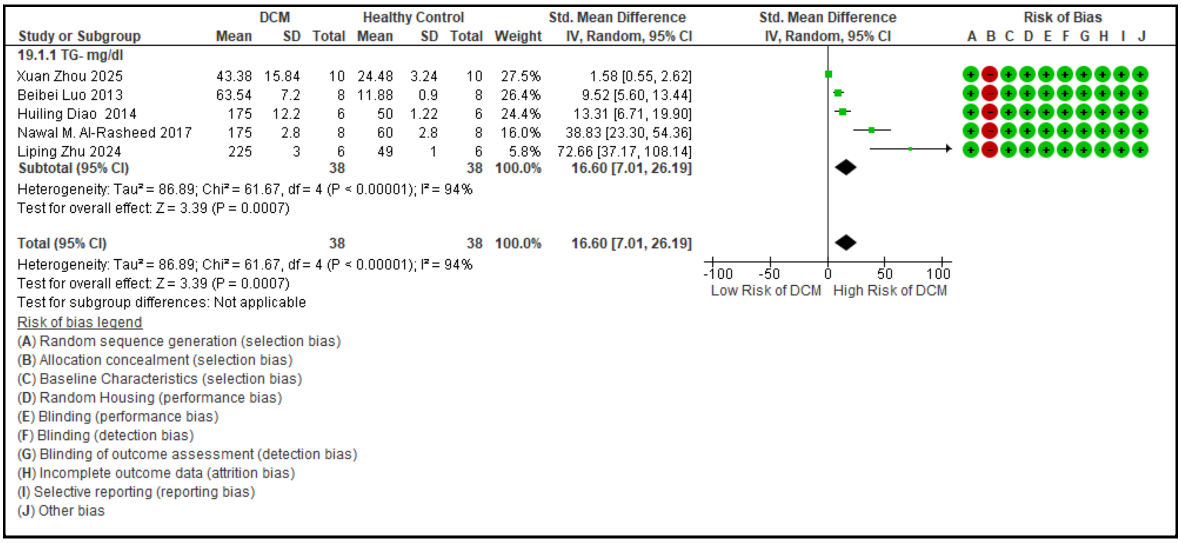

Figure S1-20: Forest plot of TG for the calculation of the effect size or SMD.

High Density Lipoprotein Cholesterol (HDL-C)

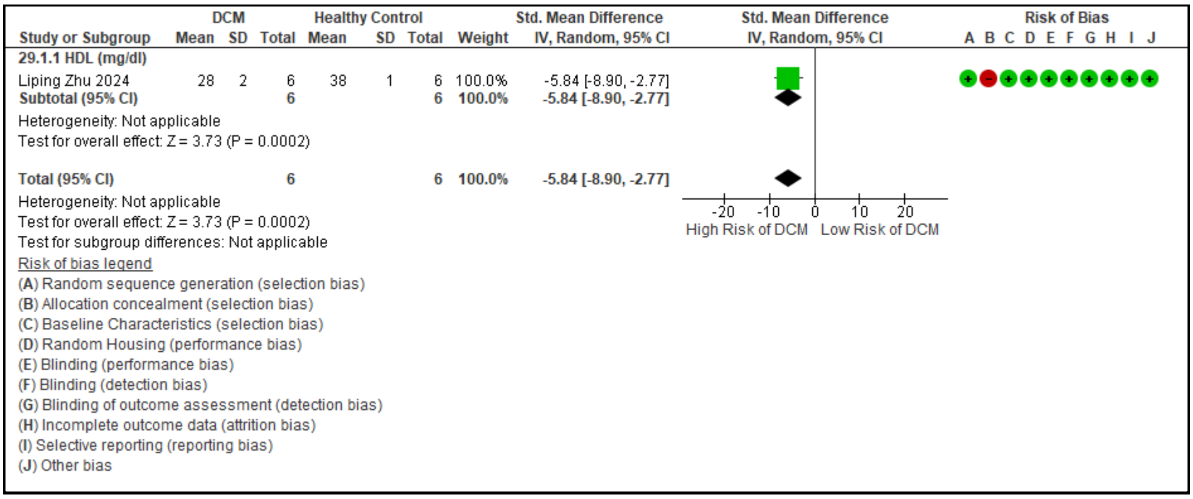

Figure S1-21: Forest plot of HDL for the calculation of the effect size or SMD.

Low Density Lipoprotein Cholesterol (LDL-C)

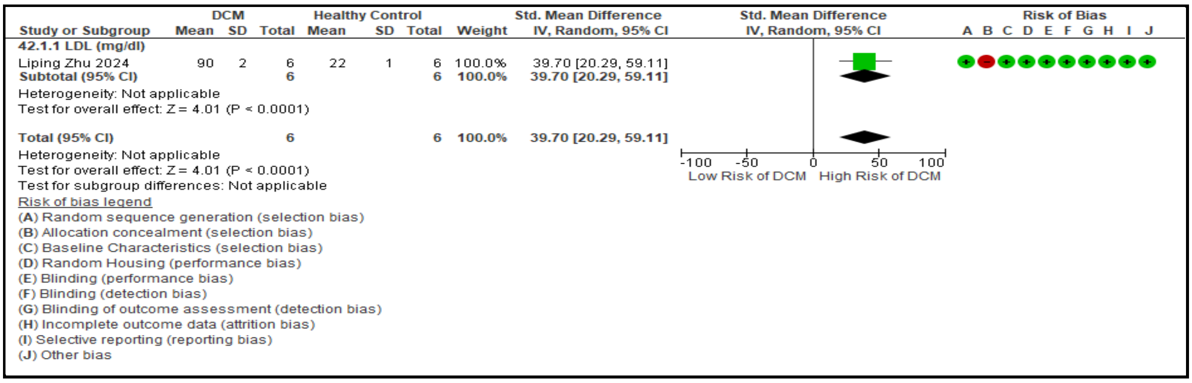

Figure S1-22: Forest plot of LDL for the calculation of the effect size or SMD.

Model 5 - Oxidative Stress Biomarkers

Glutathione (GSH)

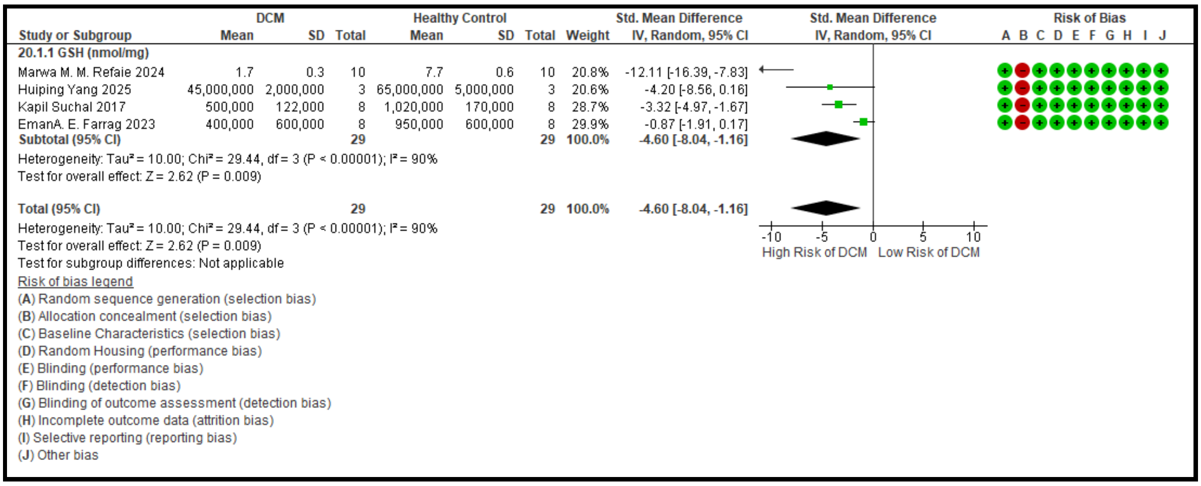

Figure S1-23: Forest plot of GSH for the calculation of the effect size or SMD.

Malondialdehyde (MDA)

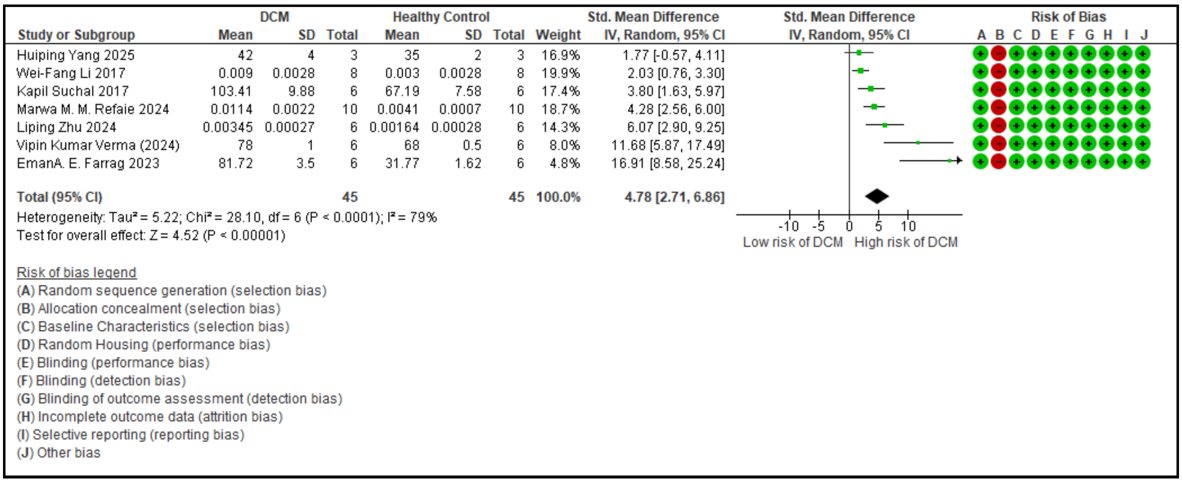

Figure S1-24: Forest plot of MDA for the calculation of the effect size or SMD.

Model 6 - Inflammatory Biomarkers

Tumour Necrosis Factor – Alpha (TNF-A)

Study or Subgroup	DCM Mean	DCM SD	DCM Total	Healthy Control Mean	Healthy Control SD	Healthy Control Total	Weight	Std. Mean Difference IV, Random, 95% CI
**23.1.1 TNF-A (mRNA FOLD IN CARDIAC TX)**								
Yuwei Zhang 2020	5	14.8	14	1	3.7	14	17.8%	0.36 [-0.39, 1.11]
**Subtotal (95% CI)**			**14**			**14**	**17.8%**	**0.36 [-0.39, 1.11]**
Heterogeneity: Not applicable								
Test for overall effect: Z = 0.94 (P = 0.35)								
**23.1.2 TNF-A in serum (pg/ml)**								
Wen-ke Wang 2017	350,000	3,000,000	8	100,000	2,000,000	8	16.6%	0.09 [-0.89, 1.07]
Huiling Diao 2014	27,000	7,000	10	25,000	5,000	10	17.1%	0.31 [-0.57, 1.20]
Xuan Zhou 2025	92.61	11.05	10	80.4	4.44	10	16.5%	1.39 [0.39, 2.39]
Marwa M. M. Refaie 2024	57.5	10.6	10	29.1	4	10	13.9%	3.40 [1.93, 4.86]
**Subtotal (95% CI)**			**38**			**38**	**64.2%**	**1.20 [-0.03, 2.43]**
Heterogeneity: Tau² = 1.26; Chi² = 16.34, df = 3 (P = 0.0010); I² = 82%								
Test for overall effect: Z = 1.91 (P = 0.06)								
**23.1.3 TNF-A-in cardiac tx (pg/mg)**								
Liping Zhu 2024	35.62	0.82	6	17.46	1.18	6	1.4%	16.50 [8.37, 24.63]
**Subtotal (95% CI)**			**6**			**6**	**1.4%**	**16.50 [8.37, 24.63]**
Heterogeneity: Not applicable								
Test for overall effect: Z = 3.98 (P < 0.0001)								
**23.1.4 β-Actin**								
Han Wu 2016	1.6	3	8	1	1	8	16.6%	0.25 [-0.73, 1.24]
**Subtotal (95% CI)**			**8**			**8**	**16.6%**	**0.25 [-0.73, 1.24]**
Heterogeneity: Not applicable								
Test for overall effect: Z = 0.50 (P = 0.61)								
**Total (95% CI)**			**66**			**66**	**100.0%**	**1.11 [0.11, 2.11]**
Heterogeneity: Tau² = 1.31; Chi² = 33.10, df = 6 (P < 0.0001); I² = 82%								
Test for overall effect: Z = 2.17 (P = 0.03)								
Test for subgroup differences: Chi² = 16.43, df = 3 (P = 0.0009), I² = 81.7%								

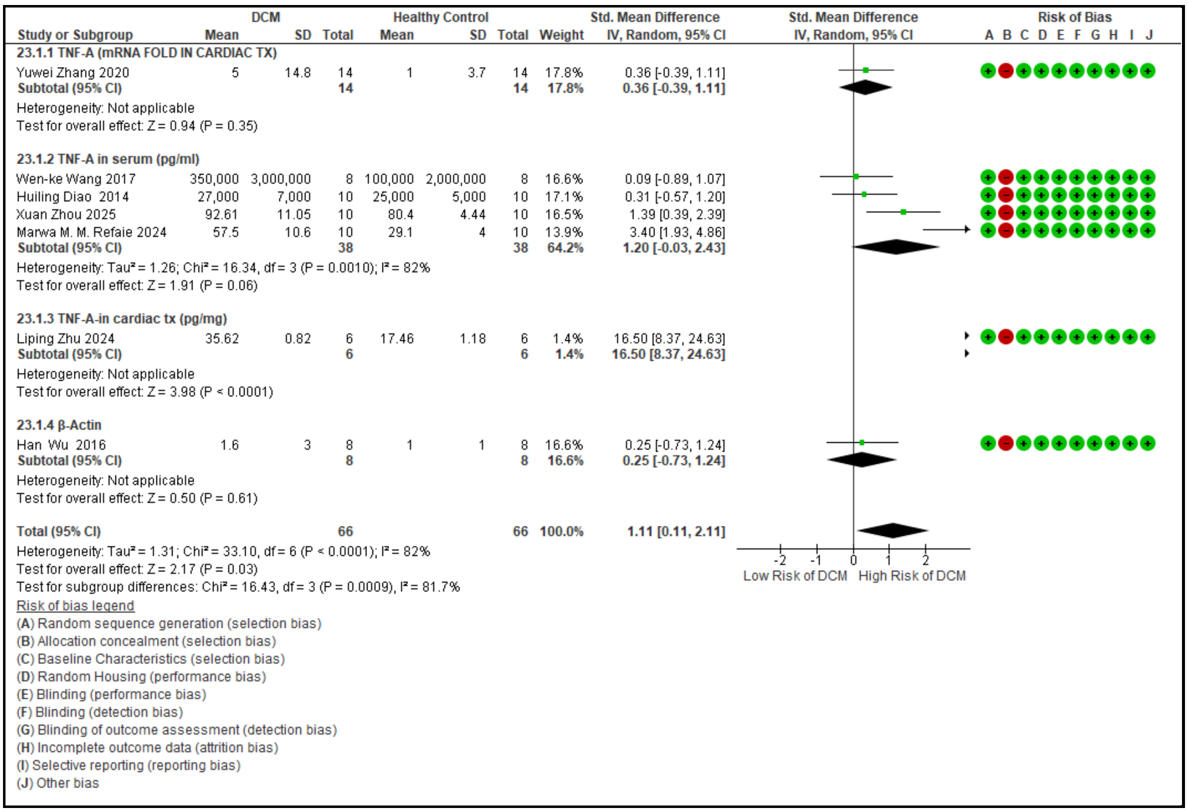

Risk of bias legend
(A) Random sequence generation (selection bias)
(B) Allocation concealment (selection bias)
(C) Baseline Characteristics (selection bias)
(D) Random Housing (performance bias)
(E) Blinding (performance bias)
(F) Blinding (detection bias)
(G) Blinding of outcome assessment (detection bias)
(H) Incomplete outcome data (attrition bias)
(I) Selective reporting (reporting bias)
(J) Other bias

Figure S1-25: Forest plot of TNF-a for the calculation of the effect size or SMD.

Interleukin -Six (IL-6)

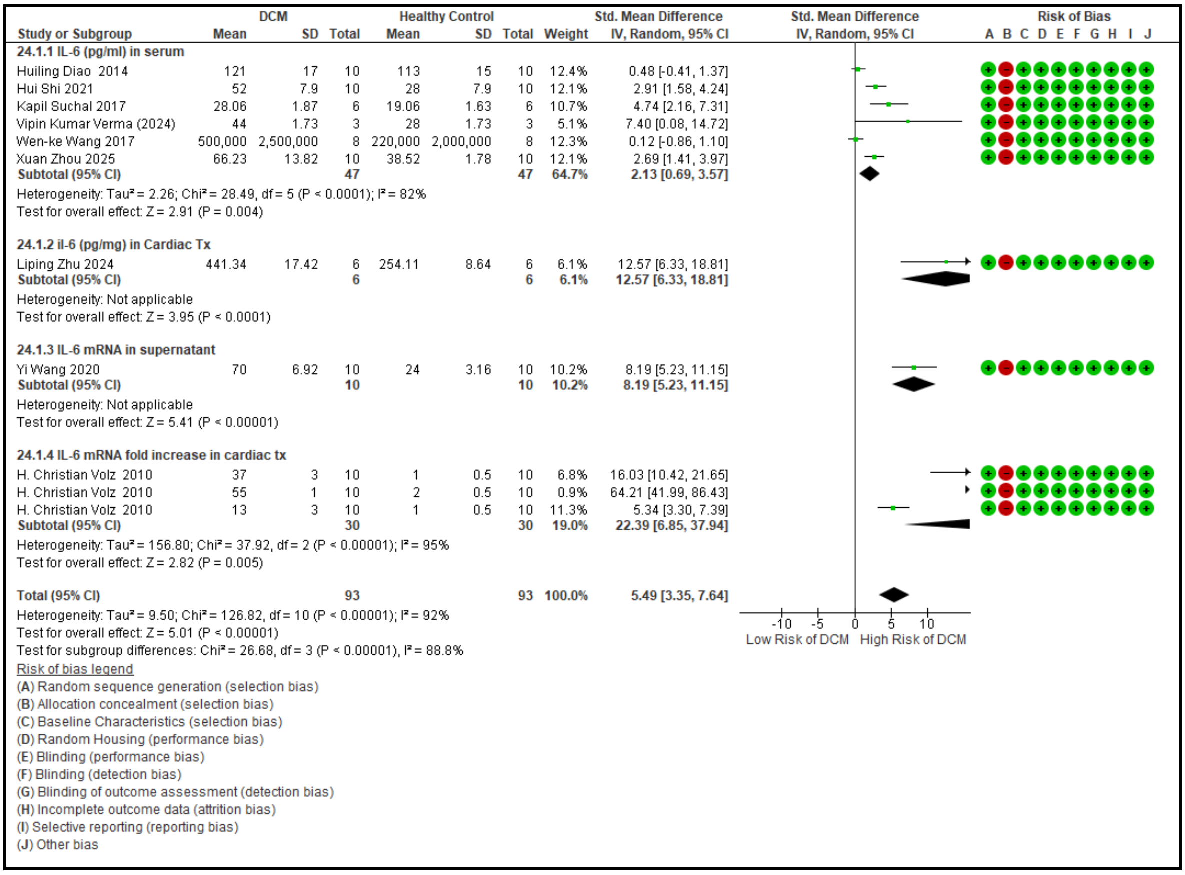

Figure S1-26: Forest plot of IL-6 for the calculation of the effect size or SMD.

Interleukin -One Beta (IL-1β)

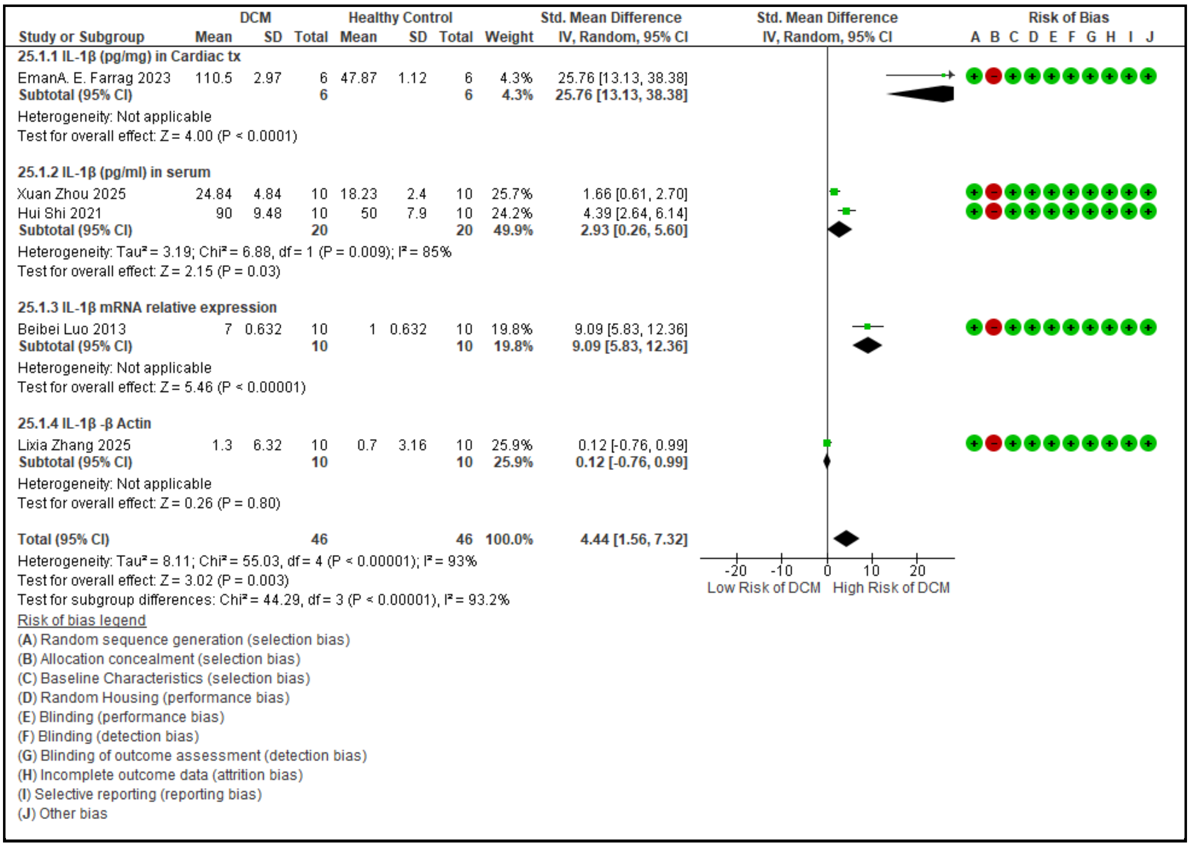

Figure S1-27: Forest plot of IL-1β for the calculation of the effect size or SMD.

Model 7 - Signalling Pathway Biomarkers

Nuclear Factor -Kappa Beta (NF-kB)

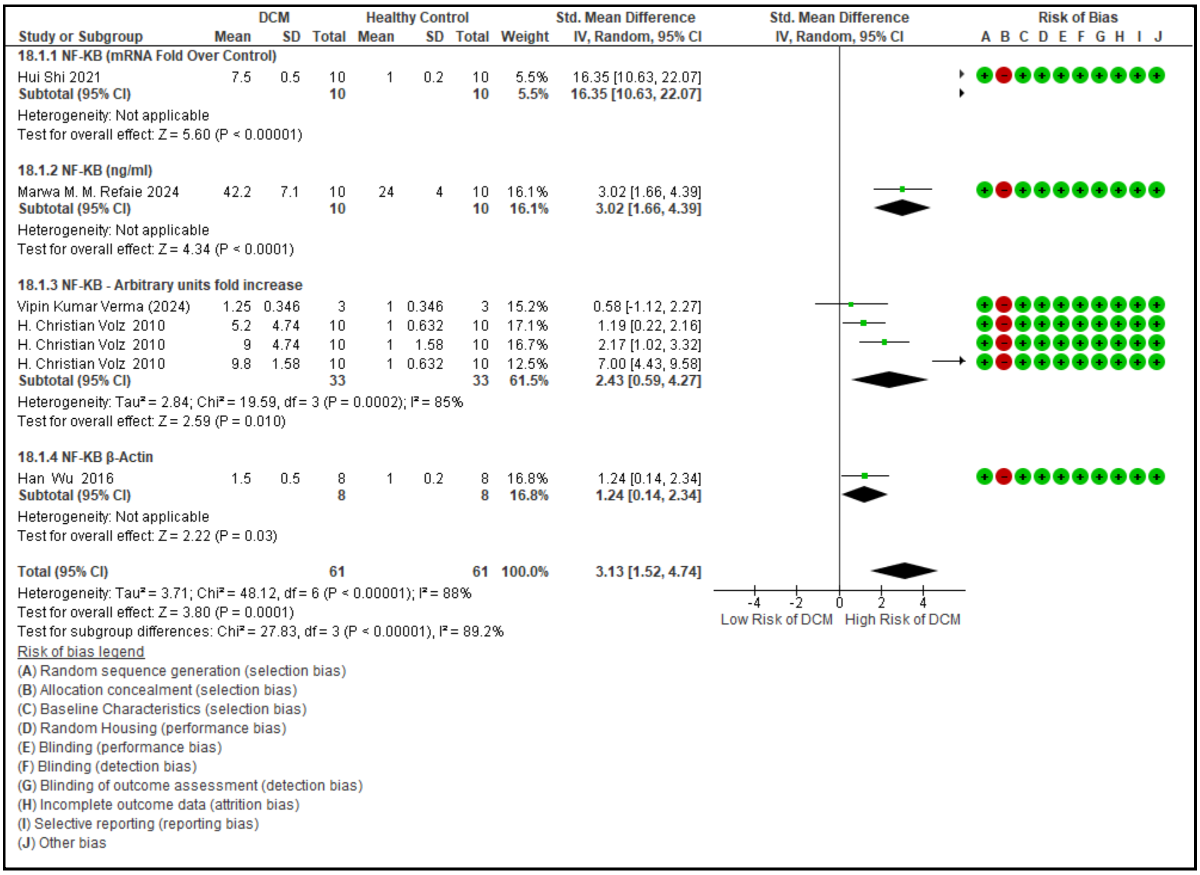

Figure S1-28: Forest plot of NF-kB for the calculation of the effect size or SMD.

Toll-Like Receptor -Four (TLR-4)

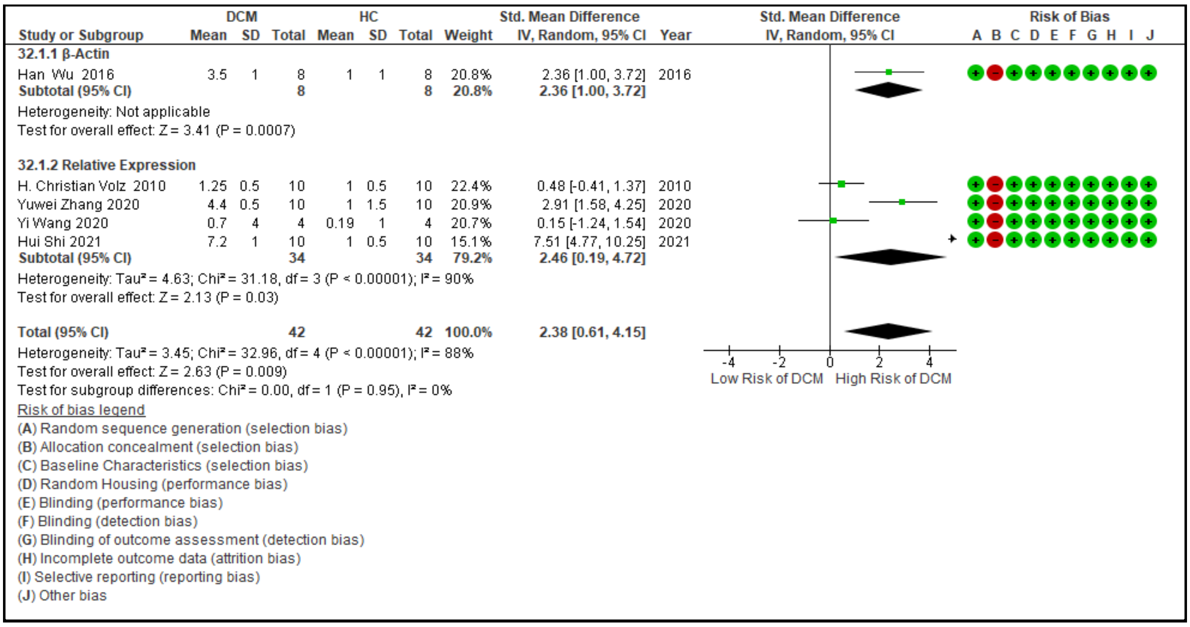

Figure S1-29: Forest plot of TLR4 for the calculation of the effect size or SMD.

Cleaved Caspase- 3 (CC3)

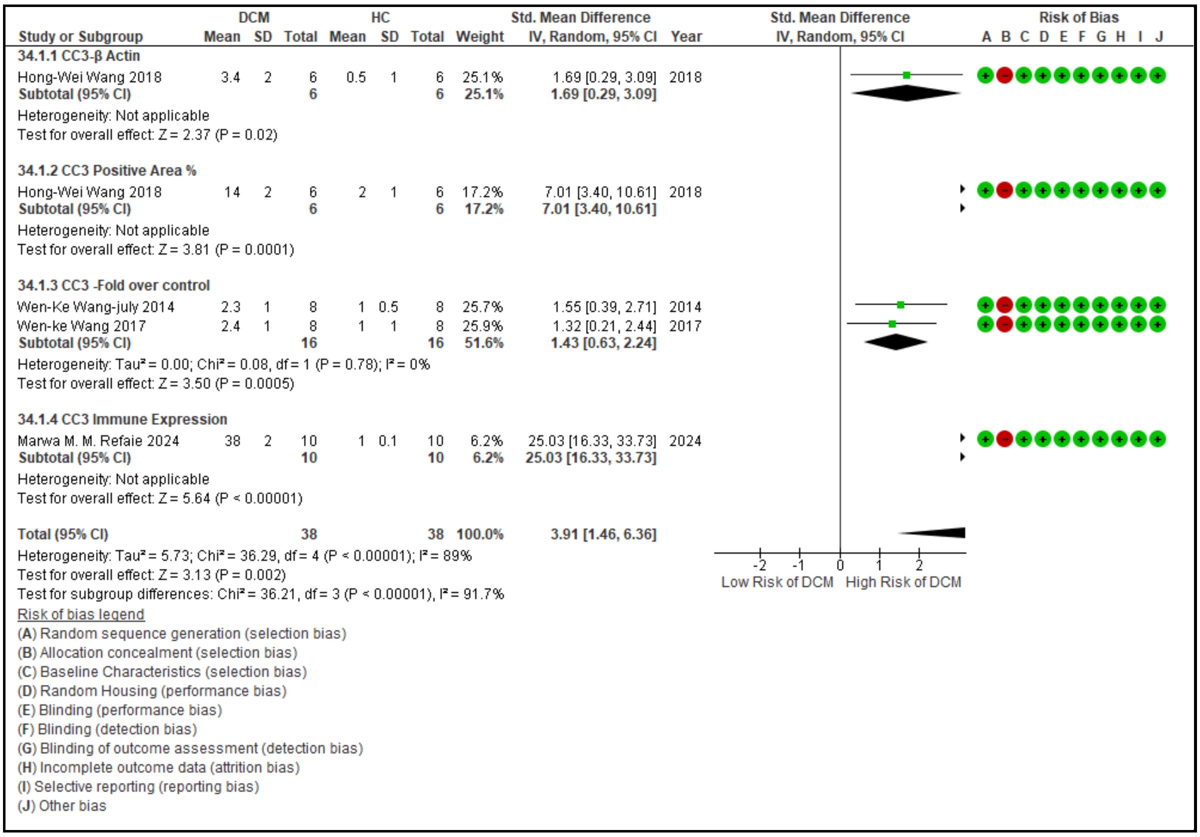

Figure S1-30: Forest plot of CC3 for the calculation of the effect size or SMD.

NLR family pyrin domain containing - 3 (NLRP3)

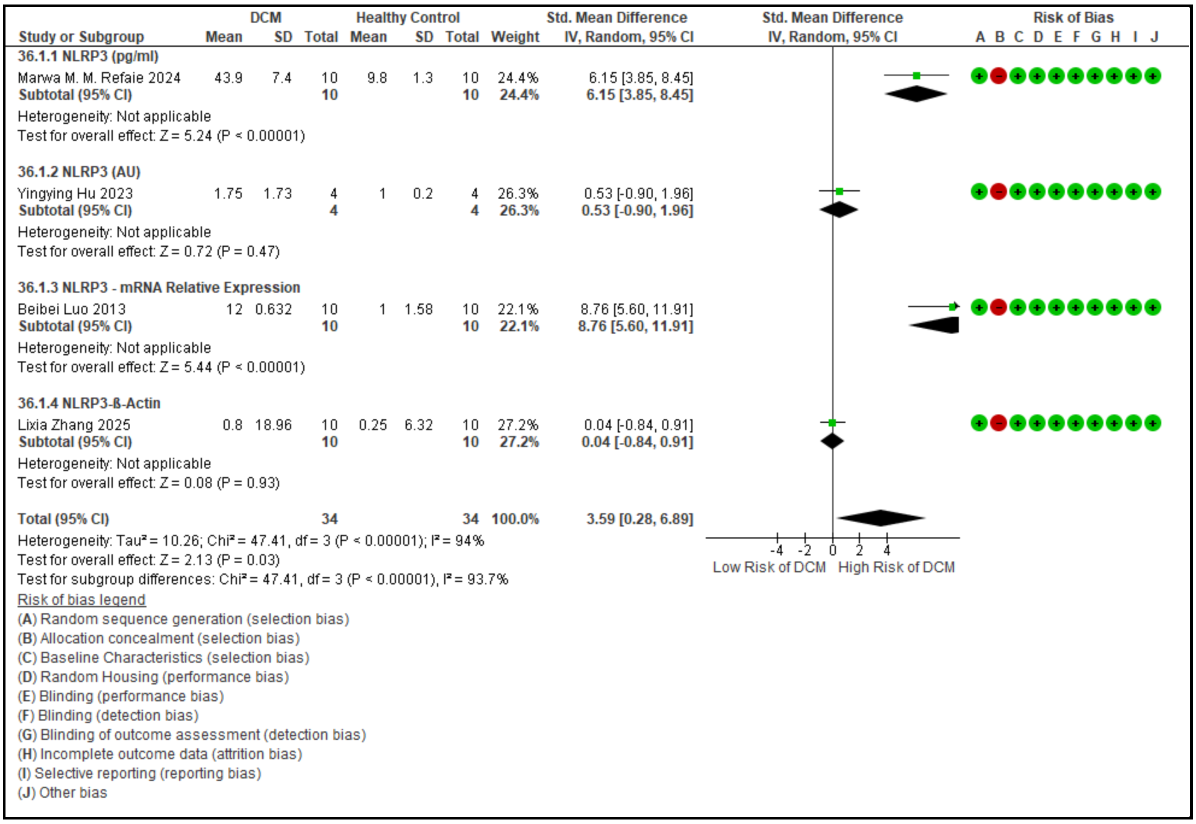

Figure S1-31: Forest plot of NLRP3 for the calculation of the effect size or SMD.

pERK 1/2 / t-ERK 1/ 2

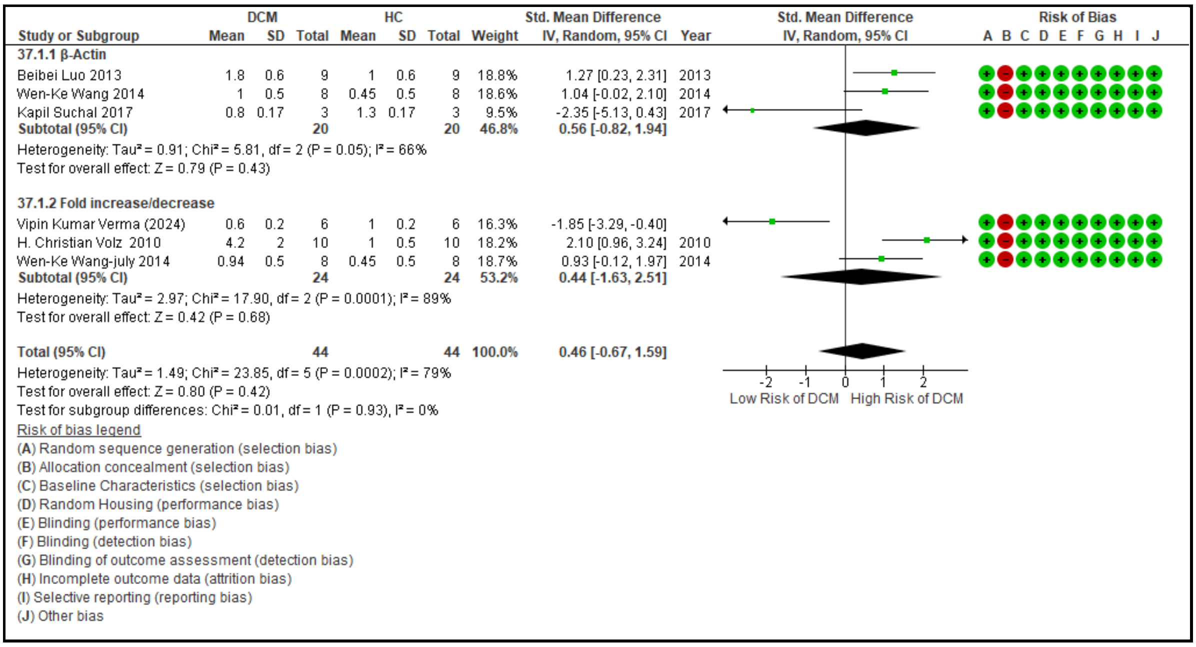

Figure S1-32: Forest plot of pERK1/2/t-ERK1/2 Ratio for the calculation of the effect size or SMD.

p-JNK / t-JNK

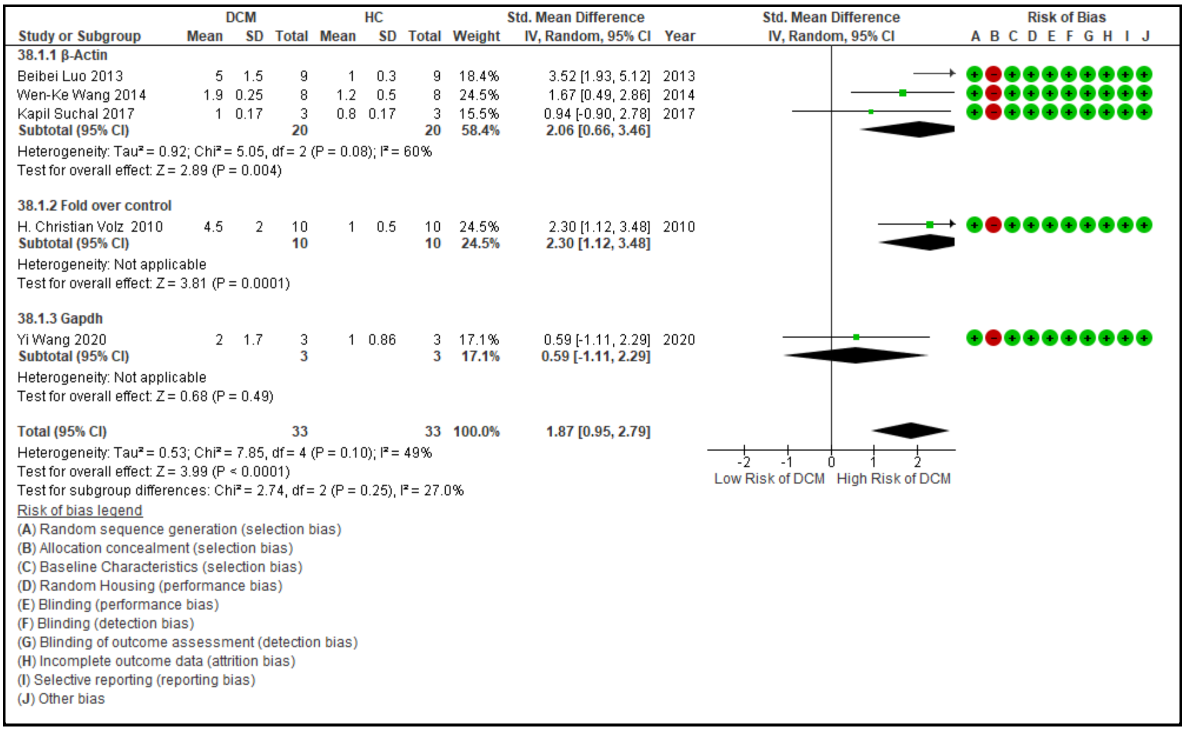

Figure S1-33: Forest plot of pJNK/t-JNK Ratio for the calculation of the effect size or SMD.

Transforming Growth Factor - Beta (TGF-β)

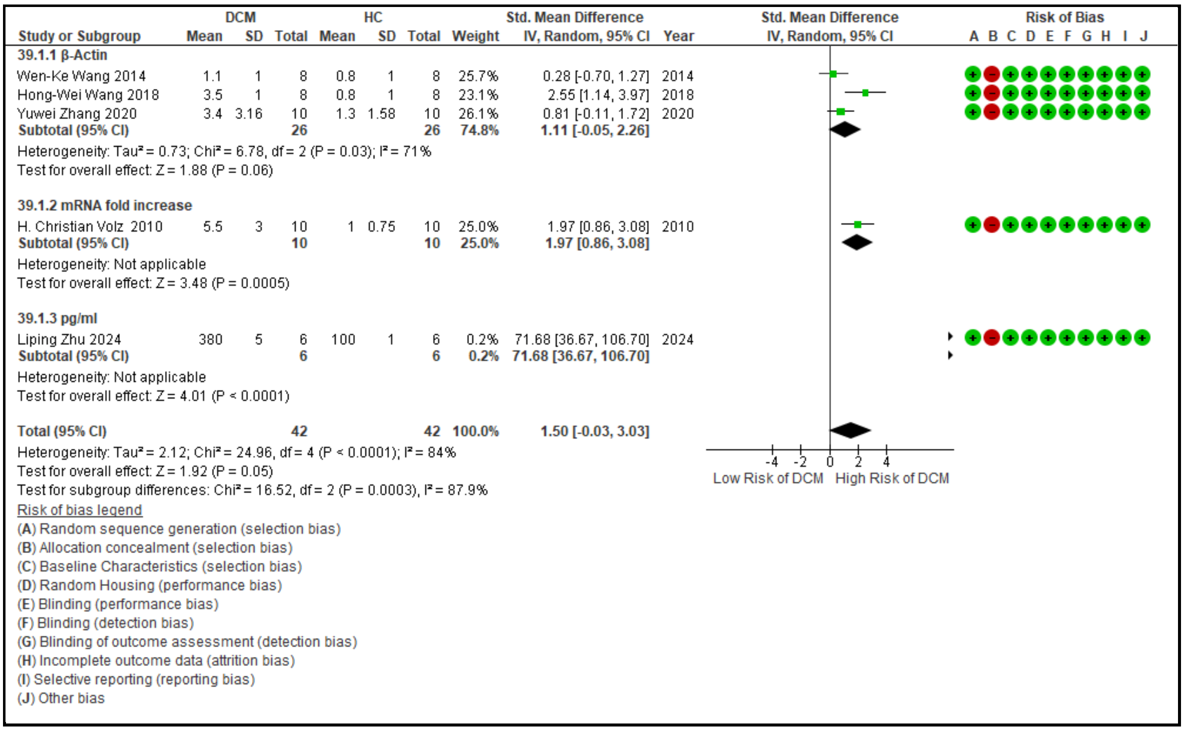

Figure S1-34: Forest plot of TGF-β calculation of the effect size or SMD.

Model 8 - Fibrosis Biomarker

Fibrosis percentage (FB%)

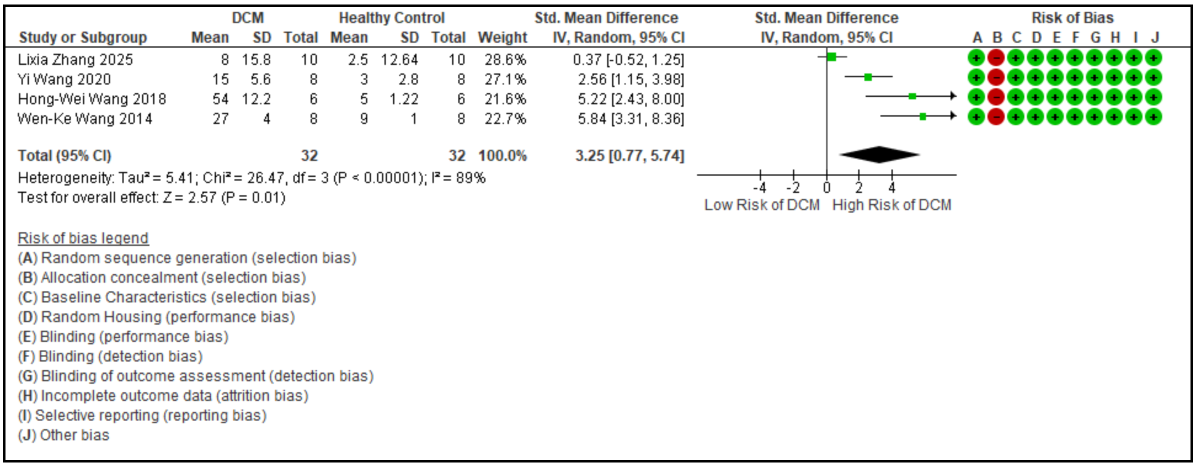

Figure S1-35: Forest plot of FB% for the calculation of the effect size or SMD.

Collagen I (Col I)

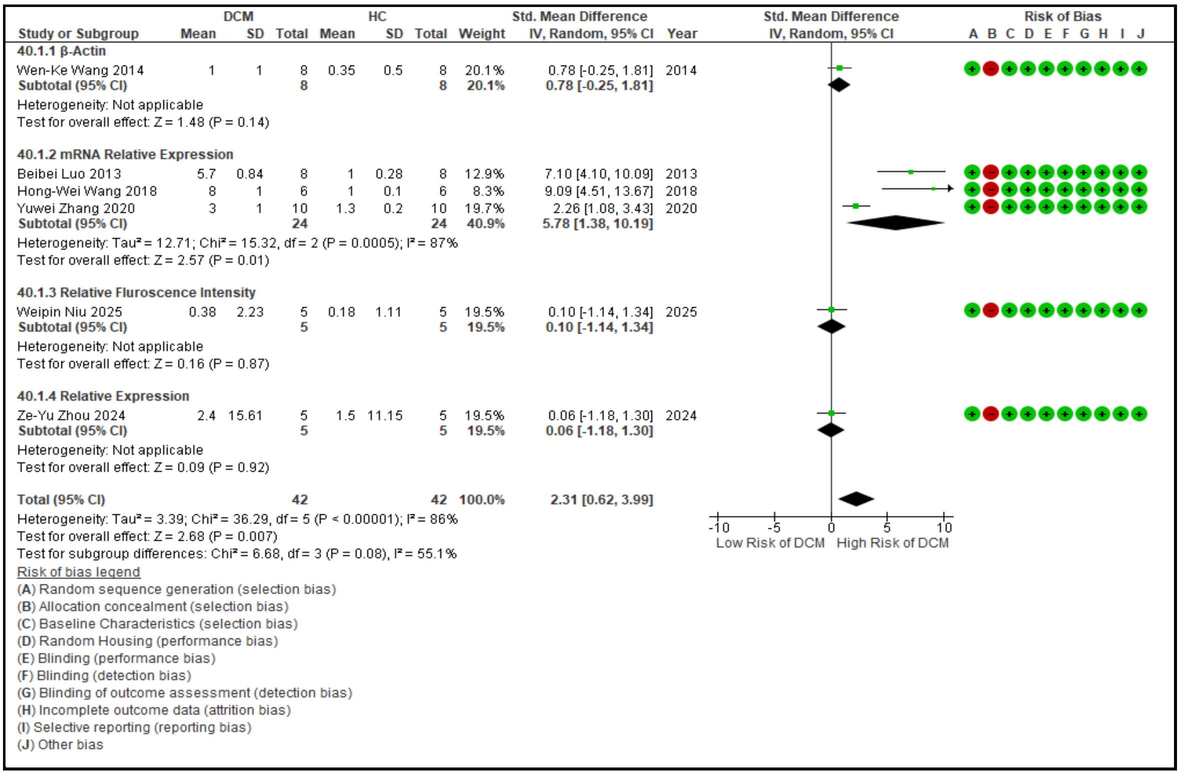

Figure S1-36: Forest plot of Collagen I for the calculation of the effect size or SMD.

Collagen III (Col III)

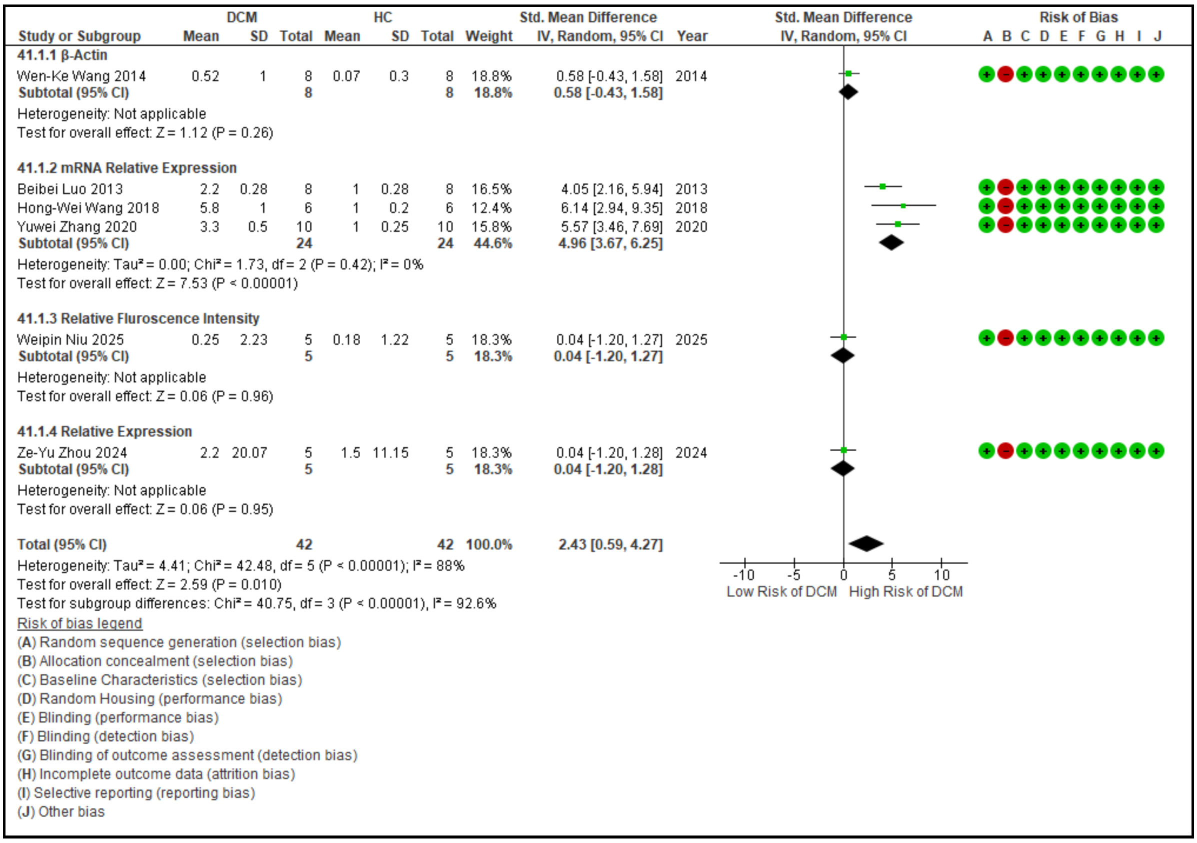

Figure S1-37: Forest plot of Collagen III for the calculation of the effect size or SMD.

Legend: Figure S1-1 to S1-37 are forest plots constructed using data pertaining to the biomarkers tested in this study. 37 biomarkers are grouped into 8 models: *viz*, the AGEs, cardiometabolic, glycaemic, lipid, oxidative stress, inflammatory, signalling pathway -related and fibrosis %. Out of the 37 biomarkers, 30 showed significant increases or decreases as per their metabolic activities, signifying high risk of manifesting DCM in the rodent models tested. The number of biomarkers that produced significant differences in each model consisted of; **2**/ 2 in model 1, **9**/13 in model 2, **2** /3 in model 3, **4** /4 in model 4, **2** /2 in model 5, **3**/3 in model 6, **5**/7 in model 7 and **3** /3 in model 8. An overall 81% of high risk for developing DCM has been indicated in this study out of the 37 biomarkers that were assessed. The forest plots calculated the effect size or the standardized mean difference (SMD) and the 95% confidence limits plus the heterogeneity tests: Tau squared, Chi squared and the **I** squared tests and their probabilities. The overall effect of the outcome was given by the Z score and its probability. These were all continuous data that followed a random effects model.
