# Supplementary material for: Investigating the biomarkers of diabetic-cardiomyopathy with the high mobility group box-1 as a potential anti-inflammatory therapeutic target: Systematic Review and meta-analysis
Source: Front Endocrinol (Lausanne). 2026 Jan 14;16:1714219. doi: 10.3389/fendo.2025.1714219 (PMC12846985; doi:10.3389/fendo.2025.1714219)
Supplement: Supplementary file 2 [file DataSheet2.pdf]

SUPPLEMENTARY SECTION 2: FUNNEL PLOTS OF THE BIOMARKERS.

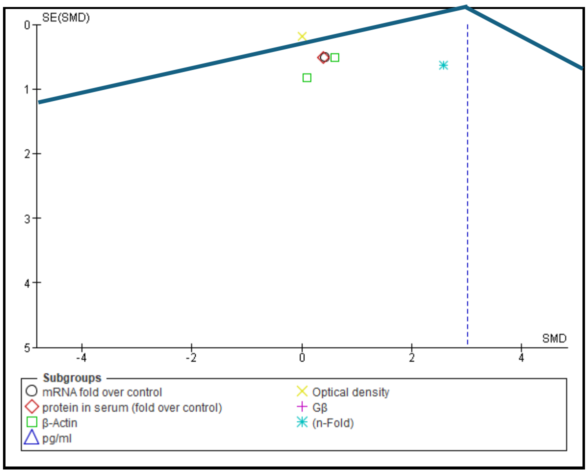

Fig. S2-1 HMGB1

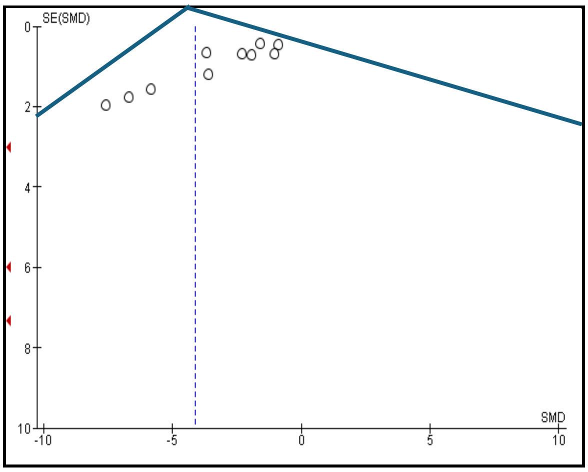

Fig. S2-2 EF%

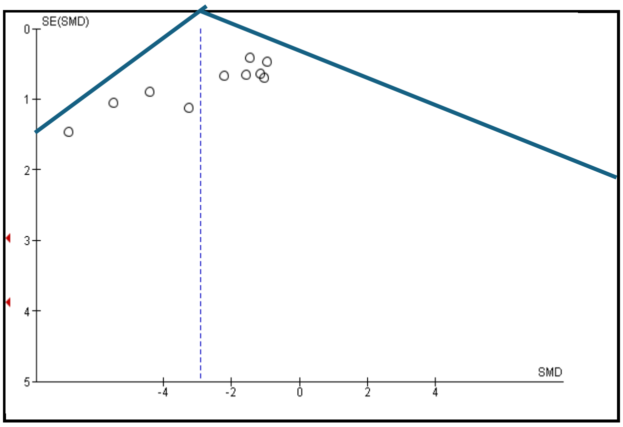

Fig. S2-3 FS%

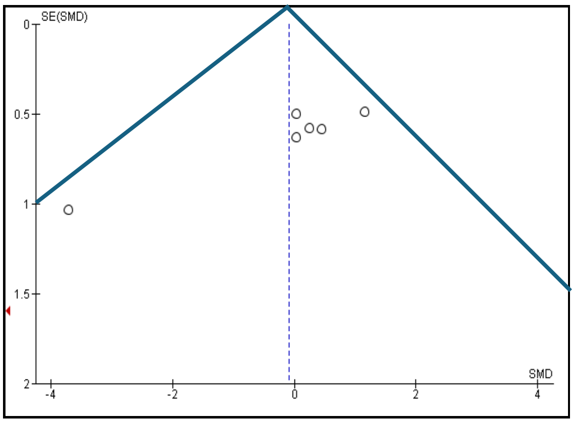

Fig. S2-4 LVIDD

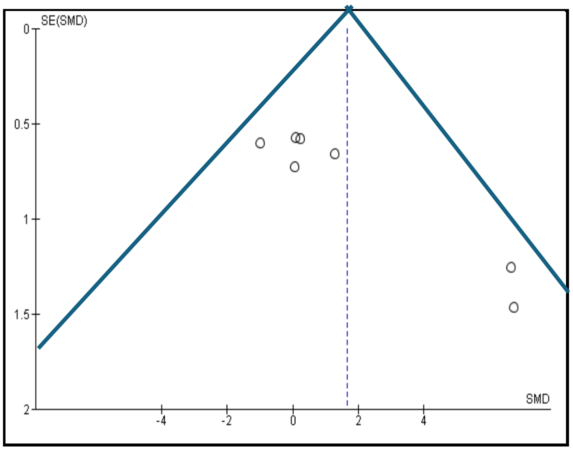

Fig. S2- 5 LVIDS

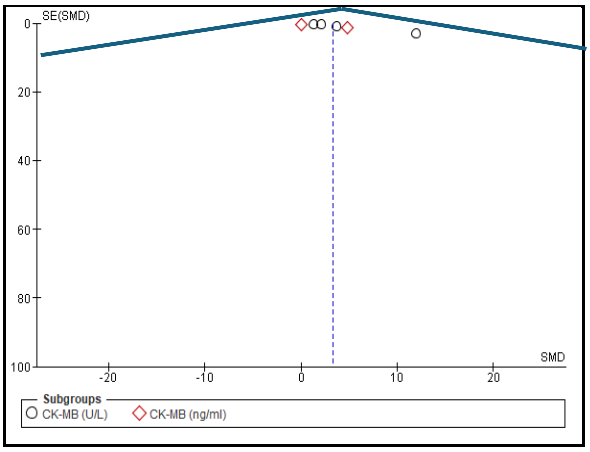

Fig. S2-6 CK-MB

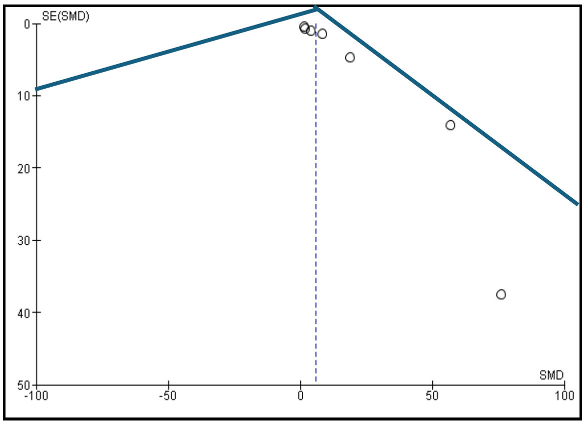

Fig. S2- 7 LDH

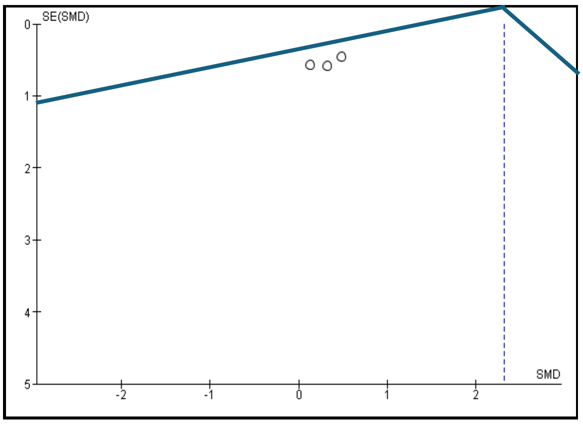

Fig. S2-8 HW/BW

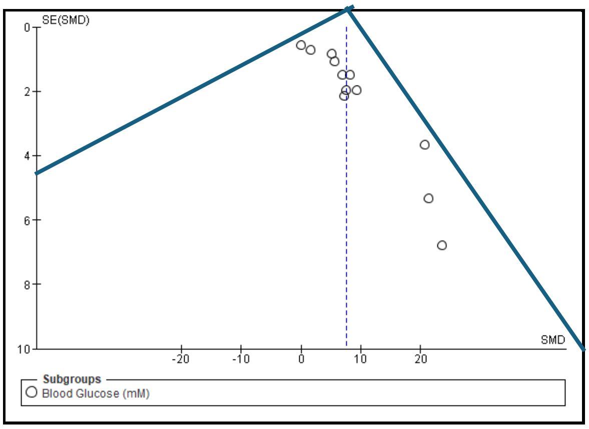

Fig. S2-9 BG

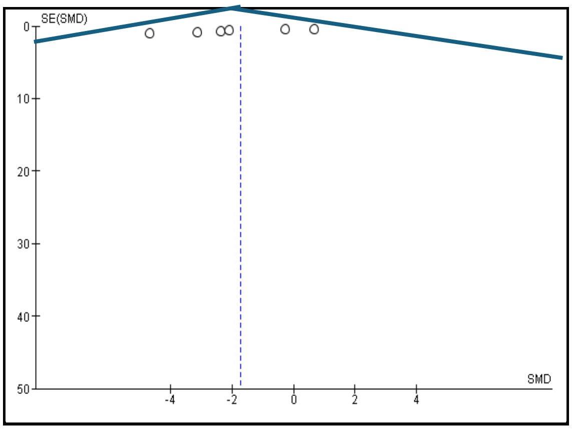

Fig. S2 -10 BW

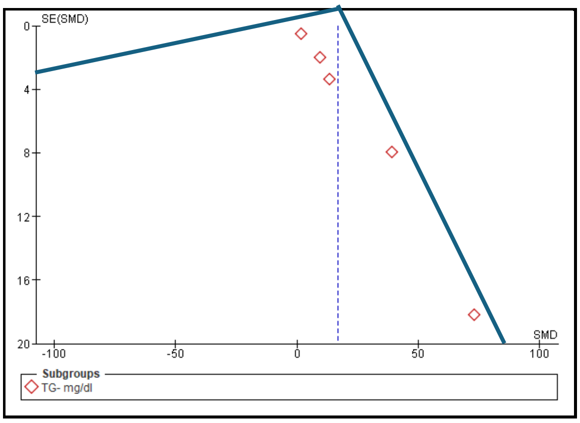

Fig. S2-11 TG

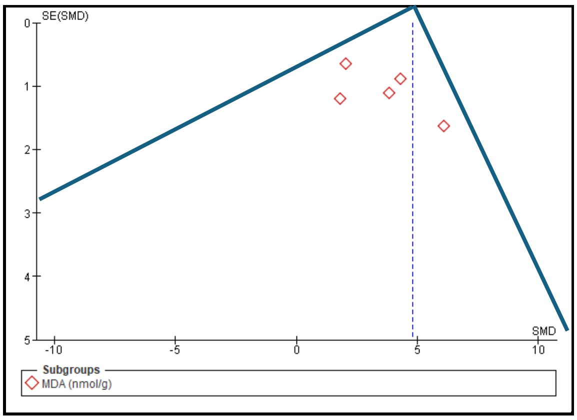

Fig. S2- 12 MDA

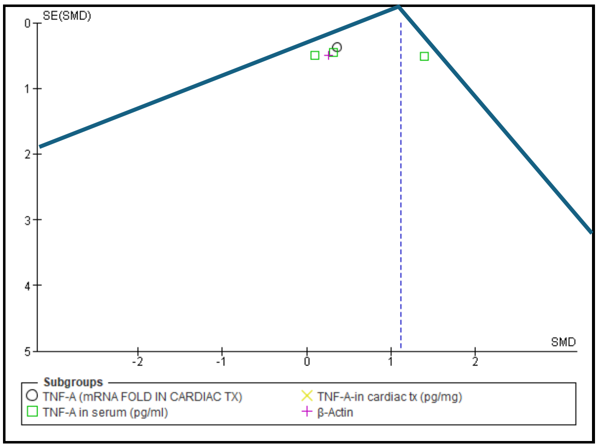

Fig. S2-13 TNF-A

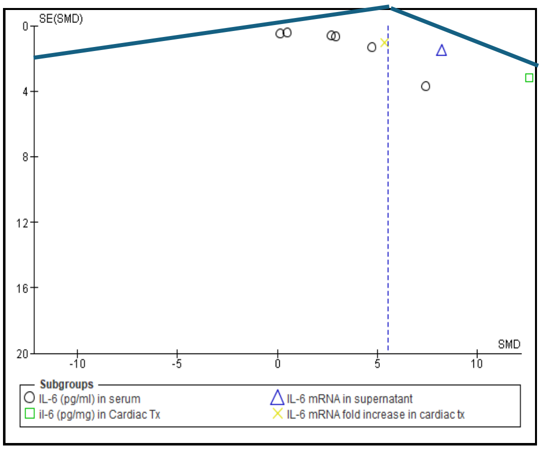

Fig. S2-14 IL-6

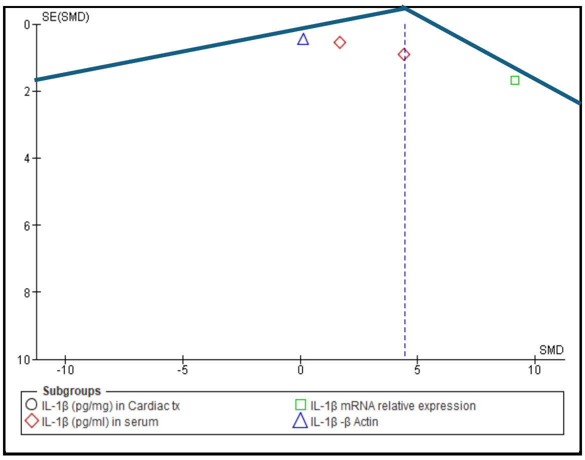

Fig. S2-15 IL-1β

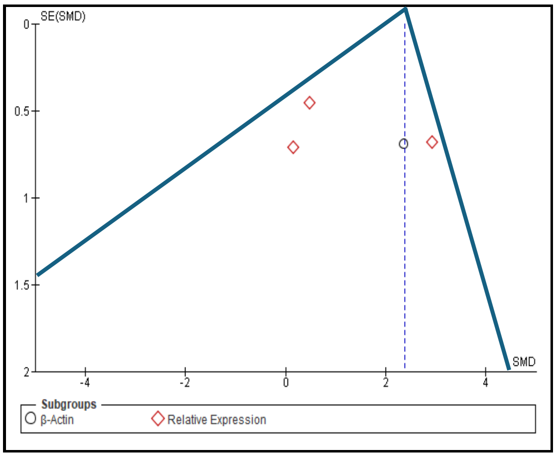

Fig. S2-16 TLR4

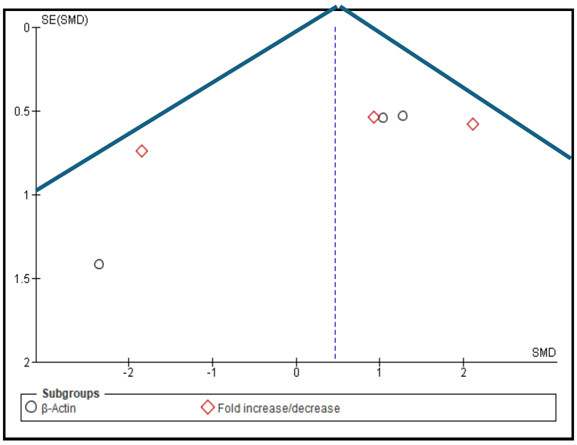

Fig. S2-17 p-ERK 1/ 2 /t-ERK 1/ 2

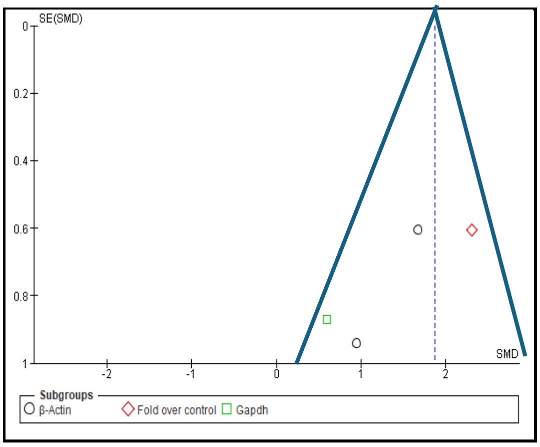

Fig. S2-18 p-JNK / t-JNK

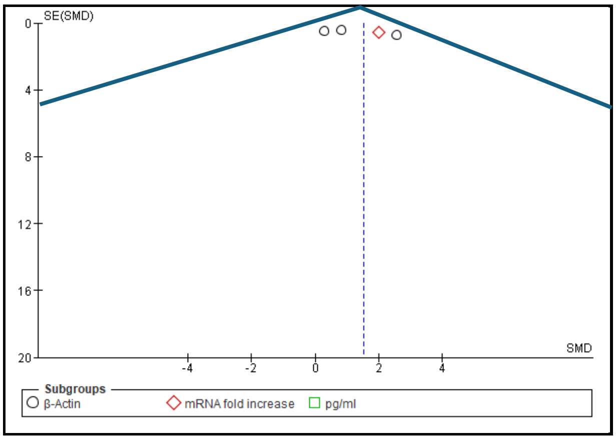

Fig. S2-19 TGF-β

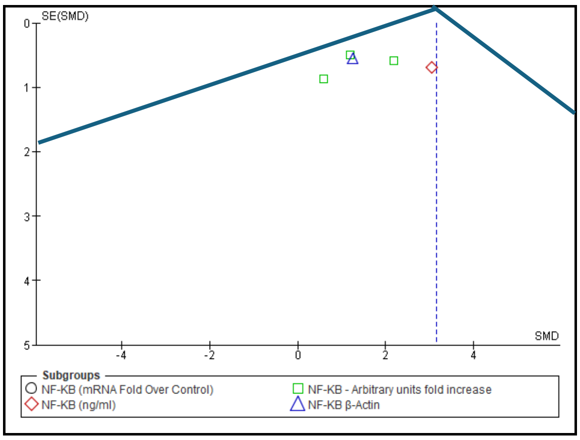

Fig. S2-20 NF-KB

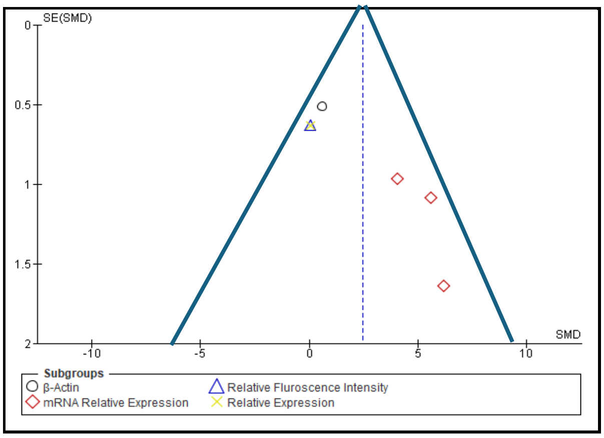

Fig. S2-21 Col III

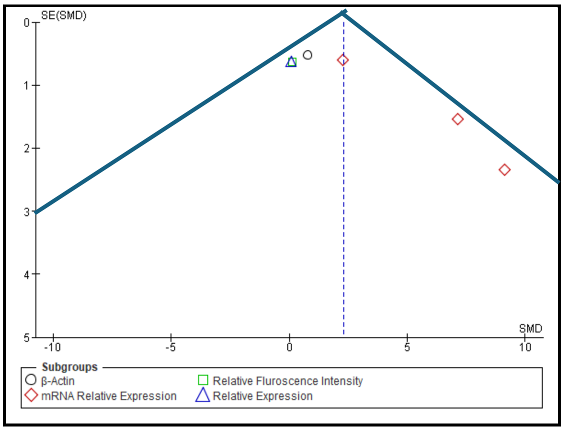

Fig. S2-22 Col I

Legend: Figures S2-1 to S2-22 includes the funnel plots of the biomarkers which had more than 5 included studies in the present meta-analysis. None of the biomarkers had a perfectly symmetrical funnel plot but most of the markers displayed some symmetry indicating minimal risk of bias in reporting bias. However, certain funnel plots did not show the exact number of the included studies either. Out of the total of 37 biomarkers tested, only 22 had included studies > 5. In most of the funnel plots, the studies which had large effect sizes or SMD were shown gathered at the top of the funnel whereas those with smaller effect sizes or SMD were present towards the bottom. There was more strength in the biomarkers having studies with larger effect sizes gathered at the top that were shown in 14 of the funnel plots displayed above.
