# Supplementary material for: Investigating the biomarkers of diabetic-cardiomyopathy with the high mobility group box-1 as a potential anti-inflammatory therapeutic target: Systematic Review and meta-analysis
Source: Front Endocrinol (Lausanne). 2026 Jan 14;16:1714219. doi: 10.3389/fendo.2025.1714219 (PMC12846985; doi:10.3389/fendo.2025.1714219)
Supplement: Supplementary file 3 [file DataSheet3.pdf]

SUPPLEMENTARY SECTION 3 – DATA TABLES OF THE BIOMARKER MODELS.

Table S3-1 (a) and (b): The summary statistics of the (a) forest plot and (b) non-parametric Mann Whitney U test of the Biomarker Model-1.

(a)

OUTCOME	#STUDIES	# Control Animals	# DCM Animals	SMD	95% CI	Z SCORE	*P*<0.05	TAU^2	I^2 %	CHI SQ	DF	*P*<0.05
AGEs	3	40	41	5.43	1.73, 9.13	2.88	0.004	12.29	93	61.25	4	0.00001
HMGB1	7	123	133	3.00	1.58, 4.42	4.15	0.0001	3.68	91	102.82	9	0.00001

(b)

OUTCOME	Mann Whitney U	(*P*<0.05)
AGEs	3	0.7000
HMGB1	0	0.0286

Table S3-2: The summary statistics of the (a) forest plot and (b) non-parametric Mann Whitney U test of the Biomarker Model-2.

Biomarker	# Studies	# Animals	SMD	95%CI	Z Score	P<0.05	Tau^2	I^2	Chi ^{2}	df	P<0.05	Mann Whitney U	P<0.05
HR	3	40	12.20	-1.59, 26.0	1.73	0.08	110.18	96	48.09	2	0.00001	2.5	0.5000
HW	4	65	4.73	-0.09, 9.37	2.0	0.05	20.45	95	57.44	3	0.00001	7	0.8857
HW/BW	7	89	2.31	0.63. 3.99	2.69	0.007	3.48	87	38.96	6	0.00001	3	0.0152
EF%	13	207	-4.13	-5.56, -2.69	5.65	0.0007	4.61	87	89.69	12	0.00001	20	0.0004
FS%	14	187	-2.70	-3.66, -1.74	5.53	0.00001	2.09	79	57.73	12	0.00001	28	0.0093
LVIDD	10	147	-0.09	-1.62, 1.44	0.11	0.91	4.08	90	68.88	7	0.00001	23	0.3823
LVIDS	9	121	1.64	0.04, 3.23	2.01	0.04	3.89	88	50.80	6	0.00001	16.50	0.3281
LVDV	1	44	4.93	3.35, 6.50	6.12	0.00001	NS	NS	NS	NS	NS	UC	UC
LVSV	4	68	1.38	-1.38, 4.13	0.98	0.33	5.49	93	27.96	3	0.00001	4	>0.9999
CK-MB	8	112	3.28	1.04, 5.52	2.87	0.0004	6.41	89	61.15	8	0.00001	13	0.4848

**CTPN**	**4**	**60**	**37.93**	**4.28, 71.58**	**2.21**	**0.03**	**1056.98**	**95**	**54.89**	**3**	**0.00001**	**6**	**0.6857**
**LDH**	**7**	**94**	**5.66**	**2.48, 8.84**	**3.49**	**0.0005**	**10.59**	**91**	**51.04**	**6**	**0.00001**	**19**	**0.5350**
**BP**	**3**	**56**	**1.71**	**-0.71, 4.13**	**1.39**	**0.17**	**4.08**	**92**	**24.30**	**2**	**0.0001**	**3**	**0.7000**

Table S3-3 (a) and (b): The summary statistics of the (a) forest plot and (b) non-parametric Mann Whitney U test of the Biomarker Model-3.

(a)

**OUTCOME**	**#STUDIES**	**#ANIMALS**	**SMD**	**95% CI**	**Z SCORE**	***P*<0.05**	**TAU^2**	**I^2 %**	**CHI SQ**	**DF**	***P*<0.05**
**BG**	**12**	**187**	**7.54**	**4.97, 10.12**	**5.74**	**0.00001**	**15.74**	**91**	**122.89**	**11**	**0.00001**
**INS**	**2**	**32**	**-9.46**	**-27.72, 8.80**	**1.02**	**0.31**	**162.42**	**92**	**14.55**	**1**	**0.0001**

BW	7	105	-1.74	-3.46, -0.02	1.98	0.05	4.07	88	51.71	6	0.00001

(b)

OUTCOME	# CONTROL ANIMALS	# DCM ANIMALS	Mann Whitney U	(P<0.05)
BG	94	93	9	0.0001
INS	16	16	NED	NED
BW	52	53	18	0.4557

Table S3-4 (a) and (b): The summary statistics of the (a) forest plot and (b) non-parametric Mann Whitney U test of the Biomarker Model-4.

(a)

OUTCOME	#STUDIES	#ANIMALS	SMD	95% CI	Z SCORE	P<0.05	TAU^2	I^2 %	CHI SQ	DF	P<0.05
TC	4	64	7.38	1.85, 12.92	2.61	0.009	16.98	92	37.08	3	0.00001
TG	5	76	16.60	7.01, 26.19	3.39	0.0007	86.89	94	61.67	4	0.00001
HDL	1	12	-5.84	-8.90, -2.77	3.73	0.0002	NS	NS	NS	NS	NS
LDL	1	12	39.70	20.29, 59.11	4.01	0.0001	NS	NS	NS	NS	NS

(b)

OUTCOME	# CONTROL ANIMALS	# DCM ANIMALS	Mann Whitney U	(P<0.05)
TC	32	32	0	0.0286
TG	38	38	3	0.0476
HDL	6	6	ND	ND
LDL	6	6	ND	ND

Table S3-5 (a) and (b): The summary statistics of the (a) forest plot and (b) non-parametric Mann Whitney U test of the Biomarker Model-5.

(a)

OUTCOME	#STUDIES	#ANIMALS	SMD	95% CI	Z SCORE	P<0.05	TAU^2	I^2 %	CHI SQ	DF	P<0.05
GSH	4	58	-4.60	-8.04, -1.16	2.62	0.009	10	90	29.44	3	0.00001
MDA	7	90	4.78	2.71, 6.86	4.52	0.00001	5.22	79	28.10	6	0.0001

(b)

OUTCOME	# CONTROL ANIMALS	# DCM ANIMALS	Mann Whitney U	(P<0.05)
GSH	29	29	5	0.4857
MDA	45	45	15	0.2593

Table S3-6 (a) and (b): The summary statistics of the (a) forest plot and (b) non-parametric Mann Whitney U test of the Biomarker Model-6.

(a)

OUTCOME	#STUDIES	#ANIMALS	SMD	95% CI	Z SCORE	*P*<0.05	TAU^2	I^2 %	CHI SQ	DF	*P*<0.05
TNF-A	7	132	1.11	0.11, 2.11	2.17	0.03	1.31	82	33.10	6	0.0001
IL-6	9	186	5.49	3.35, 7.64	5.01	0.00001	9.50	92	126.82	10	0.00001
IL-1β	5	92	4.44	1.56, 7.32	3.02	0.003	8.11	93	55.03	4	0.00001

(b)

OUTCOME	# CONTROL ANIMALS	# DCM ANIMALS	Mann Whitney U	(*P*<0.05)
TNF-A	66	66	6	0.6757
IL-6	93	93	10	0.2229
IL-1β	46	46	1	0.6667

Table S3-7 (a) and (b): The summary statistics of the (a) forest plot and (b) non-parametric Mann Whitney U test of the Biomarker Model-7.

(a)

OUTCOME	#STUDIES	#ANIMALS	SMD	95% CI	Z SCORE	*P*<0.05	TAU^2	I^2 %	CHI SQ	DF	*P*<0.05
NF-kB	5	122	3.13	1.52, 4.74	3.80	0.0001	3.71	88	48.12	6	0.00001
TLR4	5	84	2.38	0.61, 4.15	2.63	0.009	3.45	88	32.96	4	0.00001
CLEAVED CASPASE 3	4	76	3.91	1.46, 6.36	3.13	0.002	5.73	89	36.29	4	0.00001
NLRP3	4	68	3.59	0.28, 6.89	2.13	0.03	10.26	94	47.41	3	0.00001
pERK1/2 / t-ERK1/2 RATIO	6	88	0.46	-0.67, 1.59	0.80	0.42	1.49	79	23.85	5	0.0002
pJNK/t-JNK RATIO	5	66	1.87	0.95, 2.79	3.99	0.0001	0.53	49	7.85	4	0.10
TGF-β	5	84	1.50	-0.03, 3.03	1.92	0.05	2.12	84	24.96	4	0.0001

(b)

OUTCOME	# CONTROL ANIMALS	# DCM ANIMALS	Mann Whitney U	(*P*<0.05)
NF-kB	61	61	0	0.0286
TLR4	42	42	3	0.2000
CLEAVED CASPASE 3	38	38	NED	NED
NLRP3	34	34	NED	NED
pERK1/2 /t-ERK1/2 RATIO	44	44	3.5	0.8000
pJNK/t-JNK RATIO	33	33	1.5	0.3000
TGF-β	42	42	1	0.2000

Table S3-8 (a) and (b): The summary statistics of the (a) forest plot and (b) non-parametric Mann Whitney U test of the Biomarker Model-8.

(a)

OUTCOME	#STUDIES	#ANIMALS	SMD	95% CI	Z SCORE	*P*<0.05	TAU^2	I^2 %	CHI SQ	DF	*P*<0.05
FB%	4	64	3.25	0.77, 5.74	2.57	0.01	5.41	89	26.47	3	0.00001
Collagen I	6	84	2.31	0.62, 3.99	2.68	0.007	3.39	86	36.29	5	0.00001
Collagen III	6	84	2.43	0.59, 4.27	2.59	0.010	4.41	88	42.48	5	0.00001

(b)

OUTCOME	# CONTROL ANIMALS	# DCM ANIMALS	Mann Whitney U	(*P*<0.05)
FB%	32	32	1	0.0571
Collagen I	42	42	0	0.1000
Collagen III	42	42	0	0.1000

Legend: The summary statistics of the 37 biomarkers belonging to the 8 biomarker models are tabulated to give the spread of the data and the statistical parameters obtained from the forest plots and the non-parametric Mann Whitney U test. The forest plots calculate the SMD or the effect size and the 95% confidence interval and the statistics of heterogeneity. The forest plots evaluate the biomarkers as the primary outcomes, and the Mann Whitney U test calculates the secondary outcomes between the healthy control animals and the animals induced with DCM and statistical validity of the data which are relevant to this study. Out of the total of 37 biomarkers, significant differences occurred in only 8 biomarkers at $p < 0.05$ in the secondary outcomes which included HMGB1, EF%, FS%, HW/BW, BG, TC, TG, and NF-KB. The assumptions made include the data not showing conformity with a normal distribution in the non-parametric Mann Whitney U test being independent samples having smaller sample size.
