# Supplementary material for: Investigating the biomarkers of diabetic-cardiomyopathy with the high mobility group box-1 as a potential anti-inflammatory therapeutic target: Systematic Review and meta-analysis
Source: Front Endocrinol (Lausanne). 2026 Jan 14;16:1714219. doi: 10.3389/fendo.2025.1714219 (PMC12846985; doi:10.3389/fendo.2025.1714219)
Supplement: Supplementary file 4 [file DataSheet4.pdf]

SUPPLEMENTARY SECTION -4 -RECEIVER OPERATOR CURVES (ROCs) OF THE BIOMARKERS.

Table S4-1

FIGURE	Biomarker	AUC	95% CI	SEM	P<0.05	Control #	DCM #
S4-1	AGEs	0.7778	0.3545 to 1.000	0.2160	0.2752	3	3
S4-2	HMGB1	1.000	1.000 to 1.000	0.000	0.0209*	4	4
S4-3	HR	0.7222	0.2448 to 1.000	0.2436	0.3827	3	3
S4-4	HW	0.5625	0.1283 to 0.9967	0.2215	0.7728	4	4
S4-5	HW/BW	0.9167	0.7424 to 1.000	0.08893	0.0163*	6	6
S4-6	EF%	0.8225	0.6594 to 0.9856	0.08322	0.0052*	13	13
S4-7	FS%	0.8056	0.6176 to 0.9935	0.09588	0.0111*	12	12
S4-8	LVIDD	0.6406	0.3505 to 0.9308	0.1480	0.3446	8	8
S4-9	LVIDS	0.6633	0.3587 to 0.9679	0.1554	0.3067	7	7
S4-10	LVSV	0.5556	0.03209 to 1.000	0.2671	0.8273	3	3
S4-11	CK-MB	0.6389	0.3106 to 0.9672	0.1675	0.4233	6	6
S4-12	CTPN	0.6875	0.2894 to 1.000	0.2031	0.3865	4	4
S4-13	LDH	0.6122	0.3059 to 0.9186	0.1563	0.4822	7	7
S4-14	BP	0.6667	0.1332 to 1.000	0.2722	0.5127	3	3

S4-15	BG	1.000	1.000 to 1.000	0.000	<0.0001*	12	12
S4-16	SINS	0.7500	0.2022 to 1.000	0.2795	0.4386	2	2
S4-17	BW	0.6327	0.3000 to 0.9654	0.1697	0.4062	7	7
S4-18	TC	1.000	1.000 to 1.000	0.000	0.0209*	4	4
S4-19	TG	0.8800	0.6401 to 1.000	0.1224	0.0472*	5	5
S4-20	GSH	0.6875	0.2894 to 1.000	0.2031	0.3865	4	4
S4-21	MDA	0.6939	0.4074 to 0.9804	0.1462	0.2248	7	7
S4-22	TNF-A	0.6250	0.2141 to 1.000	0.2096	0.5637	4	4
S4-23	IL-6	0.8000	0.4943 to 1.000	0.1559	0.1172	5	5
S4-24	NF-kB	0.9375	0.7616 to 1.000	0.08976	0.0433*	4	4
S4-25	TLR4	0.8125	0.4647 to 1.000	0.1775	0.1489	4	4
S4-26	pERK1 /2/ t-ERK 1/2	0.6111	0.1174 to 1.000	0.2519	0.6625	3	3
S4-27	pJNK / t-JNK	0.8333	0.4759 to 1.000	0.1824	0.1904	3	3
S4-28	TGF-β	0.8889	0.5985 to 1.000	0.1481	0.1266	3	3
S4-29	Fibrosis %	0.9375	0.7616 to 1.000	0.08976	0.0433*	4	4
S4-30	Col I	1.000	1.000 to 1.000	0.0000	0.0039*	3	3
S4-31	Col III	1.000	1.000 to 1.000	0.0000	0.0495*	3	3

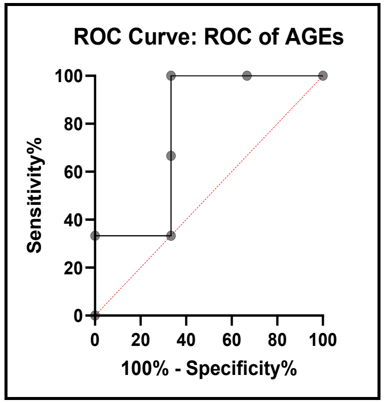

Fig.S4-1

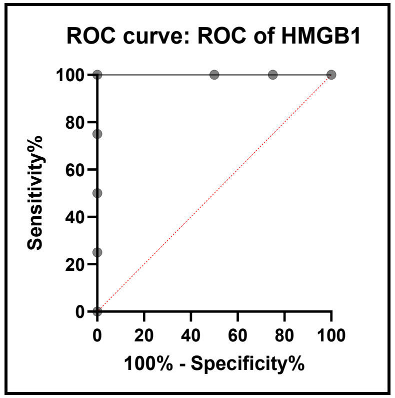

Fig.S4-2

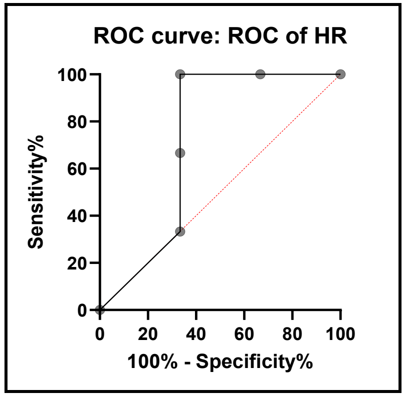

Fig.S4-3

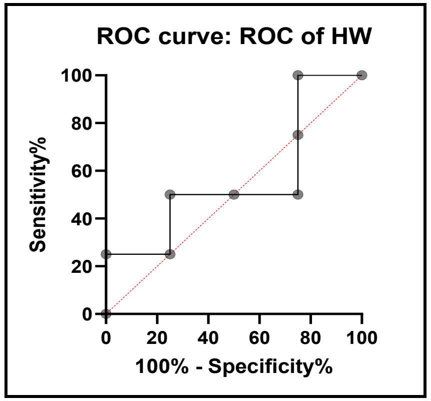

Fig.S4-4

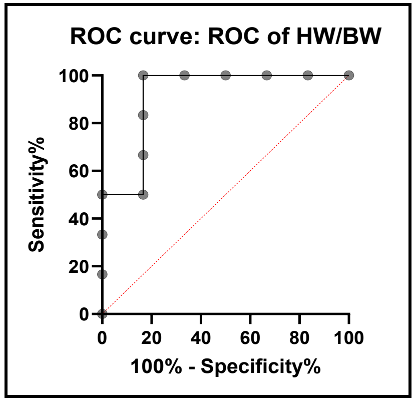

Fig.S4-5

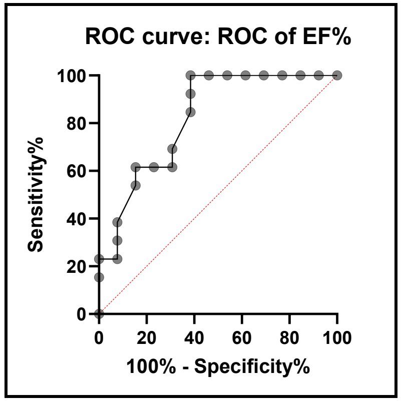

Fig.S4-6

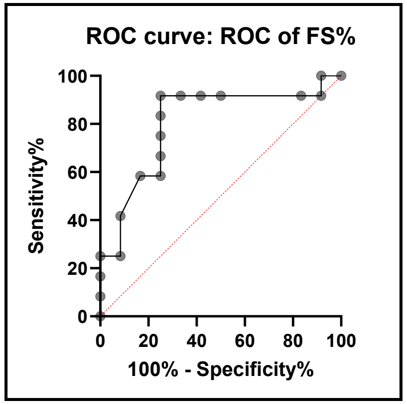

Fig.S4-7

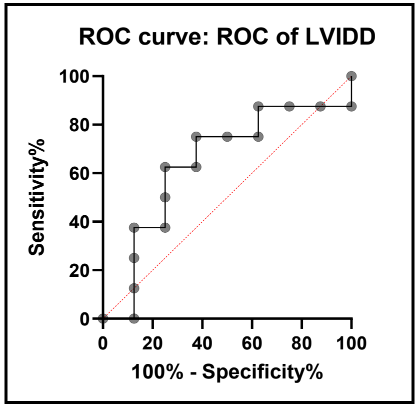

Fig.S4-8

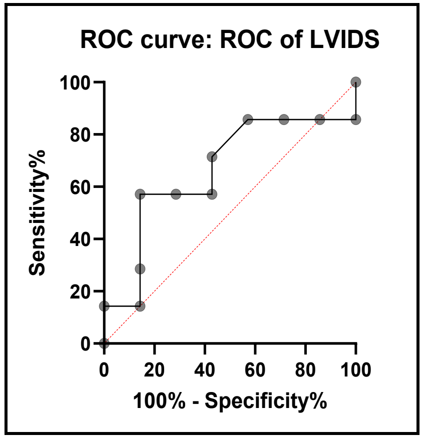

Fig.S4-9

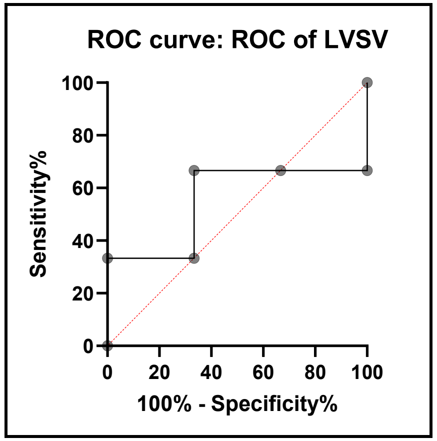

Fig.S4-10

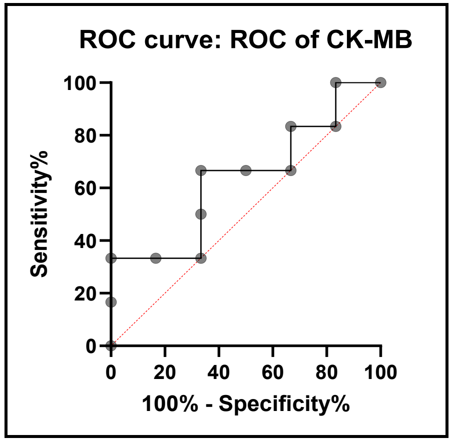

Fig.S4-11

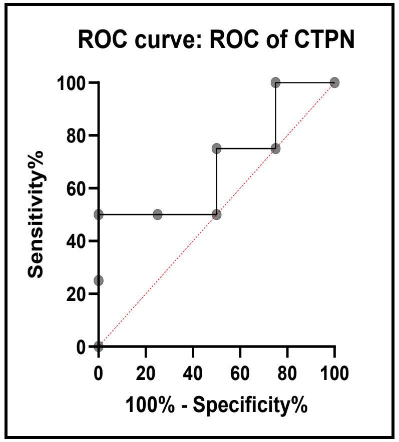

Fig.S4-12

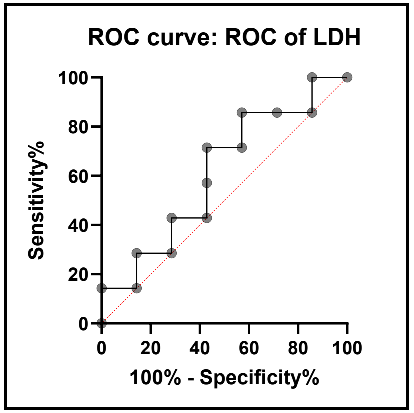

Fig.S4-13

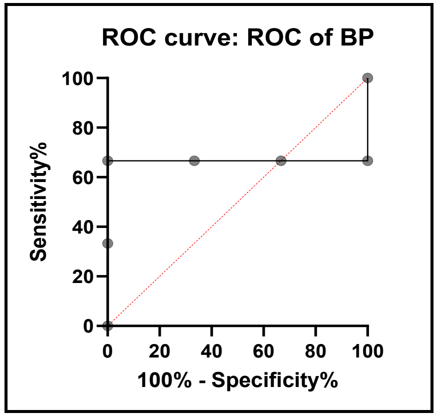

Fig.S4-14

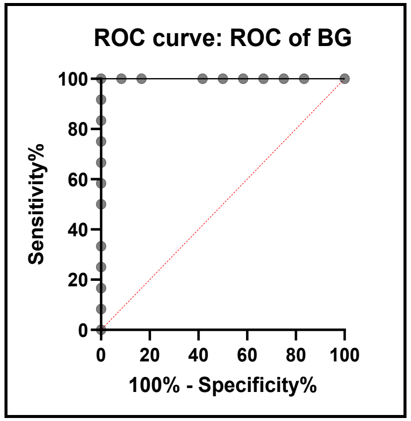

Fig.S4-15

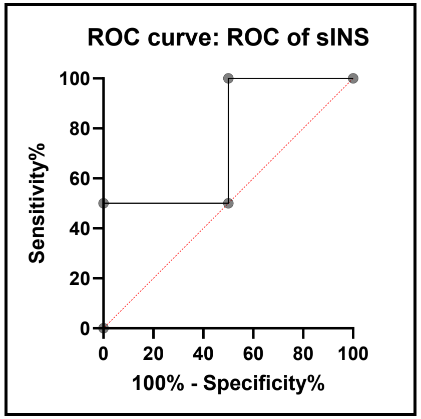

Fig.S4-16

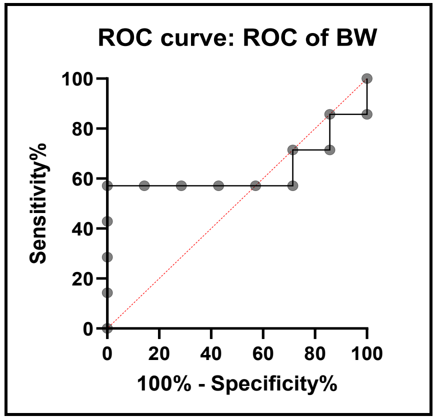

Fig.S4-17

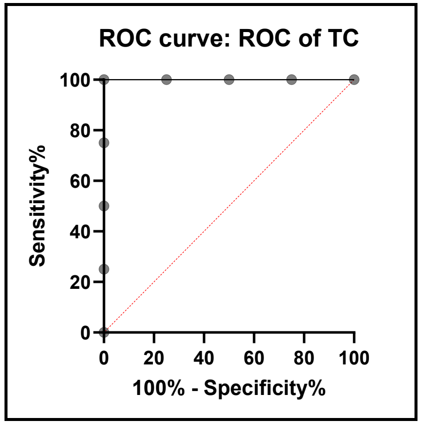

Fig.S4-18

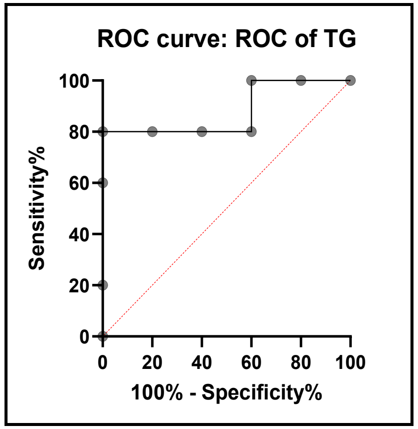

Fig.S4-19

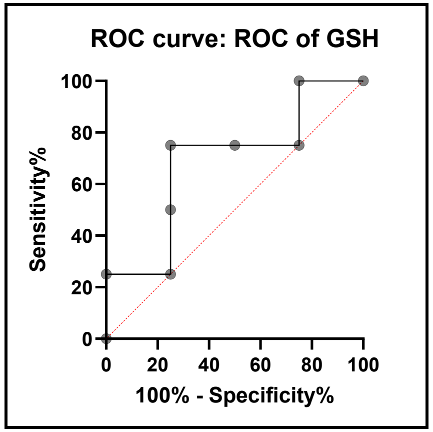

Fig.S4-20

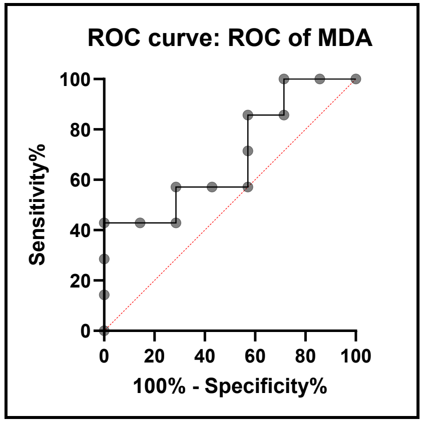

Fig.S4-21

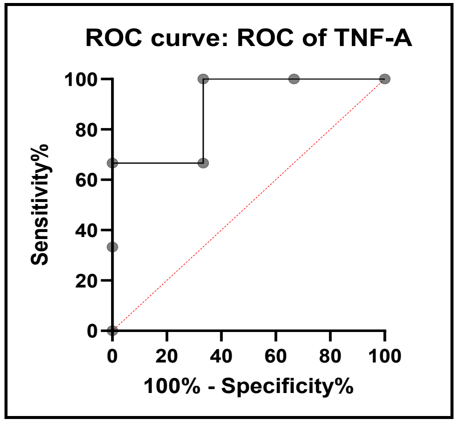

Fig.S4-22

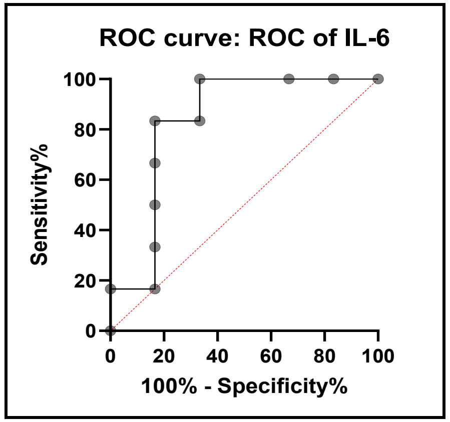

Fig.S4-23

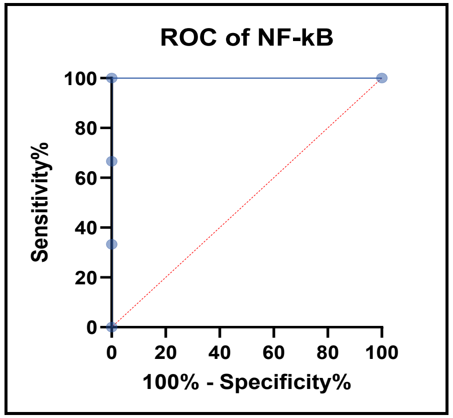

Fig.S4-24

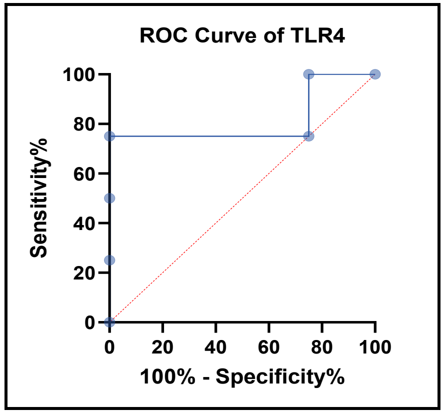

Fig.S4-25

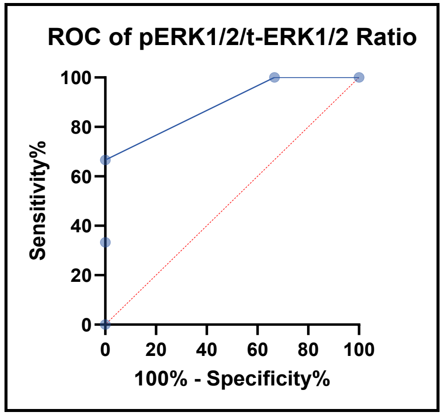

Fig.S4-26

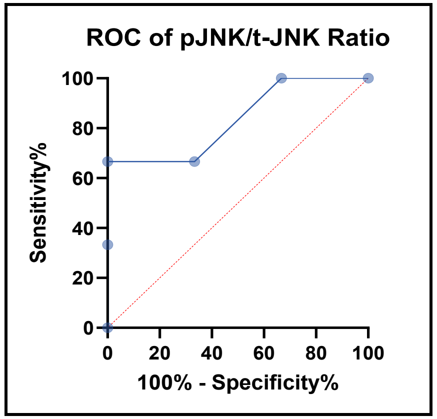

Fig.S4-27

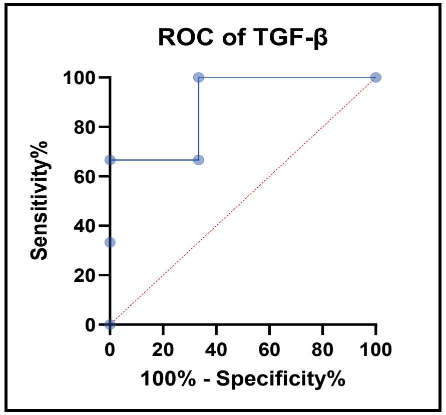

Fig.S4-28

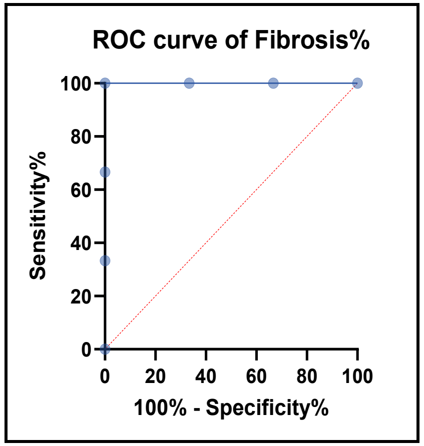

Fig.S4-29

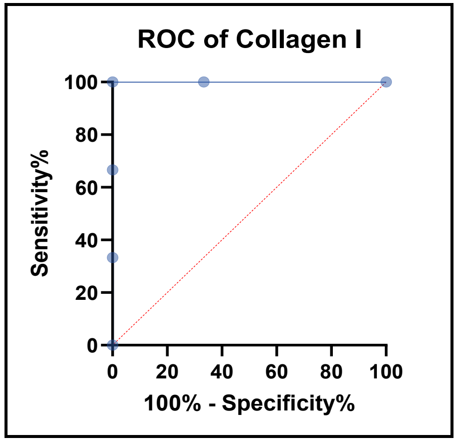

Fig.S4-30

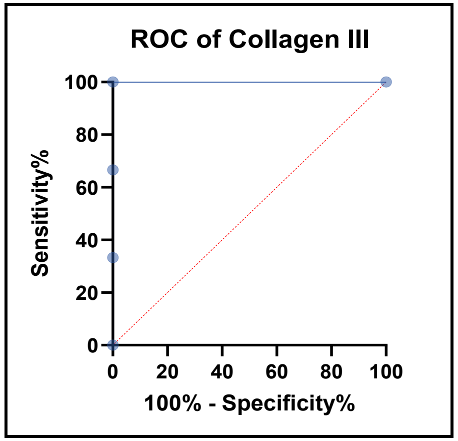

Fig.S4-31

Legend: Figure S4-1 to S4-31 displays the receiver operator curves of the biomarkers which had 3 or more than 3 included studies. Out of the 37 biomarkers, 31 ROC curves were constructed and there were no outliers in 25 biomarkers whereas HW, LVIDD, LVIDS, LVSV, BP, BW showed outliers. Table S4-1 gives the area under the curve, the 95% confidence intervals, the standard error of the mean and the probability at <0.05. The ROC of eleven biomarkers showed significant differences in the ROC curves which included the biomarkers HMGB1, HW/BW, EF%, FS%, BG, TC, TG, NF-KB, Fibrosis %, Col I and Col III.
