# Supplementary material for: Investigating the biomarkers of diabetic-cardiomyopathy with the high mobility group box-1 as a potential anti-inflammatory therapeutic target: Systematic Review and meta-analysis
Source: Front Endocrinol (Lausanne). 2026 Jan 14;16:1714219. doi: 10.3389/fendo.2025.1714219 (PMC12846985; doi:10.3389/fendo.2025.1714219)
Supplement: Supplementary file 5 [file DataSheet5.pdf]

PRISMA 2020 CHECKLIST

**TITLE**	1	**Investigating the biomarkers of diabetic-cardiomyopathy with the high mobility group box-1 as a potential anti-inflammatory therapeutic target: Systematic Review and Meta-Analysis.**	Pg<br>1
**ABSTRACT**	2	**PRISMA 2020 Abstracts checklist**	Pg

**SECTION AND TOPIC**	**ITEM #**	**CHECKLIST ITEM**	**ABSTRACT**	Pg
TITLE	1	Identify the report as a systematic review.	**Investigating the biomarkers of diabetic-cardiomyopathy with the high mobility group box-1 as a potential anti-inflammatory therapeutic target: Systematic Review and Meta-Analysis.**	1
**BACKGROUND**				
OBJECTIVES	2	Provide an explicit statement of the main objective(s) or question(s) the review addresses.	The aims of this study included identifying and assessing the prominent risk factors/biomarkers of Diabetic Cardiomyopathy (DCM) and evaluating the impact of the HMGB1 nuclear protein as a prospective anti-inflammatory therapeutic option on those risk factors.	3-4
**METHODS**				
ELIGIBILITY CRITERIA	3	Specify the inclusion and exclusion criteria for the review.	The healthy control consisted of healthy mice and rats comprising of the rodent model utilised in the present study. They were compared with streptozotocin -induced diabetic age -matched male counterparts diagnosed with DM-induced cardiomyopathy in published research studies from year 2010 to 2024 were included. Reviews, not DCM, clinical studies and rodents other than mouse and rat were excluded.	

			INFORMATION SOURCES	4	Specify the information sources (e.g. databases, registers) used to identify studies and the date when each was last searched.	PUBMED, Science Direct, Scopus, Springer-Nature and Wiley Online Library databases were searched from 1/1/2010 to 1/12/2024.		
			RISK OF BIAS	5	Specify the methods used to assess risk of bias in the included studies.	The risk of bias was assessed with the Cochrane (SYRCLE) tool, which is relevant to pre-clinical subjects.		
			SYNTHESIS OF RESULTS	6	Specify the methods used to present and synthesize results.	A random effects model for continuous data was followed and analysed by RevMan 5.4 and GraphPad Prism 10 software.		
			**RESULTS**					
			INCLUDED STUDIES	7	Give the total number of included studies and participants and summarise relevant characteristics of studies.	29 relevant case-control studies having 2979 rodents were included. Study characteristics are summarised in Table 1.		9
			SYNTHESIS OF RESULTS	8	Present results for main outcomes, preferably indicating the number of included studies and participants for each. If meta-analysis was done, report the summary estimate and confidence/credible interval. If comparing groups, indicate the direction of the effect (i.e. which group is favoured).	37 risk factors/biomarkers were identified from 29 included studies and were grouped into 8 primary outcome models, that favoured DCM synonymous with a high risk of CVD. (see table below)		S1 9

**Risk Factor**	**# Included studies**	**# of Animals**	**Effect estimate**	**95% CI**	**P<0.05**
**AGEs**	**3**	**81**	**5.43**	**1.73, 9.13**	**0.0002**
**HMGB1**	**11**	**256**	**3.00**	**1.58, 4.42**	**0.0001**
**HR**	**3**	**40**	**12.20**	**-1.59, 26.00**	**0.08**
**HW**	**4**	**45**	**4.73**	**0.09, 9.37**	**0.05**
**HW/BW**	**6**	**89**	**2.31**	**0.63, 3.99**	**0.007**
**EF%**	**13**	**207**	**-4.13**	**-5.56, -2.69**	**0.00001**
**FS%**	**12**	**187**	**-2.91**	**-3.96, -1.86**	**0.00001**

					**LVIDD**	**8**	**119**	**-0.09**	**-1.62, 1.44**	**0.91**
					**LVIDS**	**7**	**93**	**1.64**	**0.04, 3.23**	**0.04**
					**LVSV**	**3**	**52**	**1.38**	**-1.38, 4.13**	**0.33**
					**CK-MB**	**8**	**112**	**3.28**	**1.04, 5.52**	**0.004**
					**CTN**	**4**	**60**	**37.93**	**4.28, 71.58**	**0.03**
					**LDH**	**7**	**94**	**5.66**	**2.48, 8.84**	**0.0005**
					**BP**	**3**	**56**	**1.71**	**-0.71, 4.13**	**0.17**
					**BG**	**12**	**187**	**7.54**	**4.97, 10.12**	**0.00001**
					**SINS**	**2**	**32**	**-9.46**	**-27.72, 8.80**	**0.31**
					**BW**	**7**	**105**	**-1.74**	**-3.46, -0.02**	**0.05**
					**TC**	**4**	**64**	**7.38**	**1.85, 12.92**	**0.009**
					**TG**	**5**	**76**	**16.60**	**7.01, 26.19**	**0.0007**
					**GSH**	**4**	**58**	**-4.60**	**-8.04, -1.16**	**0.009**
					**MDA**	**7**	**90**	**4.78**	**2.71, 6.86**	**0.00001**
					**TNF-A**	**7**	**132**	**1.11**	**0.11, 2.11**	**0.03**
					**IL-6**	**9**	**186**	**5.49**	**3.35, 7.64**	**0.00001**
					**IL-1β**	**5**	**92**	**4.44**	**1.56, 7.32**	**0.003**
					**NF-kB**	**5**	**122**	**3.13**	**1.52, 4.74**	**0.00001**
					**TLR4**	**5**	**84**	**2.38**	**-0.61, 4.15**	**0.009**
					**Cleaved CASPASE 3**	**4**	**76**	**3.91**	**1.46, 6.36**	**0.002**

			**NLRP3** | **4** | **68** | **3.59** | **0.28, 6.89** | **0.03**
			**pERK1 /2/t-ERK 1 /2** | **5** | **88** | **0.46** | **0.67, 1.59** | **0.42**
			**pJNK/ t-JNK** | **5** | **66** | **1.87** | **0.95, 2.79** | **0.0001**
			**TGF-β** | **5** | **84** | **1.50** | **-0.03, 3.03** | **0.05**
			**FB%** | **4** | **64** | **3.25** | **0.77, 5.74** | **0.01**
			**Collagen I** | **6** | **84** | **2.31** | **0.62, 3.99** | **0.007**
			**Collagen III** | **6** | **84** | **2.43** | **0.59, 4.27** | **0.01**
**DISCUSSION**			
LIMITATIONS OF EVIDENCE	9	Provide a brief summary of the limitations of the evidence included in the review (e.g. study risk of bias, inconsistency, and imprecision).	The main limitation is having a few studies for certain risk factors of importance. However, the minimum participant number was 44 and the maximum participants included was more than 240.
INTERPRETATION	10	Provide a general interpretation of the results and important implications.	Our findings have strong implications for improving the quality of life and health economics related to vascular disease in DCM. A significant positive correlation between HMGB1 and NF-kB underpins the importance of suppressing inflammation by way of inhibiting the HMGB1 activity either in the nuclear or extracellular milieu which could be the key to resolving DCM and warrants further investigations.
**OTHER**			
FUNDING	11	Specify the primary source of funding for the review	No funding was received.
REGISTRATION	12	Provide the register name and registration number.	This study was registered at the PROSPERO register in October 2024 and obtained CRD42024597641.

INTRODUCTION			Pg
RATIONALE	3	DCM is a group of cardiometabolic diseases which is common in the community having a 1.1% prevalence but a 31% high morbidity and mortality. The significance of DCM is premature heart failure, which occurs in 30 to 40% of individuals while for most of the time, patients remain asymptomatic until the condition is worsened. Cardiomyopathy can manifest in any age group or gender. The DCM patients develop heart failure and death up to 55.9% in the first 5 years of a diagnosis in DCM. For those having had diabetes for 15 years, the likelihood of developing heart failure and its mortality rate is 65.8% . The Australian men diagnosed with DCM are more prone to developing DCM than their female counterparts. It is because of the existing lack of consensus in defining criteria for DCM and having a poor prognosis. The total healthcare costs of CVD were projected to exceed Australian dollars (AUD) 61.89 (61.79–88.66) billion, and productivity losses will account for AUD 78.75 (49.40–295.25) billion, driving the total cost to surpass AUD 140.65 (123.13–370.23) billion. Considering all these facts, there remains an urgent need for producing effective diagnostic criteria and treatment strategies put in place for which defining the risk factors and identifying HMGB1 as a possible therapeutic target form the basis for undertaking this study. This study aims to evaluate the relationship between DCM and the molecular signalling pathways that involve HMGB1 in a preclinical rodent model which combined both rats and mice induced with diabetes that had progressed further to DCM. These 30 biomarkers are evaluated in a systematic review and meta-analysis to assess their therapeutic potential as an anti-inflammatory treatment modality that is pre-tested in the rodents.	4 To 6
OBJECTIVES	4	• To identify and assess the prominent risk factors of *Diabetes mellitus* -induced cardiomyopathy.<br>• To investigate the influence and impact of the HMGB1 nuclear protein on the aforesaid risk factors.<br>• To ascertain whether HMGB1 could be used as a potential anti-inflammatory option in attenuating the inflammation associated with DCM in the rodents.	6

METHODS			
ELIGIBILITY CRITERIA	5	1. Be mouse and rat models<br>2. Be of around 8 weeks of age<br>3. Induced with diabetes/hyperglycaemia<br>4. With or without HMGB1 as a risk factor<br>5. Preclinical research studies<br>6. Case-control studies that compared rodents induced with DCM with an age - matched healthy cohort.<br>7. Reporting data for AGEs, cardiometabolic, glycaemic, lipid, inflammatory, oxidative stress, signalling mechanism and /or fibrotic biomarkers.<br>8. Published in English<br>9. Published since year 2010 till end of 2024	
INFORMATION SOURCES	6	Records identified from Databases<br>• PubMed *(n*= 1840)<br>• Science Direct (*n*=569)<br>• Scopus (n=26)<br>• Springer link (n= 63)<br>• Wiley Online library (n=481)<br><br>Total studies retrieved =2979<br>Eligible studies =29<br>Rejected Studies = 2950	6
SEARCH STRATEGY	7	The search strategy consisted of searching the databases with keywords after specifying the year range. The databases were accessed *via* the Victoria University's library resources webpage. The keywords consisted of:<br>• Diabetic cardiomyopathy AND mouse AND /OR rat AND HMGB1<br>• Diabetes AND cardiomyopathy AND mouse AND high mobility group box-1<br>• T2D AND rodent model AND HMGB1<br>• Diabetes mellitus AND cardiomyopathy AND mouse OR rat AND hmgb1<br>• Myocardial fibrosis AND mouse AND rat AND HMGB1<br>• HMGB1 AND type-2 diabetes AND mouse AND rat AND heart dysfunction/fibrosis<br>• Type-1 diabetes AND hmgb1 AND rat AND mouse<br>• T2D AND T1D AND RAT AND MOUSE AND HMGB1<br>• Myocardial infarction/reperfusion injury AND mouse /Rat AND hmgb1	6

SELECTION PROCESS	8	The first author conducted a thorough review of the full text articles identified in the database searches and extracted the data, under the guidance of an expert as per the pre-specified inclusion criteria defined. The basic characteristics of the included studies are defined in Table 1, S2, S3.	
DATA COLLECTION PROCESS	9	A standardized, pre-piloted form (data extraction spreadsheets) was used to extract data from the included studies for assessment of study quality and evidence synthesis. All included studies were screened by the first author under the guidance of an expert according to the criteria suggested in the Cochrane guidelines as outlined in the Cochrane Handbook for Systematic Reviews of pre-clinical disease subjects. The extracted information included baseline characteristics (age, gender, body weight, number of participants by gender, year of publication, first author's name, country of publication, how diabetes was induced), details of the disease (disease duration) control conditions, risk factor metrics, and for each primary outcome, the mean, standard deviation (SD), and the participant number (n) were recorded for both the control and intervention/disease groups. (Table 1, S2, S3)	
DATA ITEMS	10a	There are 8 primary outcome models that were evaluated with secondary outcomes. They comprised of 37 biomarkers grouped into: (1) Advanced glycation end products (AGEs) consisting of AGEs and high mobility group box 1 (HMGB1), (2) Cardiometabolic consisting of the heart rate, heart weight, heart weight to body weight ratio, ejection fraction percentage, fractional shortening percentage, left ventricular internal diameter at diastole, left ventricular internal diameter at systole, left ventricular diastolic volume, left ventricular systolic volume, creatinine kinase in brain and muscle, cardiac troponin, lactate dehydrogenase and blood pressure (3) Glycaemic biomarkers consisting of blood glucose, serum insulin and body weight, (4) Lipid biomarkers consisting of total cholesterol, total triglycerides, high-density lipoproteins and low-density lipoproteins, (5) Oxidative Stress biomarkers consisting of glutathione and malondialdehyde, (6) Inflammatory biomarkers consisting of tumour necrosis factor - alpha, interleukin-six and interleukin - one beta, (7) Signalling pathway biomolecules consisting of nuclear factor kappa beta, toll-like receptor 4, NLRP3, cleaved caspase 3, phosphorylated ERK 1 /2 to total ERK 1/ 2 ratio, phosphorylated JNK to total JNK ratio and transforming growth factor beta, (8) Fibrosis -related biomarkers consisting of fibrosis percentage in heart tissue, Collagen I and Collagen III in heart tissue. The secondary outcomes were computed using the non-parametric Mann Whitney U test by computing the U statistic and the probabilities.	22 - 25
	10b	The data gathered also included the year of publication, the name of the first author, the number of healthy control and STZ-induced DCM animals consisting of mouse and rat, the gender, the mean age of the rodents in weeks, the body weight in grams, the limitations, how STZ was administered, the buffer solution used as the vehicle. Missing data was requested from the authors up to two times. If there was no response from the authors, the study was excluded from the analysis. The first author extracted the data under the guidance of an expert, and any discrepancy identified was resolved through discussion with him where and when necessary.	

STUDY RISK OF BIAS ASSESSMENT	11	This author carried out the ROB evaluation and arrived at a conclusion under the guidance of an expert in this field. In the meta-analysis, a summary of the overall ROB items is depicted in a risk stratified summary ROB graph. The quality of the body of evidence was maintained with the PRISMA 2020 guidelines, published by the Cochran collaboration, given in the handbook. The reporting biases can originate in numerous ways such as multiple publications appearing as duplicates, not being included in a given database, missing out a rapid or delayed publication, not being included in a citation list as well as reporting of selective outcomes only. There are also publications with limited access, publications in different languages other than English and unpublished relevant research items. All these reasons constitute of reporting bias which could overtly influence the effect size of a given primary outcome. The reporting bias tends to produce overly optimistic outcomes from the intervention effects. Our search strategy was designed to comprehensively mitigate those flaws associated with reporting bias and for this, funnel plots and the ROC for each biomarker was carried out.	16
EFFECT MEASURES	12	Since all the evaluated primary outcomes consisted of continuous data, a standardized mean difference (SMD) was calculated.	
SYNTHESIS METHODS	13a	The first author extracted the data under the guidance of an expert and any discrepancy identified was resolved through discussion with him when necessary. Data was tabulated in an excel spreadsheet that compared the healthy control group with the DCM group that met the eligibility criteria.	
	13b	The results were synthesized using both a narrative approach and quantitative meta-analysis that was presented in the results and interpreted in the discussion sections. The data was converted to one unit when the data was reported in different units. Different units of analysis were included if the minimum individual participant number exceeded 8.	
	13c	The methods used to display the results of individual studies were the forest plot, bar graphs of the non-parametric Mann Whitney U test, ROC, heatmap, computation of the Pearson's and Spearman's correlation coefficient and the coefficient of determination, significant at $p<0.05$.	S1
	13d	The 30 primary outcomes were grouped into 8 models and analysed in a meta-analysis between the control and treatment/intervention groups to identify which model comprised of the strongest risk factors for developing DCM. Statistical heterogeneity was evaluated by the I squared metric. The RevMan 5.4 version software was used for the data synthesis and to construct the forest plots. Assessing heterogeneity is an important step in meta-analysis and can be done using various methods, such as visual inspection of forest plots, and the I-squared statistic (I^2). Forest plot is a graphical representation of the effect sizes and confidence intervals of each included study in the meta-analysis. If there is heterogeneity, the individual study effect sizes will be spread out and not clustered around a central point. I^2 metric is another measure of the proportion of variability in effect sizes across studies due to heterogeneity. I^2 value between 0% to 25% indicates low heterogeneity, while a value greater than 50% suggests high heterogeneity.	25

	13e	Subgroup analysis is important when there is significant heterogeneity in the overall results and was used to investigate whether the impact of DCM varies across different subgroups of studies based on intervention characteristics or outcomes. Subgroup analysis was only conducted when there are more than 8 participants in any set of given studies where different units of measurement have been used. The random-effects model is often preferred over the fixed-effects model in the presence of heterogeneity, as it considers the variability in effect sizes across studies, providing a more cautious estimate of the true effect size.	19
	13f	Sensitivity analysis is carried out to prove that the statistical plots, particularly the forest plots are flexible and can be updated. It demonstrates that the forest plot is sensitive to any change in its values and it is not merely a static plot but a dynamic entity. An example is given using the forest plot that is constructed for the HMGB1 biomarker in this study (Fig Xa). In its last subgroup the 95% CI include 0.00 which when deleted, gives an entirely different value (Fig X b).	25
REPORTING BIAS ASSESSMENT	14	Assessing publication bias involved examining whether there is a systematic difference between published studies and unpublished studies that have the same research question. One commonly used method for assessing publication bias is by using funnel plots, which visually display the relationship between effect size estimates and study precision. To interpret this plot: studies with larger sample sizes will have smaller standard errors and will be plotted near the top of the graph, while studies with smaller sample sizes will have larger standard errors and will be plotted near the bottom of the graph. If there is no publication bias, the studies will be evenly distributed around the overall effect size estimate, forming a symmetrical funnel shape, while if there is publication bias, studies with larger effect sizes or smaller standard errors may be more likely to be published, resulting in an asymmetrical funnel plot.	S2
CERTAINITY ASSESSMENT	15	Certainty assessment was carried out by compiling the Q statistics to confirm there were no outliers, and the ROC that calculates the area under the curve.	
**RESULTS**			
STUDY SELECTION	16a	Records identified from Databases<br>• PubMed (n= 1840)<br>• Science Direct (n=569)<br>• Scopus (n=26)<br>• Springer link (n= 63)	7

- Wiley Online library (n=481)

Total studies retrieved =2979

Eligible studies =29 7

Rejected Studies = 2950 included duplicates, reviews, book chapters, conference papers and posters, not rodent studies, published before year 2010, not in English, only *in vitro* studies, no healthy control, cross sectional studies, authors not responded, and excluded with other reasons such as not reporting the biomarkers that were selected or having less than 8 animals for a given biomarker.

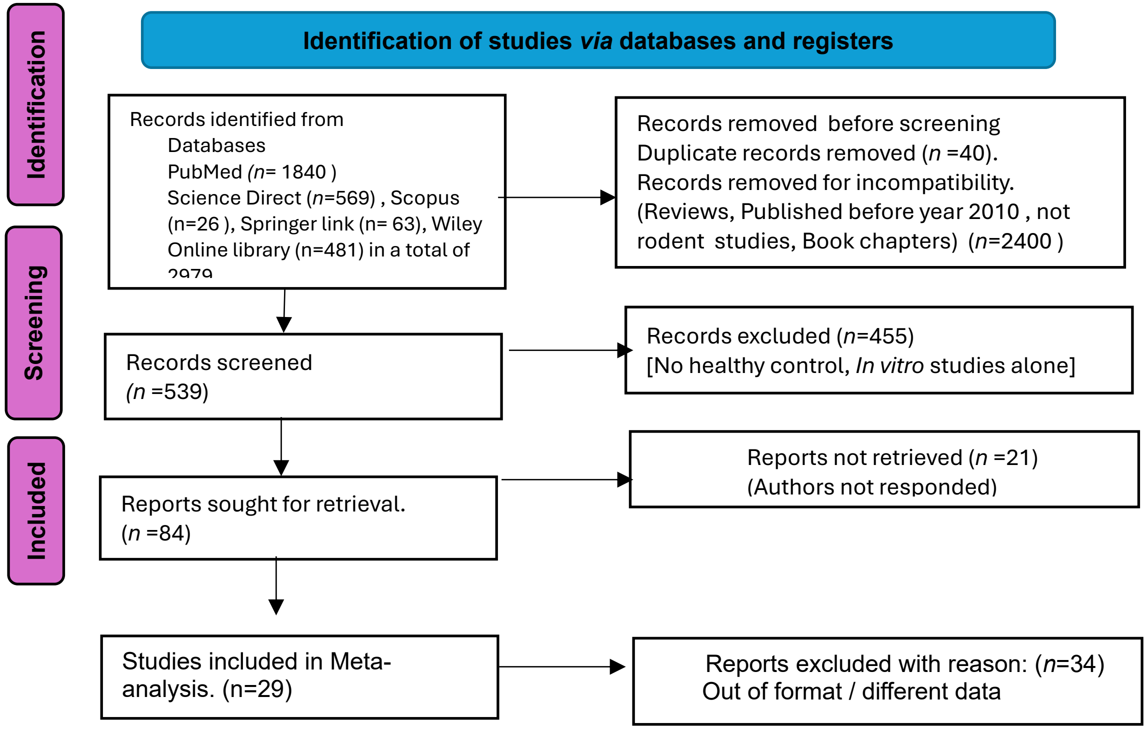

Figure 1: A modified version of the PRISMA flowchart of year 2020 used for reporting the included studies, (Adopted from the *Cochrane Collaboration. Org*).

<table>
<tr><td></td><td>16b</td><td>EXAMPLES OF EXCLUDED STUDIES
<table>
<tr><th>YEAR</th><th>FIRST AUTHOR</th><th>DOI/JOURNAL REFERENCE</th><th>ACCEPT/
REJECT</th><th>REASON</th></tr>
<tr><td>2016</td><td>Han Wu</td><td>http://dx.doi.org/10.1155/2016/3896147
or
Volume 2016, Article ID 3896147, 11 page</td><td>R</td><td>Review</td></tr>
<tr><td>2015</td><td>Tao Liu</td><td>International Journal of Cardiology 184C (1):318-320
or
DOI:10.1016/j.ijcard.2015.02.088</td><td>R</td><td>Clinical study</td></tr>
</table>
</td><td>15</td></tr>
<tr><td>STUDY CHARACTERISTICS</td><td>17</td><td>Table 1: Study Characteristics</td><td>9
-
13</td></tr>
</table>

STUDY #	YEAR	FIRST AUTHOR	RODENT MODEL	DISEASE TYPE	CONTROL/ TREATMENT #	STZ DOSE	BG CUT OFF POINT	SEX	AGE (WKS)	BODY WEIGHT (g)	PRIMARY OUTCOMES	REF
1	2010	Christian Volz	C57BL/6	DM	10/10	50mg/kg i.p. for 5days	>22 mM	M	10-14	NS	HMGB1, TNF-A, IL-6, NF-KB, pERK1/2, pJNK, TLR4, LVEDD, LVEDS	[34]
2	2012	Francesca Delucchi	Wistar rats (*Rattus norvegicus*)	T1D	25/54	60 mg/kg	13.8 mM	M	12-14	373.9 ±2.7	LVEDP, LVSP, HMGB1	[65]
3	2013	Beibei Luo	Sprague Dawley rats	T2D	15/15	35 mg/kg single i.p	≥11.1 mM	NS	NS	100-120	NLRP3, TC, TG, IL-1β	[66]
4	2014	Huiling Diao	Sprague Dawley rats	DM	10/10	50 mg/ kg	11.0 mM	M	NS	NS	HMGB1, TNF-A, IL-6, BG, NF-KB	[67]
5	2014	Wen-Ke Wang (January)	C57BL/6j	T1D	8/8	50mg/kg i.p. for 5 days	16 mM	NS	8-12	25-30	HMGB1, LVEDd, LVEF, FS%, BG, Fibrosis%, Collagen I, Collagen III, MMP9, TGF-1β	[68]
6	2014	Wen-Ke Wang	C57BL/6j	DM	8/8	60 mg/kg	≥16.7 mM	M	8	NS	HMGB1, ClC3, Bax/Bcl-2, ERK1/2	[69]

		(July)										
7	2015	Aibin Tao	C57BL/6	DM	3/3	50mg/kg i.p. for 3 days	>22 mM	M	6	NS	HMGB1, IL-33, Myocardial Fibrosis %, Collagen I, ESPVR, EDPVR	[35]
8	2016	Han Wu	Mice	DM	8/8	40 mg/kg i.p. for 5 days	>13.9mM	M	NS	30.4 ± 1.5	Serum HMGB1, HMGB1/β-Actin, BG, BW, RAGE, TLR4, NF-KB, TNF-A, iNOS, NADPH,	[71]
9	2017	Kapil Suchal	Albino Wistar rats	DM	10/12	70 mg/kg i.p. for 3 days	13.8 mM	M	10-12	150± 200	ERK1/2, phospho (p)-ERK1/2 (p-ERK1/2), c-Jun N-terminal kinase (JNK), p-JNK, NF-κBp65, caspase-3, β-actin, Bcl-2, Bax, p38, RAGE, TXNIP, GSH, MDA, SOD, CAT	[72]
10	2017	Nawal M. Al-Rasheed	Wistar rats	T1D	8/8	55 mg/kg single i. p	>11,11 mM	M	10	160-180	NO, GSH, MDA, SOD, TNF-a, CK-MB, LDH, troponin I, H&E, CRP, HDL-C, LDL-C. VLDL-C	[73]
11	2017	Wei-Fang Li	Sprague-Dawley rats	DM	8/8	60 mg/kg single i. p	NS	M	6-8	220-250	LVIDs, LVIDd, IVSd, FS%, GP, SOD, MDA, BW, HR	[74]

		12	2017	Weng-Ke Wang	C57/BL6J	DM	8/8	50mg/kg i.p. for 5 days	≥16.7 mM	M	6-8	25-30	PBG, HR, SBP, BW, LVIDs, EF%, TNF-a, IL-6, MAPK, P38, P-JNK, Bcl-2, HMGB1	[75]	
		13	2018	Hong-Wei Wang	Wistar rats	DM	6-8/6-8	50 mg/kg single i. p	≥16.7 mM	M	NS	200± 220	cTn-I, CK-MB, TGF-β1, α-SMA, p-Smad3, Bcl-2, cleaved-caspase-3, collagen I and III	[76]	
		14	2020	Yi Wang	C57BL/6 AND Sprague-Dawley rats	T1D	6/7	100 mg/kg single i.p	>12 mM	M	8	18-22, 200-220	NF-KB, MAPK, TLR4, MyD88, CK-MB, ANP, TGFβ1, MMP2, MMP9, AGEs, FBG, BW, IVSD, IVSS, TNF-a, IL-6	[78]	
		15	2020	Yuwei Zhang	C57BL/6	T1D, T2D	14/14	50 mg/kg	16.7 mM	M	6-8	NS	TLR4, ANP, TNF-a, IL-6, ICAM-1, VCAM-1, EF%, FS%, LVSV, LVDV, Type I and III collagen	[719]	
		16	2021	Hui Shi	Sprague Dawley rats	DM	15/15	35 mg/kg	16.7 mM	M	8-9	200 ± 20	TLR4, MyD88, NF-kB p65, TNF-a, IL-1b, IL-6, EF%, FS%, Bax, BCL-2, Caspase-3	[80]	
		17	2023	Eman A. E. Farrag	Sprague–Dawley rats	T2D	6/12	27.5 mg/kg single i.p.	> 11.1 mM	M	NS	160± 20	FBG, INS, CK-MB, LDH, HW/BW, H&E, HOMA-IR, AGE, RAGE, HMGB1, IL-1β, MDA, GSH	[82]	

		18	2023	Yingying Hu	Sprague-Dawley rats	T2D	8/8	30mg/kg i.p. for 3 days	>16.7 mM			180-220	NLRP3, NF-KB, TLR4, HMGB1, ASC, GSDMD, IL-1β, IL-18	[83]	
		19	2024	Marwa M. M. Refaie	Wistar albino rats	DM	10/10	45 mg/kg single i.p.	≥ 16.7 mM	M	4	90± 100	FBG, HbA1C, TAC, GSH, MDA, ATII, Caspase1, HW, HW/BW, NLRP3, TNF-A, IL-1β, NF-KB	[87]	
		20	2024	Feng Hu	C57BL/6N	T2D	6/6	100 mg/kg single i.p.	11.1 mM	M	8	NS	LVEF, LVSF, pERK 1/2/ERK 1/2	[45]	
		21	2024	Jinxiu Zhu	C57BL/6J	DM	10/10	50 mg/kg i.p. for 5 days	> 16.7 mM	M	4	NS	LVEF%, FS%, LVIDd, LVIDs, FBG, BW, HW, HW/BW, TLR4, NF-kB	[46]	
		22	2024	Liping Zhu	Albino Wistar rats	T2D	6/6	60 mg/kg single i.p.	11.11 mM	M	NS	180-200	IL-1β, IL-6, TNF-α, MDA, TGF-β1, BG, Caspase 3, TC, TG, HDL, LDL, CK-MB, CTPN	[47]	
		23	2024	Qihui Huang	Sprague Dawley rats	T2D	6/6	30 mg/kg single i.p.	≥ 11.1 mM	M	10	250-300	CK-MB, LDH, EF%, FS%, LVSV, MDA	[48]	
		24	2024	Vipin Kumar Verma	Albino Wistar rats	T1D	8/8	40 mg/kg single i.p.	22.2 mM	M	10-12	150-200	GSH, MDA, CK-MB, LDH, TNF-A, IL-6, Caspase 3, HMGB1.	[49]	
		25	2024		C57BL/6	T2D	6/6	80	NS	M	8	NS	EF%, FS%, LVIDd, LVIDs, Collagen I, Collagen III	[50]	

		**Ze-Yu Zhou**				**mg/kg single i.p.**						
**26**	**2024**	**Xuan Zhou**	**C57BL/ 6JNifdc**	**T2D**	**10/10**	**30 mg/kg i.p. for 7 days**	**≥ 11.1 mM**	**M**	**6**	**NS**	**CK-MB, LDH, TC, TG, IL-1β, IL-6, EF%, FS%, LVIDd, LVIDs**	**[51]**
**27**	**2025**	**Huiping Yang**	**C57BL/6J**	**T2D**	**5/5**	**40 mg/kg i.p. for 3 days**	**≥ 11.1 mM**	**M**	**3**	**NS**	**GSH, MDA, LDH, LVEF%, FS%, LVIDd, LVIDs**	**[52]**
**28a**	**2025**	**Weipin Niu**	**Piezo1 dtT/dtT**	**T1D**	**10/10**	**55 mg/kg i.p. for 5 days**	**> 16.7 mM**	**M**	**8**	**NS**	**EF%, FS%, LVIDd, LVIDs, HW, HW/TL**	**[53]**
**28b**	**2025**	**Weipin Niu**	**Piezo1 ΔMyh6**	**T2D**	**10/10**	**35 mg/kg i.p. for 3 days**	**> 16.7 mM**	**M**	**3**	**NS**	**EF%, FS%, LVIDd, LVIDs, HW, HW/TL**	**[53]**
**29**	**2025**	**Lixia Zhang**	**C57BL/6J**	**DM**	**10/10**	**60 mg/kg i.p. for 5 days**	**≥ 11.1 mM**	**M**	**6-8**	**NS**	**EF%, FS%, Fibrosis%, NLRP3, IL-1β**	**[54]**

<table>
<tr>
<td>RISK OF BIAS ASSESSMENT</td>
<td>18</td>
<td>

RISK OF BIAS

The 29 included studies were not devoid of risk of bias, but each study had at least 3 common confounders such as allocation concealment or blinding of the outcome (in performance and detection). All the studies stated the investigations were carried out in compliance with the Helsinki declaration. Therefore, it was unclear whether they had utilized the random sequence generation or not. However, not mentioning allocation concealment, blinding for performance and detection, all the studies have non-intuitively placed them under the high risk of bias category. Apart from not blinding the investigators and the participants, there were no other serious confounders that could have classified these studies as having high risk of bias.

There were no issues with the intervention or the outcome of results as the experimental groups were animals induced with diabetes and the quantification of the risk factors, or the biomarkers was quite straightforward which yielded a high or low quantification using the established standardized measurements. There was no apparent reporting bias because both positive and negative outcomes were reported in these studies. The issue of missing data did not arise in any of the included studies. All the biochemical investigations were mostly carried out as per the previously established protocols or analytical methods such as ELISA, immunofluorescence or IHC that was performed according to the manufacturer's specifications.

(a)

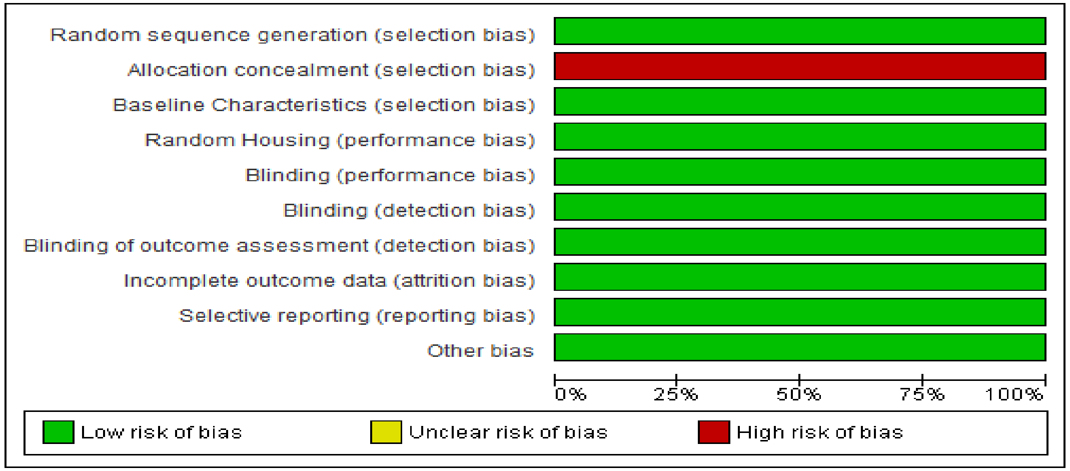

</td>
<td>16</td>
</tr>
</table>

			17

(b)

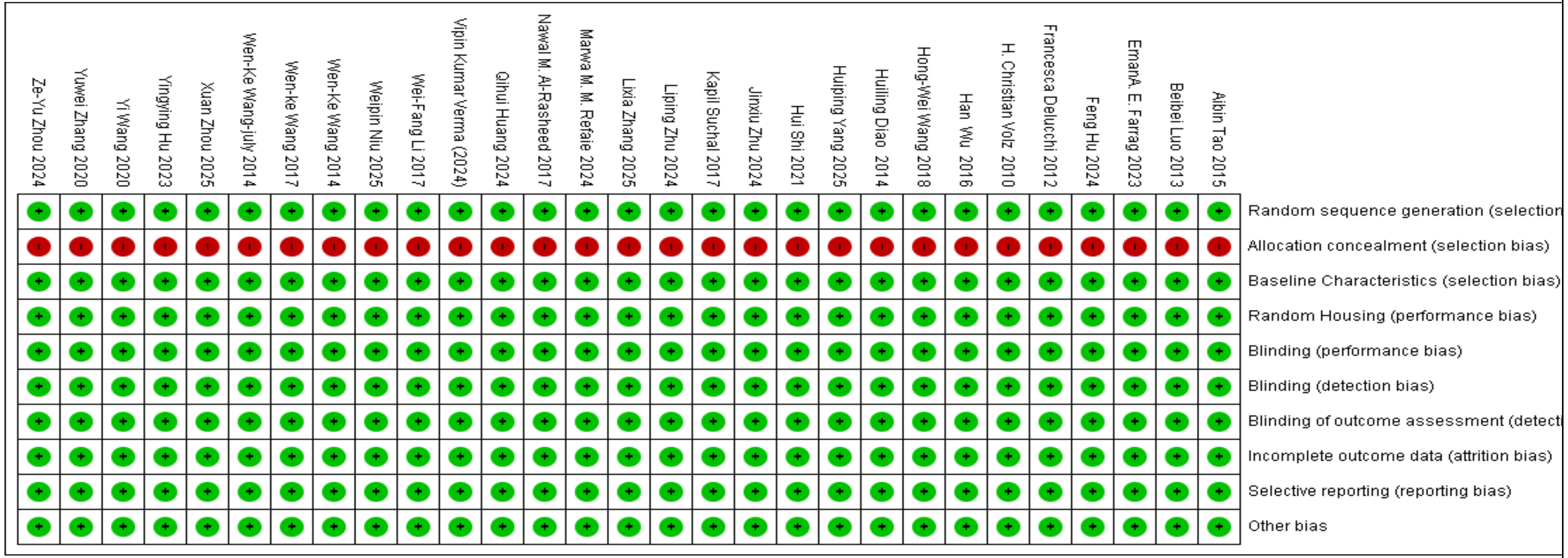
Ze-Yu Zhou 2024
Yuwei Zhang 2020
Yi Wang 2020
Yingying Hu 2023
Xuan Zhou 2025
Wen-Ke Wang-july 2014
Wen-ke Wang 2017
Wen-Ke Wang 2014
Weipin Niu 2025
Wei-Fang Li 2017
Vipin Kumar Verma (2024)
Qihui Huang 2024
Nawal M. Al-Rasheed 2017
Marwa M. M. Refaie 2024
Lixia Zhang 2025
Liping Zhu 2024
Kapil Suchal 2017
Jinxiu Zhu 2024
Hui Shi 2021
Huiping Yang 2025
Huiling Diao 2014
Hong-Wei Wang 2018
Han Wu 2016
H. Christian Volz 2010
Francesca Delucchi 2012
Feng Hu 2024
EmanA. E. Farrag 2023
Beibei Luo 2013
Aibin Tao 2015
Random sequence generation (selection
Allocation concealment (selection bias)
Baseline Characteristics (selection bias)
Random Housing (performance bias)
Blinding (performance bias)
Blinding (detection bias)
Blinding of outcome assessment (detect
Incomplete outcome data (attrition bias)
Selective reporting (reporting bias)
Other bias

(b)

		Figure 2 (a -b): The Risk of Bias (ROB) assessment of this present study as per the SYRCLE criteria of the Cochrane Organisation guidelines for pre- clinical studies. (a) The summary of the overall ROB, (b) ROB of the individual included studies.	
	19	**H. Christian Volz (2010)**	

RISK FACTOR	DCM MEAN	SD	n	CONTROL MEAN	SD	n
HW/BW	6.5	6.32	10	4	3.16	10
pERK1/2	4	12.64	10	1	1.58	10
pJNK	4.5	6.32	10	1	1.58	10
LVID-d	6	3.16	10	3	1.58	10
FS%	30	3.16	10	48	3.16	10
NF-KB (mRNA)	7	12.64	10	1	1.58	10
TLR4	1.25	1.58	10	1	1.58	10
TGF-B (mRNA)	5.5	12.64	10	1	3.16	10
HMGB1 (mRNA)	6.5	3.16	10	1	1.58	10

Francesca Deluchchi (2012)

RISK FACTOR	DCM MEAN	SD	n	CONTROL MEAN	SD	n
HMGB1 (OD)	0.9	15.57	64	0.7	5.19	54

Beibei Luo (2013)

RISK FACTOR	DCM MEAN	SD	n	CONTROL MEAN	SD	n
HW/BW	4.8	0.24	6	3.8	0.24	6

LVID-d	7.5	0.24	6	6.2	1.22	6
EF%	50	2.44	6	82	4.88	6
FS%	35	4.88	6	55	4.88	6
TNF-A	125	3.46	3	25	1.73	3
NF-KB	12.5	3.46	3	1	0.17	3
IL-1β	3.75	0.8	3	1	0.4	3
IL-6	90	3.46	3	10	0.17	3
NLRP3	3.5	1.73	3	1	0.4	3
Caspase 1	2.5	3.4	3	1	0.17	3
ASC	4.5	1.7	3	1	0.17	3
TC	4.5	1.7	3	1	0.17	3
TG	2.72	0.08	8	0.65	0.06	8

Huiling Diao (2014)

**RISK FACTOR**	**DCM MEAN**	**SD**	**n**	**CONTROL MEAN**	**SD**	**n**
BG	18.1	2.1	10	5.1	0.5	10
BP	115	9	10	108	9	10
TNF-A	27	7	10	25	5	10
IL-6	121	17	10	113	15	10
CTPN	0.19	0.05	10	0.17	0.03	10
TG	175	12.2	6	50	1.22	6

Wen-Ke Wang-January (2014)

**RISK FACTOR**	**DCM MEAN**	**SD**	**n**	**CONTROL MEAN**	**SD**	**n**
Fibrotic %	25	11.2	8	9	2.8	8
LVID-d	3.5	2.8	8	3.4	2.8	8
EF%	55	11	8	77.6	7.1	8

FS%	21.3	4.1	8	30.1	3.42	8

Wen-Ke Wang-July (2014)

RISK FACTOR	DCM MEAN	SD	n	CONTROL MEAN	SD	n
CC3	2.5	1.4	8	1	2.8	8

Aibin Tao (2015)

RISK FACTOR	DCM MEAN	SD	n	CONTROL MEAN	SD	n
LVDV	0.4	4.88	6	0.18	1.22	6
LVSV	2.9	2.44	6	5.4	2.44	6
HMGB1/β Actin	**1.2**	**6.92**	**3**	**0.65**	**1.73**	**3**

Han Wu (2016)

RISK FACTOR	DCM MEAN	SD	n	CONTROL MEAN	SD	n
BG	27.2	2.8	8	7.4	0.5	8
BW	23	1.5	8	30.4	1.5	8
HMGB1/ β-Actin	2.75	2.8	8	1	2.8	8
HMGB1 (serum)	425	11.2	8	325	2.8	8
TNF-A / β-Actin	1.6	2.8	8	1	5.6	8
RAGE/ β-Actin	1.5	2.8	8	1	1.4	8
TLR4/ β-Actin	3.5	2.8	8	1	2.8	8
iNOS/ β-Actin	1.3	5.6	8	1	2.8	8
NF-kB/ β-Actin	1.5	1.4	8	1	1.4	8

Wen-Ke Wang (2017)

RISK FACTOR	DCM MEAN	SD	n	CONTROL MEAN	SD	n
BG	24.36	3.37	8	6.11	1.06	8
BW	24.7	1.08	8	27.87	1.72	8
BP	102.75	14.3	8	108.24	13.9	8
TNF-A	0.35	3	8	0.1	2	8
IL-6	0.5	2.5	8	0.22	2	8
P-JNK	3.25	2	8	1	1	8
p-38	1.75	2	8	1	1	8
HMGB1 (mRNA)	3	5.6	8	1	2.8	8
CC3	2.5	5.6	8	1	5.6	8

Kapil Suchal (2017)

RISK FACTOR	DCM MEAN	SD	n	CONTROL MEAN	SD	n
BG	505.31	28.98	6	503.5	26.81	6
BW	134.04	8.71	6	132.32	6.85	6
NF-KB	1	1.73	3	0.7	1.73	3
LDH	764.98	69.29	6	505.94	53.65	6
CK-MB	662.6	62.43	6	422.66	57.87	6
IL-6	28.06	1.87	6	19.06	1.63	6
TNF-A	23	2.36	6	15.5	2.02	6
AGEs	26.54	3.14	6	24.89	1.61	6
MDA	103.41	9.88	6	67.19	7.58	6
CK-MB	662.6	62.43	6	422.66	57.87	6
GSH	0.5	0.122	6	1.02	0.17	6
SOD	2.1	1.24	6	4.35	1.17	6
CAT	3.22	0.7564	6	5.96	1.36	6

Nawal M. Al-Rasheed (2017)

RISK FACTOR	DCM MEAN	SD	n	CONTROL MEAN	SD	n
TC	200	2.8	8	55	2.8	8
TG	175	2.8	8	60	2.8	8
HDL	19	2.8	8	35	2.8	8
LDL	150	2.8	8	20	2.8	8

Wei-Fang Li (2017)

RISK FACTOR	DCM MEAN	SD	n	CONTROL MEAN	SD	n
HR	380	2.8	8	180	1.4	8
BW	250	2.8	8	248	2.8	8
BMI	31	4	25	25	4	20
LVID-d	70	2.8	8	80	2.8	8
LVID-s	50	2.8	8	30	2.8	8
FS%	80	2.8	8	100	2.8	8
SOD	25	2.8	8	5	2.8	8
MDA	9	2.8	8	3	2.8	8
Nrf2	0.4	11.2	8	1	1.4	8

Hong -Wei Wang (2018)

RISK FACTOR	DCM MEAN	SD	n	CONTROL MEAN	SD	n
BG	28	2.44	6	8	2.44	6

BW	410	2.44	6	110	2.44	6
HW/BW	5.2	12.2	6	2	4.88	6
Fibrotic area %	54	12.2	6	5	1.22	6
C-TPN (µg/ml)	440	21.96	6	40	2.44	6
CK-MB (ng/ml)	85	12.2	6	19	9.76	6
Cleaved caspase-3	3.4	9.6	6	0.5	4.8	6

Yi Wang (2020)

RISK FACTOR	DCM MEAN	SD	n	CONTROL MEAN	SD	n
BW (g)	25.3	1	7	29.68	2.32	6
HW (mg)	117.8	3.75	7	125.1	8	6
HW/BW	4.66	0.081	7	4.218	0.159	6
HW/TL	9	8.4	8	7	5.6	8
Fibrotic area %	15	5.6	8	3	2.8	8
LVID-d (mm)	0.74	0.02	7	0.84	0.03	6
LVID-s (mm)	0.95	0.053	7	1.1	0.2	6
EF%	70.57	6.07	7	82.12	4.88	6
FS%	36.14	5.28	7	44.48	4.63	6
TNF-A (mRNA)	3.8	5.19	3	1	1.73	3
TLR4 in heart tx	0.7	10	4	0.19	4	4
MD2 in heart tx	0.78	8	4	0.1	4	4
AGEs (pg/ml) Cell culture medium	598	4	8	225	4	8
AGEs (pg/mg) In heart tx	102	4.88	6	52	7.32	6
AGEs in serum (µg /ml)	22	21	9	13	11.2	8

Yuwei Zhang (2020)

RISK FACTOR	DCM MEAN	SD	n	CONTROL MEAN	SD	n
BG (mM)	20.49	1.63	14	11.56	1.72	14
LVSV	35	7.48	14	11	3.74	14
LVDV	68	7.48	14	38	3.74	14
EF%	48.26	5.21	14	67.18	4.73	14
FS%	27.33	3.2	14	35.33	1.74	14
TLR4	0.8	0.5	14	4.4	4	14
IL-1β (mRNA)	6.2	2	14	1	0.5	14
TNF-A (mRNA)	5	14.8	14	1	3.7	14
TGF-β (mRNA)	3.5	6.92	3	1.4	1.73	3
Collagen I (mRNA)	3	6.92	3	0.8	0.865	3
Collagen III (mRNA)	3.3	5.19	3	1.3	1.73	3
ICAM-1	5	5.19	14	1	0.865	14
VCAM-1	4.3	6.05	14	1.3	3.46	14

Hui Shi (2021)

RISK FACTOR	DCM MEAN	SD	n	CONTROL MEAN	SD	n
EF%	40	15.2	15	68	19	15
FS%	28	11.4	15	48	15.2	15
NF-kB (mRNA)	8.5	3.16	10	1	1.58	10
TLR4	8.5	3.16	10	1	1.58	10
MD2	3.8	3.16	10	1	1.58	10
IL-1β (pg/ml)	90	9.48	10	50	7.9	10
IL-6 (pg/ml)	52	7.9	10	28	7.9	10
TNF-A (pg/ml)	68	6.32	10	34	6.32	10

Eman A. E. Farrag (2023)

RISK FACTOR	DCM MEAN	SD	n	CONTROL MEAN	SD	n
BG (mg/dl)	396	18.24	6	96.67	2.8	6
Serum insulin (ng/ml)	0.106	0.006	6	0.223	0.004	6
HR (bpm)	224.83	19.85	6	102.83	6.49	6
BW (g)	229	9.9	6	270.8	14.4	6
HW (mg)	1265	69	6	692	51	6
HW/BW	5.53	0.3	6	2.57	0.31	6
HOMA-IR	2.99	0.31	6	1.54	0.06	6
Apoptosis Rate	44	15.2	15	14	11.4	15
IL-1β (pg/mg)	110.5	2.97	6	47.87	1.12	6
LDH (U/L)	2235	14.14	6	1400	13.04	6
AGEs (ng/mg)	3.55	0.27	6	0.82	0.08	6
HMGB1	23	17	6	4	3.66	6
MDA	81.72	3.5	6	31.77	1.62	6
CK-MB	0.51	0.04	6	0.36	0.01	6
GSH (mmol/mg)	0.4	0.08	6	0.95	0.09	6

Yingying Hu (2023)

RISK FACTOR	DCM MEAN	SD	n	CONTROL MEAN	SD	n
BG (mM)	18	2.23	5	4	1.115	5
EF%	40	2	4	79	2	4
FS%	20	2	4	45	2	4
GSDMD	1.5	2	4	1	1	4

IL-1β	2.5	8	4	1	4	4
Cell Viability	0.3	0.865	3	0.6	1.73	3
Caspase I	1.5	2	4	1	1	4

Marwa M. M. Refaie (2024)

**RISK FACTOR**	**DCM MEAN**	**SD**	**n**	**CONTROL MEAN**	**SD**	**n**
BG (mM)	21.6	3.2	10	7.3	1.2	10
HW (mg)	443.1	35.9	10	306	25.2	10
BP (Hg mm)	164.5	12.1	10	107.1	8	10
HbA1c%	8.6	1.1	10	4.5	0.7	10
Ang II (pg/ml)	516	61.9	10	200.3	13.6	10
NF-kB (ng/ml)	42.2	7.1	10	24	4	10
LDH (U/L)	271.1	18	10	150.6	8.8	10
NLRP3 Inflammasome (pg/ml)	43.9	7.4	10	9.8	1.3	10
CK-MB (U/L)	60.7	9.8	10	42	7.3	10
TNF-A (pg/ml)	57.5	10.6	10	29.1	4	10
C-TPN (ng/ml)	6.9	1.2	10	0.8	0.1	10
Caspase I (pg/ml)	202.5	20.8	10	17.4	3	10
MDA (nmol/mg)	11.4	2.2	10	4.1	0.7	10
TAC (mmol/L)	0.5	0.06	10	0.9	0.08	10

Caspase III	35	6.32	10	1	1.58	10

Feng Hu (2024)

RISK FACTOR	DCM MEAN	SD	n	CONTROL MEAN	SD	n
EF%	56	1.11	5	58	2.23	5

Jinxiu Zhu (2024)

RISK FACTOR	DCM MEAN	SD	n	CONTROL MEAN	SD	n
BG (mM)	25.0	1.0	10	8.0	0.5	10
HW (mg)	121	2.46	10	103	1.75	10
HW/BW	4.55	0.19	6	3.78	0.09	10
LVIDd (mm)	3.9	1.22	6	3.3	1.22	6
LVIDs (mm)	2.55	0.131	10	1.87	0.045	10
EF%	67.4	2.75	10	42.2	1.08	10
BW (g)	26.8	3.0	10	27.5	2.0	10

Liping Zhu (2024)

RISK FACTOR	DCM MEAN	SD	n	CONTROL MEAN	SD	n
TG (mg/dl)	225.0	3.0	6	49.0	1.0	6

MDA (nmol/mg)	3.45	0.27	6	1.64	0.28	6
IL-6 (pg/mg)	441.34	17.42	6	254.11	8.64	6
TC (mg/dl)	220.0	3.0	6	50.0	1.0	6
HDL (mg/dl)	28.0	2.0	6	38.0	1.0	6
TGF-1β (pg/ml)	380.0	5.0	6	100.0	1.0	6
LDL (mg/dl)	90.0	2.0	6	22.0	1.0	6
CK-MB (U/L)	1900.0	2.0	6	1200.0	3.0	6
TNF-A (pg/ml)	35.62	0.82	6	17.46	1.18	6
C-TPN (pg/ml)	135	2	6	10	1	6

Qihui Huang (2024)

**RISK FACTOR**	**DCM MEAN**	**SD**	**n**	**CONTROL MEAN**	**SD**	**n**
HR (bpm)	378	0.5	6	380	0.7	6
EF%	68	2	6	78	1	6
LVIDs	4.0	3.0	6	3.8	2.0	6
CK-MB (U/L)	2300.0	2.0	6	1100.0	10.0	6

Vipin Kumar Verma (2024)

**RISK FACTOR**	**DCM MEAN**	**SD**	**n**	**CONTROL MEAN**	**SD**	**n**
NF-KB	1.25	0.346	3	1.0	0.346	3
IL-6 (pg/ml)	44.0	1.73	3	28.0	1.73	3

pERK 1 /2/ t-ERK 1/ 2 (fold change)	0.6	0.2	6	1.0	0.2	6
CK-MB (U/L)	500.0	4.88	6	450.0	2.44	6
MDA (nmol/mg)	78.0	1.0	6	68.0	0.5	6

Ze-Yu Zhou (2024)

RISK FACTOR	DCM MEAN	SD	n	CONTROL MEAN	SD	n
BG (mM)	20.0	12.2	5	5.0	4.88	6
sINS	75.0	4.88	6	50.0	1.22	6
LVIDd	5.0	4.88	6	4.0	2.44	6
LVIDs	3.9	4.88	6	3.0	2.44	6
LVSV	54.0	4.0	6	50.0	2.0	6
HW/BW	6	2.23	6	5.5	4.46	6
EF%	41	1.22	6	55	2.44	6
Col I (Relative expression)	2.4	15.61	5	1.5	11.15	5
Col III (relative expression)	2.2	20.07	5	1.5	11.15	5

Xuan Zhou (2024)

RISK FACTOR	DCM MEAN	SD	n	CONTROL MEAN	SD	n
TG	2.41	0.88	10	1.36	0.18	10
sINS	6.0	1.0	10	8.0	5.0	10

IL-6 (pg/ml)	66.23	13.82	10	38.52	1.78	10
IL-1β (pg/ml)	24.84	4.84	10	18.23	2.4	10
TC (mM)	3.72	0.91	10	1.99	0.43	10
CK-MB (U/L)	4.81	1.41	10	3.14	0.95	10
TNF-A (pg/ml)	92.61	11.05	10	80.4	4.44	10

Huiping Yang (2024)

RISK FACTOR	DCM MEAN	SD	n	CONTROL MEAN	SD	n
BG (mM)	34.0	1.0	5	8.0	1.0	5
EF%	50	9	5	82	7	5
LVIDd (mm)	3.2	11.0	5	2.9	8.0	5
LVIDs (mm)	2.2	6.0	3	1.6	12.0	5
sINS	12.5	1.0	5	6.5	1.0	5
GSH	45.0	2.0	3	65.0	5.0	3
LDH (U/L)	1000.0	2.0	3	850.0	1.0	3
MDA (nmol/g)	42.0	4.0	3	35.0	2.0	3

Wipin Niu (2025)

RISK FACTOR	DCM MEAN	SD	n	CONTROL MEAN	SD	n
EF%	55	2.44	6	75	2.44	6
LVIDd	3.9	1.22	6	3.3	1.22	6

LVIDs	3.9	1.22	6	2.2	1.22	6
Col I (relative intensity)	0.38	2.23	5	0.18	1.11	5
Col III (relative intensity)	0.25	2.23	5	0.18	1.22	5

Lixia Zhang (2025)

**RISK FACTOR**	**DCM MEAN**	**SD**	**n**	**CONTROL MEAN**	**SD**	**n**
IL-1β (β-Actin)	1.3	6.32	10	0.7	3.16	10
EF%	36	15.8	10	50	12.64	10
Fibrosis %	8.0	15.8	10	2.5	12.64	10
NLRP3 Inflammasome (pg/ml)	0.8	18.96	10	0.25	6.32	10

RESULTS OF SYNTHESIS	20a	**MODEL 1**<br><br>Model I consists of the **AGEs** (SMD 5.73, 95% CI 1.73, 9.13; p < 0.10) and **HMGB1** (SMD 3.00, 95% CI 1.58, 4.42; p < 0.0001) titres in the vascular tissue measured with a forest plot and non-parametric-Mann Whitney U (MWU) test bar graph. This AGEs were quantified by 3 included studies while HMGB1 was measured by 7 included studies. This model had 40 animals in total in the DCM group matched by 41 animals in the control group for the AGEs and 133 animals in the DCM group matched by 123 animals in the control group for HMGB1. The MWU -statistic of the AGEs was (U=3, *p*=0.7) and that of the HMGB1 was (U= 0, *p*=0.0286). The area under the curve plotted from a ROC was (AUC = 0.7778, *P*=0.2752) for the AGEs and (AUC=1.00, *p*=0.0209) for HMGB1. A significant increase in the HMGB1 titres was observed in the DCM group compared to the control, indicating its potential to upregulate	34

		inflammation in the vascular tissue. The AGEs and HMGB1 were reported together in one model because HMGB1 serve as a ligand for the receptor of the AGEs which are the RAGE although it was not quantified by any of the included studies.<br><br>**MODEL 2**<br><br>Model 2 consists of 12 **cardiometabolic biomarkers** which included the heart rate (HR) (SMD 12.20, 95% CI -1.59, 26.00; p = 0.08), heart weight (HW) SMD 4.73, 95% CI 0.09, 9.37; p = 0.05), heart weight to body weight ratio (HW/BW) (SMD 2.31, 95% CI 0.63, 3.99; p = 0.007), left ventricular internal dimension at end -diastole (LVIDd) SMD -0.09, 95% CI -1.62, 1.44; p = 0.91), left ventricular internal dimension at end -systole (LVIDs) (SMD 1.64, 95% CI 0.04, 3.23; p = 0.04), left ventricular volume at end-diastole (LVDV), (SMD 4.93, 95% CI 3.35, 6.50; p = 0.00001), left ventricular volume at end-systole (LVSV), (SMD 1.38, 95% CI -1.38, 4.13; p =0.33), ejection fraction percentage (EF%) (SMD -4.13, 95% CI -5.56, -2.69; p = 0.00001), fractional shortening percentage (FS%) (SMD -2.91, 95% CI -3.96, -1.86; p = 0.00001), creatine kinase in the muscles and brain (CK-MB) (SMD 3.28, 95% CI 1.04, 5.52; p =0.004), cardiac troponin (C-TPN) (SMD 37.93, 95% CI 4.28, 71.58; p =0.03), lactate dehydrogenase (LDH) (SMD 5.66, 95% CI 2.48, 8.84; $p < 0.0005$), and blood pressure (BP) (SMD 1.71, 95% CI -0.71, 4.13; p = 0.17) which were measured in the vascular tissue of rodents comprising mice and rats and were assessed with a forest plot, Mann Whitney U test and the ROC. The number of studies from which these biomarkers were reported are HR (3), HW (4), HW/BW (6), LVIDd (8), LVIDs (7), LVDV (1), LVSV (3), EF% (13), FS% (12), CK-MB (8), C-TPN (4), LDH (7), and BP (3). The number of animals utilized in the control and DCM groups respectively, for each biomarker included in brackets were HR (20, 20), HW (32, 33), HW/BW (44, 45), LVIDd (59, 60), LVIDs (47, 46), LVDV (14, 14), LVSV (26, 26), EF% (103,104), FS% (93, 94), CK-MB (56, 56), C-TPN (30, 30), LDH (47, 47) and BP (28, 28). The Mann Whitney U test was carried out with all the biomarkers in Model 2 and the U statistics are: HR (U = 2.5, p = 0.5), HW (U= 7.0, p = 0.8857), HW/BW (U=3.0, p = 0.0152), EF% (U= 20.0, p = 0.0004), FS% (U = 28.0, p =0.0093), LVIDd (U = 23.0, p = 0.3128), LVIDs (U = 16.50, p = 0.3281), LVSV (U = 4.0, $p > 0.9999$), CK-MB (U = 13.0, p = 0.4848), C-TPN (U = 6.0, p = 0.6857), LDH (U = 19, p = 0.5350) and BP (U = 3.0, p = 0.7). The area under the curve plotted from a ROC was HR (AUC = 0.7222, p=0.3827), HW (AUC = 0.5625, p=0.7728), HW/BW (AUC = 0.9167, p=0.0163), EF% (AUC = 0.8225, p=0.0052), FS% (AUC = 0.8056, p=0.0111), LVIDd (AUC = 0.6406 p=0.3446), LVIDs (AUC = 0.6633, p=0.3067), LVSV (AUC = 0.5556, p=0.8273), CK-MB (AUC = 0.6389, p=0.4233), C-TPN (AUC = 0.6875, p=0.3865), LDH (AUC 0.6122, p=0.4822), and BP (AUC = 0.6667, p=0.5127). The ejection fraction percentage and the fractional shortening percentage were significantly decreased in the DCM group and the LVSV was significantly increased in the DCM group compared to the control, indicating the presence of established DCM and it is exacerbated by reduced EF%, FS% and increased LVSV in the rodents that were investigated. The correlation coefficients were calculated for the 13 cardiometabolic biomarkers and HMGB1 nuclear protein as the dependant variable with computing the Pearson's correlation coefficient (r), (R$^{2)}$ and the probability in HR (r= -0.9998, R^2 =0.9997, p =0.0113), HW (r=0.8545 R^2=0.7301, p=0.1455), HW/BW (r= -0.9551, R^2=0.9122, p=0.0449), EF% (r=0.9580, R^2=0.9178, p=0.0420), FS% (r=-0.8289, R^2= 0.6870, p=0.1711), LVIDD (r= -0.5847, R^2= 0.3418, p=0.4153), LVIDS	35

		(r=0.5464, R^2= 0.2986, *p*= 0.4536), LVSV (r=0.6635, R^2= 0.4402, *p*= 0.3365), CK-MB (r= -0.4633, R^2 =0.2147, *p*= 0.5367), C-TPN (r = -0.1332, R^2= 0.01775, *p*=0.8668), LDH (r= -0.6803, R^2= 0.4628, *p*=0.3197) and BP (r= -0.3226, R^2= 0.1041, *p*=0.7909).<br><br>**MODEL 3**<br><br>Model 3 is known as the **glycaemic biomarker model,** consisting of the blood glucose level (BG), the serum insulin level (sINS) and the body weight (BW). These three parameters are vital for managing *Diabetes mellitus*, which is a metabolic disorder that develops diabetic cardiomyopathy (DCM) as a secondary complication. BG was measured in two different units, mM and mg/dl but was treated as one group measured in mM units in the forest plot. The results showed a significant increase in BG in the DCM group compared to its control as hyperglycaemia is the primary risk factor of DM. BG (SMD 7.54, 95% CI 4.97, 10.12, *p* = 0.00001), sINS (SMD -9.46, 95% CI -27.72, 8.80, p > 0.31) and BW (SMD -1.74, 95%CI -3.46, -0.02, *p* =0.05) are risk factors as well as biomarkers of DMC although the sINS and BW produced non-significant outcomes. BG was reported by a total of 12 included studies divided into 10 and 2 in the first and second subgroups respectively. The sINS were reported by 4 studies and BW was reported by 7 studies. The number of animals used in the control and the DCM groups comprise of BG (94, 93), sINS (16, 16), and BW (52, 53) respectively. BG was a biomarker which produced a significant increase in its level in the non-parametric Mann Whitney U test compared to the control (U= 9, *p* < 0.0001) while the other two showed no significant difference compared to the control. The ROC data include the area under the curve with the probability given as BG (AUC = 1.000, *p* = 0.0001), sINS (AUC = 0.7500, *p*=0.4386) and BW (AUC = 0.6327, *p* = 0.4062). The correlation coefficient analysis was carried out between these 3 glycaemic biomarkers and HMGB1 nuclear protein as the dependant variable with computing the Pearson's correlation coefficient (r), (R^2) and the probability in BG (r=0.5962, R^2=0.3555, *p*=0.4038) , sINS (r= -0.3042, R^2= 0.09254, *p*= 0.6958), and BW for mice (r= 0.4763, R^2= 0.2268, *p*= 0.5237).<br><br>**MODEL 4**<br><br>Model 4 is known as the **lipid biomarker model**, consisting of the total cholesterol level (TC), and the total triglyceride level (TG). These two parameters are vital for managing diabetic cardiomyopathy (DCM) caused by dyslipidaemia or hypercholesterolemia which are high risk factors of DCM. TC and TG were measured in two different units, mM and mg/dl treating but were converted into one group in the forest plot. The results showed a significant increase in both TC and TG in the DCM group compared to its control. TC (SMD 7.38, 95% CI 1.85, 12.92, *p* =0.009), and TG (SMD 16.60, 95%CI 7.01, 26.19, *p* =0.0007) are risk factors as well as biomarkers of DCM and TC was reported by a total of 4 included studies and TG was reported by a total of 5 studies. The number of TC animals used in the control and the DCM groups comprised of (32, 32) and TG (38, 38) respectively. Both biomarkers produced non-significant increases in each level in the non-parametric-Mann Whitney U test compared to the control in TC (U=0, *p* =0.0286) while TG showed (U=3, *p* = 0.0476)] compared to the control. HDL and LDL were reported only by one study; hence those two parameters were excluded. The ROC data include	38<br><br>38

		the area under the curve with the probability given as for TC (AUC = 1.000, *p* = 0.0209) and TG (AUC = 0.8800, *p* = 0.0472). The correlation coefficient analysis was carried out between these 2 lipid biomarkers and HMGB1 nuclear protein as the dependant variable and calculating the Pearson's correlation coefficient (r), (R^2) and the probability in TC (r= -0.5215, R^2= 0.2720, *p*= 0.4785), and in TG (r= -0.5156, R^2= 0.2658, *p*= 0.4844).<br>**MODEL 5**<br>Model 5 is known as the **oxidative stress biomarker model**, consisting of the glutathione level (GSH), and the malondialdehyde level (MDA). These two parameters are vital for managing DCM caused by lipid peroxidation and high ROS generation thereby increasing inflammation which makes GSH and MDA high risk factors of DCM. GSH and MDA were measured in three and two different units respectively, but we treated them as one group each in the respective forest plots. The results showed significant changes in both GSH and MDA in the DCM groups compared to the control in the forest plots. GSH was markedly reduced (SMD – 4.60, 95% CI – 8.04, - 1.16, p <0.009), and MDA was markedly elevated (SMD 4.78, 95%CI2.71, 6.86, p <0.00001) as they are biomarkers of DCM. GSH was reported by a total of 4 included studies. MDA was reported by a total of 7 studies. The number of animals used in the control and the DCM groups comprised of (29, 29) in the GSH group and (45, 45) in the MDA group respectively. Both biomarkers produced no significant differences in each biomarker in the non-parametric Mann Whitney U test compared to the control: GSH (U= 5.000, *p* =0.4857) while MDA showed (U= 15.0000, *p* = 0.2593)] compared to the control. The ROC data include the area under the curve with the probability given as (AUC = 0.6875, *p* = 0.3865) for GSH and (AUC = 0.6939, *p* = 0.2248) for MDA. The Pearson's correlation coefficient (r), (R^2) and the probability in GSH (r= 0.4644, R^2 = 0.2157, *p* =0.5356), and in MDA (r= 0.6932, R^2 = 0.4805, *p* =0.3068) were calculated.	39
		**MODEL 6**<br>Model 6 comprises of the **inflammatory biomarker model**, consisting of the pro-inflammatory cytokines: TNF -alpha (SMD 1.11, 95% CI 0.11, 2.11, p =0.03), IL-6 (SMD 5.49, 95% CI 3.35, 7.64, *p* <0.00001), and IL-1β (SMD 4.44, 95% CI 1.56, 7.32, *p* =0.003). These 3 biomarkers should be downregulated to manage DCM caused by widespread severe inflammation which otherwise will upregulate systemic inflammation making TNF-A, IL-6 and IL-1β high risk factors of DCM. The total animal number utilised in TNF-A was 66 in the control group and 66 in the DCM group, in IL-6 there were 93 in the control group and 93 in the DCM group. In IL-1β there were 46 in the control group and 46 in the DCM group. All 3 biomarkers showed no difference in the DCM group compared to the control in the non-parametric Mann Whitney U- test given by TNF-A (U= 6.0, *p* = 0.6856), IL-6 (U= 10.0, *p* = 0.2229) and IL-1β (U= 1.0, *p* = 0.6667). The ROC in each biomarker was (AUC = 0.6250, *p* = 0.5637) for TNF-A, and (AUC = 0.8, *p* = 0.1172) for IL-6, but not for IL-1β. The correlation coefficient analysis between each of the 3 biomarkers and the HMGB1 was quantified with the Pearson's correlation coefficient, the	39

		coefficient of determination and the probability are reported as: TNF-A (r = - 0.6723, R^2 = 0.4520, *p* = 0.3277), IL-6 (r = - 0.3846, R^2 = 0.1479, *p* = 0.6154), IL-1β (r = -0.1238, R^2 = 0.01532, *p* = 0.9210).<br><br>**MODEL 7**<br><br>Model 7 consists of the **signalling pathway biomarker model** that is associated with the signalling pathways that involve the systemic inflammation that is inherent in the subjects of DCM. This model includes 7 types of molecules namely, NF-KB, TLR4, NLRP3 inflammasome, pERK 1 / 2 /t-ERK 1/ 2, pJNK /t-JNK, Cleaved caspase 3 (CC3), and TGF-β. All these molecules are mostly upregulated in the DCM group in their respective forest plots, and some are downregulated. Several molecules showed high strong positive correlation or negative correlation with HMGBI, the nuclear protein under investigation in this study in correlation analysis. Among the upregulated signalling molecules in the forest plots include NF-kB (SMD 3.13 95%CI 1.52, 4.74, *p*=0.0001), TLR4 (SMD 2.38, 95%CI 0.61, 4.15, *p* =0.009), CC3 (SMD 3.91 95%CI 1.46, 6.36 *p* <0.002), NLRP3 inflammasome (SMD 3.59, 95%CI 0.28, 6.89, *p* =0.03), pERK 1 /2 /t- ERK 1/ 2, (SMD 0.46, 95% CI -0.67, 1.59, *p* <0.42) , pJNK /t-JNK (SMD 1.87 95%CI 0.95, 2.79, *p* =0.0001), and TGF-β (SMD 1.50 95%CI -0.03, 3.03 *p*=0.05). The Mann Whitney U - statistic and probabilities of each molecular type are: NF-KB (U= 0.0, *p* = 0.0286), TLR4 (U=3.0, *p* = 0.200), pERK 1 / 2 /t-ERK 1/ 2 (U= 3.5, *p* = 0.80), pJNK /t-JNK (U= 1.5, *p* = 0.30), and TGF-β (U= 1.0, *p* = 0.2). The ROC in each biomarker was (AUC = 0.9375, *p* = 0.0433) for NF-kB, (AUC = 0.8125, *p* = 0.1489) for TLR4, and (AUC = 0.6111, *p* = 0.6625) for pERK 1 / 2 /t-ERK 1/ 2, (AUC = 0.8333, p = 0.1904) for pJNK /t-JNK, (AUC = 0.8889, p = 0.1266) TGF-β. The correlation analysis between each of the 7 biomarkers and the HMGB1 nuclear protein was quantified with the Pearson's correlation coefficient, the coefficient of determination and the probability reported as: NF-KB (r = - 0.4092, R^2 = 0.1675, *p* = 0.5908), TLR4 (r = - 0.9617, R^2 = 0.9248, *p* = 0.0383), pERK 1 / 2 /t-ERK 1/ 2, (r = -0.3336, R^2 = 0.1113, *p* = 0.7835), pJNK /t-JNK (r = -0.3090, R^2 = 0.09546, *p* = 0.8000), TGF-β (r = 0.5372, R^2 = 0.2885, *p* = 0.6390).<br><br>**MODEL 8**<br><br>Model 8 comprises of the **fibrotic biomarker model,** which includes the fibrosis percentage, collagen I and collagen III content in the cardiac tissue. Fibrosis of the heart wall causes damage to the cardiac tissue and collagen deposits in the cardiomyocytes, and the growth of fibroblasts increase the risk of manifesting DCM. The forest plot of fibrosis % included 4 studies in which the control and DCM groups consisted of 32 animals each. The forest plot displayed fibrosis % as having high risk of developing DCM in the rodents (SMD 3.25, 95%CI 0.77, 5.74, *p*=0.01) and the Collagen I (SMD 2.31, 95%CI 0.62, 3.99, *p* <0.007) and Collagen III (SMD 2.43, 95%CI 0.59, 4.27, *p* =0.01) content also served as high risk factors of DCM. The number of animals in the control and DCM groups each in the forest plot of Collagen I and Collagen III was 42. In the Mann Whitney U test, all 3 biomarkers showed no significant difference between the control and DCM groups: Fibrosis % (U= 1.0000, *p* = 0.0571), Collagen I (U=0.0000, *p* = 0.1), Collagen III (U = 0.0000,	40<br><br>42

		p = 0.1). The correlation analysis between each of the 3 biomarkers and the HMGB1 nuclear protein was quantified with the Pearson's correlation coefficient, the coefficient of determination and the probability are reported as: Fibrosis % (r = -0.2487, R^2 = 0.06187, *p* = 0.7513), Collagen I (r = 0.8384, R^2 = 0.7030, *p* = 0.3669), Collagen III (r = 0.9565, R^2 = 0.9149, *p* = 0.1885). The ROC of the Fibrosis % (AUC= 0.9375, *p*= 0.0433), Collagen I (AUC= 1.0000, *p*= 0.0039), and Collagen III (AUC= 1.0000, *p*= 0.0495) all showed a significant difference in the DCM group in comparison to control.<br><br>**RISK OF BIAS**<br><br>The SYRCLE tool evaluates the bias arising from confounding factors, the selection of participants, and deviations from the intended intervention, the handling of missing data, measurements of the outcomes and the method of reporting results with an overall assessment of bias in each study. All the eligible studies that were included in this meta-analysis did not show any risk of bias that in any of the parameters that were assessed in the SYRCLE ROB tool. All these studies were screened for any potential items of bias which yielded no confounders, randomized selection of the animals, direct identification of the interventions without deviating into unintended interventions which also had no missing data. Further, there were no grey areas regarding the measurement of the outcomes or in the reporting of the results obtained from a properly planned research study which had appropriate controls introduced at appropriate timepoints. Thus, the overall appraisal did not produce any concerns regarding any significant bias in the present study. In all the eligible studies, the risk of bias remained conspicuously low which validates the findings of our study. This was applicable to all the models that were investigated. For each included study, the ten criteria of the SYRCLE ROB tool were applied and assessed, ranking them into unclear or high or low ROB. These results were then summarized into a ROB summary and a table showing the individual assessments of the 29 included studies.	43
	20b	(see table below)	S3

Biomarker	TAU^2	Z Score	P< 0.05	Mann Whitney U Statistic	P<0.05	I squared Statistic	Chi-Squared Statistic	DF	P<0.05
**AGEs**	**12.29**	**2.88**	**0.004**	**3**	**0.7000**	**93**	**61.25**	**4**	**0.00001**
**HMGB1**	**3.00**	**4.15**	**0.0001**	**0**	**0.0286***	**91**	**102.82**	**9**	**0.00001**
**HR**	**110.18**	**1.73**	**0.08**	**2.5**	**0.5000**	**96**	**48.09**	**2**	**0.00001**
**HW**	**20.45**	**2.00**	**0.05**	**7**	**0.8857**	**95**	**57.44**	**3**	**0.00001**
**HW/BW**	**3.48**	**2.69**	**0.007**	**3**	**0.0152***	**87**	**38.96**	**5**	**0.0001**

			**EF%**	4.61	5.65	0.00001	20	0.0004*	87	89.69	12	0.00001		
			**FS%**	2.35	5.42	0.00001	28	0.0093*	80	56.34	11	0.00001		
			**LVIDD**	4.08	0.11	0.91	23	0.3128	90	68.88	7	0.00001		
			**LVIDS**	3.89	2.01	0.04	16.50	0.3281	88	50.80	6	0.00001		
			**LVSV**	5.49	0.98	0.33	4	>0.9999	93	27.96	2	0.00001		
			**CK-MB**	6.41	2.87	0.004	13	0.4848	89	61.15	7	0.00001		
			**C-TPN**	1056.98	2.21	0.03	6	0.6857	95	54.89	3	0.00001		
			**LDH**	10.59	3.49	0.0005	19	0.5350	88	51.04	6	0.00001		
			**BP**	4.08	1.39	0.17	3	0.7000	92	24.30	2	0.00001		
			**BG**	15.74	5.74	0.00001	9	<0.0001*	91	122.89	11	0.00001		
			**sINS**	162.42	1.02	0.31	18	0.4557	93	14.55	1	0.0001		
			**BW**	4.07	1.98	0.05	0	0.0286*	88	51.71	6	0.00001		
			**TC**	16.98	2.61	0.009	3	0.0476*	92	37.08	3	0.00001		
			**TG**	86.89	3.39	0.0007	5	0.4857	94	61.67	4	0.00001		
			**GSH**	10.00	2.62	0.009	15	0.2593	90	29.44	3	0.00001		
			**MDA**	5.22	4.52	0.00001	6	0.6856	79	28.10	6	0.0001		
			**TNF-A**	1.31	2.17	0.03	10	0.2229	82	33.10	6	0.0001		
			**IL-6**	9.50	5.01	0.00001	1	06667	92	126.82	10	0.00001		
			**IL-1β**	8.11	3.02	0.003	9	<0.0001*	93	55.03	4	0.00001		
			**NF-KB**	3.71	3.80	0.0001	0	0.0286*	88	48.12	6	0.00001		
			**TLR4**	3.45	2.63	0.009	3	0.2000	88	32.96	4	0.00001		
			**CC3**	5.73	3.13	0.002	-	-	89	36.29	4	0.00001		

NLRP3	10.26	2.13	0.03	-	-	94	47.41	3	0.00001
pERK1 /2/t-ERK 1 /2	1.49	0.80	0.42	3.5	0.8000	79	23.85	5	0.0002
pJNK/ t-JNK	0.53	3.99	0.0001	1.5	0.3000	49	7.85	4	0.10
TGF-β	2.12	1.92	0.05	1	0.2000	84	24.96	4	0.0001
FIB%	5.41	2.57	0.01	1	0.0571	89	26.47	3	0.00001
COL I	3.39	2.68	0.007	0	0.1	86	36.29	5	0.00001
COL III	4.41	2.59	0.010	0	0.1	88	42.48	5	0.00001

RISK FACTOR/ MODEL	BIOMARKER	EFFECT SIZE	P < 0.05	COHEN'S D 0.2 SMALL EFFECT 0.5 MEDIUM EFFECT >0.8 LARGE EFFECT
ADVANCED GLYCATION END PRODUCTS	AGEs	5.43	0.004	LARGE EFFECT
	HMGB1	3.0	0.0001	LARGE EFFECT
CARDIOMETABOLIC BIOMARKERS	HR	12.20	0.08	LARGE EFFECT
	HW	4.73	0.05	LARGE EFFECT
	HW/BW	2.31	0.007	LARGE EFFECT
	EF%	-4.13	0.00001	LARGE EFFECT
	FS%	-2.91	0.00001	LARGE EFFECT

33

				**LVIDd**	-	0.91	**SMALL EFFECT**		
				**LVIDs**	1.64	0.04	**LARGE EFFECT**		
				**LVDV**	4.93	0.00001	**LARGE EFFECT**		
				**LVSV**	-1.38	0.33	**LARGE EFFECT**		
				**CK-MB**	3.28	0.004	**LARGE EFFECT**		
				**CTPN**	37.93	0.03	**LARGE EFFECT**		
				**LDH**	5.66	0.0005	**LARGE EFFECT**		
				**BP**	1.71	0.17	**LARGE EFFECT**		
			**GLYCAEMIC BIOMARKERS**	**BG**	7.54	0.00001	**LARGE EFFECT**		
				**SINS**	-9.46	0.31	**LARGE EFFECT**		
				**BW**	-1.74	0.05	**MEDIUM EFFECT**		
			**LIPID BIOMARKERS**	**TC**	7.38	0.009	**LARGE EFFECT**		
				**TG**	16.60	0.0007	**LARGE EFFECT**		
			**OXIDATIVE STRESS BIOMARKERS**	**GSH**	-4.60	0.009	**LARGE EFFECT**		
				**MDA**	4.78	0.00001	**LARGE EFFECT**		
			**INFLAMMATORY BIOMARKERS**	**TNF-A**	1.11	0.03	**LARGE EFFECT**		
				**IL-6**	5.49	0.00001	**LARGE EFFECT**		
				**IL-1β**	4.44	0.003	**LARGE EFFECT**		
			**SIGNALLING PATHWAY BIOMARKERS**	**NF-KB**	3.13	0.0001	**LARGE EFFECT**		

	TLR4	2.38	0.009	LARGE EFFECT
	TGF-β	3.91	0.002	LARGE EFFECT
	CC3	3.59	0.03	LARGE EFFECT
	NLRP3		0.42	LARGE EFFECT
	pERK1 /2/ t-ERK 1 /2	1.87	0.0001	LARGE EFFECT
	pJNK/ t-JNK	1.50	0.05	LARGE EFFECT
FIBROSIS BIOMARKERS	FIBROSIS %	3.25	0.01	LARGE EFFECT
	Collagen I	2.31	0.007	LARGE EFFECT
	Collagen III	2.43	0.010	LARGE EFFECT

Cohen classified effect sizes as small (d = 0.2), medium (d = 0.5), and large (d ≥ 0.8).

	20c	There are 3 ways in which heterogeneity between the included studies was analysed, as per the Cochrane guidelines. They are (1) visual inspection of forest plots based on the individual effect sizes, (2) the Q statistic, and (3) the I squared statistic. According to the I squared statistic, the included studies in this meta-analysis have high heterogeneity because I squared metric greater than 70% is considered having high heterogeneity. It is also important to examine the magnitude and direction of its effect. In this case a random effects model was used, and the direction of the results was definitely favouring the T2D indicating high risk of DCM.	19

The Q statistic

RISK FACTOR	HIGHEST MEAN (A)	MEAN CLOSEST TO HIGHEST MEAN (B)	DIFFERENCE BETWEEN A AND B (C)	RANGE	DIVIDE (C) BY RANGE	Q STATISTIC	CRITICAL VALUE OF Q AT *P*=0.05	Q STATISTIC IS LESS THAN CRITICAL VALUE	ACCEPT/REJECT NULL HYPOTHESIS (MAX VALUE IS NOT AN OUTLIER
AGEs	88.16	12.96	75.20	87.66	75.20/ 87.66	0.8578	0.2763	More	AN OUTLIER
HMGB1	15.75	14.18	1.57	15.66	1.57/ 15.66	10.0255	0.2686	More	AN OUTLIER
HR	85.42	7.63	77.79	82.38	77.79/ 82.38	0.9442	0.3005	More	AN OUTLIER
HW	8.72	8.08	0.64	7.60	0.64/ 7.60	0.0842	0.2513	Less	NOT AN OUTLIER
HW/BW	8.96	4.96	4.00	8.83	4/8.83	0.4530	0.2288	More	AN OUTLIER
EF%	-0.88	-1.03	0.15	35.00	0.15/35	0.0042	0.1846	Less	NOT AN OUTLIER
FS%	-0.94	-1.03	0.09	10.74	0.09/ 10.74	0.0083	0.1876	Less	NOT AN OUTLIER
LVIDD	7.77	1.15	6.62	7.74	6.62/ 7.74	0.8552	0.2106	More	AN OUTLIER
LVIDS	6.75	6.65	0.10	6.60	0.10/ 6.60	0.0151	0.2273	Less	NOT AN OUTLIER
LVDV	4.93	4.09	0.84	-	-				
LVSV	3.94	1.17	2.77	2.99	2.77/ 2.99	0.9264	0.2725	More	AN OUTLIER

19

**CK-MB**	**253.44**	**153.61**	**99.83**	**252.11**	**99.83/ 252.11**	**0.3959**	**0.2302**	**More**	**AN OUTLIER**
**C-TPN**	**72.98**	**46.76**	**26.22**	**72.52**	**26.22/ 72.52**	**0.3615**	**0.2594**	**More**	**AN OUTLIER**
**LDH**	**75.89**	**56.67**	**19.22**	**74.43**	**19.22/ 74.43**	**0.2582**	**0.2257**	**More**	**AN OUTLIER**
**BP**	**5.36**	**0.74**	**4.62**	**4.99**	**4.62/ 4.99**	**0.9258**	**0.2655**	**More**	**AN OUTLIER**
**BG (mM)**	**23.48**	**21.26**	**2.22**	**23.42**	**2.22/ 23.42**	**0.0947**	**0.1881**	**Less**	**NOT AN OUTLIER**
**sINS**	**-0.75**	**-19.43**	**18.68**	**18.68**	**18.68/ 18.68**	**1.000**	**0.3293**	**More**	**AN OUTLIER**
**BW**	**113.49**	**0.68**	**112.81**	**3.98**	**112.81/ 3.98**	**28.34**	**0.2182**	**More**	**AN OUTLIER**
**TC**	**73.59**	**50.80**	**22.79**	**71.26**	**22.79/ 71.26**	**0.3198**	**0.2541**	**More**	**AN OUTLIER**
**TG**	**72.66**	**38.83**	**33.83**	**71.08**	**33.83/ 71.08**	**0.4759**	**0.2408**	**More**	**AN OUTLIER**
**GSH**	**-0.87**	**-12.11**	**11.24**	**11.24**	**11.24/ 11.24**	**1.0000**	**0.2622**	**More**	**AN OUTLIER**
**MDA**	**16.91**	**11.68**	**5.23**	**15.14**	**5.23/ 15.14**	**0.3454**	**0.2288**	**More**	**AN OUTLIER**
**TNF-A**	**3.40**	**1.39**	**2.01**	**3.31**	**2.01/3.31**	**0.6072**	**0.2408**	**More**	**AN OUTLIER**
**IL-6**	**7.40**	**4.74**	**2.66**	**7.28**	**2.66/7.28**	**0.3653**	**0.2257**	**More**	**AN OUTLIER**
**IL-1β**	**4.39**	**1.66**	**2.73**	**2.73**	**2.73/2.73**	**1.0000**	**0.3005**	**More**	**AN OUTLIER**

**NF-kB**	**7.0**	**2.17**	**4.83**	**6.42**	**4.83/ 6.42**	**0.7523**	**0.2513**	**More**	**AN OUTLIER**
**TLR4**	**7.51**	**2.91**	**4.60**	**7.36**	**4.60/ 7.36**	**0.625**	**0.2488**	**More**	**AN OUTLIER**
**CC3**	**1.55**	**1.32**	**0.23**	**0.23**	**0.23/ 0.23**	**1.0000**	**0.3293**	**More**	**AN OUTLIER**
**pERK1 /2/t-ERK 1 /2**	**1.27**	**1.04**	**0.23**	**1.31**	**0.23/ 1.31**	**0.1755**	**0.3005**	**Less**	**NOT AN OUTLIER**
**pJNK/ t-JNK**	**3.52**	**1.67**	**1.85**	**2.58**	**1.85/ 2.58**	**0.7170**	**0.3005**	**More**	**AN OUTLIER**
**TGF-β**	**2.55**	**0.81**	**1.74**	**2.27**	**1.74/ 2.27**	**0.7665**	**0.2725**	**More**	**AN OUTLIER**
**FIB%**	**5.84**	**5.22**	**0.62**	**5.47**	**0.62/ 5.47**	**0.1133**	**0.2541**	**Less**	**NOT AN OUTLIER**
**COL I**	**9.09**	**7.10**	**1.99**	**6.83**	**1.99/ 6.83**	**0.2913**	**0.2804**	**More**	**AN OUTLIER**
**COL III**	**6.14**	**5.57**	**0.57**	**2.09**	**0.57/ 2.09**	**0.2727**	**0.2804**	**More**	**AN OUTLIER**

	20d	Four studies which had an effect size of 0.00 were removed from the forest plot on SBP (given on the right). It changed the original SMD of 0.41 at 95% confidence interval (0.29, 0.53) to a more robust SMD of 0.45 (0.33, 0.57).	25

<table>
<tr><td></td><td></td><td></td><td></td></tr>
<tr><td>REPORTING BIASES</td><td>21</td><td>
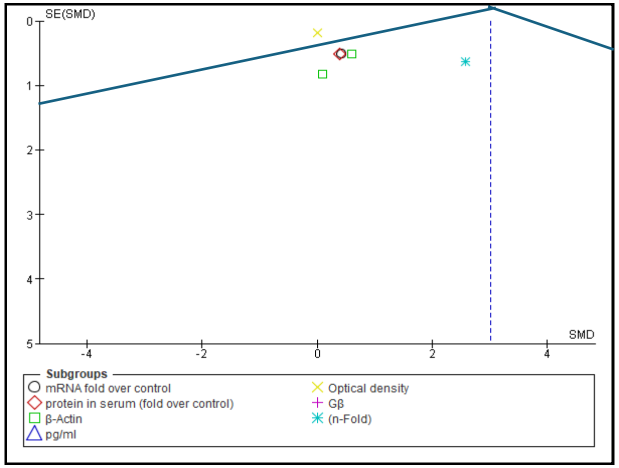

Fig. S2-1 HMGB1
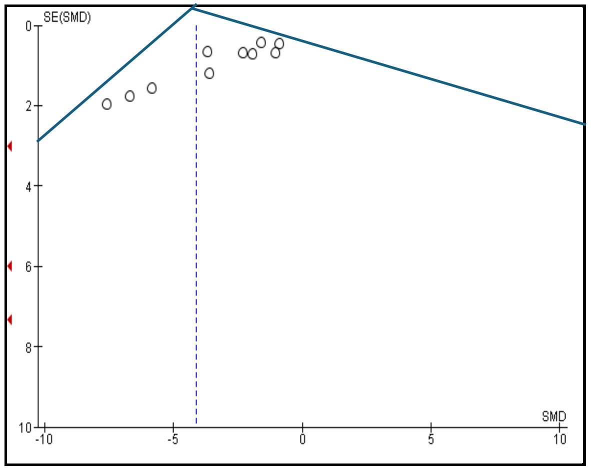

Fig. S2-2 EF%
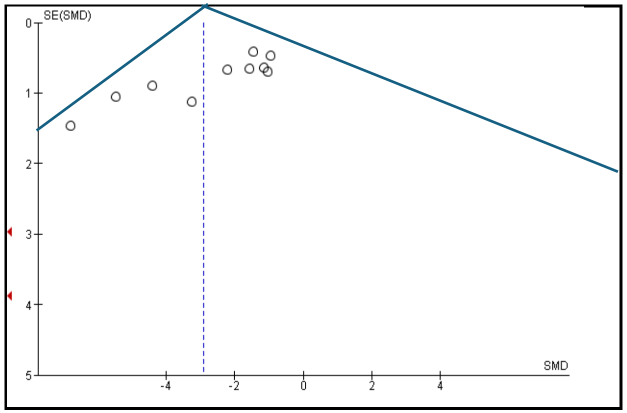

Fig. S2-3 FS%
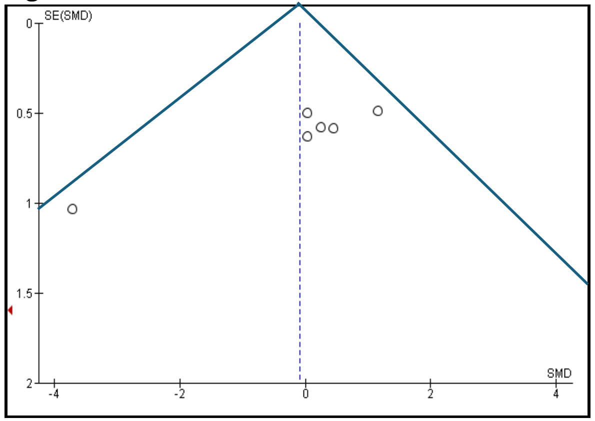

Fig. S2-4 LVIDD
</td><td>S2
43</td></tr>
</table>

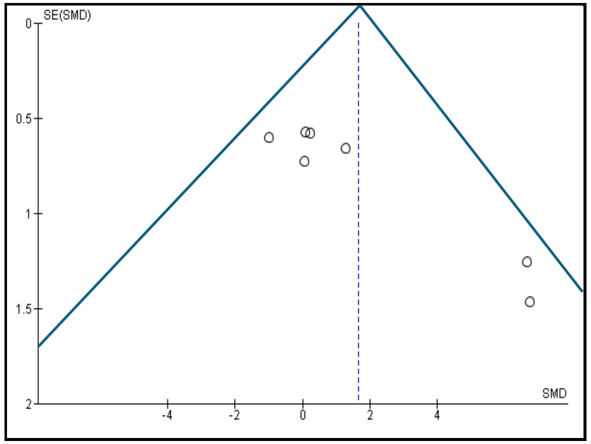

Fig. S2- 5 LVIDS

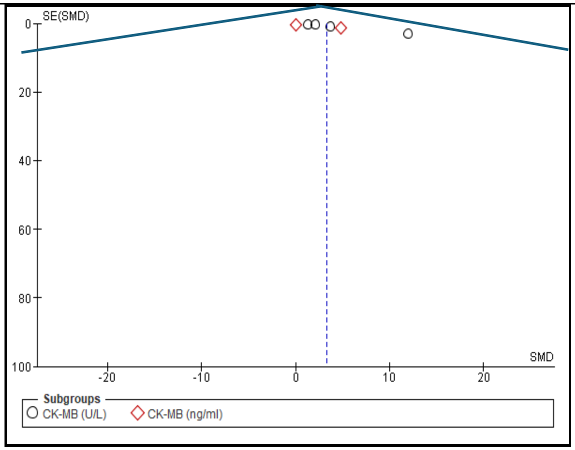

Fig. S2-6 CK-MB

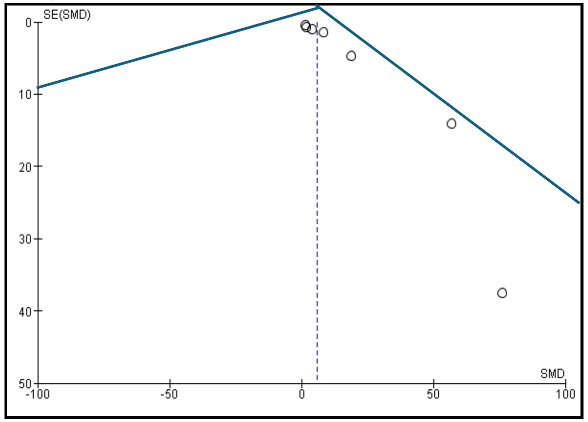

Fig. S2- 7 LDH

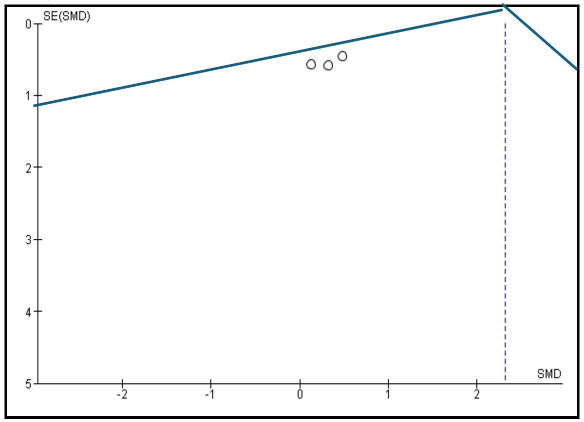

Fig. S2-8 HW/BW

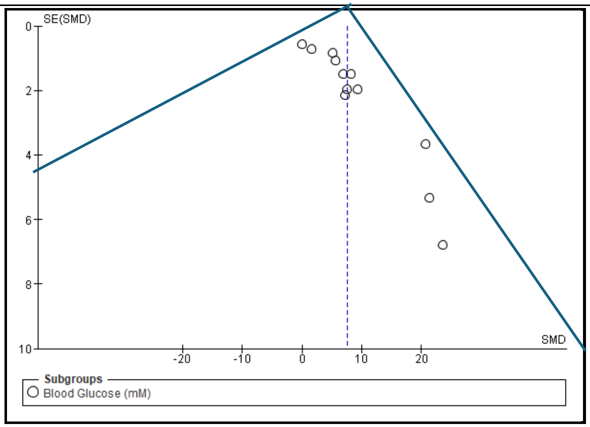

Fig. S2-9 BG

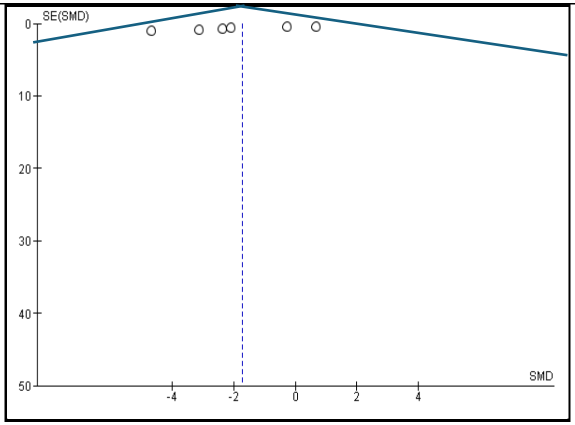

Fig. S2 -10 BW

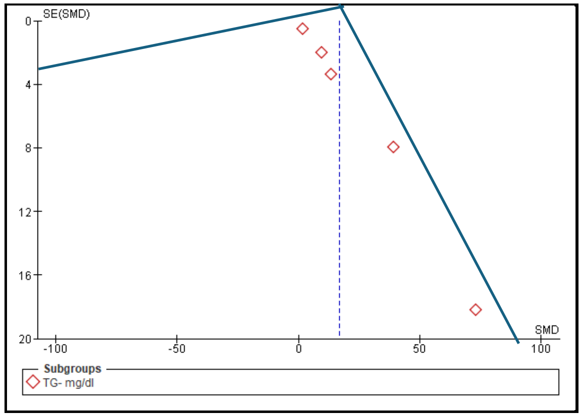

Fig. S2-11 TG

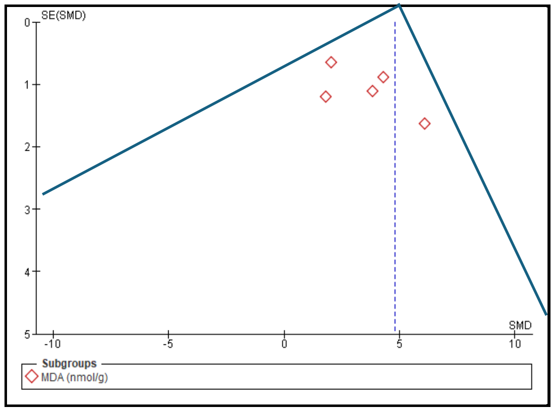

Fig. S2- 12 MDA

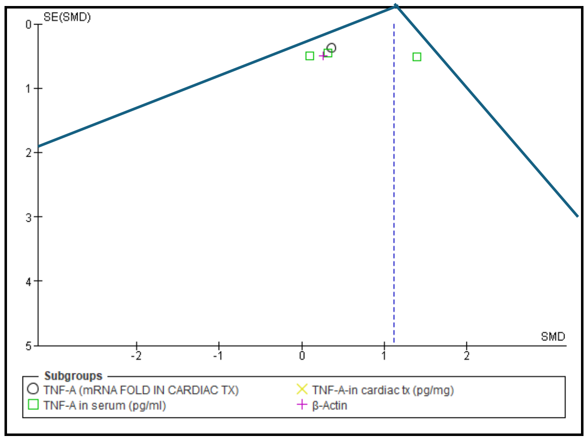

Fig. S2-13 TNF-A

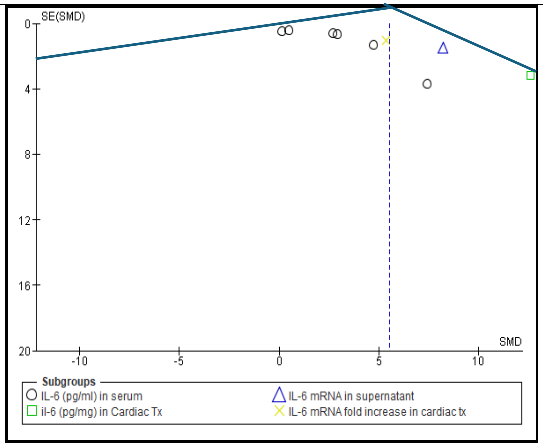

Fig. S2-14 IL-6

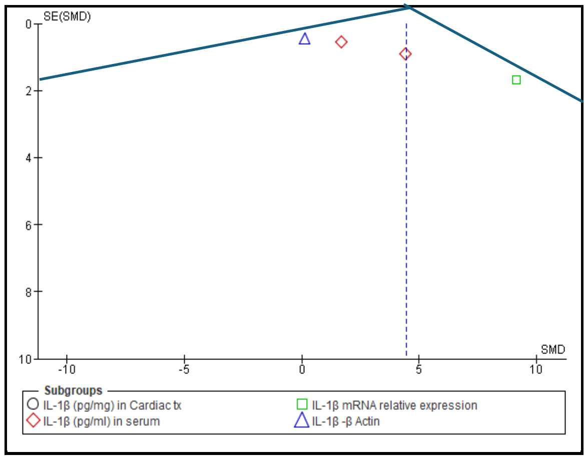

Fig. S2-15 IL-1β

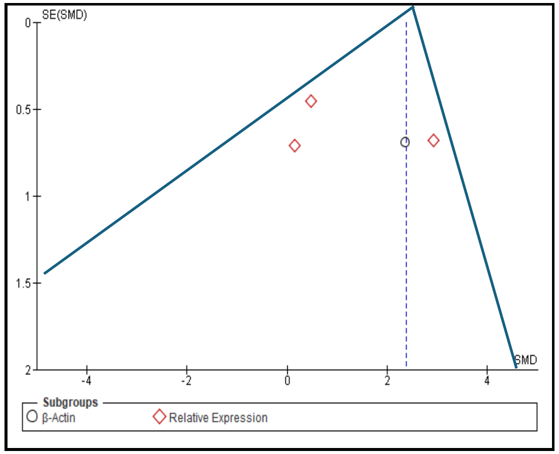

Fig. S2-16 TLR4

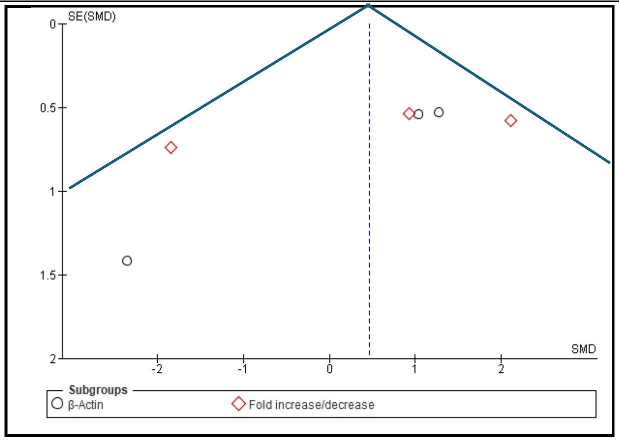

Fig. S2-17 p-ERK 1/ 2 /t-ERK 1/ 2

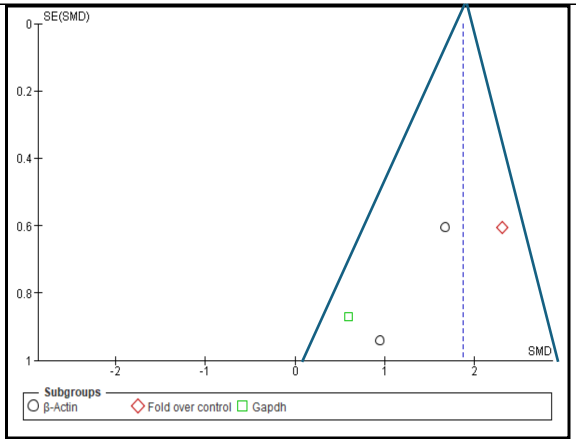

Fig. S2-18 p-JNK / t-JNK

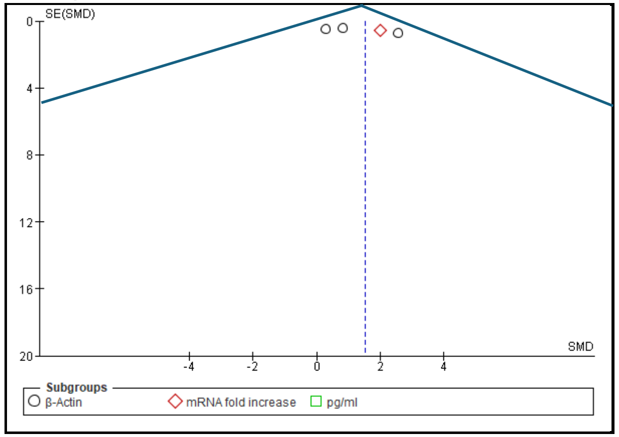

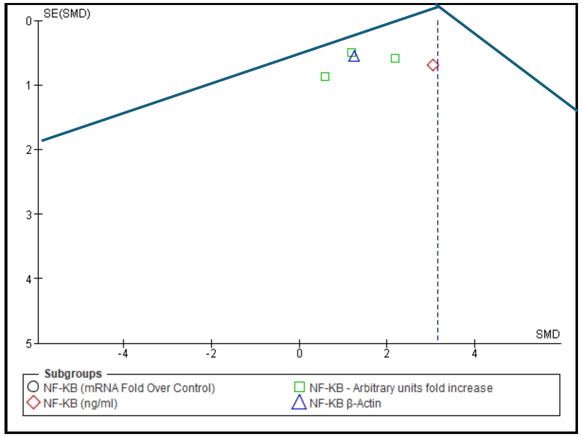

Fig. S2-19 TGF-β

Fig. S2-20 NF-KB

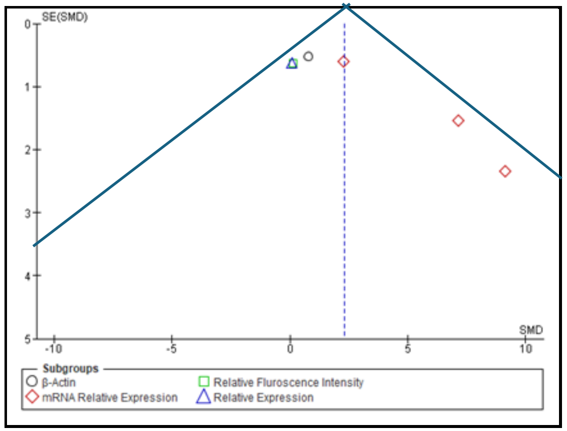

Fig. S2-21 Col III

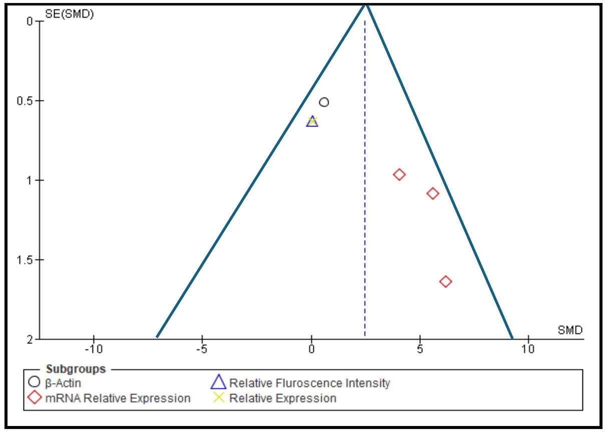

Fig. S2-22 Col I

CERTAINITY OF EVIDENCE 22

Outcome/Bio marker	Standard<br><br>Normal for HC	Mean HC of present study	Mean T2D obtained from present study	Reference
**AGEs**	1 to 30 μg/mL	13	22	1
**HMGB1**	50–300 pg/mL	325	418	2
**HR**	450 to 750 bpm	241	319	3
**HW**	100 – 200 g	374	608	4
**HW/BW**	4.5 – 5.5 mg/g BW	3.19	5.47	5
**EF%**	6O-70%	74%	51.7%	6

**FS%**	25-42%	47.8%	31.8%	6
**LVIDD**	3-4 mm	2.62	15.9	7
**LVIDS**	1.5-2.5 mm	1.55	2.7	8
**LVDV**	70–90 microliters (µL)	41.6	61.3	8
**LVSV**	40 to 45 microliters (µL)	10.25	22.76	9
**CK-MB**	50 to 200 IU/L	124.8	240.66	10
**C-TPN**	15 -20 pg/mL	10	35	11
**LDH**	1,000–2,000 IU/L	420.25	780.16	12
**BP**	100-122 Hg mm	112	131.31	13
**BG (mM)**	4-7.5 mmol/L	6.49	22.97	14
**BG (mg/dl)**	72-135 mg/dL	237.7	382.4	14
**sINS**	0.5 – 1.5 ng/mL	3.16	12.5	15
**BW**	22-35 g	109.6	124	16
**TC**	90-150 mg/dL	55	200	17
**TG**	80-120 mg/dL	55	175	17
**GSH**	7–10 µmol/g tissue	1.02	0.5	18
**MDA**	1-2 nmol/mg	3.55	10.2	19
**TNF-A**	5-20 pg/mL	25.6	61.6	20
**IL-6**	2-15 pg/mL	6	64.2	21
**IL-1β**	2-10 pg/mL	39.3	93.3	22
**NF-kB**	Too low to be estimated in tissues or serum	0.28	0.62	23

**TLR4**	Too low to be estimated in tissues or serum	1	3.5	24
**NLRP3**	Too low to be estimated in tissues or serum	9.8	43.9	25
**pERK1 /2/t-ERK 1 /2**	0.15-0.3	0.725	2.57	26
**p-JNK/ t-JNK**	0.2 -0.3	1	4.5	27
**TGF-β**	2000-3000 pg/mL	1	5.5	28
**FIB%**	0%	5.25%	27.75%	29
**COL I**	14.5%	1.1%	5.56%	30
**COL III**	3.2%	1%	14%	31

References

1 https://doi.org/10.1053/j.jrn.2023.09.005

2 Valdes-Ferrer et al., 2014 – *"HMGB1 is a key mediator of anaemia in a murine model of sepsis survivors"*, published in *The Journal of Immunology*

3 Kramer et al., 1993, as discussed in the review by **Calvet & Seebeck (2023)** – *"What to consider for ECG in mice with special emphasis on telemetry"*, published in *Mammalian Genome*

4 Orr-Choward Rodent Blood Collection Guidelines provide body weight benchmarks that help contextualize organ weights

5 Sugihara et al., 2013 – *"Usefulness of Running Wheel for Detection of Congestive Heart Failure in Dilated Cardiomyopathy Mouse Model"*, published in *PLOS ONE*

6 Vinhas et al., 2013 – *"Transthoracic echocardiography reference values in juvenile and adult 129/Sv mice"*, published in *Cardiovascular Ultrasound*

7 Kane AE, Bisset ES, Heinze-Milne S, Keller KM, Grandy SA, Howlett SE. Maladaptive Changes Associated With Cardiac Aging Are Sex-Specific and Graded by Frailty and Inflammation in C57BL/6 Mice. J Gerontol A Biol Sci Med Sci. 2021 Jan 18;76(2):233-243. doi: 10.1093/gerona/glaa212. PMID: 32857156; PMCID: PMC7812442.

8 Zacchigna S, Paldino A, Falcão-Pires I, Daskalopoulos EP, Dal Ferro M, Vodret S, Lesizza P, Cannatà A, Miranda-Silva D, Lourenço AP, Pinamonti B, Sinagra G, Weinberger F, Eschenhagen T, Carrier L, Kehat I, Tocchetti CG, Russo M, Ghigo A, Cimino J, Hirsch E, Dawson D, Ciccarelli M, Oliveti M, Linke WA, Cuijpers I, Heymans S, Hamdani N, de Boer M, Duncker DJ, Kuster D, van der Velden J, Beauloye C, Bertrand L, Mayr M, Giacca M, Leuschner F, Backs J, Thum T. Towards standardization of echocardiography for the evaluation of left ventricular function in adult rodents: a position paper of the ESC Working Group on Myocardial Function. Cardiovasc Res. 2021 Jan 1;117(1):43-59. doi: 10.1093/cvr/cvaa110. PMID: 32365197.

9 Brojakowska A, Jackson CJ, Bisserier M, Khlgatian MK, Grano C, Blattnig SR, Zhang S, Fish KM, Chepurko V, Chepurko E, Gillespie V, Dai Y, Lee B, Garikipati VNS, Hadri L, Kishore R, Goukassian DA. Lifetime Evaluation of Left Ventricular Structure and Function in Male C57BL/6J Mice after Gamma and Space-Type Radiation Exposure. Int J Mol Sci. 2023 Mar 13;24(6):5451. doi: 10.3390/ijms24065451. PMID: 36982525; PMCID: PMC10049327.

10 Kanneboyina Nagaraju Heather Gordish: George Carlson (Dept. Physiology, Kirksville College of Osteopathic Medicine
Kirksville, MO. 63501-1497, USA)
11 Löwbeer C, Forsberg AM, Tokuno S, Hemdahl AL, Gustafsson SA, Valen G. Cardiac troponin T content in heart and skeletal muscle and in blood samples from ApoE/LDL receptor double knockout mice. Clin Chim Acta. 2004 Jun;344(1-2):73-8. doi: 10.1016/j.cccn.2004.02.006. PMID: 15149873.

12 Okulmus, C., Icil, N.I., Turkyilmaz, O. *et al.* Clinical chemistry reference intervals for swiss albino strain mice commonly used in scientific studies. *Comp Clin Pathol* **34**, 567–574 (2025). https://doi.org/10.1007/s00580-025-03683-w

13 David L. Mattson, Comparison of arterial blood pressure in different strains of mice, *American Journal of Hypertension*, Volume 14, Issue 5, May 2001, Pages 405–408, https://doi.org/10.1016/S0895-7061(00)01285-1

14 Kangudia Mbaya, J., Mbanzulu Pita Nsonizau, D., Mbanzulu Nsolani, N. and Zosuruna Mbuy, T. (2023) Blood Glucose Reference Interval in Mus musculus Mice. Open Access Library Journal, 10: e9988. https://doi.org/10.4236/oalib.1109988

15 UQ Animal Ethics Committee - Standard Operating Procedure LAB_057 Insulin Tolerance Test in Mice Institutional author: Research Ethics and Integrity AEC Reviewed & Approved: 01/12/2022

16 Jackson laboratory research centre, USA (2025) [WWW.JAX.ORG]

17 DLAM | Pathology & Laboratory Medicine Services David Geffen School of Medicine at UCLA 650 Charles E. Young Drive South, CHS:5V-109 Los Angeles, CA 90095-1718 ph: (310) 206-8120

18 Vuolo, M.M., da Silva-Maia, J.K., Batista, Â.G. (2022). The GSH Colorimetric Method as Measurement of Antioxidant Status in Serum and Rodent Tissues. In: Betim Cazarin, C.B. (eds) Basic Protocols in Foods and Nutrition. Methods and Protocols in Food Science . Humana, New York, NY. https://doi.org/10.1007/978-1-0716-2345-9_12

		**19** Malaysian Journal of Medicine and Health Sciences (eISSN 2636-9346)<br><br>**20** ©2018 Thermo Fisher Scientific Inc.<br><br>**21** Feng Q, Li CZ, Zou YH, Wang XY, Yang X, Zhang R, Liu ZQ, Zhang RR. IL6/CCL2 from M2-polarized microglia promotes breast cancer brain metastasis and the reversal effect of β-elemene. Front Pharmacol. 2025 May 15;16:1547333. doi: 10.3389/fphar.2025.1547333. PMID: 40444044; PMCID: PMC12119470.<br><br>**22** Promega Corporation · 2800 Woods Hollow Road · Madison, WI 53711-5399 USA · Toll Free in USA 800-356-9526 · 608-274-4330 · Fax 608-277-2516 TM646 ·<br><br>**23** J Clin Invest. 2001;107(1):7-11. https://doi.org/10.1172/JCI11830.<br><br>**24** Vaure C, Liu Y. A comparative review of toll-like receptor 4 expression and functionality in different animal species. Front Immunol. 2014 Jul 10;5:316. doi: 10.3389/fimmu.2014.00316. PMID: 25071777; PMCID: PMC4090903.<br><br>**25** Kelley N, Jeltema D, Duan Y, He Y. The NLRP3 Inflammasome: An Overview of Mechanisms of Activation and Regulation. Int J Mol Sci. 2019 Jul 6;20(13):3328. doi: 10.3390/ijms20133328. PMID: 31284572; PMCID: PMC6651423.<br><br>**26** Int J Clin Exp Med 2019;12(5):5069-5077 www.ijcem.com /ISSN:1940-5901/IJCEM0088928<br><br>**27** Wang, J., An, F.S., Zhang, W. *et al.* Inhibition of c-Jun N-Terminal Kinase Attenuates Low Shear Stress-Induced Atherogenesis in Apolipoprotein E-Deficient Mice. *Mol Med* **17**, 990–999 (2011). ttps://doi.org/10.2119/molmed.2011.00073<br><br>**28** Khan, S.A., Joyce, J. & Tsuda, T. Quantification of active and total transforming growth factor-β levels in serum and solid organ tissues by bioassay. BMC Res Notes 5, 636 (2012). https://doi.org/10.1186/1756-0500-5-636<br><br>**29** https://www.biocompare.com/Editorial-Articles/616968-A-Guide-to-Fibroblast-Markers<br><br>**30** Scientific Reports | (2023) 13:4490 | https://doi.org/10.1038/s41598-023-31566-z<br><br>**31** Jianping Gao, Zhenhu Guo, Yang Zhang, Yuying Liu, Fangyu Xing, Junjie Wang, Xi Luo, Yingjun Kong, Guifeng Zhang, Age-related changes in the ratio of Type I/III collagen and fibril diameter in mouse skin, *Regenerative Biomaterials*, Volume 10, 2023, rbac110, https://doi.org/10.1093/rb/rbac110	
**DISCUSSION**			
	23a	The high mobility group box 1 or HMGB1 is a nuclear protein which consists of a spectrum of metabolic properties in persons diagnosed with pre-clinical diabetes. CVD manifests consequent to the development of diabetes which first originates as endothelial dysregulation (ED) in the blood vessels. We have carried out a systematic review and a meta-analysis which pooled the data from 29 included primary studies that investigated the effect of 37 biomarkers on HMGB1 that are related to type 2-diabetes induced-ED in rodents including mouse and rat. HMGB1 is widely known as an inflammatory	4-6

		and angiogenic cytokine that is associated with the immune marker NF-kB, TLR4 and several more which increases its inflammatory properties that could be utilized in the treatment of T2D-induced DCM.	
	23b	There are many limitations to this study given the fact that out of screening over 2700 publications, only 29 qualified as included studies. There were studies which did not specify the subtype of diabetes, neither did they mention whether their control group consisted of healthy participants. Also, there were studies that did not have a clearcut disease subtype but mentioned that their study participants included some T1D and some T2D -induced animals. They gave the total numbers in each subtype that was included but did not measure the biochemical and demographic data into two separate groups. The second foremost limitation was not having enough studies to represent a particular type of outcome measure like having only 2 studies or just one study reporting the data which did not satisfy the power calculation in terms of the number of included studies. However, those single studies were included in a forest plot each given the fact that the number of participants exceeded 6-8 in total. There were discrepancies in measurements because the mean value was not accompanied by the standard deviation value where the length of the error bars was physically measured/estimated when displayed on bar graphs or on single data (dot) plots related to the SEM from which the SD was calculated by multiplying the length in SI units, usually in mm, by the square root of the number of animals in the given study. When the interquartile range was given, the SD was calculated by dividing the IQR by the square root of the number of animals. Most of the studies screened were cross sectional studies in which both the control and diseased /intervention groups had diagnosed diabetes, or many studies evaluated the effect of some medication on the disease progression which did not conform to our PICOS statement. The reliability of the outcomes was not supported by having smaller sample size, not having done repeats, adoption of different methodologies to evaluate the same outcome and presenting certain outcomes as percentages which cannot be compared between the control and the intervention groups which were some weaknesses encountered in the included studies. Some studies were retrospective studies from which data had been gathered several years ago.	60
	23c	There are limitations also in the review process such as in retrieving full-text articles of eligible research studies from electronic databases. In some instances, the authors did not respond to our requests for full-text reports or data that are missing such as the standard deviation.	60
	23d	The forest plot, the Mann Whitney U test, and the ROC have identified HMGB1 to be a biomarker which carries significant results out of the 37 biomarkers evaluated in this study. More research studies are nevertheless needed to elevate HMGB1 to the level of the cytokine biomarkers which are already in clinical use but offers hope to the drug manufacturers in the pharmaceutical industry. Further, customised treatment strategies could be revised with adjusting the dose and frequency of the insulin intake. One of the latest findings on the impact of HMGB1 on diabetes is the modulation of the gut microbiome to regulate the inflammatory activity of HMGB1 which has been proven successful in a mouse model, and the outcome of this study will further enhance its utility value. The manipulation of the different immune pathways that influence the immune activity of HMGB1 as a molecule which enhances the responses of immune molecular inhibitors in cancer studies, it may also lead towards understanding the role of HMGB1 in T2D-induced DCM. Additionally, investigating the proteomics, transcriptomes and epigenetic factors will add more value to this preliminary screening of the biomarkers into using them as either a diagnostic marker or a therapeutic target for DCM as future studies.	61
**OTHER INFORMATION**			

**REGISTRATION AND PROTOCOL**	24a	The meta-analysis was registered at the PROSPERO register in January 2023 (Available from: https://www.crd.york.ac.uk/prospero/ CRD. “Risk factors of diabetic cardiomyopathy in rodent models with relevance to HMGB1 as a therapeutic target: Systematic Review and Meta-Analysis. 2024 CRD42024597641 (Ongoing)”	
	24b	The protocol for this study is available in the PROSPERO website.	
	24c	The amendments to the protocol have not been uploaded yet. It will be done in due course, soon.	
**SUPPORT**	25	No financial support was received for this study.	1
**COMPETING INTERESTS**	26	The authors do not have competing interests for this study.	1
**AVAILABILITY OF DATA, CODE AND OTHER MATERIAL**	27	Since this meta-analysis is not published yet, none of the materials mentioned is not publicly available yet. Any of these materials can be obtained by contacting the first author *via* email for correspondence.	1
